# Catalytic Nitrous Oxide Degradation with Group 15 Clusters

**DOI:** 10.1021/jacs.5c09618

**Published:** 2025-08-06

**Authors:** Bono van IJzendoorn, Reece Lister-Roberts, Nikolas Kaltsoyannis, Meera Mehta

**Affiliations:** † Department of Chemistry, 6396 University of Oxford, 12 Mansfield Road, Oxford OX1 3QR, U.K.; ‡ Department of Chemistry, 5292 University of Manchester, Oxford Road, Manchester M13 9PL, U.K.

## Abstract

Nitrous oxide (N_2_O) is sometimes referred to as the forgotten greenhouse gas, but ignoring it would be a mistake. N_2_O has a greenhouse warming potential 300× that of CO_2_, and anthropogenic emissions are increasing. Yet, compared to CO_2_, homogeneous catalysts that mediate its reduction are scarce. We present a range of cluster catalysts based on abundant and inexpensive p-block elements that mediate the conversion of N_2_O to environmentally benign N_2_. The catalysts studied offer many critical advantages, and systems can be tuned for performance, recyclability, selectivity, air stability, and commercial availability. Pnictogen clusters present themselves as a general platform in N_2_O reduction chemistry, and control reactions confirm that these clusters offer access to reactivity that simple monopnictogen molecules do not. Mechanistic investigations reveal that the low-valent clusters can access a –1/+1 redox couple, which goes beyond classical main group redox couples and will unlock a vault of hitherto unknown chemical space.

## Graphical abstract

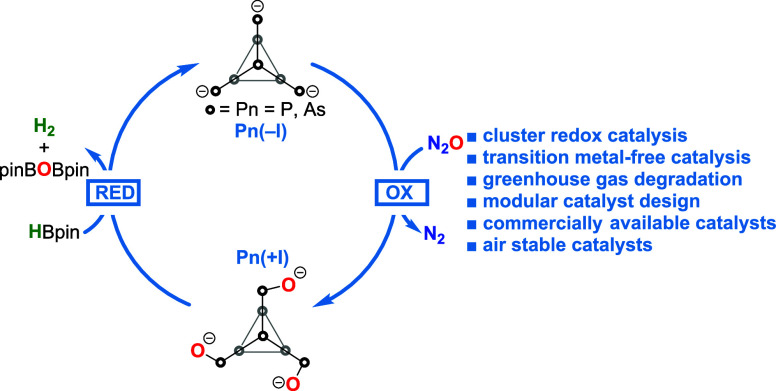

## Introduction

The presence of nitrous oxide (N_2_O) in the atmosphere is no laughing matter. N_2_O is a leading contributor to ozone depletion, with an estimated greenhouse warming potential 300× greater than CO_2_ and a lifetime of ∼114 years.^1^ Anthropogenic emission of N_2_O has been increasing by approximately 0.2% per annum,^2^ and one remediation strategy is to convert N_2_O into N_2_. Chemically, transfer of O and generation of N_2_ is thermodynamically favorable; nature does this in microbial denitrification processes.^3^ However, due to its high kinetic inertness, only a handful of synthetic homogeneous catalysts, primarily reliant on transition metals,^4–7^ have been found to mediate N_2_O degradation (Figure 1a).

**Figure 1:**
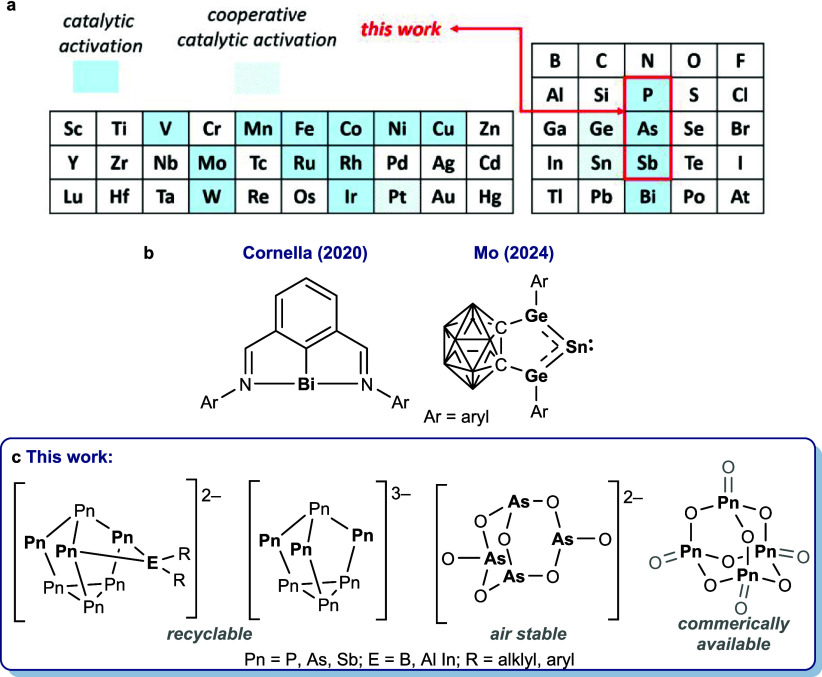
(a) Homogeneous catalysts based on these elements mediate N_2_O reduction. (b) Molecular structures of reported p-block catalysts that deoxygenate N_2_O. (c) General structures of the cluster catalysts studied in this work.

There has been global interest in developing organo-main-group catalysts as sustainable alternatives to transition metal systems. And although numerous p-block compounds, including frustrated Lewis pairs (FLPs) and carbenes,^8–10^ have been reported to capture and cleave the N–O bond of N_2_O stoichiometrically, catalytic degradation remains a significant challenge. One early example of transition metal-free homogeneous N_2_O reduction was reported by Cantat and coworkers in 2019, with fluoride anion as a (pre)­catalyst.^11^ Then, Cornella reported a Bi­(I) species that mediates N_2_O deoxygenation via a 2e^–^ Bi (I)/Bi­(III) redox cycle (Figure 1b).^12^ And, in 2024, Mo et al. reported an Sn complex that mediates this reduction through cooperativity with neighboring Ge atoms.^13^ These precedents demonstrate that low oxidation state main group compounds interact with N_2_O to be oxidized and release N_2_, but in order to achieve catalytic turnover, the generated oxidized product must be susceptible to reduction. This reduction is a key limitation in explaining why lighter main group systems have previously eluded N_2_O reduction catalysis. For example, phosphines react with N_2_O to give phosphine oxides, where the phosphorus +5 oxidation state is highly stable.^8,9^

Although a new field, there is growing interest in employing main group clusters in catalysis,^14^ in part because clusters can be understood as intermediaries between discrete molecules and difficult-to-study bulk solids—allowing them to act as soluble, atom-precise molecular prototypes. Compared to homogeneous systems, heterogeneous catalysts are often preferred for high-volume industrial processes, given their greater propensity for recycling and easy separation, which minimizes downstream purification costs.^15^ Heterogeneous pnictogens like red phosphorus, violet phosphorus, and black arsenic are up-and-coming candidates for catalysis, but their poorly defined structures (which contain clusters),^16^ together with extreme insolubility, make understanding the reactivity of these materials difficult. Meanwhile, heptapnictogen clusters ([Pn_7_]; Pn = pnictogen, structure shown in Figure 1c) are easily synthesized and, owing to their solubility in common solvents, in situ monitoring of reactivity is relatively easy. It is also noteworthy that, in 2024, Su and Zhang found that black phosphorus alloyed with Li contained [P_7_] cages.^17^ And in the context of N_2_O chemistry, Mao et al. have detected N_2_O interactions with violet phosphorus in sensor materials.^18^ Further understanding is necessary to maximize the potential of pnictogen-based materials, both as catalysts and otherwise.

[Pn_7_]^3–^ clusters present themselves as interesting candidates in N_2_O reduction chemistry as the bridging Pn atoms of the norticycle-type cage can be understood to have an oxidation state of −1 and a 2e^–^ oxidation would convert them to +1; avoiding the +5 state.^19^ With a primary focus on the [Pn_7_] frameworks, herein, we interrogate a range of pnictogen clusters to mediate the hydroborative reduction of N_2_O. Mechanistic investigations show that [P_7_]^3–^ can tap into a hitherto unknown −1/+1 redox couple. Other key findings are that the classic boron–phosphorus FLPs previously reported in N_2_O stoichiometric activation,^8^ and simple R_3_Pn molecules do not mediate N_2_O reduction, showing that these clusters enable chemistry that noncluster monopnictogen molecules cannot access. The arsenic systems were found to be the most catalytically active, and in recycling studies, catalytic competency was retained after nine cycles. Further, beyond the transfer of the N_2_O oxygen atom to boron, we establish that these oxygen atoms can be transferred to sulfur, constructing the first steps toward making sought-after element–oxygen bonds using N_2_O with transition metal-free catalysis. To demonstrate the reduction of other N–O bonds, nitro-functionalized organic compounds were converted to amines, which are high-value products in and of themselves for the pharmaceutical and agrochemical sectors.^20^ These are unprecedented transformations in transition metal-free cluster chemistry.

## Results and Discussion

We have previously reported on a library of group 13 functionalized heptapnictogen clusters in the reduction of CO_2_ (isoelectronic with N_2_O), with the general molecular formula [M­(18c6)]_2_[(R_2_E)­Pn_7_] (18c6 = 18-crown-6; see Figure 2a for dianion structures): [Na­(18c6)]_2_[(BBN)­P_7_] ([Na­(18c6)]_2_[**1**]; BBN = 9-borabicyclo[3.3.1]­nonane); [Na­(18c6)]_2_[(*i*Bu_2_Al)­P_7_] ([Na­(18c6)]_2_[**2**]); [Na­(18c6)]_2_[(Ph_2_In)­P_7_] ([Na­(18c6)]_2_[**3**]); and [K­(18c6)]_2_[(*i*Bu_2_Al)­As_7_] ([K­(18c6)]_2_[**4**]).^21,22^ Our initial studies focused on the reduction of N_2_O with this family of functionalized [Pn_7_] clusters, as well as the unfunctionalized [Pn_7_]^3–^ clusters, [K­(18c6)]_3_[P_7_] ([K­(18c6)]_3_[**5**]) and [K­(18c6)]_3_[As_7_] ([K­(18c6)]_3_[**6**]) from which they are prepared.^21–23^

**Figure 2:**
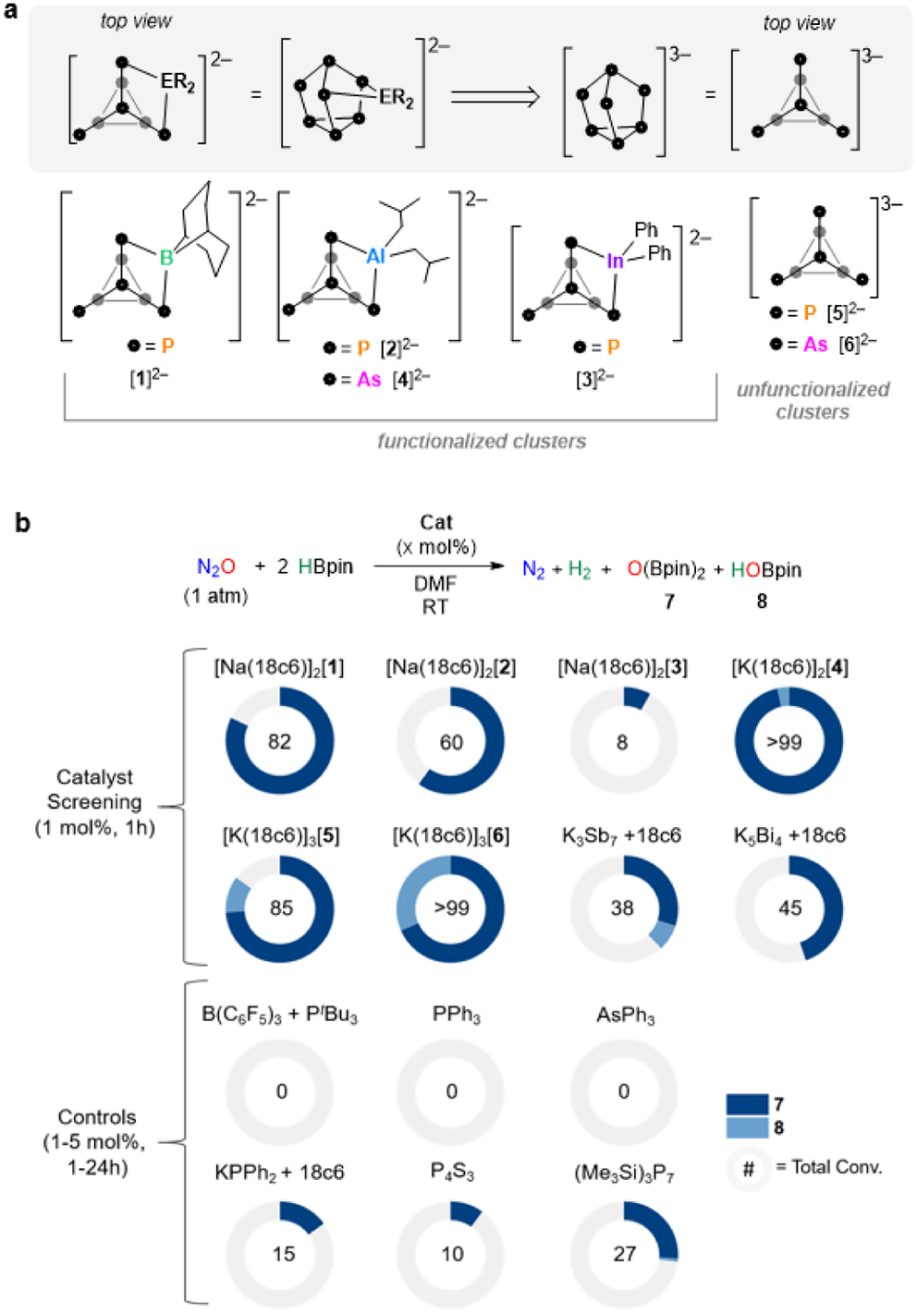
(a) Homogeneous catalysts studied that are based on the [Pn_7_] framework. (b) Catalyst screening and control reactions. Full data tables are provided in the Supporting Information 2.5 and 2.6. NMR conv. was determined by ^1^H NMR spectroscopy using C_6_H_6_ as an internal standard. For [Na­(18c6)]_2_[**1**], [Na­(18c6)]_2_[**2**], and KPPh_2_ + 18c6, background DMF hydroboration was observed, and conversions were corrected to exclude this side reaction.

First, the reduction of 1 atm of N_2_O using [Na­(18c6)]_2_[**1**] at 3.33 mol % catalyst loading with different reductants and in different solvents was screened (Supporting Information 2.1 and 2.2). Pinacol borane (HBpin) as the reductant and dimethylformamide (DMF) as the solvent gave the best results, with >99% conversion to O­(Bpin)_2_ (**7**) and H_2_, observed by ^1^H and ^11^B nuclear magnetic resonance (NMR) spectroscopy. Observation of H_2_ and **7** is in line with the hydroborative catalytic degradation of N_2_O.^6,11–13^ In these reactions, the internal nitrogen of N_2_O has been reduced to N_2_ (confirmed by ^15^N NMR studies, Supporting Information 2.3), and the hydride on the borane has been oxidized to H_2_, confirmed by D_2_ observed in the ^2^H NMR spectrum when DBpin was employed (Supporting Information 2.4).

Using the optimized conditions (DMF solvent, HBpin reducing agent, and 1 atm of N_2_O), the family of [M­(18c6)]_2_[(R_2_E)­Pn_7_] and the unfunctionalized [K­(18c6)]_3_[Pn_7_] (Pn = P, As) clusters (structures [**1**]^2^–[**4**]^2^ and [**5**]^3–^-[**6**]^3–^ shown in Figure 2a) were surveyed at 1 mol % catalyst loading (Figure 2b). First, it was confirmed that, in the absence of a catalyst, no conversion is observed. Then, at 1 mol % [Na­(18c6)]_2_[**1**], a conversion of 82% to **7** after 1 h was obtained. Under the same reaction conditions, catalysts [Na­(18c6)]_2_[**2**] and [Na­(18c6)]_2_[**3**] gave more sluggish performances. While [K­(18c6)]_2_[**4**] was found to be the most efficient, with complete consumption of the borane observed after 1 h and a product distribution of 96% **7** and 3% HOBpin (**8**). Next, the unfunctionalized clusters were tested in a similar fashion, and for [K­(18c6)]_3_[**5**], an overall conversion of 85% was observed, while [K­(18c6)]_3_[**6**] gave complete conversion. These observations suggest that the catalytic competency is primarily directed by the cluster core rather than the *exo*-group 13 functionality, contrasting our previous studies of these clusters in CO_2_ reduction, where catalytic performance was more significantly influenced by the *exo*-functional group.^21^ We have previously found that, when testing Zintl phases in solution catalysis, the addition of a cation-sequestering agent increases performance, seemingly by increasing the clusters’ solubility.^24^ Thus, the phases K_3_Sb_7_ and K_5_Bi_4_ were also probed, but with excess 18c6 under identical reaction conditions, and although worse-performing did demonstrate catalytic turnover. Although the phosphorus systems show good catalytic performance, the arsenic clusters were found to be the most active.

A series of control reactions were performed to better understand the origin of catalytic turnover; Figure 2b shows selected controls, with others provided in the Supporting Information 2.6. Previously, the FLP combination B­(C_6_F_5_)_3_ and ^*t*^Bu_3_P had been reported to stoichiometrically activate N_2_O,^8^ but when this FLP was trialed in catalytic quantities with HBpin, no catalytic turnover was achieved. Further, Ph_3_P and Ph_3_As were also tested, and again showed no catalytic activity, while KPPh_2_ gave low performance, with 15% of compound **7** generated after 1 h. These controls are of particular interest because they demonstrate that the [Pn_7_] cluster platform can access chemistry that classical FLPs and simple monopnictogen molecules cannot. The bridging atoms of the [Pn_7_]^3–^ cluster can be considered pseudochalcogen-like^19^ and thus P_4_S_3_ was tested and, although low, showed some catalytic turnover, as did the (Me_3_Si)_3_P_7_ cluster.

The turnover frequency (TOF) of [K­(18c6)]_3_[**6**] (TO*F*_max_ = 160 h^–1^) can be compared to other homogeneous catalysts reported in related N_2_O reductions (see Supporting Information 2.7.). These comparisons reveal that the performance of [K­(18c6)]_3_[**6**] is modest compared to Cornella’s Bi system (TO*F*_max_ = 3120 h^–1^); however, it is noteworthy that the time of the Bi reactions was determined using a visual color change and not a spectroscopic method.^12^ While [K­(18c6)]_3_[**6**] outperforms Mo’s stannylone system (TO*F*_max_ = 55 h^–1^) and Cantat’s method (TO*F*_max_ = 10 h^–1^).^11,13^ Compared to transition metal catalysts reported in the hydroborative reduction of N_2_O (TO*F*_max_ between 36 and 100 h^–1^),^5,6^ [K­(18c6)]_3_[**6**] remains competitive under similar or milder conditions. Meanwhile, hydrogenation of N_2_O mediated by homogeneous transition metal catalysts is often reported to be relatively slow (TO*F*_max_ = 9–16 h^–1^),^4^ especially considering the higher pressures and/or temperatures required. Very recently, Trincado and Grützmacher reported a rhodium olefin-amine carbene complex that shows very high catalytic activity (TO*F*_max_ > 1300 h^–1^) in the hydrogenation of N_2_O under moderate conditions (*pH*_2_ 1.5–3 atm, *pN*_2_ *O* 1.5–3 atm, 65–80 °C, 10 eq. ^*t*^BuOK).^7k^

### Mechanistic Investigations

To probe the mechanism of N_2_O reduction, HBpin was investigated with [**5**]^3–^ and [**6**]^3–^ in a stoichiometric fashion, and NMR spectroscopy revealed decomposition after 1 h (Figure 3a; Supporting Information 3). These reaction mixtures were subsequently pressurized with N_2_O, and no production of **7** and/or **8** was detected. However, when stoichiometric reaction mixtures of [**5**]^3–^ and [**6**]^3–^ with HBpin were flash-frozen before being allowed to react and then pressurized with N_2_O, compound **8** could be observed by NMR spectroscopy. Addition of stoichiometric amounts of HBpin to these reaction mixtures revealed complete consumption of **8** and the formation of **7**, consistent with dehydrocoupling between **8** and HBpin leading to **7**. During the catalytic studies, reaction mixtures of [Pn_7_]^3–^ and HBpin were immediately frozen prior to N_2_O addition, which led to high conversions of **7** and/or **8** being observed. However, if the [Pn_7_]^3–^ clusters were first allowed to react with HBpin and then the reaction mixture was pressurized with N_2_O, only minor amounts of **7** were detected. These observations are consistent with the first catalytic step being the reaction of [Pn_7_]^3–^ with N_2_O. When a DMF solution of [**5**]^3–^ was allowed to react with 1 atm of N_2_O, gas evolution and an instant color change were observed. ^31^P NMR spectroscopy revealed three new resonances with an integration of 3:3:1 that are correlated, indicative of a tris-functionalized cluster with C_3v_ symmetry. These resonances match literature reports for the [P_7_O_3_]^3–^ cluster (Figure 3a **I1’’’**) where the bridging phosphorus atoms have been oxidized.^25^ Although single crystals suitable for X-ray diffraction (XRD) studies could not be obtained for **I1’’’**, structurally related N-substituted clusters were fully characterized, including with XRD studies by us in 2024, where computational investigations revealed that installation of the more electronegative N resulted in oxidation of the bridging P atoms,^26^ as would be the case with the oxygen-bound P atoms in compounds **I1’**–**I1’’’**. Efforts to obtain analytically pure **I1’’’** salts were unsuccessful and prevented independent catalytic testing. However, cold stepwise addition of HBpin to in situ-generated **I1’’’** allowed for the observation of regenerated [**5**]^3–^, along with [P_5_]^−^ and [P_16_]^2^^–^ by NMR spectroscopy and high-resolution mass spectrometry (HRMS) (Figure 3a). The in situ*-*generated solution of these polyphosphides was repressurized with N_2_O, and the formation of **I1’’’** could again be observed by NMR spectroscopy and HRMS techniques. These experiments demonstrate that a [**5**]^3–^ ⇌ **I1** catalytic cycle is possible, which can be understood as a P(−1)/P­(+1) redox couple at the bridging P atoms. Low-valent redox couples have been reported with the heavy pnictogen Bi,^27^ but not with its lighter congeners P and As.

**Figure 3:**
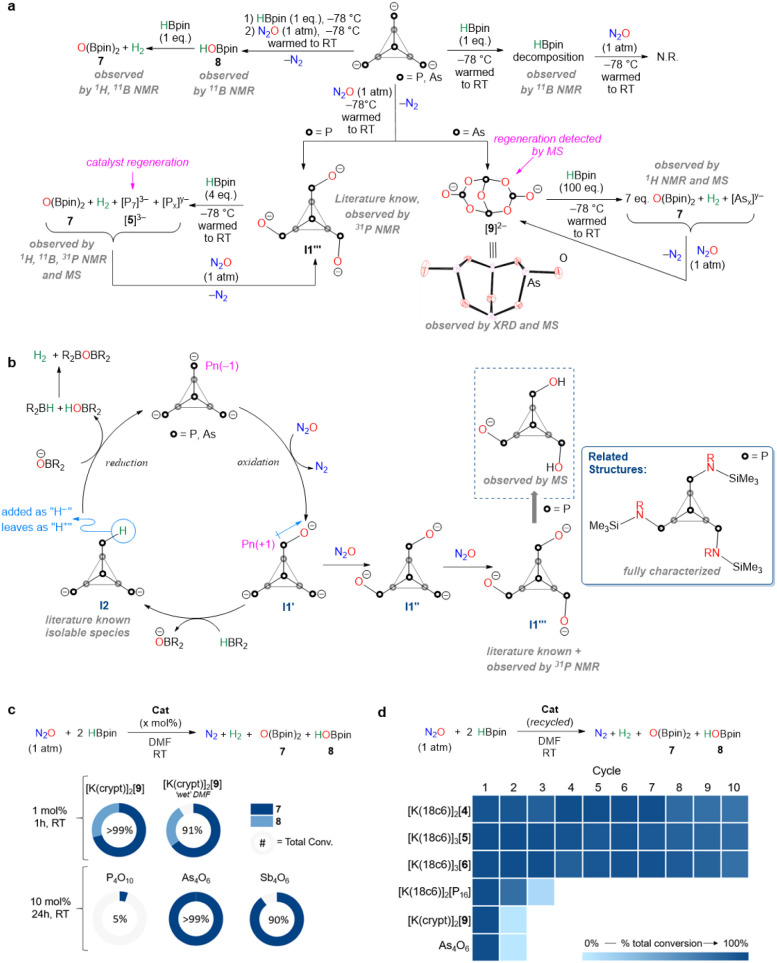
(a) Stepwise experimental studies into N_2_O reduction mechanism. (b) Postulated mechanism; see Supporting Information 10.2 for full details. (c) Hydroboration of N_2_O using Pn oxides. NMR conv. was determined by ^1^H NMR spectroscopy using C_6_H_6_ as an internal standard. Tabulated data are provided in Supporting Information 4.1. (d) Catalyst recycling studies for N_2_O hydroboration (catalyst loading = 1 mol %, except for As_4_O_6_ which was performed at 10 mol %). NMR conv. was determined by ^1^H NMR spectroscopy using C_6_H_6_ as an internal standard. Tabulated data are provided in Supporting Information 5.

To probe the catalytic cycle shown in Figure 3b further, we turned to density functional theory. Full details of the computational methodology, computational tables and figures, as well as the Cartesian coordinates and energies of all stationary points, are provided in the Supporting Information 10.2 and the source data file. In the following discussion, we focus on the reactions of [**5**]^3–^, and Figure S157 shows the computed mechanism for the cycle in Figure 3b. Cluster [**5**]^3–^ reacts with N_2_O to form **TS1**, which then decomposes to form **I1’** and release N_2_. An adduct is then formed between **I1’** and HBpin. This rearranges via **TS2** to form **I2** and eliminates ^–^OBpin. The protonated clusters [HPn_7_]^2^^–^ (**I2**) are known in the literature and can be deprotonated.^28^ The ^–^OBpin then abstracts the H atom from **I2** via **TS3** to form HOBpin (**8**) and regenerates [**5**]^3–^. Energetic Span Model (ESM) analyses for both [**5**]^3–^ and [**6**]^3–^ indicate that the relative turnover frequency (TOF) of the latter is 68× that of the P analogue, in qualitative agreement with experimental results. As described in Supporting Information section 10.2, the ESM provides insights into the key intermediates and transition states that collectively determine the kinetics and TOF of the reaction. Table S12 indicates that, for both [**5**]^3–^ and [**6**]^3–^, these key states are **I2** and **TS1** (Figure S157), respectively; these are the TOF-determining intermediate and the TOF-determining transition states. The energy difference between these two states—the largest in the catalytic cycle, i.e., the energetic span—determines the TOF. This energy difference is 3.8 kcal/mol smaller for [**6**]^3–^ than [**5**]^3–^, and hence the As system exhibits the larger TOF.

We also studied the off-cycle reaction of **I1’** with further N_2_O; the mechanism for this for [**5**]^3–^ is shown in Figure S158. **I1’** reacts with N_2_O via **TS4** to form **I1’’** and release N_2_. A similar process sees **I1’’** react with N_2_O via **TS5** to generate **I1’’’**. Comparison of the barrier heights for the reaction of **I1’** with N_2_O versus that for the H transfer in the **I1’-HBPin** adduct suggests that the latter is the more likely route.

Interestingly, when a DMF solution of [**6**]^3–^ was allowed to react with 1 atm of N_2_O a color change was observed, transitioning from dark red to colorless. While no single crystals suitable for XRD studies could be obtained when 18c6 was employed, switching to [2.2.2]­cryptand (crypt) allowed for the isolation of single crystals from the reaction mixture. XRD analysis of these crystals revealed the formation of [K­(crypt)]_2_[As_4_O_7_] ([K­(crypt)]_2_[**9**]) (anion shown in Figure 3a; 53% isolated yield). Anion [**9**]^2–^ can be described as an oxidized arsenic trioxide molecule (As_4_O_6_ that has an adamantane cage-like structure),^29^ where one of the bridging As–O bonds of As_4_O_6_ is cleaved and an oxygen atom is added. The isolation of [K­(crypt)]_2_[**9**] raises questions as to whether [**9**]^2–^ is an off-cycle product or if it has any catalytic competency. To interrogate this, 1 mol % [K­(crypt)]_2_[**9**] was tested in the hydroboration of N_2_O and revealed similar performance to [K­(18c6)]­[**6**]. Further, addition of 100 equiv of HBpin to [K­(crypt)]_2_[**9**] resulted in the formation of **7** in 14% yield, as observed by NMR spectroscopy. This is consistent with 14 HBpin molecules removing 7 oxygen atoms from [**9**]^2–^. HRMS studies of this reaction mixture showed that instead of [As_7_]^3–^ being regenerated, polyarsides [As_5_]^−^, [As_16_]^2–^ and [As_21_]^3–^ were formed. It is expected that these polyarsides could have similar reactivity to [As_7_]^3–^, and this was confirmed by reacting the in situ-generated solution of these polyarsides with N_2_O, which led to the detection of [**9**]^2–^ by HRMS.

Although [K­(crypt)]_2_[**9**] is found to have catalytic competency, this does not necessarily mean it is an active intermediate in the primary catalytic cycle. Structurally, it would be expected to form after several oxygen transfer reactions with N_2_O and, from our computational investigations, the reaction with HBpin upon installation of oxygen has a lower barrier than the reaction with more N_2_O to install more oxygens. Anion [**9**]^2–^ is likely generated near the end of the catalytic cycle when HBpin has been consumed and there is excess N_2_O. Nonetheless, the catalytic competency of [K­(crypt)]_2_[**9**] and its structural relationship to As_4_O_6_ prompted the survey of commercially available pnictogen oxides in this transformation (Figure 3c). P_4_O_10_, As_4_O_6_, and Sb_4_O_6_ were investigated, and although P_4_O_10_ showed minimal reactivity, As_4_O_6_ and Sb_4_O_6_ both gave high conversions after 24 h, albeit at higher catalyst loadings. Furthermore, similar to the air-stable P_4_O_10_, As_4_O_6_, and Sb_4_O_6_ oxides, [K­(crypt)]_2_[**9**] was also found to be air-stable. This stability prompted us to study the reduction of N_2_O without predried solvents using standard bench techniques, and with 1 mol % [K­(crypt)]_2_[**9**], high conversions to **7** + **8** (91% total) were obtained.

The observation of various [Pn_*x*_]^y–^ and [Pn_*x*_O_*y*_]^z–^ structures during our mechanistic studies demonstrates the complexity of the mechanism. Many of these [Pn_*x*_]^y–^ and [Pn_*x*_O_*y*_]^z–^ type structures are expected to be present during catalysis and operate concurrently. To demonstrate that larger polypnictogens can also be catalytically active, [P_16_]^2–^ was independently tested at 1 mol % loading under the optimized reaction conditions and showed intermediate performance compared to [**5**]^3–^ and [**6**]^3–^. Although many of the polypnictogens generated may have some catalytic competency, we would not expect them to have the exact same efficiency. It would be expected that as structures with lower performance accumulate, overall conversions should decrease. Specifically, we expect to observe depleting catalyst recyclability as the reaction mixture is reloaded with more substrates. Recyclability studies were focused on [K­(18c6)]_2_[**4**], [K­(18c6)]_3_[**5**], and [K­(18c6)]_3_[**6**] as these were the most active systems. Using 1 mol % initial loading, good recyclability was observed up to 7 cycles, after which performance slowly decreased (Figure 3d). This depletion was modest and, compared to many other homogeneous catalysts reported, which often do not report on any recyclability, an added advantage. Clusters [K­(crypt)]_2_[**9**] (1 mol %), [P_16_]^2–^ (1 mol %), and As_4_O_6_ (10 mol %) were also studied in similar recycling experiments, but significant loss in performance was observed after just 1 cycle. The recyclability of [K­(18c6)]_2_[**4**], [K­(18c6)]_3_[**5**], and [K­(18c6)]_3_[**6**] was further probed by applying recovered catalyst after one cycle in a separate batch reaction (see Supporting Information section 5.2). These experiments reveal that [K­(18c6)]_2_[**4**], [K­(18c6)]_3_[**5**], and [K­(18c6)]_3_[**6**] can be successfully recovered and recycled with only a slight decrease (5–10%) in the TOF compared to the first cycle. Further, previously, we have reported the ability of [Pn_7_]-based clusters to mediate the hydroborative reduction of CO_2_.^21,23^ Thus, selective reduction between CO_2_ and N_2_O (∼1:1 atm) with the high-performing easily synthesized clusters [K­(18c6)]_3_[**5**] and [K­(18c6)]_3_[**6**] was tested at 1 mol % loading with HBpin (Supporting Information 6). Both catalysts were found to selectively reduce N_2_O, and no CO_2_ hydroborated products were observed by ^13^C­{^1^H} and ^1^H NMR spectroscopy.

### Oxygen Transfer to Sulfur

Often, homogeneous catalysts that mediate N_2_O reduction rely on boranes or silanes to capture the oxygen, and it would be an exciting development to transfer that oxygen to build more sought-after element–oxygen bonds. Environmental scientists use sulfur-based reagents to remove the oxygen from N_*x*_O_*y*_ pollutants found in wastewater and automotive exhaust,^30^ and oxidized sulfur moieties are important scaffolds in pharmaceuticals and agrochemicals.^31^ These reports motivated us to study the transfer of oxygen atoms originating from N_2_O to sulfur. To this end, isolated [K­(crypt)]_2_[**9**] was reacted with S_8_ in DMF, resulting in an immediate color change. HRMS studies of the reaction mixture revealed the presence of several arsenic sulfide species and oxidized S_8_ products [S_7_O_6_]^2–^ and [S_8_O_6_]^2–^ (Figure 4). Single crystals were obtained from the reaction, and XRD studies confirmed the formation of S–O bonds, along with stretches corresponding to these bonds, observed by infrared (IR) spectroscopy. In a similar fashion, [K­(crypt)]_2_[**9**] was reacted with 1 atm of SO_2_, which resulted in an immediate color change, and the single crystals obtained from this reaction mixture confirmed the formation of [S_2_O_6_]^2–^. However, determining conversion from these reaction mixtures is difficult due to the lack of suitable NMR handles. Thus, 4-nitrophenyl disulfide [(NO_2_PhS)_2_] was reacted with [K­(crypt)]_2_[**9**] in a 1:1 ratio, and the reaction mixture was monitored by ^1^H and ^13^C­{^1^H} NMR spectroscopy. Near-complete consumption of (NO_2_PhS)_2_ and the formation of two new products consistent with the NMR resonances for [NO_2_PhSO_4_]^−^ and [NO_2_PhSO_3_]^−^ (86% and 12% conversions, respectively) were observed. Further, N_2_O was found to not independently react with these sulfur-based reagents. In these experiments, the oxygen that originated from N_2_O was transferred to arsenic, affording [**9**]^2–^ and then to sulfur. These reactions are important first steps in building catalytic transformations that shuttle oxygen from N_2_O to build S–O bonds, but the catalytic loop here has not yet been closed.

**Figure 4:**
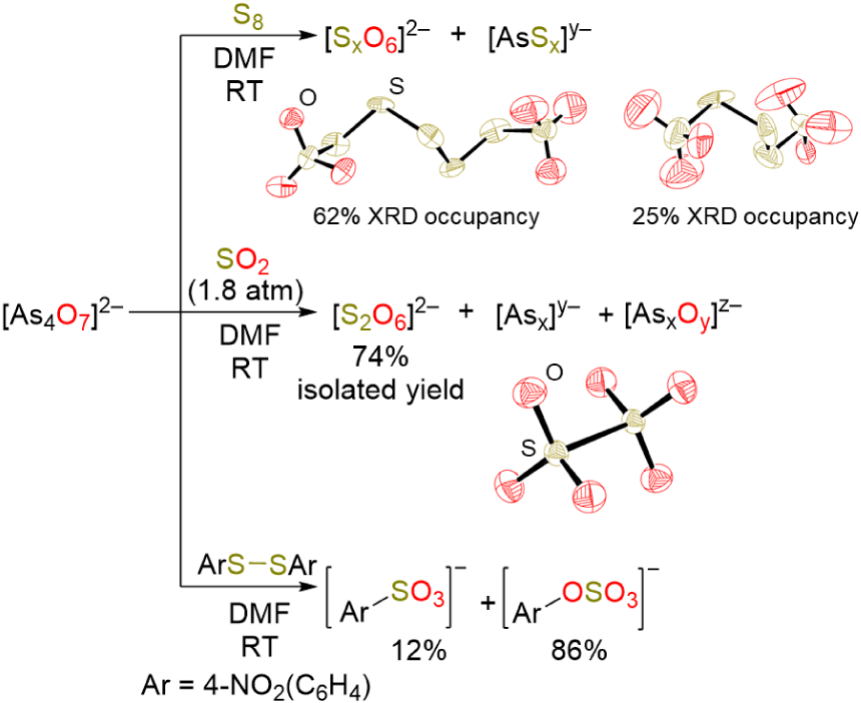
Oxidation of sulfur-containing compounds using [K­(crypt)]_2_[**9**].

### Reduction of Nitro- Functional Groups

Beyond N_2_O, the reduction of other substrates featuring N–O multiple bonds was surveyed. Specifically, using 1 mol % [K­(18c6)]_3_[**6**] in *o*DFB the reduction of nitro-functionalized compounds (**10**) to amines (**11**) was tested (see Figure 5). Initially, the reduction of nitrobenzene was targeted, and complete hydroboration was observed to afford the corresponding aminoborane **11a**. Upon aqueous workup, the aniline hydrochloride salt **12a** was isolated in 95% yield. The scope was expanded to substituted nitrobenzenes, and high conversion to the aminoboranes **11b**–**11g** was observed (88–99%) with the hydrochloride salts **12b**–**12g** isolated in high yields (74–92%). However, when 1-nitroanthracene (**10h**) was tested, the reaction proceeded more slowly and required heating at 100 °C overnight to reach 58% conversion to **11h**. Further, substitution of the phenyl group of nitrobenzene for pyridine resulted in a decreased conversion of 76% to **11i** with **12i** isolated in 68% yield, while the alkyl nitro compound 2-nitropropane (**10j**) could be reduced with good conversion to **11j** (83%) and **12j** isolated in **74**% yield. The aminoborane and ammonium salts isolated in this chemistry contrast with related work done by Mo et al. and Zhou et al., where nitrobenzenes are reduced to hydrazines, azoarenes, or azoxyarenes, instead of amines.^13,32^

**Figure 5:**
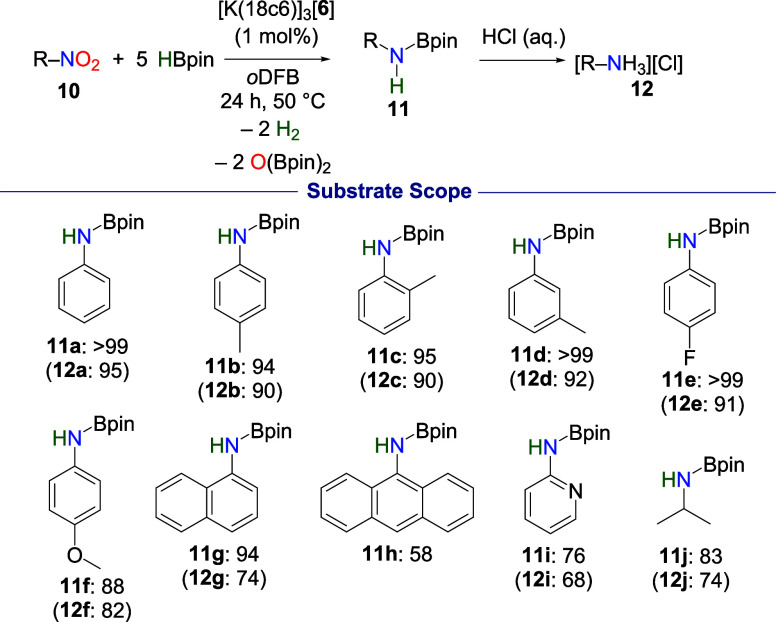
Reduction of nitrocompounds using [K­(18c6)]_3_[**6**]. Reaction conditions: 1 μmol [K­(18c6)]_3_[**6**], 0.1 mmol nitro-functionalized compound, 0.5 mmol HBpin, 0.5 mL of *o*DFB. NMR conversion was determined by integration of the crude ^1^H NMR spectrum using the toluene (0.24 mmol) as the internal standard (^1^H δ = 2.31 ppm). Isolated yields (in parentheses) of the hydrochloride salts were obtained after aqueous workup.

## Conclusion

We have studied a range of (un)­functionalized [Pn_7_] Zintl clusters, along with [Pn_*x*_O_*y*_]^z–^ clusters, in the catalytic reduction of the greenhouse gas N_2_O to environmentally benign N_2_. In general, arsenic based clusters were found to have the best performance and outperform many other homogeneous catalysts reported in related transformations. We find that the different cluster types studied offer different benefits, for example, the [Pn_7_]^3–^ (Pn = P, As) catalysts can be recycled and selectively reduce N_2_O in the presence of CO_2_, while [As_4_O_6_] is commercially available and [As_4_O_7_]^2–^ is air stable. Further, control reactions reveal that these clusters access chemistry that simple monopnictogen molecules do not. Mechanistic studies of the [P_7_]^3–^ cluster indicate the possibility of an unprecedented low-valent Pn(−1)/Pn­(+1) redox couple, and initial efforts have been made to build useful sulfur–oxygen bonds using the oxygen from N_2_O, with future work focused on closing this catalytic loop. Beyond N_2_O, the reduction of nitro-functionalized substrates to amines was successfully achieved. This work represents a significant advancement in catalysis and cluster chemistry, with the mediation of difficult and underdeveloped N–O bond reductions demonstrating good performance and providing better understanding of how main group clusters offer access to chemistries beyond the purview of their noncluster counterparts.

## Supplementary Material





## Notes

Supplemental experimental procedures, synthesis and characterization data, catalytic reactions, reactivity studies, and computational details (PDF)
Coordinates of optimized structures (XLSX)

## ABBREVIATIONS

FLPs: frustrated Lewis pair
18c6: 18-crown-6; HBpin, pinacole borane
DMF: dimethylformamide
NMR: nuclear magnetic resonance
TOF: turnover frequency
crypt: [2.2.2]­cryptand
ESM: Energetic Span Model
