# Supplementary material for: Catalytic Nitrous Oxide Degradation with Group 15 Clusters
Source: J Am Chem Soc. 2025 Aug 6;147(33):30317–25. doi: 10.1021/jacs.5c09618 (PMC12371874; doi:10.1021/jacs.5c09618)
Supplement: Supplementary file 1 [file ja5c09618_si_001.pdf]

Catalytic Nitrous Oxide Degradation with Group 15 Clusters

Bono van IJzendoorn,[1] Reece Lister-Roberts,[1,2] Nikolas Kaltsoyannis,[2*] Meera Mehta[1*]

1.Department of Chemistry, University of Oxford, 12 Mansfield Road, Oxford, OX1 3QR, U.K. meera.mehta@chem.ox.ac.uk

2. Department of Chemistry, University of Manchester, Oxford Road, Manchester, M13 9PL, U.K. nikolas.kaltsoyannis@manchester.ac.uk

Table of Contents

1. Methods and Materials 4
1.1. Experimental Considerations 4
1.2. Analytical Considerations 5
1.3. X-ray Diffraction Studies 6
1.4. Synthesis of $[Pn_X]^{y-}$ salts 7
1.4.1. Synthesis of $[K(18c6)]_3[P_7]$, $[K(18c6)_3[5]$ 7
1.4.2. Synthesis of $[K(18c6)]_3[As_7]$, $[K(18c6)_3[6]$ 9
1.4.3. Synthesis of $[K(18c6)]_2[P_{16}]$ 11
1.5. Synthesis Literature Compounds 13
1.5.1. Synthesis of $[Na(DME)_x]_3[P_7]$ 13
1.5.2. Synthesis of K_3P_7 14
1.5.3. Synthesis of $[K(DME)_x]_3[As_7]$ 14
1.5.4. Synthesis of $[Na(18c6)]_2[HP_7]$ 15
1.5.5. Synthesis of $[K(18c6)]_2[HAs_7]$ 15
1.5.6. Synthesis of $[Na(18c6)]_2[(BBN)P_7]$, $[Na(18c6)]_2[1]$ 16
1.5.7. Synthesis of $[Na(18c6)]_2[(i Bu_2Al)P_7]$, $[Na(18c6)]_2[2]$ 16
1.5.8. Synthesis of $[Na(18c6)]_2[(Ph_2In)P_7]$, $[Na(18c6)]_2[3]$ 17
1.5.9. Synthesis of $[K(18c6)]_2[(i Bu_2Al)As_7]$, $[K(18c6)]_2[4]$ 18
1.5.10. Synthesis of $(Me_3Si)_3P_7$ 19
2. Optimization and control reactions for the reduction of N_2O, and Labelled Experiments 20
2.1. Reductant optimization 20
2.2. Solvent optimization 22
2.3. ^{15}N-labelled N_2O experiment 24
2.4. DBpin labelled reactions 27
2.5. Catalyst optimization 28
2.6. Control reactions 30
2.7. Comparison to Literature Catalysts for N_2O Reduction 31
3. Mechanistic studies 33
3.1. Addition of HBpin to $[K(18c6)]_3[5]$ 33
3.2. Addition of HBpin to $[K(18c6)]_3[6]$ 34
3.3. $[K(18c6)]_3[5]$ and HBpin frozen, and then addition of N_2O 36
3.4. $K(18c6)]_3[6]$ and HBpin frozen, and then addition of N_2O 38

3.5. Addition HBpin to a catalytic amount of [K(18c6)]$_3$[5], followed by N_2O addition 40
3.6. Addition HBpin to catalytic amount [K(18c6)]$_3$[6], followed by N_2O 41
3.7. Addition of N_2O to [K(18c6)]$_3$[5] 42
3.8. Addition of HBpin to *in situ* generated $[P_7O_3]^{3-}$ 45
3.8.1. Addition of excess HBpin 45
3.8.2. Addition of increasing controlled amounts of HBpin to *in situ* generated $[P_7O_3]^{3-}$ and then subsequent N_2O reactivity 46
3.9. Addition of N_2O to K_3As_7 + crypt, synthesis of [K(crypt)]$_2$[9] 47
3.10. Addition of HBpin to [K(crypt)]$_2$[9] 52
3.11. N_2O hydroboration catalyzed by [K(18c6)]$_2$[P_{16}] 55
4. N_2O Reduction with Pnictogen Oxide Clusters 56
4.1. Pnictogen oxides mediated hydroboration of N_2O 56
4.2. N_2O reduction under areobic conditions 57
5. Catalyst recycling and recovery studies 60
5.1. Catalyst recycling in the hydroboration of N_2O 60
5.2. Catalytic hydroboration of N_2O using recovered catalysts 67
6. CO_2 vs N_2O selectivity studies 71
6.1. Reactivity of [K(18c6)]$_3$[5] with N_2O and CO_2 gas mixture 71
6.2. Reactivity of [K(18c6)]$_3$[6] with N_2O and CO_2 gas mixture 72
7. Oxygen transfer to sulfur 74
7.1. Stoichiometric Reaction [K(crypt)]$_2$[As_4O_7] with S_8 74
7.2. Attempted catalytic oxygenation of S_8 by [K(crypt)]$_2$[As_4O_7] 77
7.3. Reaction [K(crypt)]$_2$[As_4O_7] with SO_2 79
7.4. Stoichiometric reaction [K(crypt)]$_2$[As_4O_7] with 4-nitrophenyl disulfide 81
8. Hydroboration of nitro-functionalized compounds 87
8.1. General Procedure for the Hydroboration of Nitro-functionalized compounds 87
8.2. Characterization Data Hydroboration of Nitroarenes 87
9. Crystallography Tables 103
10. Density Functional Theory 106
10.1. Computational Methods for Infrared Studies 106
10.2. Computational Methods Mechanistic Studies 106
11. References 111

1. Methods and Materials

1.1. Experimental Considerations

All manipulations were performed under an inert atmosphere using standard Schlenk-line, and glovebox (MBraun Unilab) techniques, except for aqueous work-up described in section 8. Glassware was flame dried prior to use.

Dry dimethoxy ethylene (DME), benzene (C_6H_6), tetrahydrofuran (THF), diethyl ether, toluene, dimethylformamide (DMF), hexane and pentane were obtained using Innovative Technologies anhydrous engineering solvent purification systems and subsequently degassed. Pyridine was dried over activated 3 Å molecular sieves, purified by distillation, and subsequently degassed. *Ortho*-difluorobenzene (*o*DFB) was dried over CaH_2, purified by distillation, and subsequently degassed. DMF-d_7, THF-d_8, C_6D_6, were dried over activated 3 Å molecular sieves. All solvents were stored over activated 3 Å molecular sieves. Water-d_2 and methanol-d_4 were used as received (Sigma-Aldrich).

Red phosphorus, arsenic powder, naphthalene, triethylsilane (Et_3SiH), triphenylsilane (Ph_3SiH), bis(pinacolato)diboron (B_2Pin_2), catechol borane (HBcat), pinacol borane (HBpin), 9-borabicyclo[3.3.1]nonane dimer (HBBN dimer), diisobutylaluminium hydride (DiBAl-H), 1,4,7,10,13,16-hexaoxacyclooctadecane (18-crown-6, 18c6), 4,7,13,16,21,24-hexaoxa-1,10-diazabicyclo[8.8.8]hexacosane ([2.2.2]cryptand; crypt), elemental sulfur, bis(4-nitrophenyl) disulfide ($(4\text{-}NO_2PhS)_2$), nitrobenzene, 4-nitrotoluene, 2-nitrotoluene, 3-nitrotoluene, 4-fluoro nitrobenzene, 4-methoxy nitrobenzene, 1-nitronapthalene, 1-nitroantracene, 2-nitropyridine, and 2-nitropropane were purchased from commercial sources (Apollo Scientific, Sigma-Aldrich, Alfa Aesar, Fluorochem, Tokyo Chemical Industry, Thermo Fisher Scientific, and Acros Organics) and used without purification. Elemental sodium and elemental potassium were cleaned by removal of the oxide layers and washing with toluene and hexane. Carbon dioxide and N_2O was purchased from CK gas products limited. $[Na(DME)_x]_3P_7$,[1] K_3P_7,[1] $[K(DME)_x]_3As_7$,[2] $(Me_3Si)_3P_7$,[1] $[Na(18c6)]_2[HP_7]$,[3] $[K(18c6)]_2[HP_7]$,[3] $[K(18c6)]_2[HAs_7]$,[3] $[Na(18c6)]_2[(BBN)P_7]$,[4] $[K(18c6)]_2[(BBN)P_7]$,[4] $[Na(18c6)]_2[(Ph_2In)P_7]$,[5] $[K(18c6)]_2[(^iBu_2Al)As_7]$,[6] $[Na(18c6)]_2[(^iBu_2Al)P_7]$[6] and DBpin[7] were synthesized using literature procedures.

1.2. Analytical Considerations

NMR Spectroscopy. ^{1}H, ^{2}H, ^{11}B, $^{11}B\{^{1}H\}$, $^{13}C\{^{1}H\}$, ^{15}N, $^{19}F\{^{1}H\}$, $^{29}Si\{^{1}H\}$, ^{31}P, ^{31}P COSY, and ^{1}H ^{13}C HSQC NMR spectra were recorded on a Bruker AVIII 400 spectrometer (operating frequencies: 400.17 MHz, 61.41 MHz, 128.36 MHz, 100.53 MHz, 376.53 MHz, 79.48 MHz and 161.99 MHz for ^{1}H, ^{2}H, ^{11}B, ^{13}C, ^{19}F, ^{29}Si and ^{31}P, respectively) or Bruker AVIII 500 spectrometer (operating frequencies: 499.94 MHz, 160.40 MHz, 125.72 MHz, 50.67 MHz and 202.37 MHz for ^{1}H, ^{11}B, ^{13}C, ^{15}N, and ^{31}P, respectively). ^{1}H and $^{13}C\{^{1}H\}$ NMR chemical shifts were internally referenced to the residual solvent resonances (MeOD-d_4 (methanol-d_4): ^{1}H δ = 4.78, 3.31 ppm, $^{13}C\{^{1}H\}$ δ = 49.15 ppm, C_6D_6 (benzene-d_6): ^{1}H δ = 7.16 ppm, $^{13}C\{^{1}H\}$ δ = 128.02 ppm, THF-d_8 (tetrahydrofuran-d_8): ^{1}H δ = 3.58, 1.73 ppm, $^{13}C\{^{1}H\}$ δ = 67.57, 25.37 ppm, DMF-d_7 (N,N-dimethylforamide-d_7): ^{1}H δ = 8.03, 2.92, 2.75 ppm, $^{13}C\{^{1}H\}$ δ = 163.15, 34.89, 29.76 ppm), D_2O (water-d_2): ^{1}H δ = 4.79 ppm. ^{2}H, ^{11}B, ^{15}N, ^{29}Si, ^{31}P NMR chemical shifts were externally referenced to Me_4Si-d_{12}, $BF_3{\cdot}Et_2O$, NH_3, Me_4Si, H_3PO_4, respectively. Solution phase NMR samples were prepared under an inert atmosphere in 5 mm J Young NMR tubes. 1D and COSY NMR data was analyzed using MestReNova V14.0.0 software or Topspin V3.6.1 software.

Elemental Analysis. Elemental analysis was carried out by the microanalysis service of the University of Manchester using a Flash 2000 elemental analyser. Samples were prepared under a nitrogen atmosphere.

Mass spectrometry. Mass spectrometry samples were measured by the mass spectrometry service of the University of Manchester using an electrospray ionization (ESI) or atmospheric pressure chemical ionization (APCI) equipped Thermo Orbitrap Executive Plus Extended Mass Range mass spectrometer or samples were measured by the mass spectrometry service of the University of Oxford using an electrospray ionization (ESI) equipped Waters RDa bench-top time of flight mass spectrometer. Samples were prepared under a nitrogen atmosphere and directly injected into the ionization source of the mass spectrometer.

1.3. X-ray Diffraction Studies

Data collection: X-ray diffraction data for $[K(crypt)]_2$[**9**]·DMF were collected on a dual wavelength Rigaku FR-X rotating anode diffractometer using CuKα (λ = 1.54184 Å) and MoKα (0.71073 Å) radiation, respectively, equipped with an AFC-11 4-circle kappa geometry goniometer, VariMAX^{TM} microfocus optics, a Hypix-6000HE detector and an Oxford Cryosystems Cryostream 800 nitrogen flow gas system, at a temperature of 100 K. Data were collected and reduced using Rigaku CrysAlisPro v42. Absorption correction was performed using empirical methods (SCALE3 ABSPACK) based upon symmetry-equivalent reflections combined with measurements at different azimuthal angles.

X-ray diffraction data for $[K(18c6)]_3$[**5**]·C_5H_5N, $[K(18c6)]_3$[**6**], $[K(crypt)]_2[S_{6.5}O_{5.2}]$·2DMF, and $[K(crypt)]_2[S_2O_6]$ were collected an Oxford Diffraction/Agilent SuperNova diffractometer equipped with a 135 mm Atlas CCD area detector or a Rigaku XtaLAB Synergy-DW VHF equipped with a PhotonJet-R dual wavelength rotating anode and HyPix-Arc 150° detector. Crystals were mounted and quench-cooled using an Oxford Cryosystems open flow N_2 cooling device. Data were collected at 150 K using mirror monochromated CuKα radiation (λ = 1.54184 Å). Data were collected and reduced using Rigaku CrysAlisPro v42. Absorption correction was performed using empirical methods (SCALE3 ABSPACK) based upon symmetry-equivalent reflections combined with measurements at different azimuthal angles.

Crystal structure determination and refinements: The crystal structures were solved and refined against all F^2 values using the SHELX and Olex2 suite of programmes.[8] All non-hydrogen atoms were refined anisotropically. Hydrogen atoms were placed in calculated positions and refined using idealized geometries. Hydrogen isotropic atomic displacement parameters were constrained to ride with the parent atom with an appropriate multiplier for the hybridization. Disordered 18c6 and $[As_7]$ moieties in $[K(18c6)]_3[P_7]$ and $[K(18c6)]_3[As_7]$ were modelled over two positions and atomic displacement parameters were restrained to be similar using SHELX SIMU and SADI commands. The positional disordered $[As_4O_7]$ moiety in $[K(crypt)]_2[As_4O_7]$·DMF was modelled over two positions. In $[K(crypt)]_2[S_{6.4}O_{5.2}]$·2DMF disorder in the anion was modelled as three different parts ($[S_7O_6]^{2-}$, 62%; $[S_5O_7]^{2-}$, 25%; and $[S_7]^{2-}$, 13%) using SHELX (SIMU, SADI, RIGU, DELU, DANG, and DFIX) commands. These parts

significantly overlap with each other making modelling of the disorder challenging requiring a number of DELU, DANG, and DFIX commands

Crystallographic data for [K(18c6)]$_3$[**5**]·C_5H_5N, [K(18c6)]$_3$[**6**], [K(crypt)]$_2$[**9**]·DMF, [K(crypt)]$_2$[$S_{6.4}O_{5.2}$]·2DMF, and [K(crypt)]$_2$[S_2O_6] have been deposited with the CCDC (CCDC 2434057-2434061). These data can be obtained free of charge via https://www.ccdc.cam.ac.uk/structures/ (or from the Cambridge Crystallographic Data Centre, 12 Union Road, Cambridge CB21EZ, UK; Tel: (+44)1223-336-408; or deposit@ccdc.cam.ac.uk).

1.4. Synthesis of [Pn$_X$]$^{y-}$ salts

1.4.1. Synthesis of [K(18c6)]$_3$[P_7], [K(18c6)$_3$[5]

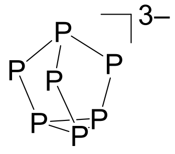

3 [K(18c6)]$^+$

To a Schlenk flask charged with a stir bar and K_3P_7 (500.0 mg, 1.5 mmol, 1 eq.), 18c6 (1186.5 mg, 4.5 mmol, 3 eq.) THF (5 mL) and pyridine (5 mL) was added forming a slurry. The reaction was allowed to react for 4 hours. The mixture was filtered yielding a clear dark red solution. The reaction mixture was filtered and hexane (40 mL) was added to the filtrate forming a yellow precipitate. The supernatant was removed *via* filtration and the residue was dried under reduced pressure which yielded a yellow solid. Crystals suitable for X-ray diffraction analysis were obtained through slow diffusion of hexane into a concentrated THF/pyridine solution. Characterization data is consistent with literature reports.[9]

Isolated Yield: 1231.1 mg, 73%.

^{1}H NMR (400 MHz, 298 K, DMF): δ = 3.61 (s, 18c6) ppm.

^{31}P NMR (162 MHz, 298 K, DMF): δ = –117.20 (br, [P_7]$^{3–}$) ppm.

Mass spectrometry (ESI): [P_7+H_2]$^–$ ([**5**+H_2]$^–$): cald. 218.8320; found: 218.8353.

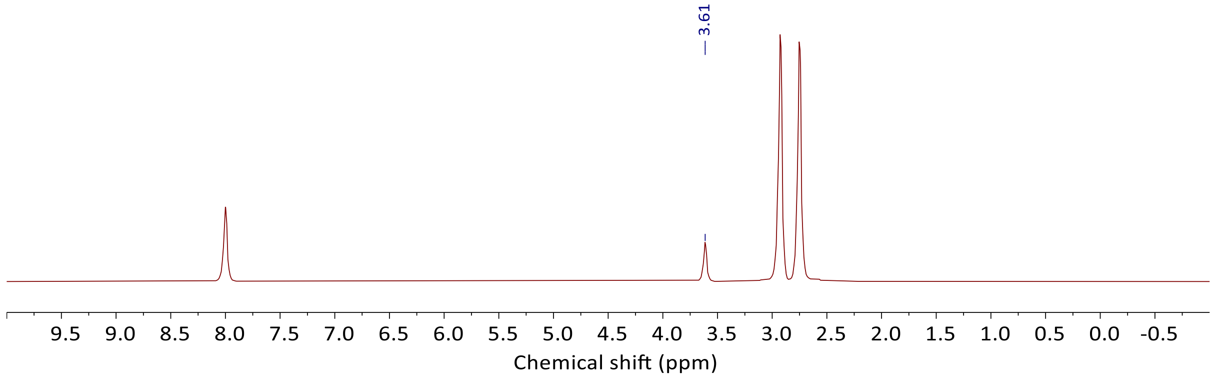

Figure S1. ^{1}H NMR spectrum (400 MHz, DMF) of $[K(18c6)]_3$[**5**].

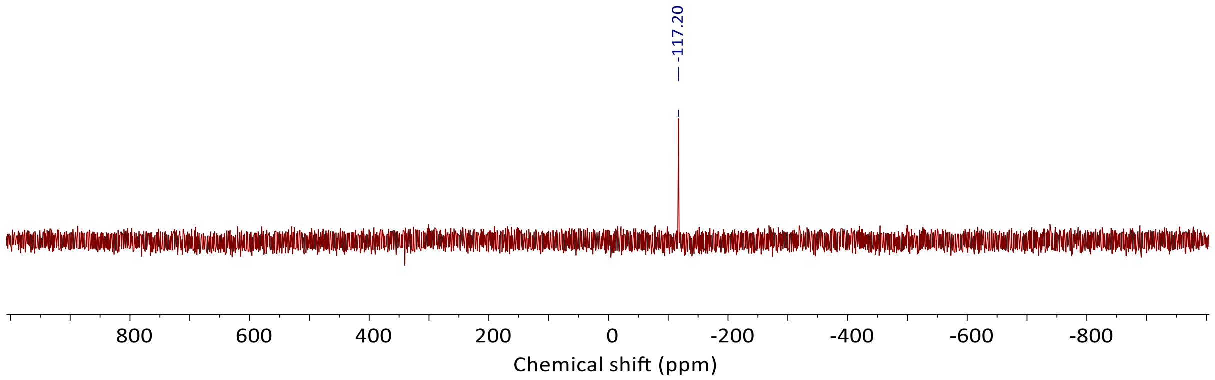

Figure S2. ^{31}P NMR spectrum (162 MHz, DMF) of $[K(18c6)]_3$[**5**].

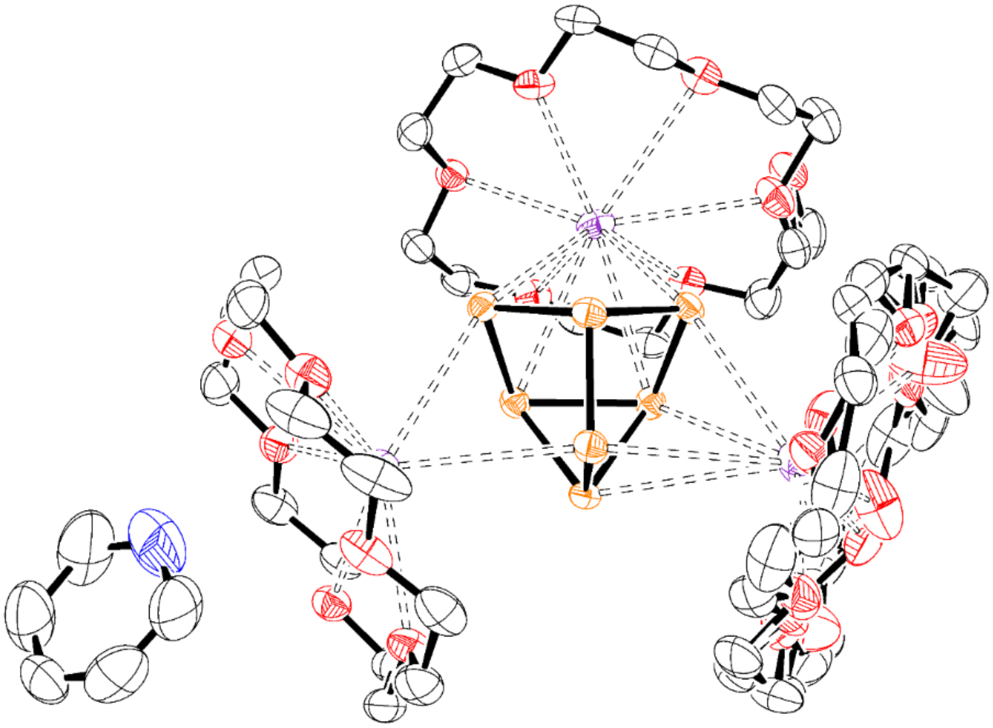

Figure S3. Molecular structure of [K(18c6)]$_3$[**5**]. Anisotropic displacement ellipsoids pictured at 50% probability. Hydrogen atoms omitted for clarity. Phosphorus: Orange; Nitrogen: Blue; Carbon: White; Potassium: Purple; Oxygen: Red. CCDC code: 2434059.

1.4.2. Synthesis of [K(18c6)]$_3$[As$_7$], [K(18c6)$_3$[6]

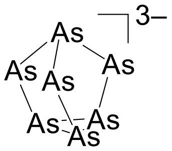

3 [K(18c6)]$^+$

To a Schlenk flask charged with a stir bar and K_3As_7 (576.0 mg, 0.9 mmol, 1 eq.), 18c6 (711.9 mg, 2.7 mmol, 3 eq.) THF (5 mL) and pyridine (5 mL) was added forming a slurry. The reaction was allowed to react for 4 hours. The mixture was filtered yielding a clear dark red solution. The reaction mixture was filtered and hexane (40 mL) was added to the filtrate forming a brown precipitate. The supernatant was removed *via* filtration and the residue was dried under reduced pressure which yielded a brown solid. Crystals suitable for X-ray diffraction analysis were obtained through slow

diffusion of hexane into a concentrated THF/pyridine solution. Characterization data is consistent with literature reports.[9]

Isolated Yield: 463.6 mg, 36%.

^{1}H NMR (400 MHz, 298 K, DMF): δ = 3.61 (s, 18c6) ppm.

Mass spectrometry (ESI): $[As_7+H_2]^-$ ([**6**+H_2]$^-$): cald. 526.4468; found: 526.4461.

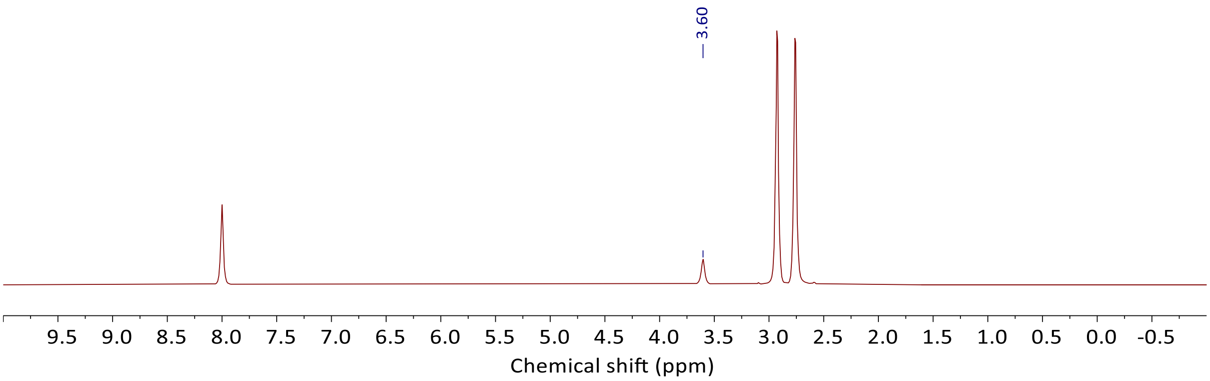

Figure S4 ^{1}H NMR spectrum (400 MHz, DMF) of $[K(18c6)]_3$[**6**].

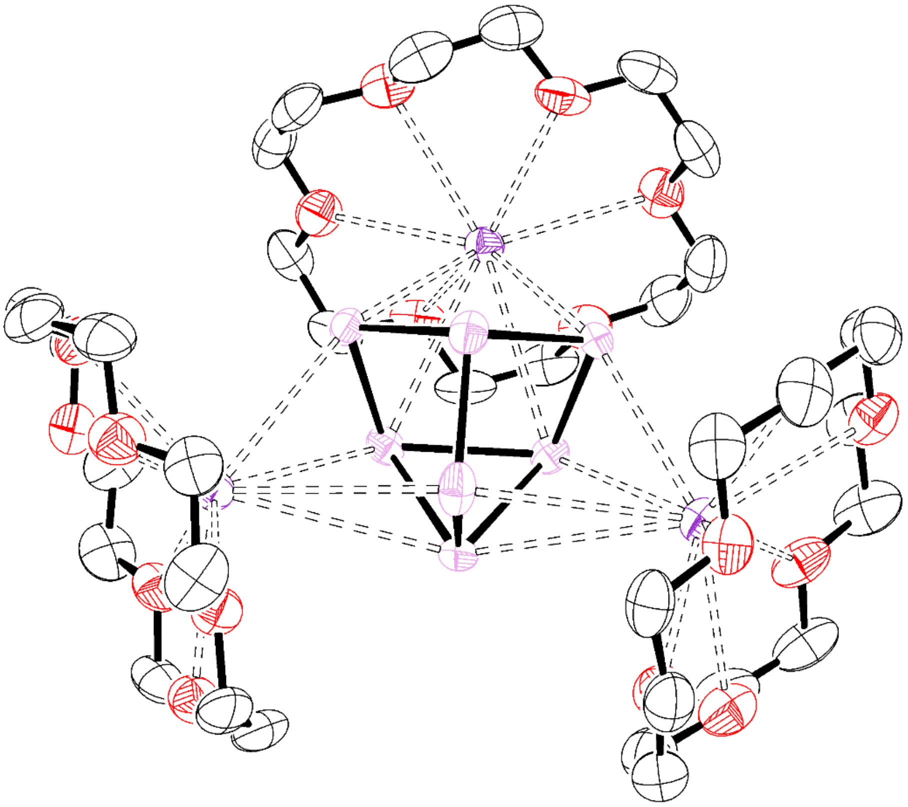

Figure S5. Molecular structure of $[K(18c6)]_3$[**6**]. Anisotropic displacement ellipsoids pictured at 50% probability. Hydrogen atoms omitted for clarity. Positional disorder omitted for clarity and major component (*ca.* 90% occupancy) shown. Arsenic: Plum; Carbon: White; Potassium: Purple; Oxygen: Red. CCDC code: 2434060.

1.4.3. Synthesis of $[K(18c6)]_2[P_{16}]$

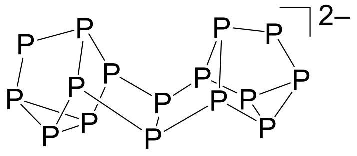

2 $[K(18c6)]^+$

A J Young ampoule with a stir bar was charged with red phosphorus (1390.0 mg, 44.8 mmol, 8 eq.), potassium (220.1 mg, 5.6 mmol, 1 eq.), 18c6 (1479.1 mg, 5.6 mmol, 1 eq.) and naphthalene (71.8 mg, 0.56 mmol, 0.1 eq.). The mixture was cooled to 0 °C and suspended in THF (20 mL). The reaction mixture was stirred overnight to give a dark suspension. The headspace of the flask was removed under vacuum and the

reaction mixture was slowly heated to 90 °C over the course of a day and left to stir 3 days until a red solution with precipitate was observed. The reaction mixture was cooled to room temperature and was filtered yielding a dark red filtrate. The volatiles were removed under reduced pressure and the solids were washed with hexane (3 x 20 mL). The product was then dried under vacuum over the course of a day, yielding a red powder. Spectroscopic data is consistent with literature reports.[10]

Isolated Yield: 1963.2 mg, 64%.

^{1}H NMR (400 MHz, 298 K, THF-d$_8$): δ = 3.61 (s, 18c6) ppm.

^{31}P NMR (162 MHz, 298 K, DMF): δ = 60.14 (d, $^{1}J_{PP}$ = 311.4 Hz, 4P), 38.77 (t, $^{1}J_{PP}$ = 250.3 Hz, 2P), 4.86 (q, $^{1}J_{PP}$ = 345.7 Hz, 2P), –32.89 – –40.75 (m, 2P), –134.52 (dd, $^{1}J_{PP}$ = 360.9, 325.8 Hz, 2P), –173.90 (dd, $^{1}J_{PP}$ = 306.9, 244.8 Hz, 4P) ppm.

Mass spectrometry (ESI): $[P_{16}+H]^{-}$: cald. 496.5875; found: 496.5890.

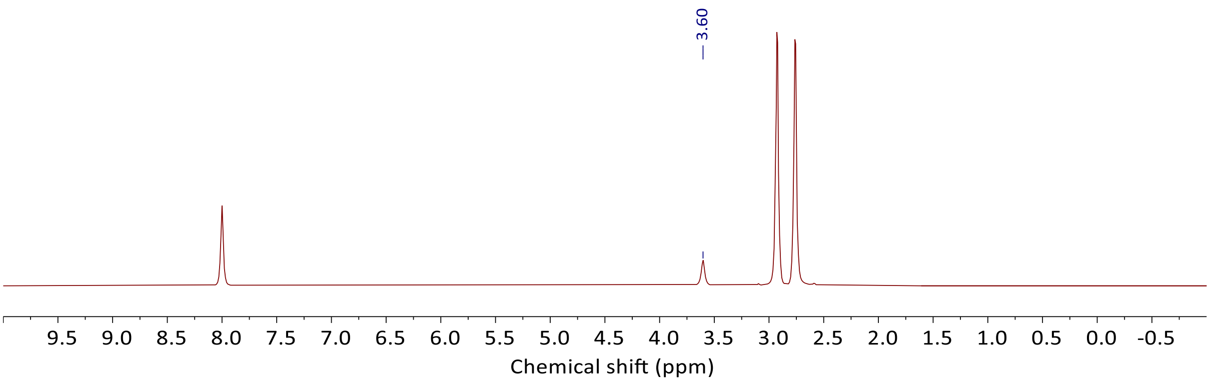

Figure S6 ^{1}H NMR spectrum (500 MHz, DMF) of $[K(18c6)]_2[P_{16}]$.

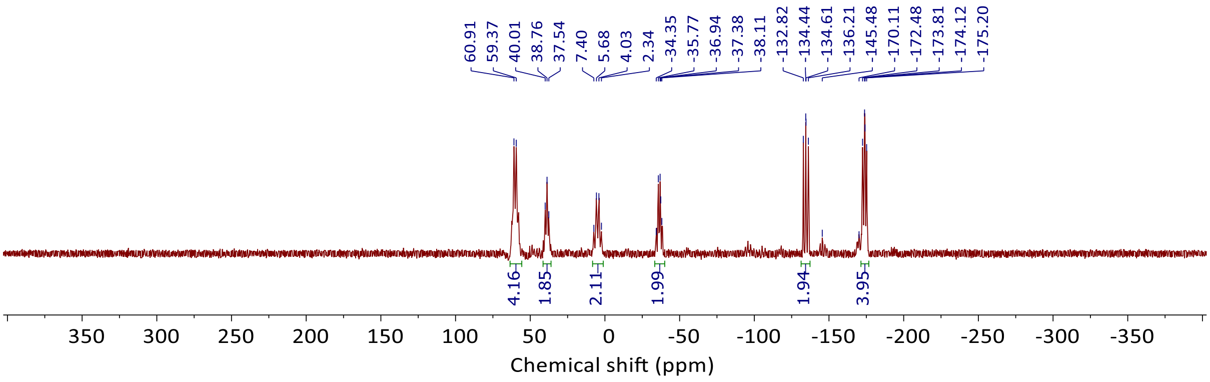

Figure S7. ^{31}P NMR spectrum (202 MHz, DMF) of $[K(18c6)]_2[P_{16}]$.

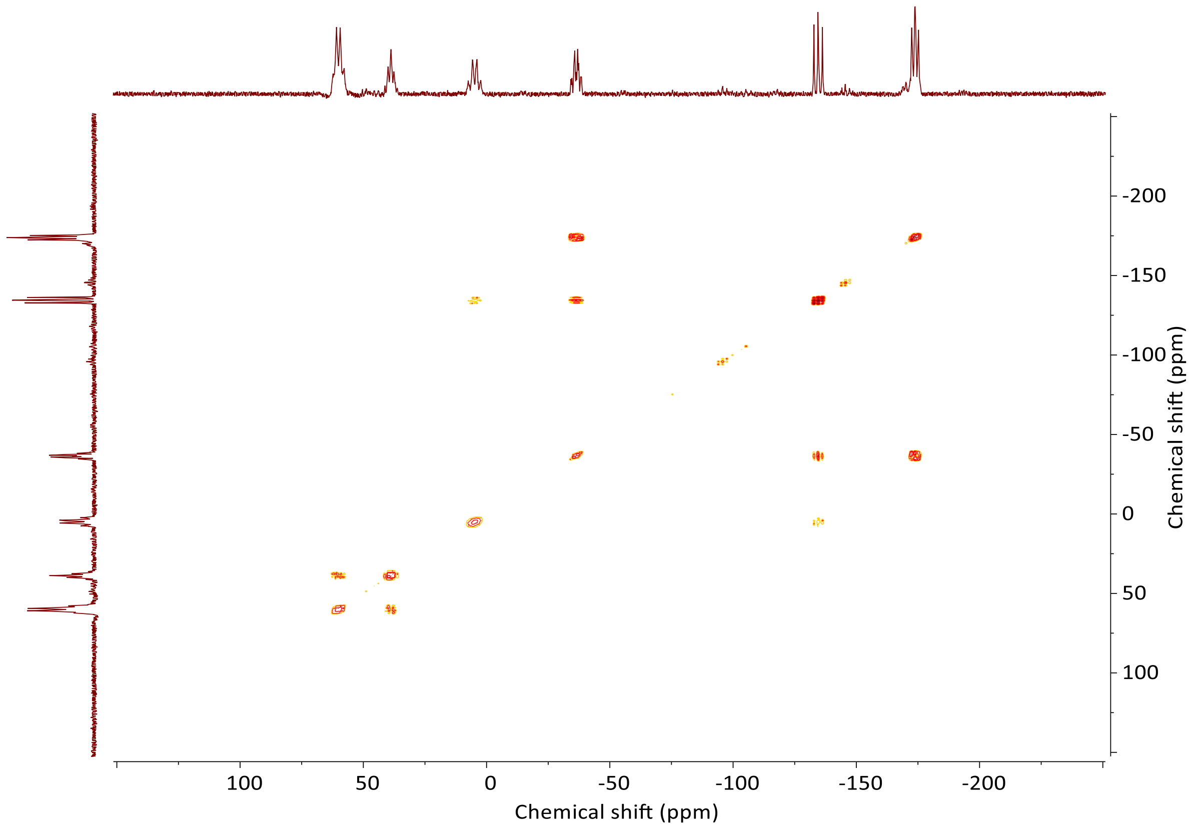

Figure S8. ^{31}P COSY NMR spectrum (202 MHz, DMF) of $[K(18c6)]_2[P_{16}]$.

1.5. Synthesis Literature Compounds

1.5.1. Synthesis of $[Na(DME)_x]_3[P_7]$

Following literature procedure,[1] a Schlenk flask with a stir bar was charged with red phosphorus (25162.1 mg, 0.81 mol, 7 eq.), sodium (8000.3 mg, 0.35 mol, 3 eq.) and naphthalene (2936.2 mg, 23 mmol, 0.2 eq.). The mixture was cooled to 0 °C and suspended in THF (35 mL) and DME (35 mL). The reaction mixture was stirred overnight to give a dark suspension. The headspace of the flask was removed under vacuum and the reaction mixture was slowly heated to 55 °C over the course of a day and left to stir 3 days until a green/yellow suspension was observed. The suspension was cooled to room temperature and was washed with pentane (3 x 40 mL). The product was then dried under vacuum over the course of a day, yielding a green/yellow powder. The $[Na(DME)_x]_3[P_7]$ was used without further purification.

Isolated Yield: 34021.5 mg, 98%.

^{31}P NMR (162 MHz, 298 K, DMF): δ = –113.02 (broad singlet) ppm.

1.5.2. Synthesis of K_3P_7

Following literature procedure,[1] a Schlenk flask with a stir bar was charged with red phosphorus (3700.8 mg, 119 mmol, 7 eq.), potassium (2000.0 mg, 51 mmol, 3 eq.) and naphthalene (218.2 mg, 1.7 mmol, 0.1 eq.). The mixture was cooled to 0 °C and suspended in THF (20 mL) and DME (20 mL). The reaction mixture was stirred overnight to give a dark suspension. The headspace of the flask was removed under vacuum and the reaction mixture was slowly heated to 55 °C over the course of a day and left to stir 3 days until a green/yellow suspension was observed. The suspension was cooled to room temperature and was washed with pentane (3 x 40 mL). The product was then dried under vacuum over the course of a day, yielding a green powder. The K_3P_7 was used without further purification.

Isolated Yield: 5510.8 mg, 96%.

^{31}P NMR (162 MHz, 298 K, DMF): δ = –112.09 (broad singlet) ppm.

1.5.3. Synthesis of $[K(DME)_x]_3[As_7]$

Following literature procedure,[2] a Schlenk flask with a stir bar was charged with grey arsenic (2250.0 mg, 30 mmol, 7 eq.), potassium (500.0 mg, 12.5 mmol, 3 eq.) and naphthalene (55.1 mg, 0.42 mmol, 0.1 eq.). The mixture was cooled to 0 °C and suspended in THF (8 mL) and DME (8 mL). The reaction mixture was stirred overnight to give a dark suspension. The headspace of the flask was removed under vacuum and the reaction mixture was slowly heated to 55 °C over the course of a day and left to stir over 7 days until a red suspension was observed. The suspension was cooled to room temperature and was washed with pentane (3 x 20 mL). The product was then dried under vacuum over the course of 3 days, yielding a red powder. The $[K(DME)_x]_3[As_7]$ was used without further purification.

Isolated Yield: 2531.6 mg, 92%.

1.5.4. Synthesis of [Na(18c6)]$_2$[HP$_7$]

Following literature procedure,[3,4] a Schlenk flask with a stir bar was charged with [Na(DME)$_x$]$_3$[P$_7$] (500.2 mg, ~1.68 mmol, 1 eq.) and the solids were suspended in pyridine (1 mL). While the suspension was stirring at 0 °C, H_2O (60.7 µL, 3.36 mmol, 2 eq.) was added dropwise after which the reaction mixture was allowed for 5 min. To the reaction mixture 18c6 (888.8 mg, 3.36 mmol, 2 eq.) was added under a flow of N_2 and the reaction mixture was allowed to react for 15 min. The mixture was filtered yielding a clear dark red solution. While stirring, the solution was concentrated under reduced pressure to *ca.* 0.5 mL after which toluene (10 mL) was added and an orange solid formed. The product was then dried under vacuum over the course of 7 day, yielding an orange powder.

Isolated Yield: 1060.1 mg, 80%.

^{1}H NMR (400 MHz, 298 K, DMF): δ = 3.57 (s, 48H, 18c6), 0.39 (broad singlet, 1H, *H*P$_7$) ppm.

^{31}P NMR (162 MHz, 298 K, DMF): δ = –24.57 (broad singlet, 1P), –111.01 (broad singlet, 6P) ppm.

1.5.5. Synthesis of [K(18c6)]$_2$[HAs$_7$]

Following literature procedure,[3,4] a Schlenk flask with a stir bar was charged with [K(DME)$_x$]$_3$[As$_7$] (1078.2 mg, ~1.68 mmol, 1 eq.) and the solids were suspended in pyridine (1 mL). While the suspension was stirring at 0 °C, H_2O (60.7 µL, 3.36 mmol, 2 eq.) was added dropwise after which the reaction mixture was allowed for 5 min. To the reaction mixture 18c6 (888.8 mg, 3.36 mmol, 2 eq.) was added under a flow of N_2 and the reaction mixture was allowed to react for 15 min. The mixture was filtered yielding a clear dark red solution. While stirring, the solution was concentrated under reduced pressure to *ca.* 0.5 mL after which toluene (10 mL) was added and a black solid formed. The product was then dried under vacuum over the course of 7 day, yielding a black powder.

Isolated Yield: 569.8 mg, 30%.

1.5.6. Synthesis of $[Na(18c6)]_2[(BBN)P_7]$, $[Na(18c6)]_2[1]$

Following literature procedure,[5] a Schlenk flask with a stir bar was charged with $[Na(18c6)]_2[HP_7]$ (2011.2 mg, 2.54 mmol, 1.0 eq.), HBBN dimer (928.1 mg, 3.80 mmol, 1.5 eq.). THF (40 mL) was added to the mixture and gas evolution was observed. The reaction mixture was allowed to stir until no gas evolution was observed. The mixture was filtered yielding a clear dark orange solution. The volatiles were removed under reduced pressure and the residue was washed with toluene (5 x 20mL). The residue was dissolved in THF (5 mL) and filtered yielding a clear dark orange solution. The solution was filtered and removal of volatiles under reduced pressure yielded glassy orange solids.

Isolated Yield: 1425.6 mg, 61%.

^{1}H NMR (400 MHz, 298 K, THF-d$_8$): δ = 3.41 (s, 48H, 18c6), 2.31 – 1.65 (m, 14H, BBN) ppm.

^{31}P NMR (162 MHz, 298 K, THF-d$_8$): δ = 76.45 (ddd, $^1J_{PP}$ = 345.1, 212.9, $^2J_{PP}$ = 60.5 Hz, 2P, bridging), 45.75 (dtd, $^1J_{PP}$ = 376.1, 234.2, $^2J_{PP}$ = 56.8 Hz, 1P, basal), –31.76 (dd (pseudo triplet), $^1J_{PP}$ = 368.6 Hz, 1P, bridging), –56.52 – –63.33 (m, 1P, apical), –153.15 – –159.99 (m, 2P, basal) ppm.

Elemental analysis for $C_{32}H_{62}BNa_2O_{12}P_7$: calcd.: C 42.12, H 6.85, N 0.0; found C 42.25, H 7.09, N 0.0.

1.5.7. Synthesis of $[Na(18c6)]_2[(iBu_2Al)P_7]$, $[Na(18c6)]_2[2]$

Following literature procedure,[6] to a Schlenk flask charged with a stir bar and $[Na(18c6)]_2[HP_7]$ (250.1 mg, 0.32 mmol, 1 eq.) THF (5 mL) was added and cooled to –30 °C forming a slurry. A solution of diisopropyl aluminium hydride (45.1 mg, 0.32 mmol. 1 eq.) in THF (5 mL) was cooled to –30 °C and dropwise added to the $[Na(18c6)]_2[HP_7]$ slurry. Gas evolution was observed, and the reaction was allowed to react for 5 min at –30 °C, after which it was warmed to RT. The mixture was filtered yielding a clear dark orange solution. The solvent was removed under reduced pressure and the residue was washed with toluene (2 x 20 mL). The residue was dissolved in THF and filtered again yielding a dark orange solution. Removal of

volatiles under reduced pressure yielded glassy orange solids. Crystals suitable for X-ray diffraction analysis were obtained through slow diffusion of hexane into a concentrated THF solution.
Isolated Yield: 187.8 mg, 63%.

^{1}H NMR (400 MHz, 298 K, THF-d$_8$): δ = 3.58 (s, 48H, *18-crown-6*, overlap with THF-d$_8$ residual resonance), 2.29 (th, $^3J_{HH}$ = 13.1, 6.6 Hz, 1H, AlCH$_2$C*H*(CH$_3$)$_2$), 1.48 (th, $^3J_{HH}$ = 13.3, 6.6 Hz, 1H, AlCH$_2$C*H*(CH$_3$)$_2$), 1.01 (d, $^3J_{HH}$ = 6.5 Hz, 6H, AlCH$_2$CH(C*H$_3$*)$_2$), 0.61 (d, $^3J_{HH}$ = 6.5 Hz, 6H, AlCH$_2$CH(C*H$_3$*)$_2$), 0.11 (d, $^3J_{HH}$ = 6.9 Hz, 2H, AlC*H$_2$*CH(CH$_3$)$_2$), –0.89 (d, $^3J_{HH}$ = 7.0 Hz, 2H, AlC*H$_2$*CH(CH$_3$)$_2$) ppm.

^{13}C{^{1}H} NMR (101 MHz, 298 K, THF-d$_8$): δ = 68.09 (s, *18-crown-6*), 26.91 (s, Al^i*Bu$_2$*), 26.86 (s, Al^i*Bu$_2$*), 26.50 (s, Al^i*Bu$_2$*), 26.11 (s, Al^i*Bu$_2$*), 25.84 (s, Al^i*Bu$_2$*), 25.72 (s, Al^i*Bu$_2$*) ppm.

^{31}P NMR (162 MHz, 298 K, THF-d$_8$): δ = –29.15 – –36.99 (m, 2P, *bridging*), –45.26 (dd, $^1J_{PP}$ = 399.6, 342.8 Hz, 1P, *bridging*), –52.58 – –55.58 (m, 1P, *basal*), -55.58 – –64.20 (m, 1P, *bridging*), –171.20 – –182.04 (m, 2P, *basal*) ppm.

Elemental analysis for $C_{32}H_{66}AlNa_2O_{12}P_7$: calcd.: C 41.21, H 7.13, N 0.0; found C 41.27, H 7.12, N 0.0

1.5.8. Synthesis of [Na(18c6)]$_2$[(Ph$_2$In)P$_7$], [Na(18c6)]$_2$[3]

Following literature procedure,[6] a Schlenk flask with a stir bar was charged with [Na(DME)$_x$]$_3$[P$_7$] (89.1 mg, 0.30 mmol, 1 eq.), 18c6 (158.5 mg, 0.60 mmol, 2 eq.) and InPh$_3$ (103.8 mg, 0.3 mmol, 1 eq.). The solids were suspended in pyridine (3 mL) and allowed to react for 2 days. The reaction mixture was filtered yielding a dark red filtrate. To this filtrate toluene was added (10 mL) yielding a brown precipitate. The solids were dried under reduced pressure over the course of 2 days, yielding a brown powder.
Isolated Yield: 92.3 mg, 29%.

^{31}P NMR (162 MHz, 298 K, DMF): δ = 6.06 – –0.01 (m, 2P), –47.56 – –55.17 (m, 1P), –67.50 (dd, $^1J_{PP}$ = 403.1, 331.0 Hz, 1P), –77.23 (dtd, $^1J_{PP}$ = 406.8, 206.0, $^2J_{PP}$ = 53.1 Hz, 1P), –166.33 – –174.46 (m, 2P) ppm.

Elemental analysis for $C_{36}H_{58}InNa_2O_{12}P_7$: calcd.: C 40.77, H 5.51, N 0.0; found C 41.12, H 5.31, N 0.0.

1.5.9. Synthesis of $[K(18c6)]_2[(iBu_2Al)As_7]$, $[K(18c6)]_2[4]$

Following literature procedure,[6] to a Schlenk flask charged with a stir bar and $[K(18c6)]_2[HAs_7]$ (250.6 mg, 0.22 mmol. 1 eq.) THF (5 mL) was added and cooled to –30 °C forming a slurry. A solution of diisopropyl aluminium hydride (31.1 mg, 0.22 mmol. 1 eq.) in THF (5 mL) was cooled to –30 °C and dropwise added to the $[K(18c6)]_2[HAs_7]$ slurry. Gas evolution was observed and the reaction was allowed to react for 5 min at –30 °C, after which it was warmed to RT. The mixture was filtered yielding a clear black solution. The solvent was removed under reduced pressure and the residue was washed with toluene (2 x 20 mL). The residue was dissolved in THF and filtered again yielding a black solution. Removal of volatiles under reduced pressure yielded dark brown solids. Crystals suitable for X-ray diffraction analysis were obtained through slow diffusion of hexane into a concentrated *o*DFB solution. In solution the cluster was found to decompose slowly on exposure to light, forming presumably elemental As.

Isolated Yield: 101.4 mg, 36%.

^{1}H NMR (400 MHz, 298 K, THF-d$_8$): δ = 3.61 (s, 48H, *18-crown-6*, overlap with THF-d$_8$ residual resonance), 2.26 (th, $^3J_{HH}$ = 13.2, 6.6 Hz, 1H, $AlCH_2C\mathit{H}(CH_3)_2$), 1.55 (th, $^3J_{HH}$ = 13.3, 6.7 Hz, 1H, $AlCH_2C\mathit{H}(CH_3)_2$), 1.03 (d, $^3J_{HH}$ = 6.5 Hz, 6H, $AlCH_2CH(C\mathit{H_3})_2$), 0.66 (d, $^3J_{HH}$ = 6.5 Hz, 6H, $AlCH_2CH(C\mathit{H_3})_2$), 0.25 (d, $^3J_{HH}$ = 6.9 Hz, 2H, $AlC\mathit{H_2}CH(CH_3)_2$), –0.71 (d, $^3J_{HH}$ = 7.0 Hz, 2H, $AlC\mathit{H_2}CH(CH_3)_2$) ppm.

^{13}C{^{1}H} NMR (101 MHz, 298 K, THF-d$_8$): δ = 68.39 (s, *18-crown-6*), 29.69 (s, $Al^i\mathit{Bu_2}$), 28.35 (s, $Al^i\mathit{Bu_2}$), 26.76 (s, $Al^i\mathit{Bu_2}$), 26.38 (s, $Al^i\mathit{Bu_2}$), 25.96 (s, $Al^i\mathit{Bu_2}$), 25.82 (s, $Al^i\mathit{Bu_2}$) ppm.

Elemental analysis for $C_{32}H_{66}AlK_2O_{12}As_7$: calcd.: C 30.20, H 5.32, N 0.0; found C 30.61, H 5.09, N 0.0

1.5.10. Synthesis of $(Me_3Si)_3P_7$

Following literature procedure,[1] a Schlenk flask with a stir bar was charged with $[Na(DME)_x]_3[P_7]$ (3000.0 mg, ~10.09 mmol, 1 eq.) and the solids were suspended in toluene (10 mL). While the suspension was stirring, Me_3SiCl (3.97 mL, 31.28 mmol, 3.1 eq.) was added dropwise after which the reaction mixture was allowed to react overnight. The mixture was filtered and the black residue was further extracted with toluene (10 mL) combining both filtrates yielding a clear yellow solution. Removal of volatiles under reduced pressure yielded yellow solids.

Isolated Yield: 3931.5 mg, 89%.

^{1}H NMR (400 MHz, 298 K, Tol-d_8): δ = 0.05 (s) ppm.

^{29}Si{^{1}H} NMR (79 MHz, 298 K, Tol-d_8): δ = 7.26 (d, $^1J_{SiP}$ = 40.2 Hz) ppm.

^{31}P NMR (162 MHz, 298 K, Tol-d_8): δ = 4.24 – –5.12 (m, 3P, bridging P), –95.21 – –104.41 (m, 1P, apical P), –152.79 – –161.20 (m, 3P, basal P) ppm.

2. Optimization and control reactions for the reduction of N_2O, and Labelled Experiments

2.1. Reductant optimization

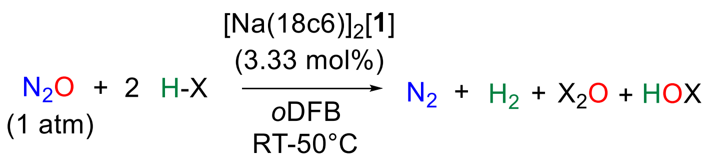

To a J Young NMR tube, a solution of $[Na(18c6)]_2$[**1**] (2.4 μmol, 0.033 eq.) in *o*DFB (0.5 mL) and reductant (0.074 mmol, 1.0 eq. silane and borane or 0.037 mmol 0.5 eq. diborane) was added. The reaction mixture was immediately frozen at –78 °C. While frozen, the headspace in the NMR tube was evacuated. The headspace was refilled with N_2O (1 atm) and the NMR tube was removed from the cooling bath and the solution was allowed to thaw. The reaction mixture was allowed to react for the specified time and temperature given in Table S1 below. The reaction was monitored by ^1H, ^{11}B and $^{11}B\{^1H\}$ NMR spectroscopy. Below are given the NMR spectra of entry 2 after 48h at RT as representative examples. NMR conv. was determined by the ratio HBpin/product by integration of the crude ^1H NMR spectrum.

Table S1. Optimization of the Reductant in the Catalytic N_2O Reduction.

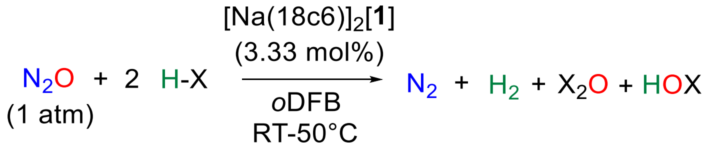

	Entry	Solvent	Reductant H–X	Temp. (°C)	Time (h)	Conv. X_2O (%)[a]	Conv. HOX (%)[a]	Total Conv. (%)[a]
reductant screening	1	*o*DFB	HBBN	RT	24	5	0	5
				50	24	25	0	25
	2	*o*DFB	HBpin	RT	24	55	0	55
					48	81	0	81
					5 days	>99	0	>99
	3	*o*DFB	HBcat	RT	24	0	0	0
				50	24	trace	0	trace
	4	*o*DFB	B_2pin_2	RT	24	0	0	0
				50	24	0	0	0
	5	*o*DFB	$HSiEt_3$	RT	24	0	0	0
				50	24	0	0	0
	6	*o*DFB	$HSiPh_3$	RT	24	0	0	0
				50	24	0	0	0
Blank control	7[b]	*o*DFB	HBpin	100	24	0	0	0

[a] NMR conv. was determined by the ratio reductant/product by integration of the crude ^1H NMR spectrum or by integration of the crude ^1H NMR spectrum using C_6H_6 or toluene as internal standard. [b] No catalyst added.

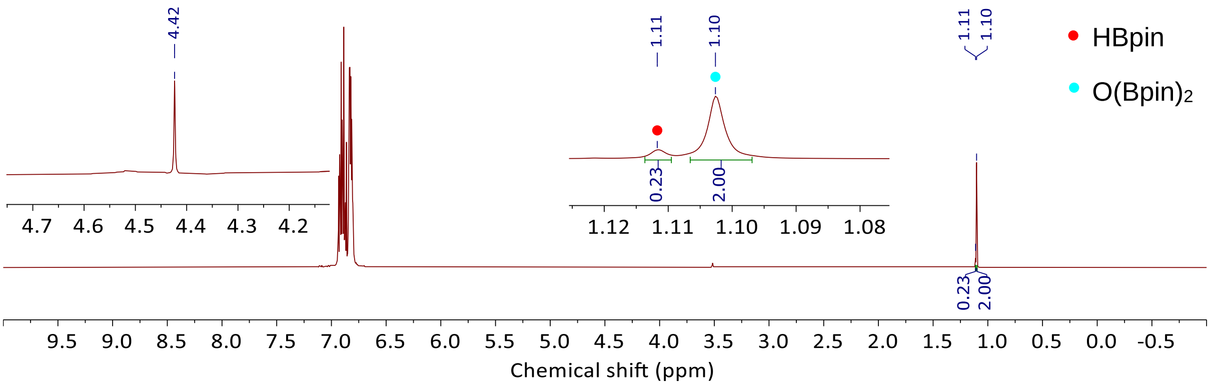

Figure S9. ^1H NMR spectrum (400 MHz, *o*DFB) of the hydroboration of N_2O catalyzed by $[Na(18c6)]_2[\mathbf{1}]$ using HBpin in *o*DFB.

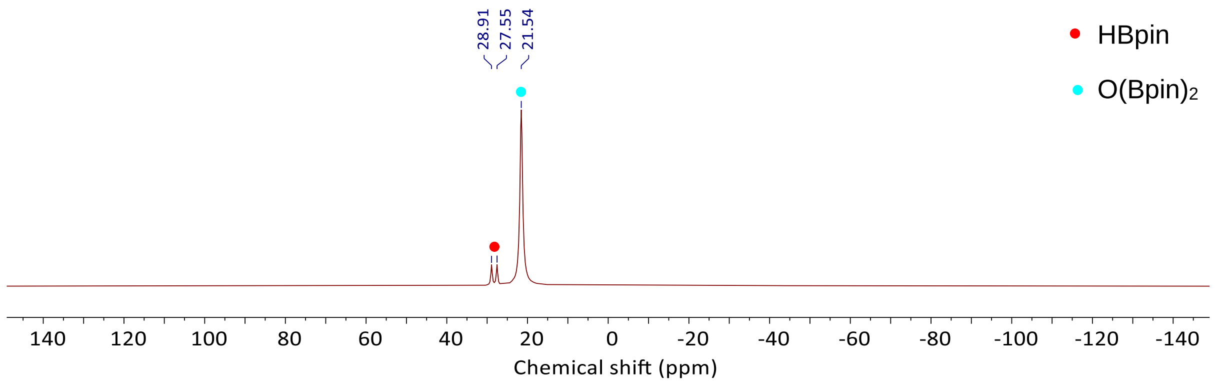

Figure S10. ^{11}B NMR spectrum (128 MHz, *o*DFB) of the hydroboration of N_2O catalyzed by $[Na(18c6)]_2$[**1**] using HBpin in *o*DFB.

2.2. Solvent optimization

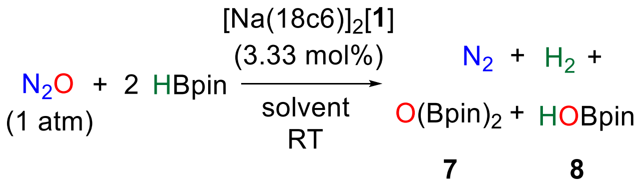

To a J Young NMR tube, $[Na(18c6)]_2$[**1**] (2.4 µmol, 0.033 eq.), solvent (0.5 mL) and HBpin (10.7 µL, 0.074 mmol, 1.0 eq.) was added. Toluene (25 µL, 0.24 mmol) was added as an internal standard (or C_6H_6 (10 µL) when DMF was used). The reaction mixture was immediately frozen at –78 °C. While frozen, the headspace in the NMR tube was evacuated. The headspace was refilled with N_2O (1 atm) and the NMR tube was removed from the cooling bath and the solution was allowed to thaw. The reaction mixture was monitored by ^{1}H, ^{11}B and $^{11}B\{^{1}H\}$ NMR spectroscopy. NMR conv. was determined by integration of the crude ^{1}H NMR spectrum using the toluene as internal standard (^{1}H δ = 2.31 ppm) (or C_6H_6 ^{1}H δ = 7.35 ppm for Table S2, entry 4). Below are given the NMR spectra of entry 4, Table S2 as representative examples.

It is noteworthy that when solvents *o*DFB, C_6D_6, THF and Pyr were studied, a black insoluble precipitate often formed during the catalysis. However, negligible to no precipitate formation was observed when DMF was employed as the solvent and complete conversion to **7** was obtained after 30 minutes, and thus DMF was selected as the solvent for subsequent studies.

Table S2. Optimization of the Solvent in the Catalytic N_2O Reduction.

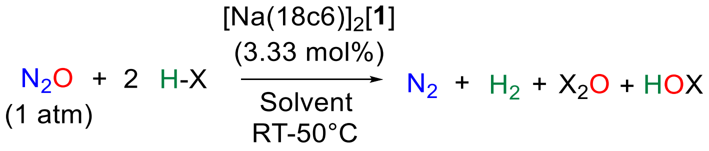

	Entry	Solvent	Reductant H–X	Temp. (°C)	Time (h)	Conv. X_2O (%)[a]	Conv. HOX (%)[a]	Total Conv. (%)[a]
solvent screening	1	C_6D_6	HBpin	RT	24	38	0	38
	2	THF	HBpin	RT	24	88	0	88
	3	Pyr	HBpin	RT	4	85	14	>99
	4	DMF	HBpin	RT	0.5	>99	0	>99

[a] NMR conv. was determined by the ratio reductant/product by integration of the crude ^1H NMR spectrum or by integration of the crude ^1H NMR spectrum using C_6H_6 or toluene as internal standard.

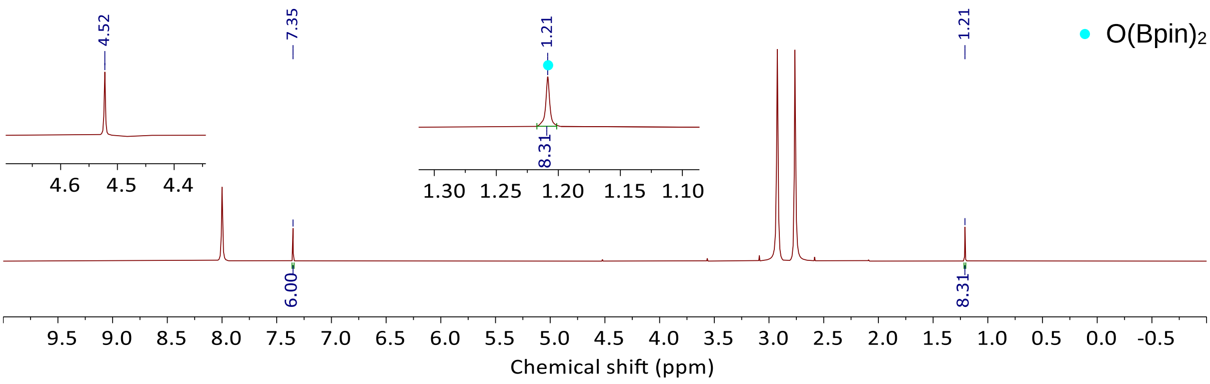

Figure S11. ^1H NMR spectrum (400 MHz, DMF) of the hydroboration of N_2O catalyzed by $[Na(18c6)]_2[\mathbf{1}]$ using HBpin.

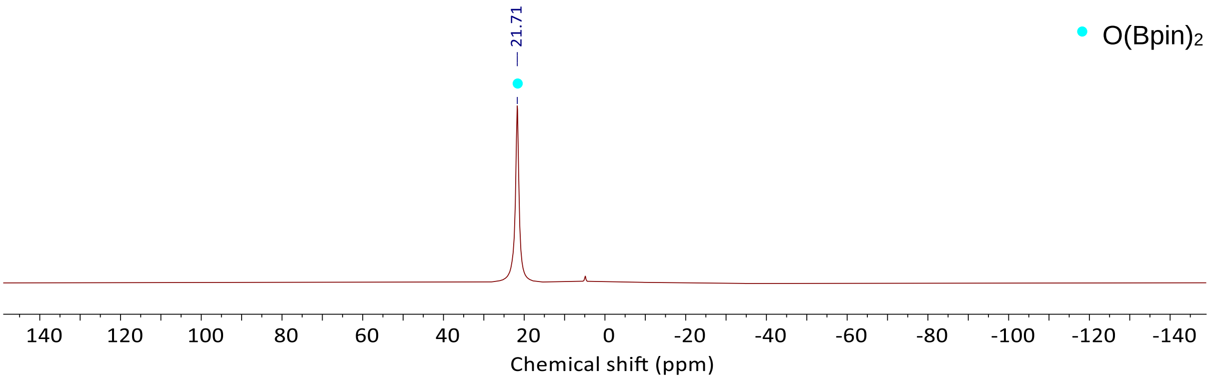

Figure S12. ^1H NMR spectrum (128 MHz, DMF) of the hydroboration of N_2O catalyzed by $[Na(18c6)]_2[\mathbf{1}]$ using HBpin.

2.3. ^{15}N-labelled N_2O experiment

Conversion to N_2 was confirmed by employing ^{15}N-labelled N_2O gas. To a J Young NMR tube, catalyst (0.7 µmol, 0.0025 eq.) in DMF (0.5 mL) and HBpin (42 µL, 0.296 mmol, 1.0 eq.) were added. C_6H_6 (10 µL, 0.11 mmol) was added as an internal standard (^{1}H δ = 7.35 ppm). The reaction mixture was immediately frozen at –78 °C. While frozen, the headspace in the NMR tube was evacuated. The headspace was refilled with $^{15}N_2O$ (1 atm) and the NMR tube was removed from the cooling bath and the solution was allowed to thaw. The reaction mixture was monitored by ^{1}H, ^{11}B and ^{15}N NMR spectroscopy. A control reaction was performed where no catalyst was added.

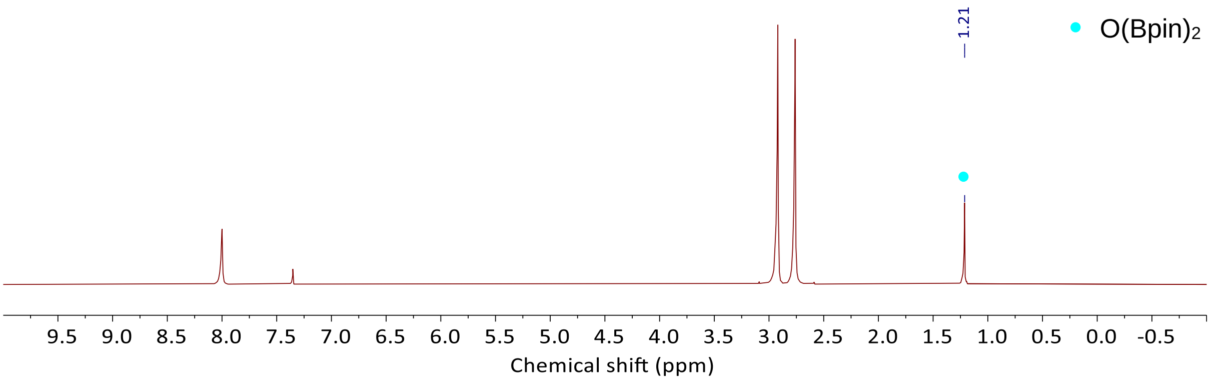

Figure S13. ^{1}H NMR spectrum (400 MHz, DMF) of the hydroboration of $^{15}N_2O$ catalyzed by [K(18c6)]$_3$[**6**] using HBpin in DMF.

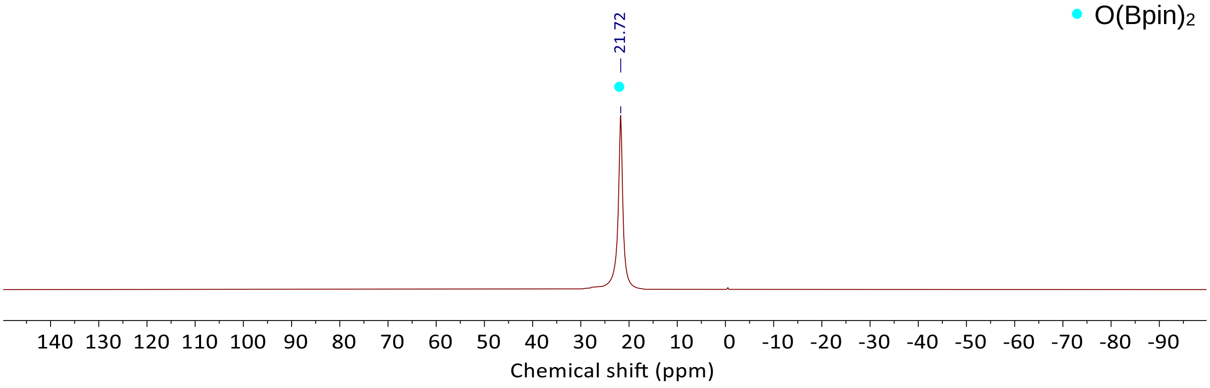

Figure S14. ^{11}B NMR spectrum (128 MHz, DMF) of the hydroboration of $^{15}N_2O$ catalyzed by [K(18c6)]$_3$[**6**] using HBpin in DMF.

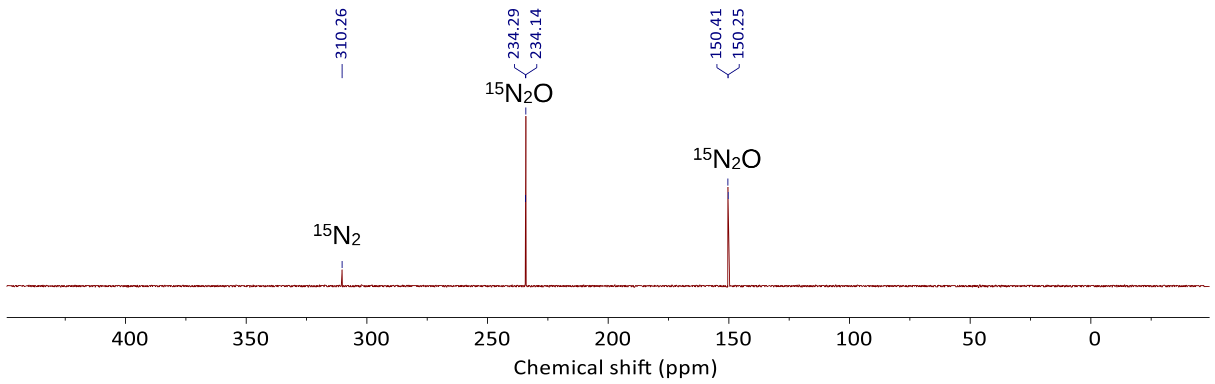

Figure S15. ^{15}N NMR spectrum (51 MHz, DMF) of the hydroboration of $^{15}N_2O$ catalyzed by $[K(18c6)]_3$[**6**] using HBpin in DMF.

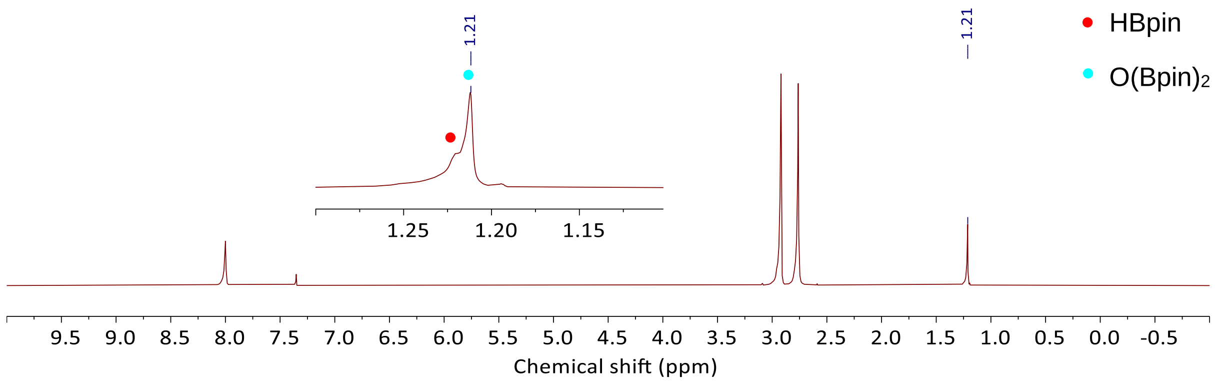

Figure S16. ^1H NMR spectrum (400 MHz, DMF) of the hydroboration of $^{15}N_2O$ catalyzed by $[K(crypt)]_2$[**9**] using HBpin in DMF.

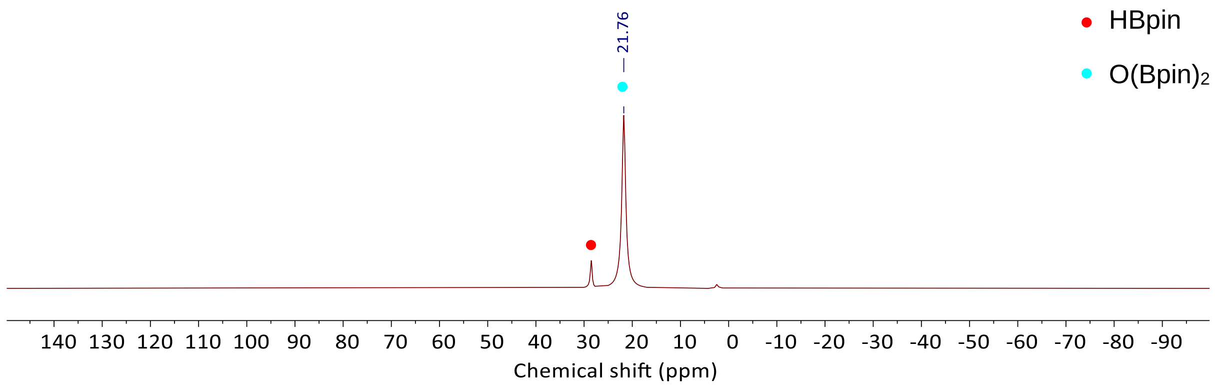

Figure S17. $^{11}B\{^1H\}$ NMR spectrum (128 MHz, DMF) of the hydroboration of $^{15}N_2O$ catalyzed by $[K(crypt)]_2$[**9**] using HBpin in DMF.

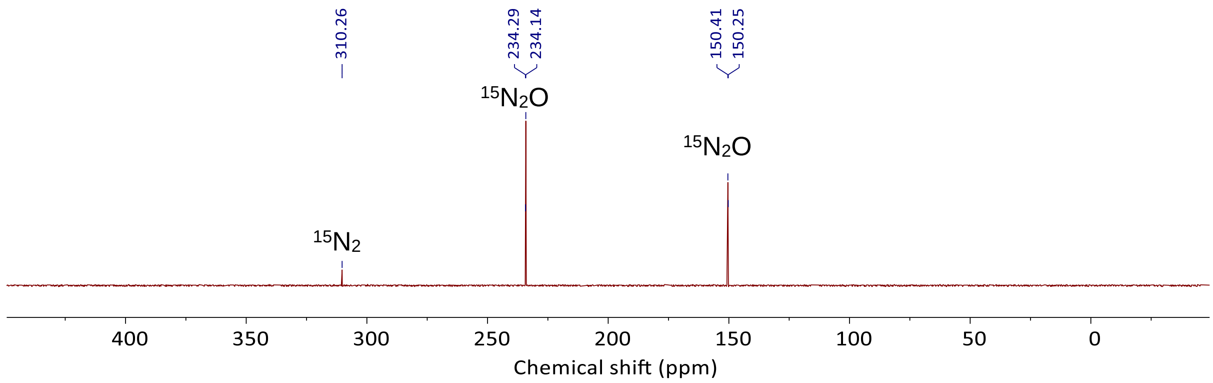

Figure S18. ^{15}N NMR spectrum (51 MHz, DMF) of the hydroboration of $^{15}N_2O$ catalyzed by $[K(crypt)]_2$[**9**] using HBpin in DMF.

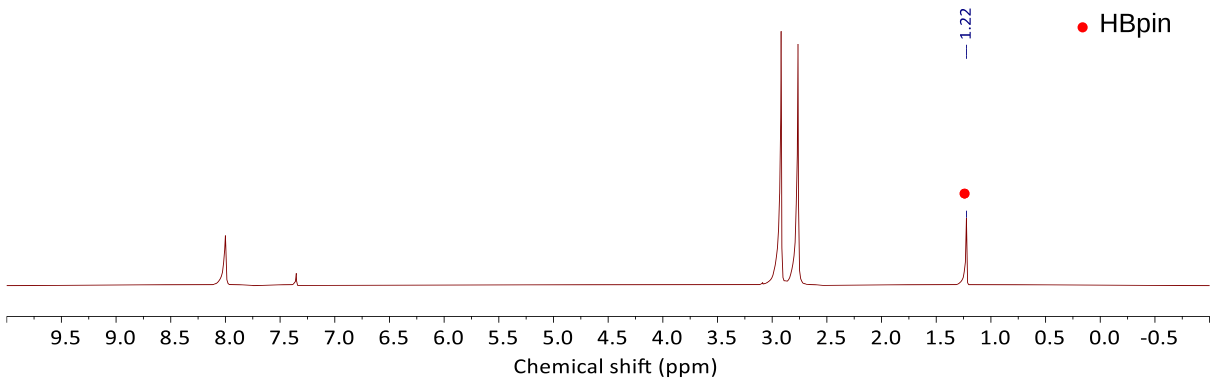

Figure S19. ^{1}H NMR spectrum (400 MHz, DMF) of the hydroboration of $^{15}N_2O$ control reaction with no catalyst.

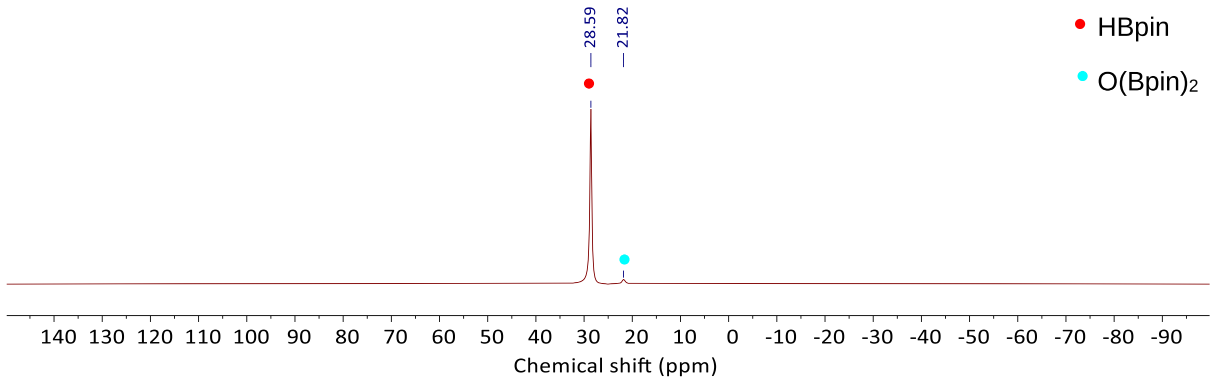

Figure S20. ^{11}B NMR spectrum (128 MHz, DMF) of the hydroboration of $^{15}N_2O$ control reaction with no catalyst.

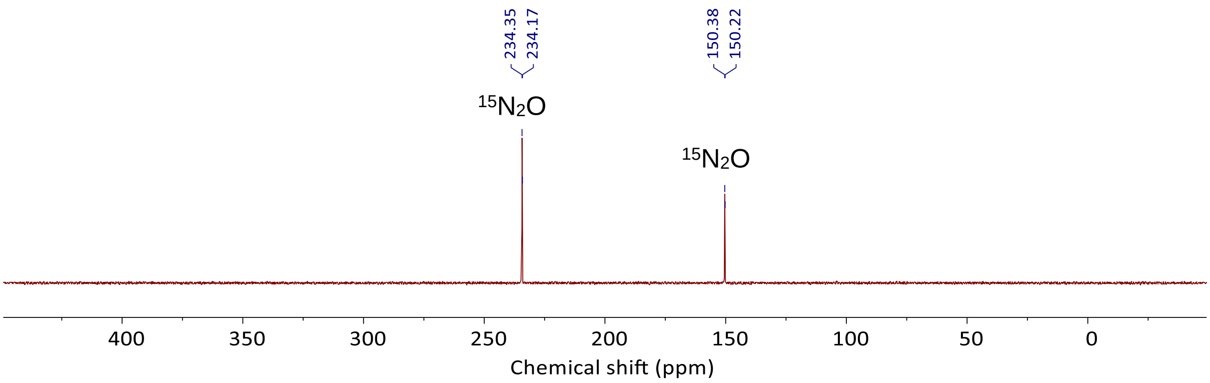

Figure S21. ^{15}N NMR spectrum (51 MHz, DMF) of the hydroboration of $^{15}N_2O$ control reaction with no catalyst.

2.4. DBpin labelled reactions

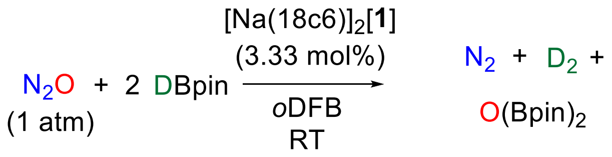

7

To a J Young NMR tube, $[Na(18c6)]_2$[**1**] (2.4 μmol, 0.033 eq.) in THF (0.5 mL) and DBpin (0.074 mmol, 1.0 eq.) in 44 μL C_6D_6 was added. The reaction mixture was immediately frozen at –78 °C. While frozen, the headspace in the NMR tube was evacuated. The headspace was refilled with N_2O (1 atm) and the NMR tube was removed from the cooling bath and the solution was allowed to thaw. The reaction mixture was monitored by ^{1}H, ^{2}H, and ^{11}B NMR spectroscopy.

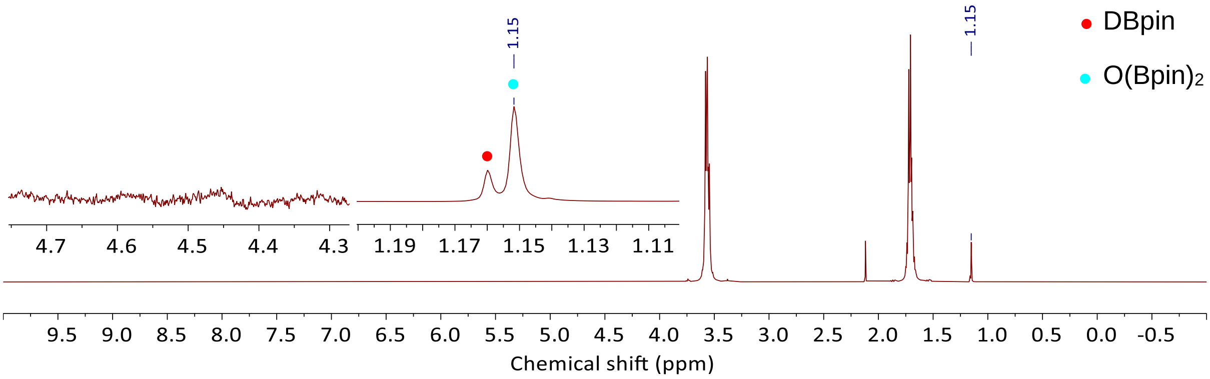

Figure S22. ^{1}H NMR spectrum (400 MHz, THF) of the hydroboration of N_2O catalyzed by $[Na(18c6)]_2$[**1**] using DBpin. Note: no H_2 has observed.

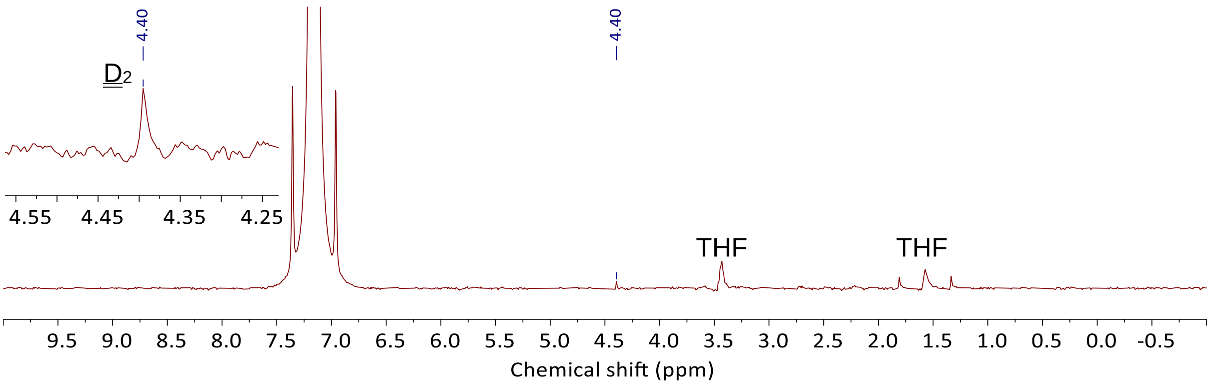

Figure S23. ^{2}H NMR spectrum (61 MHz, THF) of the hydroboration of N_2O catalyzed by $[Na(18c6)]_2[\mathbf{1}]$ using DBpin.

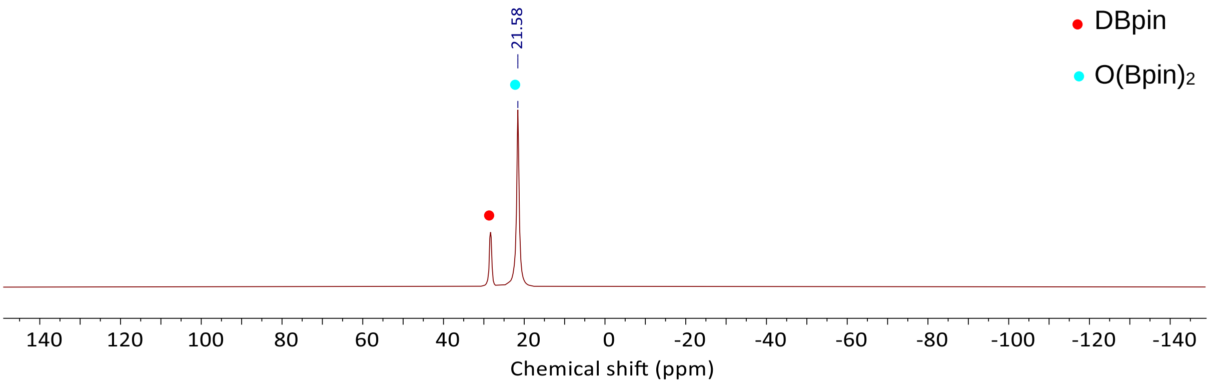

Figure S24. ^{11}B NMR spectrum (128 MHz, THF) of the hydroboration of N_2O catalyzed by $[Na(18c6)]_2[\mathbf{1}]$ using DBpin.

2.5. Catalyst optimization

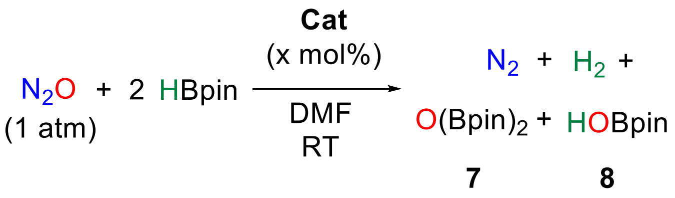

To a J Young NMR tube, a solution of catalyst (0.7 µmol, 0.01 eq.) in DMF (0.5 mL) and HBpin (10.7 µL, 0.074 mmol, 1.0 eq.) was added. C_6H_6 (10 µL, 0.11 mmol) was added as an internal standard. The reaction mixture was immediately frozen at –78 °C. While frozen, the headspace in the NMR tube was evacuated. The headspace was refilled with N_2O (1 atm) and the NMR tube was removed from the cooling bath and the solution was allowed to thaw. The reaction mixture was monitored by ^{1}H, ^{11}B and $^{11}B\{^{1}H\}$ NMR spectroscopy. NMR conv. was determined by integration of the crude ^{1}H NMR spectrum using the C_6H_6 as internal standard (^{1}H δ = 7.35 ppm). Below are given

the NMR spectra of Table S3, entry 9 as representative examples. Where trimethylamine (side-product from the hydroboration of DMF, ^{1}H $\delta = 2.11$ ppm) was observed, the NMR conv. was corrected accordingly.

Table S3. Screening Group 15 Clusters in the Catalytic N_2O Reduction.

$$\underset{(1\ \text{atm})}{N_2O} + 2\ \text{HBpin} \xrightarrow[\text{DMF, RT}]{\textbf{Cat}\ (x\ \text{mol\%})} N_2 + H_2 + \underset{\mathbf{7}}{\text{O(Bpin)}_2} + \underset{\mathbf{8}}{\text{HOBpin}}$$

	Entry	Cat	x mol%	Time (h)	**7** Conv. (%)[a]	**8** Conv. (%)[a]	Total Conv. (%)[a]
catalyst screening	1	none	-	24	0	0	0
	2	$[Na(18c6)]_2$[**1**]	1	0.5	55	0	55
			1	1	82[b]	0	82
	3	$[Na(18c6)]_2$[**2**]	1	0.5	28[b]	0	28
			1	1	60[b]	0	60
	4	$[Na(18c6)]_2$[**3**]	1	0.5	3	0	3
			1	1	8	0	8
	5	$[K(18c6)]_2$[**4**]	1	0.5	63	5	68
			1	1	96	3	>99
	6		0.1	60	94	0	94
	7	$[K(18c6)]_3$[**5**]	1	0.5	54	0	54
			1	1	74	11	85
	8		0.1	48	95	0	95
	9	$[K(18c6)]_3$[**6**]	1	0.5	52	28	80
			1	1	68	31	>99
	10		0.1	48	95	0	95
	11	K_3Sb_7 + 18c6	1	1	30	8	38
			1	3	78	21	>99
	12	K_5Bi_4 + 18c6	1	1	45	0	45

[a] NMR conv. was determined by integration of the crude ^{1}H NMR spectrum using C_6H_6 as internal standard. [b] Corrected for DMF hydroboration side reaction.

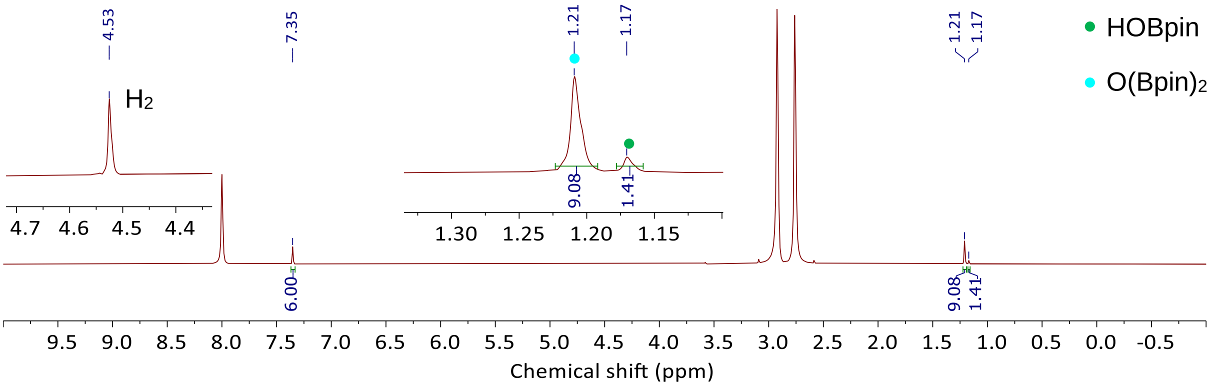

Figure S25. ^{1}H NMR spectrum (400 MHz, DMF) of the hydroboration of N_2O catalyzed by [K(18c6)]$_3$[**6**] using HBpin.

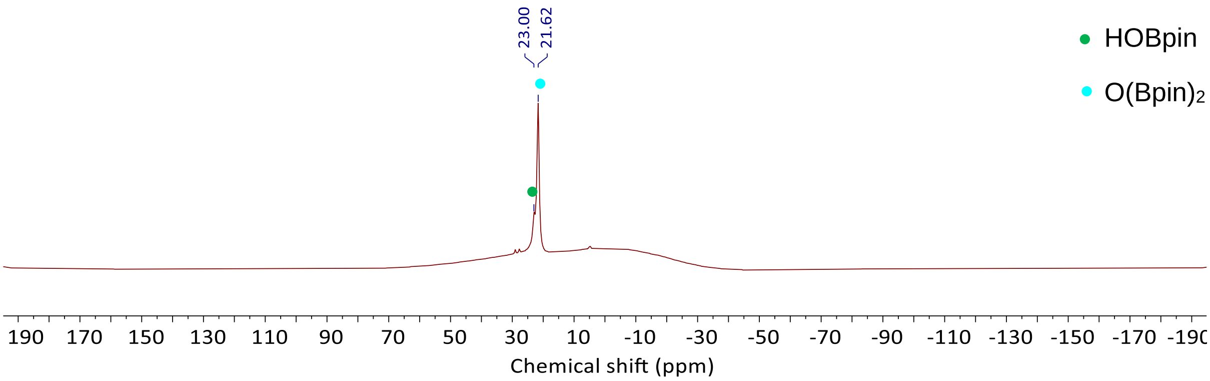

Figure S26. ^{11}B NMR spectrum (128 MHz, DMF) of the hydroboration of N_2O catalyzed by [K(18c6)]$_3$[**6**] using HBpin.

2.6. Control reactions

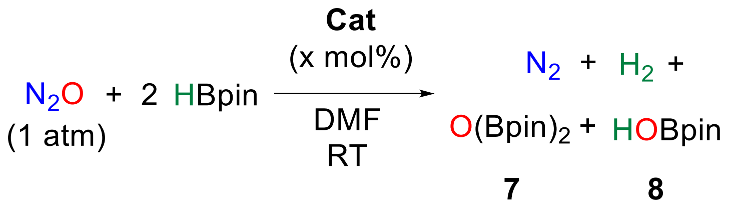

To a J Young NMR tube, catalyst (specific loading given in Table S4) in DMF (0.5 mL) and HBpin (10.7 µL, 0.074 mmol, 1.0 eq.) were added. C_6H_6 (10 µL, 0.11 mmol) was added as an internal standard. The reaction mixture was immediately frozen at –78 °C. While frozen, the headspace in the NMR tube was evacuated. The headspace was

refilled with N_2O (1 atm) and the NMR tube was removed from the cooling bath and the solution was allowed to thaw. The reaction mixture was monitored by ^{1}H, ^{11}B and $^{11}B\{^{1}H\}$ NMR spectroscopy. NMR conv. was determined by integration of the crude ^{1}H NMR spectrum using the C_6H_6 as internal standard (^{1}H δ = 7.35 ppm). Where trimethylamine (side-product from the hydroboration of DMF, ^{1}H δ = 2.11 ppm) was observed, the NMR conv. was corrected accordingly.

Table S4. Control Reactions in the Catalytic N_2O Reduction

$$\underset{(1\ \text{atm})}{N_2O} + 2\ \text{HBpin} \xrightarrow[\text{DMF, RT}]{\textbf{Cat}\ (x\ \text{mol\%})} N_2 + H_2 + \underset{\textbf{7}}{O(\text{Bpin})_2} + \underset{\textbf{8}}{\text{HOBpin}}$$

	Entry	Cat	x mol%	Time (h)	**7** Conv. (%)[a]	**8** Conv. (%)[a]	Total Conv. (%)[a]
controls	1	$BH_3 \bullet SMe_2$	10	24	0	0	0
	2	$K[B(OMe)_4]$	5	24	0	0	0
	3	NaOTf + 18c6	5	24	0	0	0
	4	$B(C_6F_5)_3$ + P^tBu_3	5	24	0	0	0
	5	Ph_3P	5	24	0	0	0
	6	Ph_3As	5	24	0	0	0
	7	$KPPh_2$ + 18c6	3	1	15[b]	0	15[b]
	8	P_4S_3	1	24	10	0	10
	9	$(Me_3Si)_3P_7$	1	24	26	1	27

[a] NMR conv. was determined by integration of the crude ^{1}H NMR spectrum using C_6H_6 as internal standard. [b] Corrected for DMF hydroboration side reaction.

2.7. Comparison to Literature Catalysts for N_2O Reduction

The table below is not an exhaustive list; the homogeneous catalysts below are selected because they reduce N_2O in a similar fashion to the work in this study.

Table S5. Comparison of TON and TOF for Catalysts Reported for N_2O Reduction.

Catalysts	Cat. loading (mol%)	Reductant	Pressure (atm)	Solvent	Temp. (°C)	TON_{max}	TOF_{max} (h^{-1})
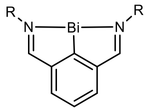 11	0.01-1	HBpin	1	THF-d_8	25	6700	3120[c]
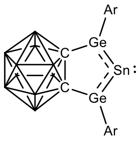 12	0.01-5	HBpin	1	C_6D_6	60	9200	55
KOR or CsF [13]	10	Me_6Si_2	1	DMSO-d_6	20	10	10
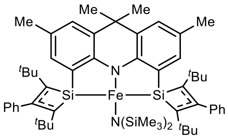 14	0.05-2.5	HBpin	1	C_6D_6	50	1400	58
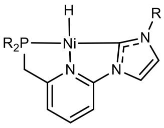 15	0.5-2.0	HBpin	2	THF-d_8	RT	200	100
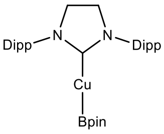 16	0.1-1	B_2Pin_2	1	C_6H_6	80	859	36
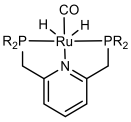 17	0.23	*pH₂* 4 atm	*pN₂O* 3 atm	THF	65	417	9
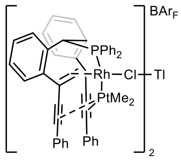 18	-	*pH₂* 2 atm	*pN₂O* 2 atm	THF	RT	587	12
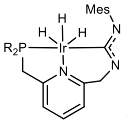 19	-	*pH₂* 1 atm	*pN₂O* 1 atm	THF	55	328	16
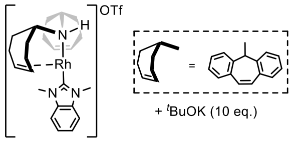 20	0.004	*pH₂* 1.5-3 atm	*pN₂O* 1.5-3 atm	THF	65-80	235000	1300
[K(18c6)]₃[**5**] [this work]	1	HBpin	1	DMF	RT	100[a]	85
[K(18c6)]₃[**6**] [this work]	1	HBpin	1	DMF	RT	100[b]	160
[K(crypt)]₂[**9**] [this work]	0.1-1	HBpin	1	DMF	RT	1000	100

pH_2 = partial pressure hydrogen. *pN_2O* = partial pressure nitrous oxide.
[a] not including recycling experiments, including recycling TON_{max} = 916.
[b] not including recycling experiments, including recycling TON_{max} = 921.
[c] this turnover frequency is determined by using a visual colour change that is observed as the time of the reaction, not a quantified spectroscopy.

3. Mechanistic studies

3.1. Addition of HBpin to [K(18c6)]$_3$[**5**]

To a J Young NMR tube, a solution of [K(18c6)]$_3$[**5**] (30.0 mg, 0.026 mmol, 1 eq.) in DMF (0.5 mL) and HBpin (3.7 µL, 0.026 mmol, 1 eq.) was added and allowed to react for 30 min. The reaction was monitored by ^{1}H, ^{11}B, ^{11}B{^{1}H} and ^{31}P NMR spectroscopy. Subsequent addition of N_2O to the solution did not result in any changes observed by ^{1}H and ^{11}B NMR spectroscopy.

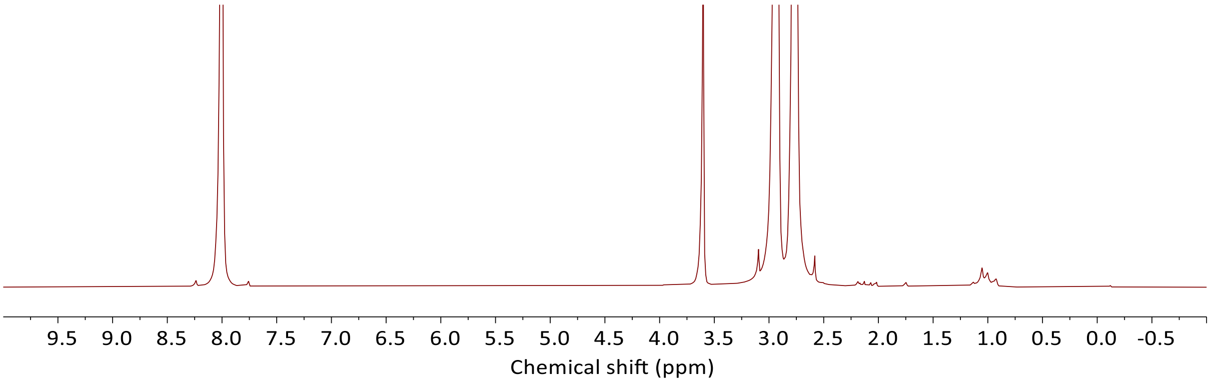

Figure S27. ^{1}H NMR spectrum (400 MHz, DMF) of the addition of HBpin to [K(18c6)]$_3$[**5**].

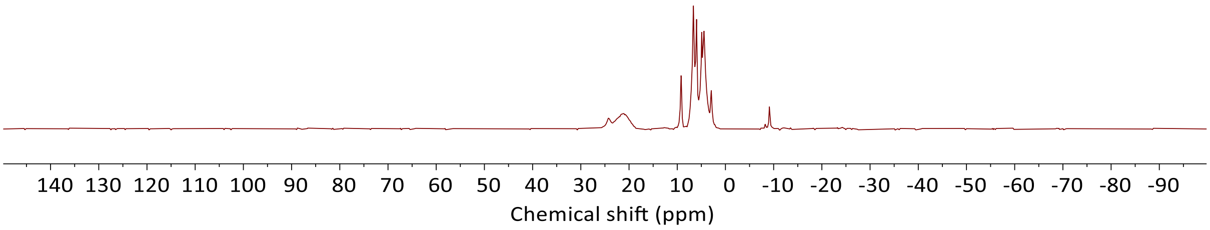

Figure S28. ^{11}B NMR spectrum (128 MHz, DMF) of the addition of HBpin to [K(18c6)]$_3$[**5**].

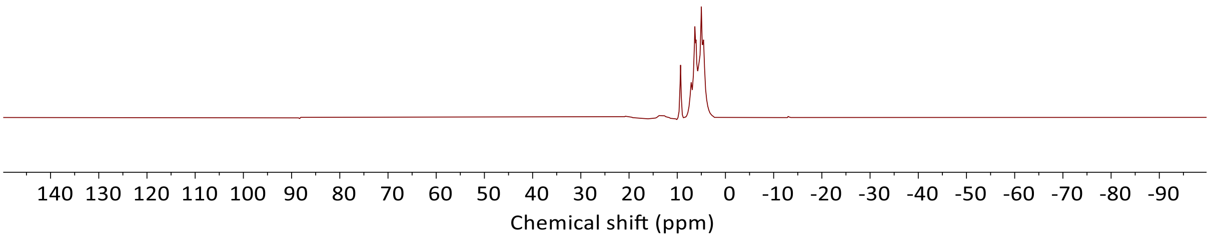

Figure S29. $^{11}B\{^1H\}$ NMR spectrum (128 MHz, DMF) of the addition of HBpin to $[K(18c6)]_3[$**5**$]$.

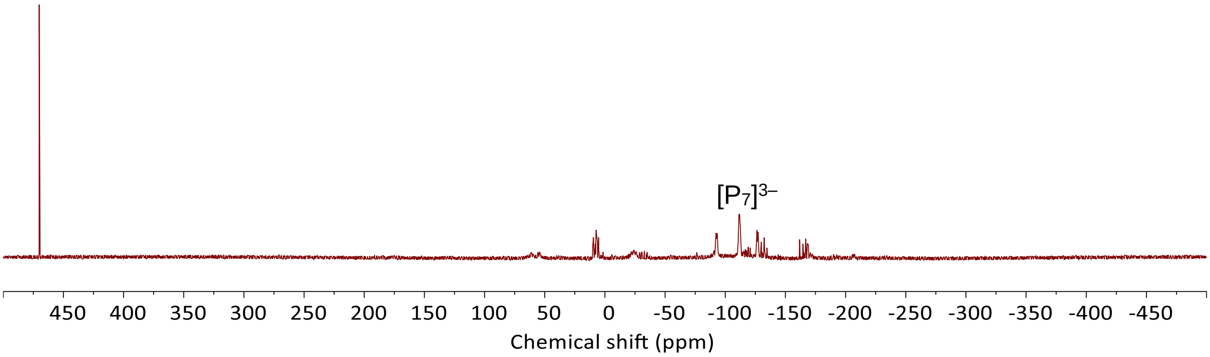

Figure S30. ^{31}P NMR spectrum (162 MHz, DMF) of the addition of HBpin to $[K(18c6)]_3[$**5**$]$.

3.2. Addition of HBpin to $[K(18c6)]_3[6]$

To a J Young NMR tube, a solution of $[K(18c6)]_3[$**6**$]$ (37.0 mg, 0.026 µmol, 1 eq.) in DMF (0.5 mL) and HBpin (3.7 µL, 0.026 mmol, 1 eq.) was added and allowed to react for 30 min. The reaction was monitored by ^1H, ^{11}B and $^{11}B\{^1H\}$ NMR spectroscopy. Subsequent addition of N_2O to the solution did not result in any changes observed by ^1H and ^{11}B NMR spectroscopy.

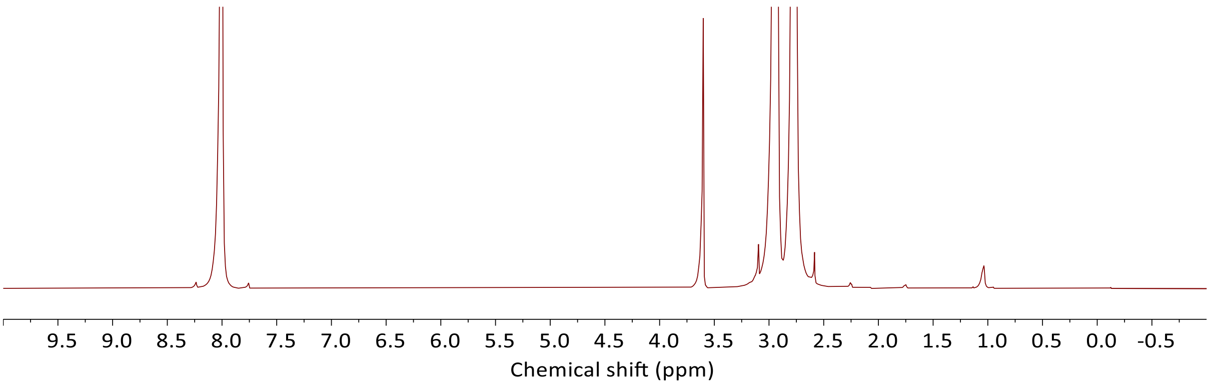

Figure S31. ^{1}H NMR spectrum (400 MHz, DMF) of the addition of HBpin to $[K(18c6)]_3$[**6**].

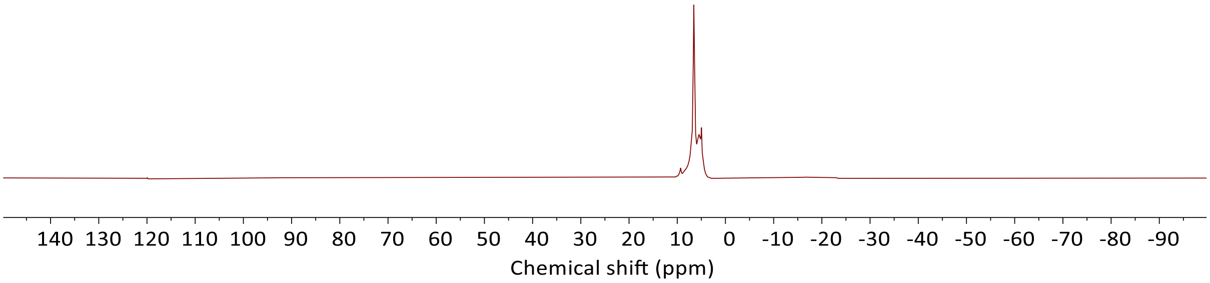

Figure S32. ^{11}B NMR spectrum (128 MHz, DMF) of the addition of HBpin to $[K(18c6)]_3$[**6**].

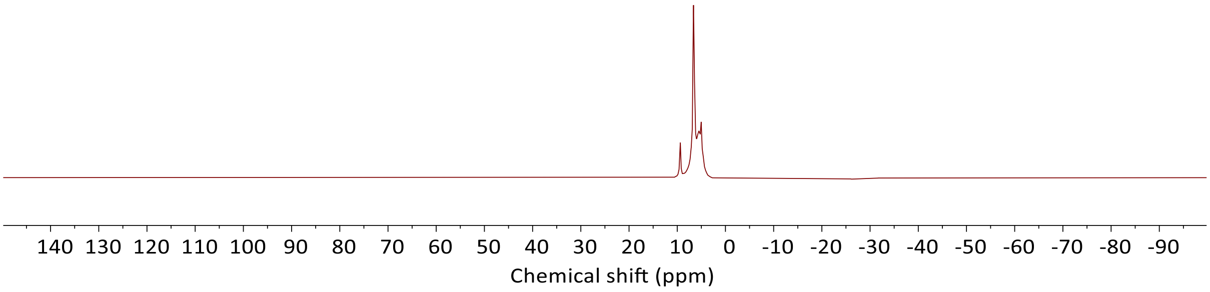

Figure S33. ^{11}B{^{1}H} NMR spectrum (128 MHz, DMF) of the addition of HBpin to $[K(18c6)]_3$[**6**].

3.3. [K(18c6)]$_3$[5] and HBpin frozen, and then addition of N_2O

To a J Young ampoule, a solution of [K(18c6)]$_3$[**5**] (30.0 mg, 0.026 mmol, 1 eq.) in DMF (0.2 mL) was added. To a separate ampoule a solution of HBpin (3.7 µL, 0.026 mmol, 1 eq.) in DMF (0.4 mL) was added. The [K(18c6)]$_3$[**5**] solution was frozen at –78 °C. The HBpin solution was added onto the frozen [K(18c6)]$_3$[**5**] solution, instantly freezing. While frozen, the headspace in the ampoule was evacuated. The headspace was refilled with N_2O (1 atm) and the ampoule was removed from the cooling bath and the solution was allowed to thaw. The reaction was allowed to react for 30 min before it was transferred into a J Young NMR tube, benzene was added to the reaction as an internal standard. The reaction was analyzed by ^1H, ^{11}B, and $^{11}B\{^1H\}$ NMR spectroscopy. The reaction was repeated as described above and then additional HBpin (3.7 µL, 0.026 mmol, 1 eq.) was added, after which the reaction mixture was analyzed by ^1H and ^{11}B NMR spectroscopy. Compound **8** was observed when only 1 eq. of HBpin was utilized, which was found to be consumed to give **7** when the additional equivalent of HBpin was added.

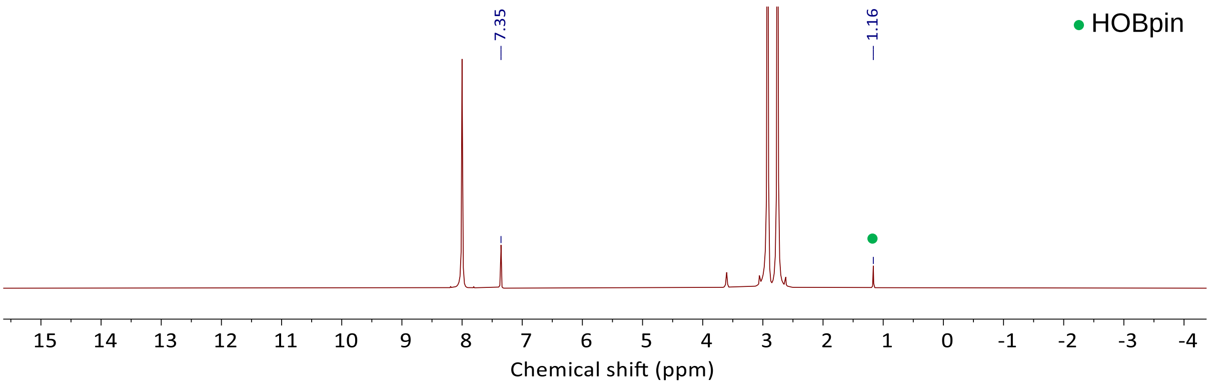

Figure S34. ^1H NMR spectrum (400 MHz, DMF) of reaction when [K(18c6)]$_3$[**5**] and HBpin (1 eq.) are flash frozen and then N_2O added.

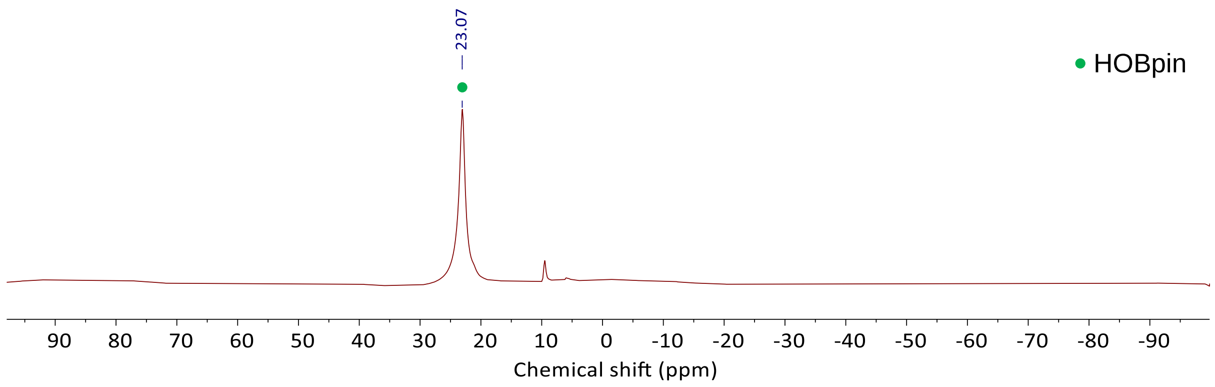

Figure S35. ^{11}B NMR spectrum (128 MHz, DMF) of reaction when $[K(18c6)]_3$[**5**] and HBpin (1 eq.) are flash frozen and then N_2O added.

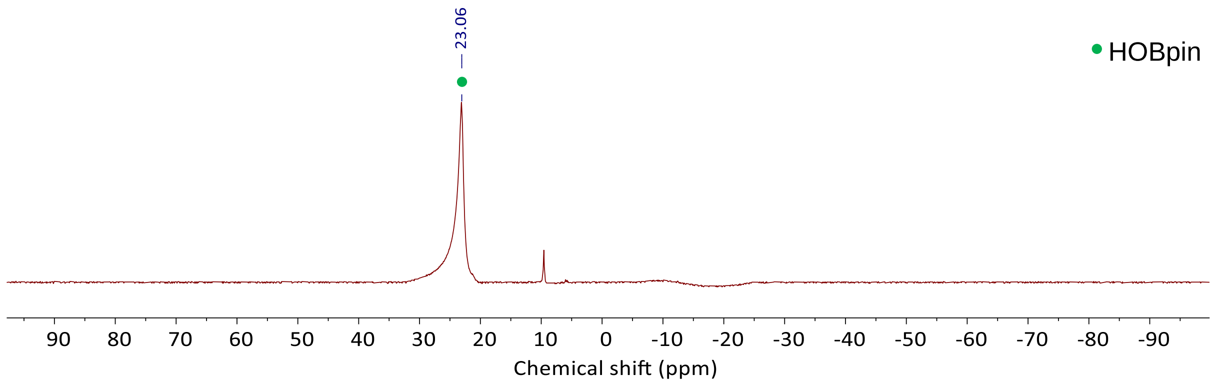

Figure S36. $^{11}B\{^1H\}$ NMR spectrum (128 MHz, DMF) of reaction when $[K(18c6)]_3$[**5**] and HBpin (1 eq.) are flash frozen and then N_2O added.

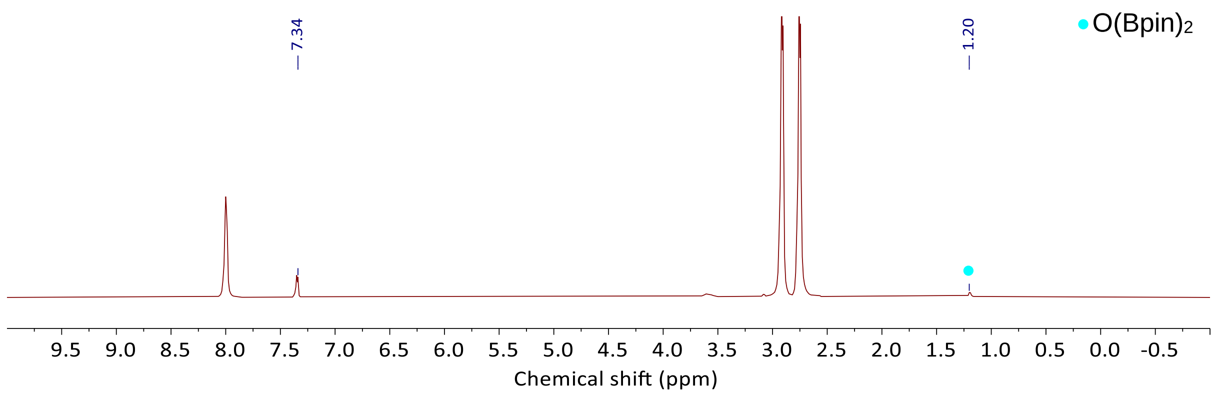

Figure S37. ^1H NMR spectrum (128 MHz, DMF) of reaction when $[K(18c6)]_3$[**5**] and HBpin (1 eq.) are flash frozen, then N_2O added, and then additional HBpin added.

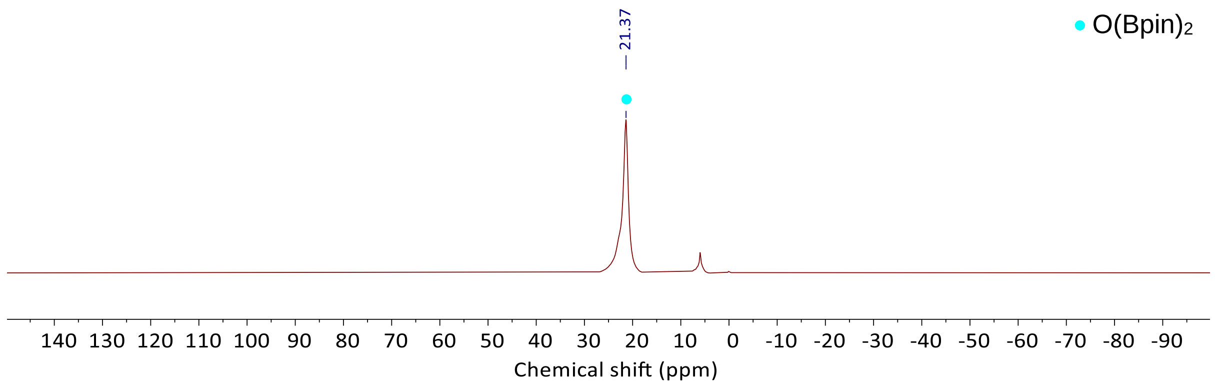

Figure S38. $^{11}B\{^{1}H\}$ NMR spectrum (128 MHz, DMF) of reaction when [K(18c6)]$_3$[**5**] and HBpin (1 eq.) are flash frozen, then N_2O added, and then additional HBpin added.

3.4. K(18c6)]$_3$[6] and HBpin frozen, and then addition of N_2O

To a J Young ampoule, a solution of [K(18c6)]$_3$[**6**] (37.0 mg, 0.026 mmol, 1 eq.) in DMF (0.2 mL) was added. To a separate ampoule a solution of HBpin (3.7 µL, 0.026 mmol, 1 eq.) in DMF (0.4 mL) was added. The [K(18c6)]$_3$[**6**] solution was frozen at –78 °C. The HBpin solution was added onto the frozen [K(18c6)]$_3$[**6**] solution, instantly freezing. While frozen, the headspace in the ampoule was evacuated. The headspace was refilled with N_2O (1 atm) and the ampoule was removed from the cooling bath and the solution was allowed to thaw. The reaction was allowed to react for 30 min before it was transferred into a J Young NMR tube, benzene was added to the reaction as an internal standard. The reaction was analyzed by ^{1}H, ^{11}B, and $^{11}B\{^{1}H\}$ spectroscopy. The reaction was repeated as described above and then additional HBpin (3.7 µL, 0.026 mmol, 1 eq.) was added, after which the reaction mixture was analyzed by ^{1}H and ^{11}B NMR spectroscopy. Compound **8** was observed when only 1 eq. of HBpin was utilized, which was found to be consumed to give **7** when the additional equivalent of HBpin was added.

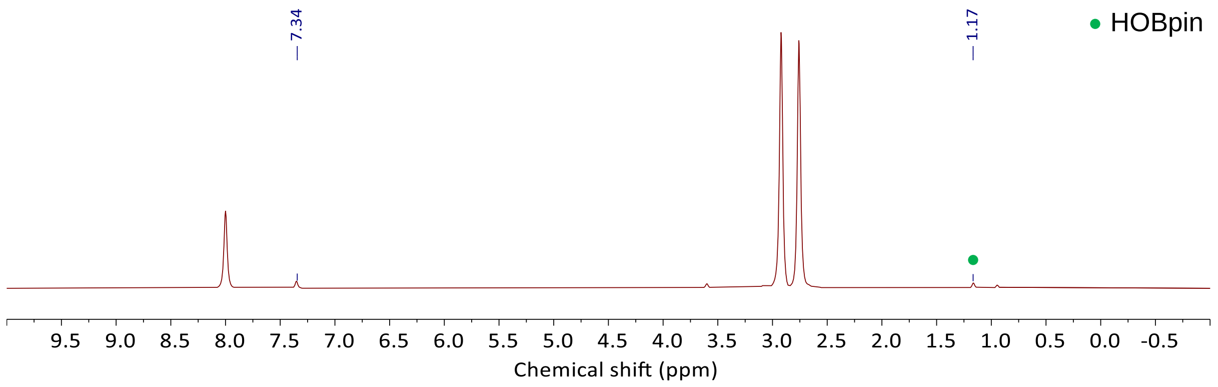

Figure S39. ^{1}H NMR spectrum (400 MHz, DMF) of reaction when $[K(18c6)]_3$[**6**] and HBpin (1 eq.) are flash frozen and then N_2O added.

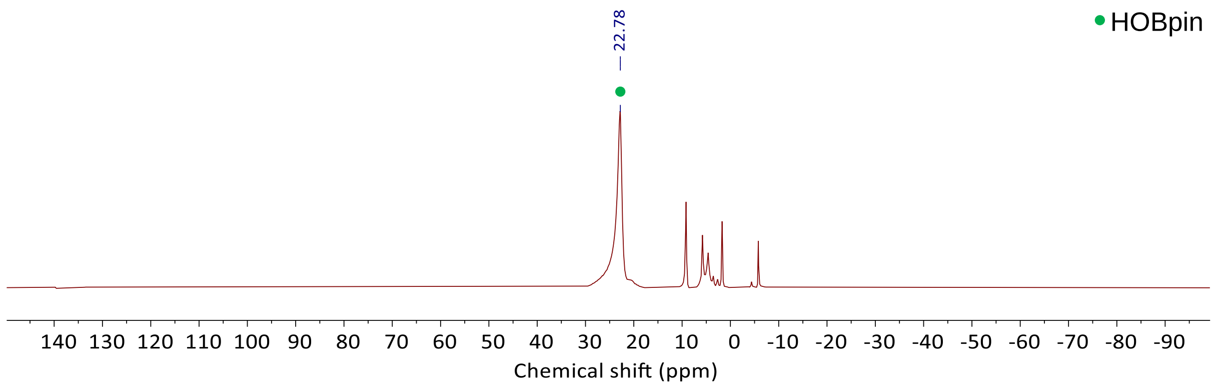

Figure S40. ^{11}B NMR spectrum (128 MHz, DMF) of reaction when $[K(18c6)]_3$[**6**] and HBpin (1 eq.) are flash frozen and then N_2O added.

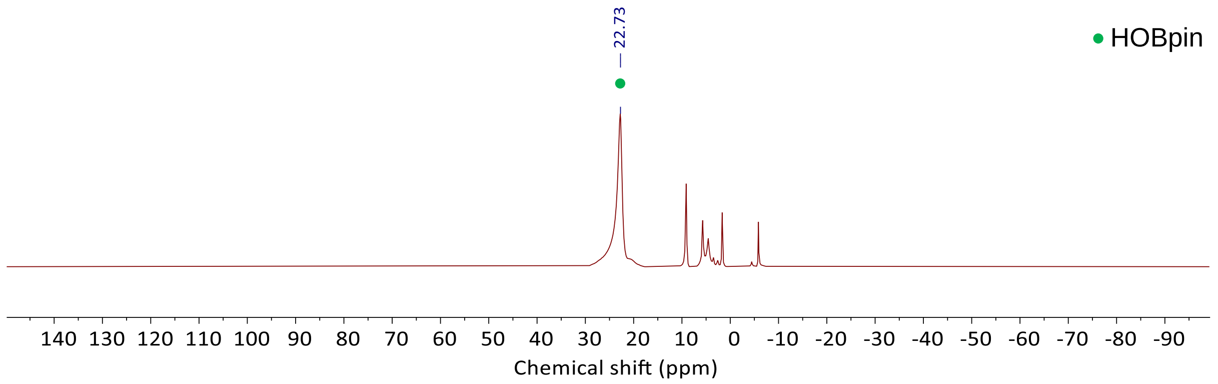

Figure S41. $^{11}B\{^{1}H\}$ NMR spectrum (128 MHz, DMF) of reaction when $[K(18c6)]_3$[**6**] and HBpin (1 eq.) are flash frozen and then N_2O added.

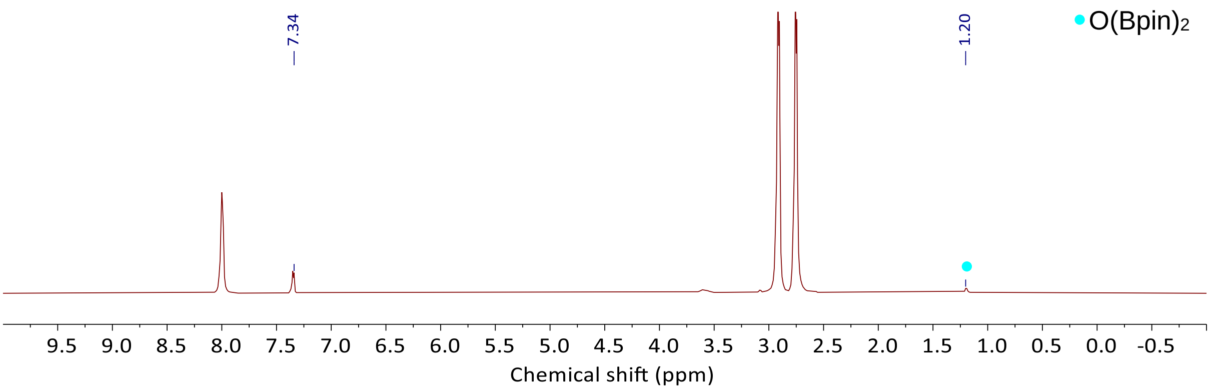

Figure S42. ^{1}H NMR spectrum (128 MHz, DMF) of reaction when [K(18c6)]$_3$[**6**] and HBpin (1 eq.) are flash frozen, then N_2O added, and then additional HBpin added.

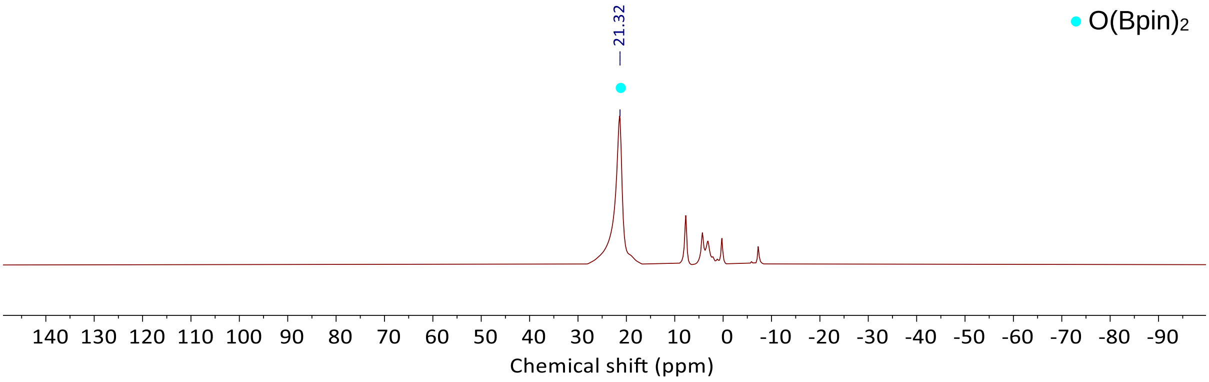

Figure S43. $^{11}B\{^{1}H\}$ NMR spectrum (128 MHz, DMF) of reaction when [K(18c6)]$_3$[**6**] and HBpin (1 eq.) are flash frozen, then N_2O added, and then additional HBpin added.

3.5. Addition HBpin to a catalytic amount of [K(18c6)]$_3$[5], followed by N_2O addition

To a J Young NMR tube, a solution of [K(18c6)]$_3$[**5**] (0.7 µmol, 0.01 eq.) in DMF (0.5 mL) and HBpin (10.7 µL, 0.074 mmol, 1.0 eq.) was added. C_6H_6 (10 µL, 0.11 mmol) was added as an internal standard. The reaction mixture was allowed to react for 16 h after which it was frozen at –78 °C. While frozen, the headspace in the NMR tube was evacuated. The headspace was refilled with N_2O (1 atm) and the NMR tube was removed from the cooling bath and the solution was allowed to thaw. The reaction was monitored by ^{1}H, ^{11}B and $^{11}B\{^{1}H\}$ NMR spectroscopy. NMR conv. was determined by integration of the crude ^{1}H NMR spectrum using the C_6H_6 as internal standard (^{1}H δ = 7.35 ppm).

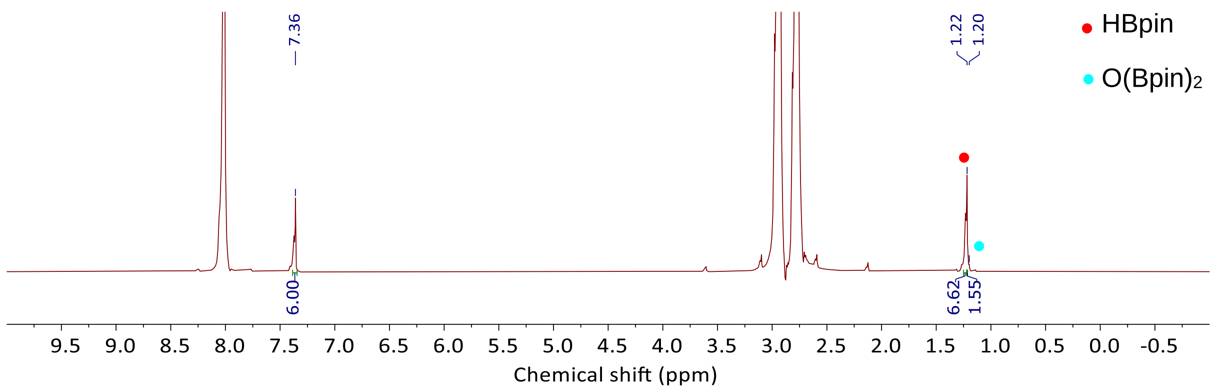

Figure S44. ^{1}H NMR spectrum (400 MHz, DMF) of reaction when $[K(18c6)]_3$[**5**] and HBpin (100 eq.) are reacted and then N_2O added.

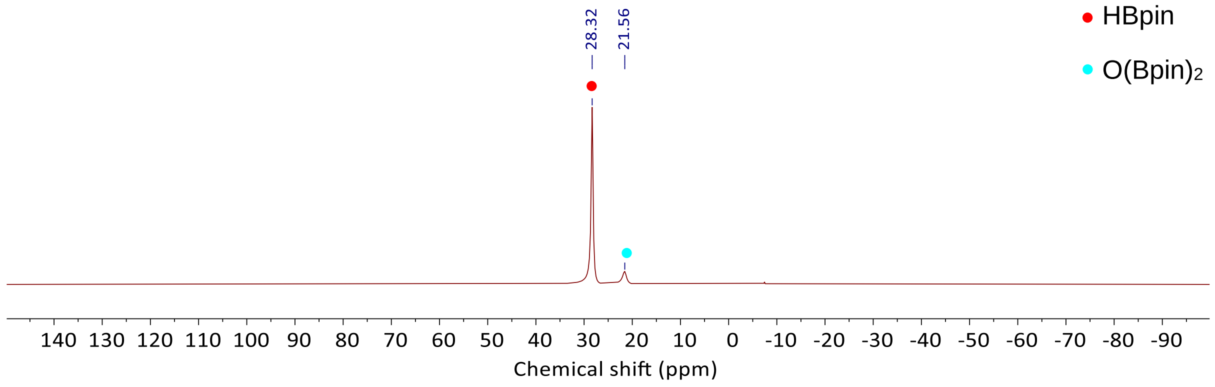

Figure S45. ^{11}B NMR spectrum (400 MHz, DMF) of reaction when $[K(18c6)]_3$[**5**] and HBpin (100 eq.) are reacted and then N_2O added.

3.6. Addition HBpin to catalytic amount $[K(18c6)]_3$[6], followed by N_2O

To a J Young NMR tube, a solution of $[K(18c6)]_3$[**6**] (0.7 μmol, 0.01 eq.) in DMF (0.5 mL) and HBpin (10.7 μL, 0.074 mmol, 1.0 eq.) was added. C_6H_6 (10 μL, 0.11 mmol) was added as an internal standard. The reaction mixture was allowed to react for 16 h after which it was frozen at –78 °C. While frozen, the headspace in the NMR tube was evacuated. The headspace was refilled with N_2O (1 atm) and the NMR tube was removed from the cooling bath and the solution was allowed to thaw. The reaction was monitored by ^{1}H, ^{11}B and $^{11}B\{^{1}H\}$ NMR spectroscopy. NMR conv. was determined by integration of the crude ^{1}H NMR spectrum using the C_6H_6 as internal standard (^{1}H δ = 7.35 ppm).

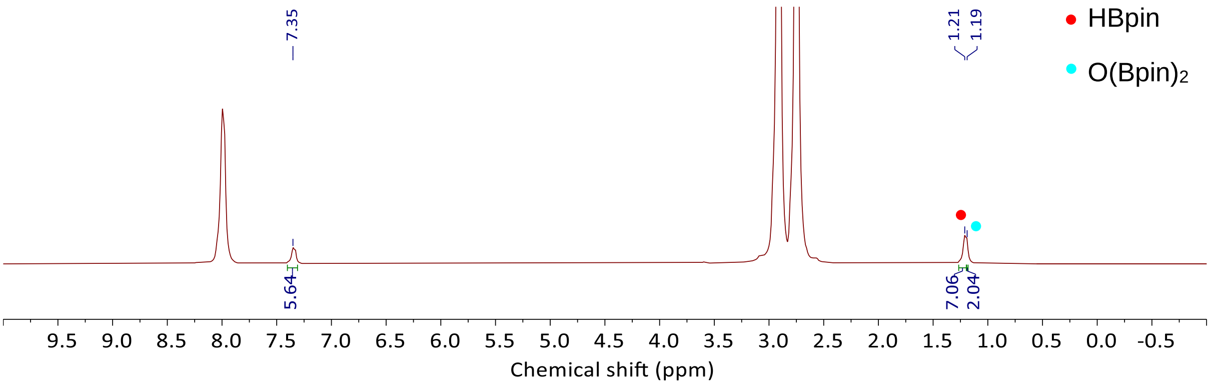

Figure S46. ^{1}H NMR spectrum (400 MHz, DMF) of reaction when $[K(18c6)]_3$[**6**] and HBpin (100 eq.) are reacted and then N_2O added.

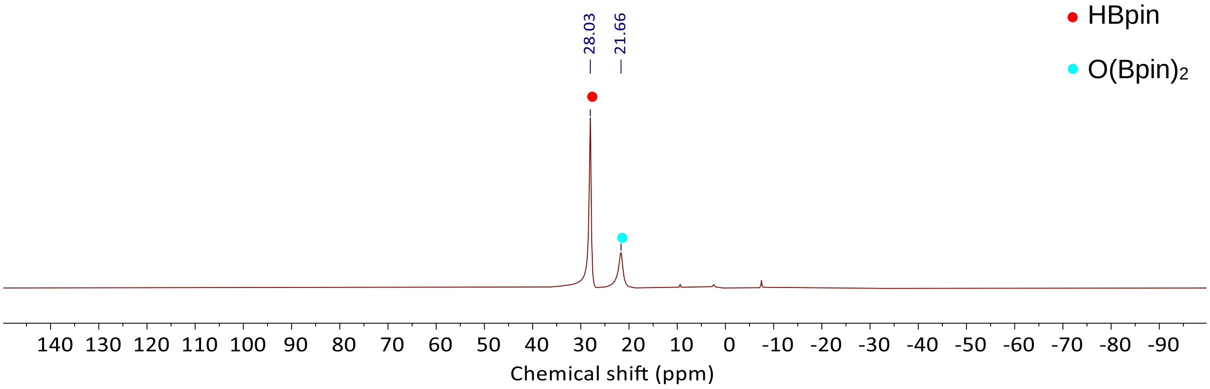

Figure S47. ^{11}B NMR spectrum (400 MHz, DMF) of reaction when $[K(18c6)]_3$[**6**] and HBpin (100 eq.) are reacted and then N_2O added.

3.7. Addition of N_2O to $[K(18c6)]_3$[5]

To a J Young NMR tube, a solution of $[K(18c6)]_3$[**5**] (20.0 mg, 0.018 mmol) in DMF (0.5 mL) was added. The reaction mixture was immediately frozen at –78 °C. While frozen, the headspace in the NMR tube was evacuated. The headspace was refilled with N_2O (1 atm) and the NMR tube was removed from the cooling bath and the solution was allowed to thaw for 30 min. An immediate colour change was observed from dark red to yellow. The reaction mixture was monitored by ^{31}P NMR spectroscopy.

Mass spectrometry (ESI): P_7O_3+H_2 ($[P_7O_3+H_2]^-$): cald. 266.8173; found: 266.8219.

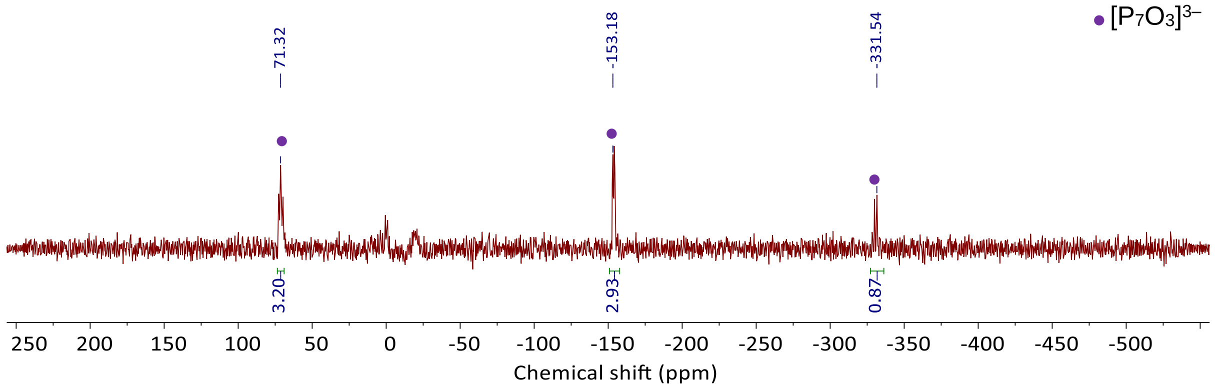

Figure S48. ^{31}P NMR spectrum (202 MHz, DMF) of the addition of N_2O to $[K(18c6)]_3$[**5**]. This data is consistent with literature reported $[P_7O_3]^{3-}$.[21]

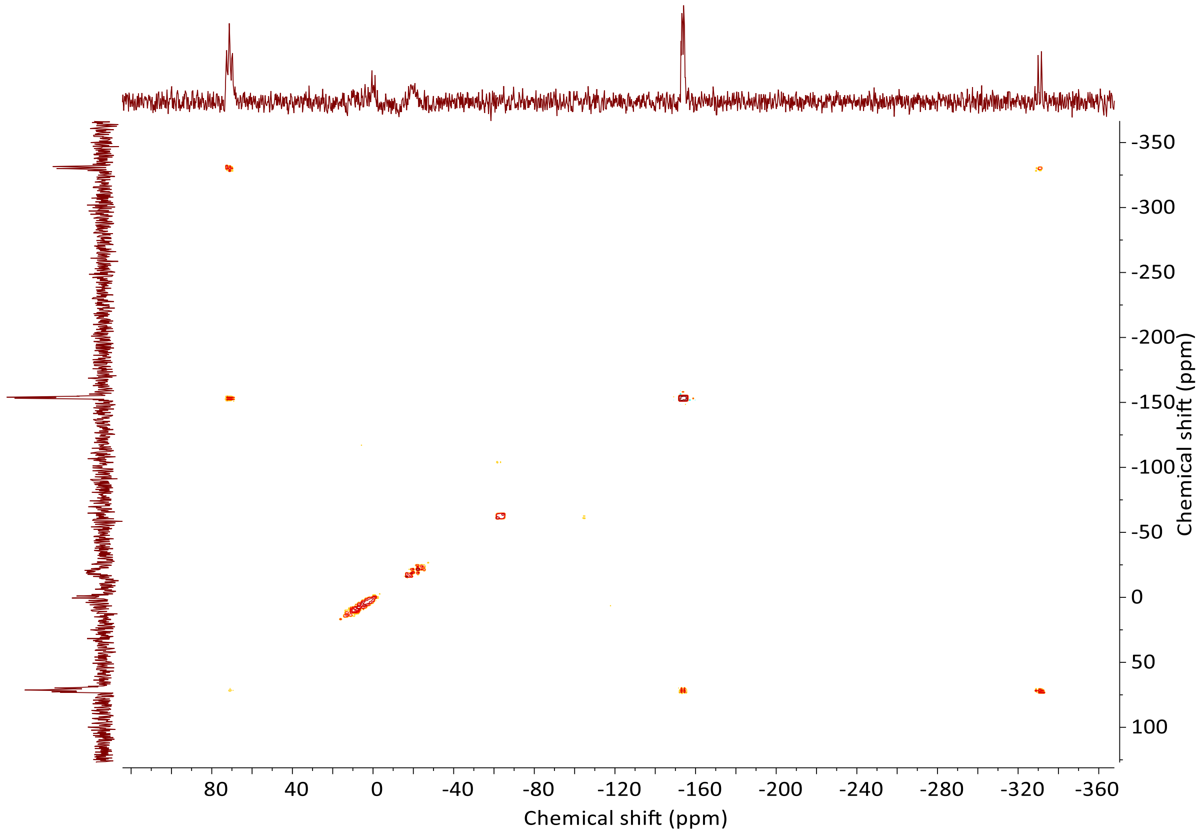

Figure S49. ^{31}P COSY NMR spectrum (202 MHz, DMF) of the addition of N_2O to $[K(18c6)]_3$[**5**].

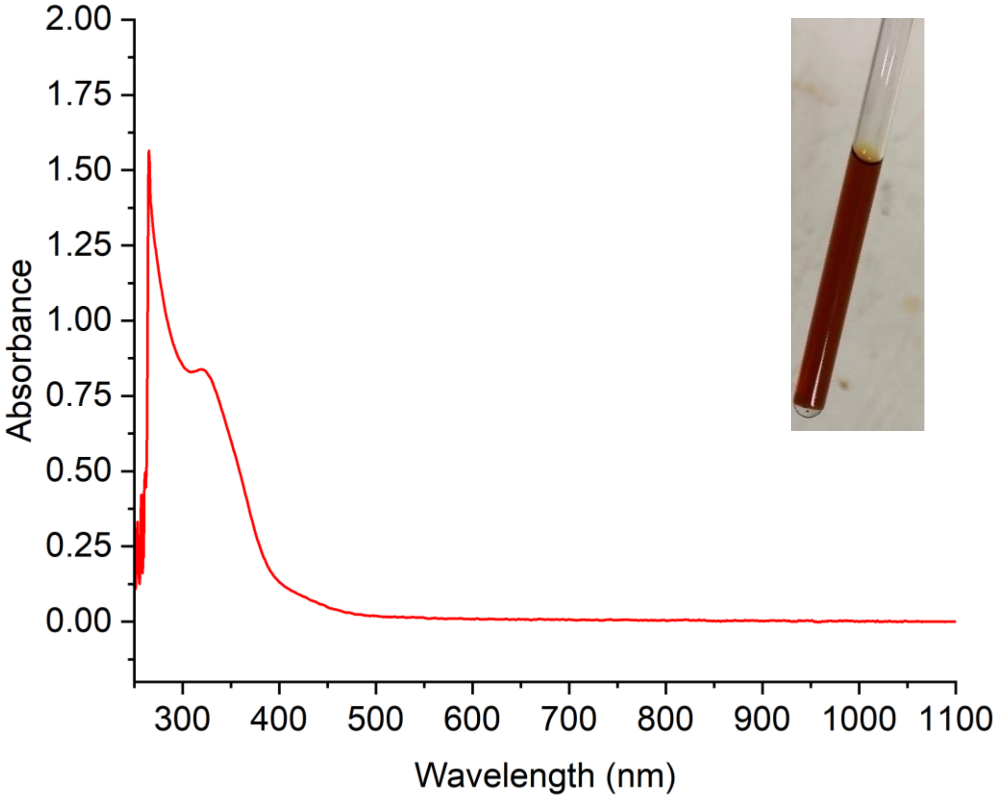

Figure S50. UV-Vis spectrum (0.06 mM, DMF) of $[K(18c6)]_3$[**5**].

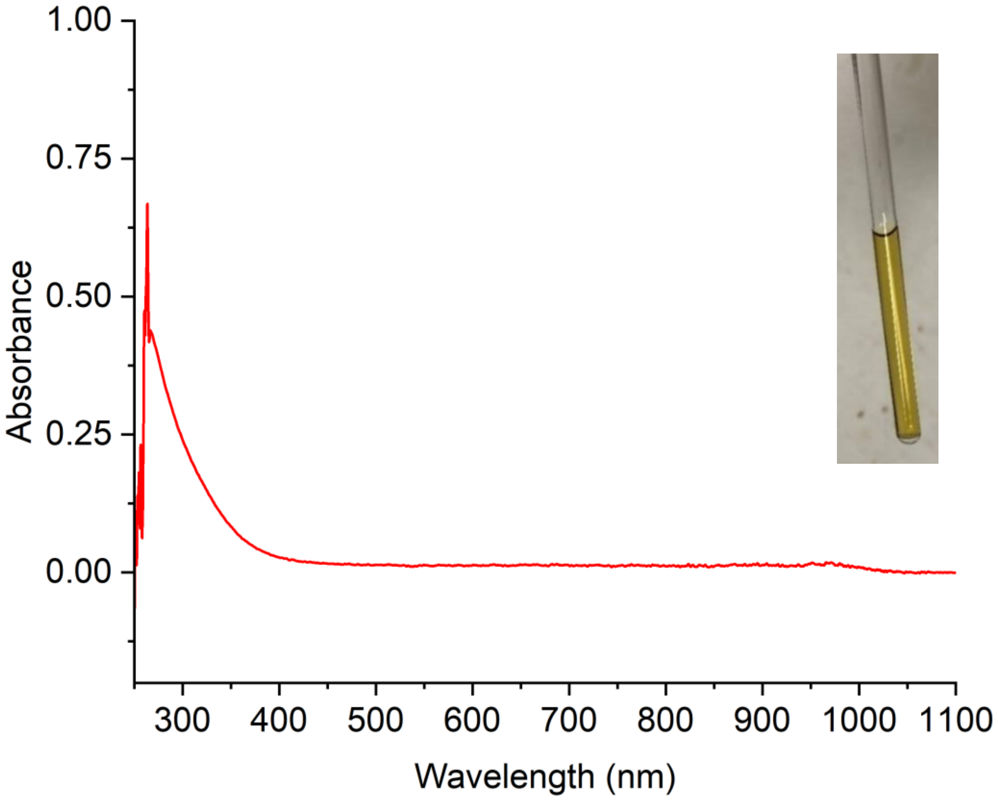

Figure S51. UV-Vis spectrum (0.06 mM, DMF) of the addition of N_2O to $[K(18c6)]_3$[**5**].

3.8. Addition of HBpin to *in situ* generated $[P_7O_3]^{3-}$

3.8.1. Addition of excess HBpin

A solution of $[P_7O_3]^{3-}$ was formed *in situ* as described above in section 3.7. on 0.02 mmol scale. This solution was degassed and HBpin (29 µL, 0.2 mmol, 100 eq.) was added and allowed to react for 30 min. The reaction mixture was monitored by ^{1}H and ^{11}B NMR spectroscopy.

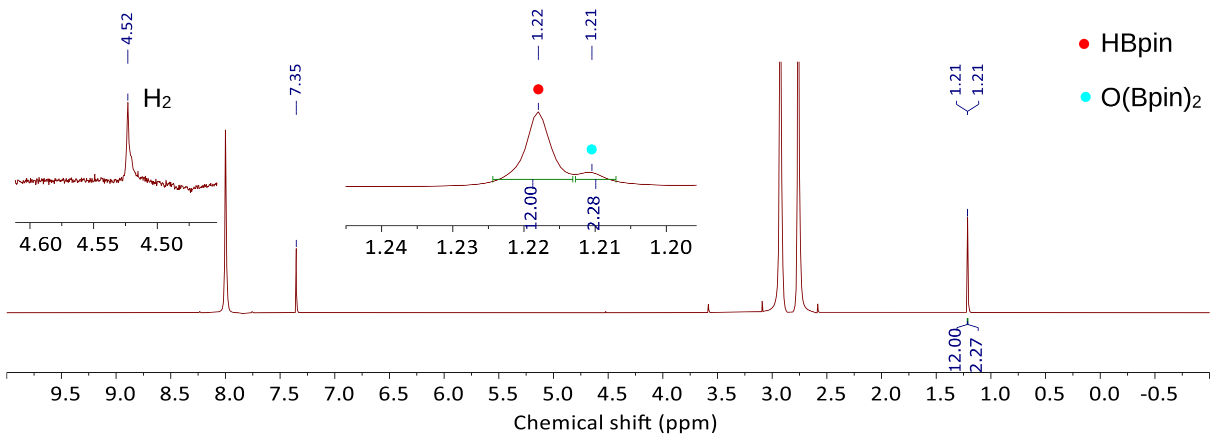

Figure S52. ^{1}H NMR spectrum (400 MHz, DMF) of the addition of 100 eq. HBpin to $[P_7O_3]^{3-}$.

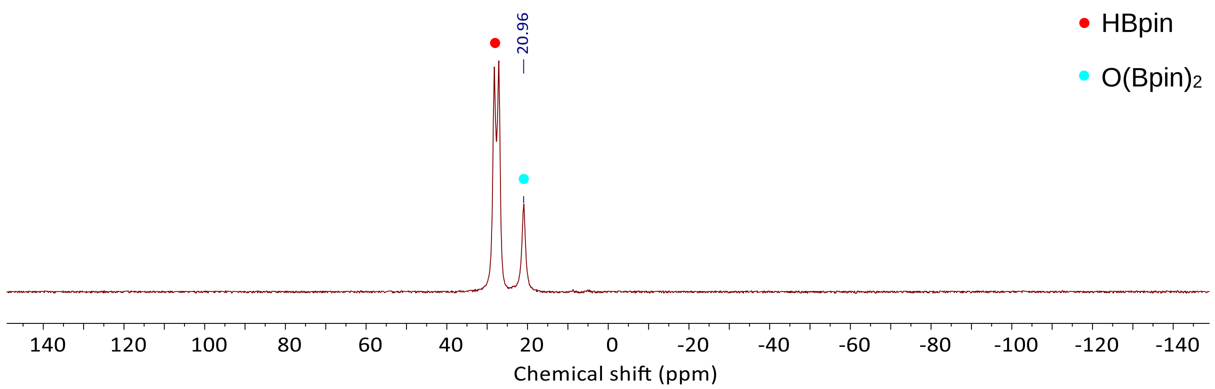

Figure S53. ^{11}B NMR spectrum (128 MHz, DMF) of the addition of 100 eq. HBpin to $[P_7O_3]^{3-}$.

3.8.2. Addition of increasing controlled amounts of HBpin to *in situ* generated $[P_7O_3]^{3-}$ and then subsequent N_2O reactivity

A solution of $[P_7O_3]^{3-}$ was formed *in situ* as described above in section 3.7. on 0.018 mmol scale. This solution was degassed and HBpin (1.3 µL, 0.009 mmol, 0.5 eq.) was added. Before the HBpin reacted, the solution was flash frozen at –78 °C and subsequently allowed to slowly thaw, and analyzed by ^{31}P NMR spectroscopy. This was repeated 8 times until no $[P_7O_3]^{3-}$ was observed in the ^{31}P NMR spectrum. The reaction revealed reformation of $[P_7]^{3-}$, along with other polyphosphides (Figure S54). This reaction mixture was re-pressurized with N_2O and again studied by ^{31}P NMR spectroscopy, which revealed reformation of $[P_7O_3]^{3-}$, along other polyphosphides (Figure S55).

Mass spectrometry (ESI): $[P_5]^-$: cald. 154.8694; found: 154.8692; $[P_{21}]^{3-}$: cald. 216.8169; found: 216.8190; $[H_2P_7]^-$: cald.: 218.8320; found: 218.8386.

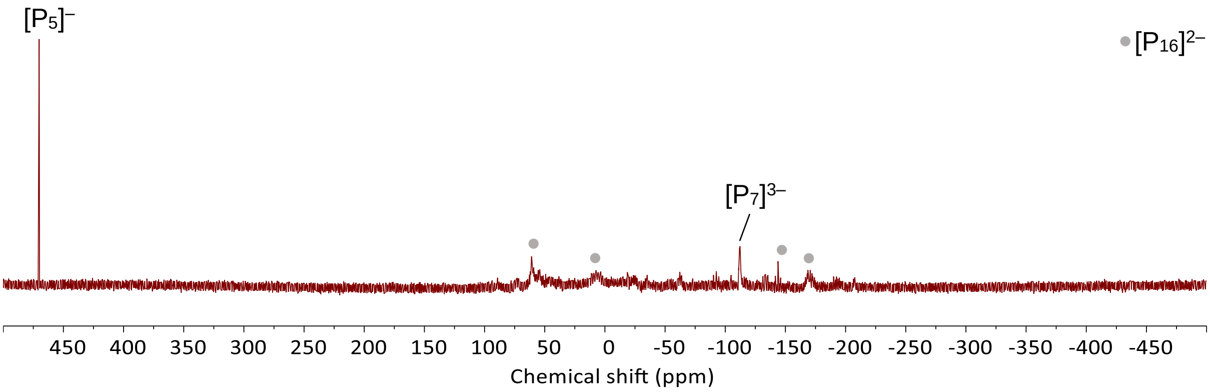

Figure S54. ^{31}P NMR spectrum (400 MHz, DMF) of the incremental addition of 4 eq. HBpin to $[P_7O_3]^{3-}$.

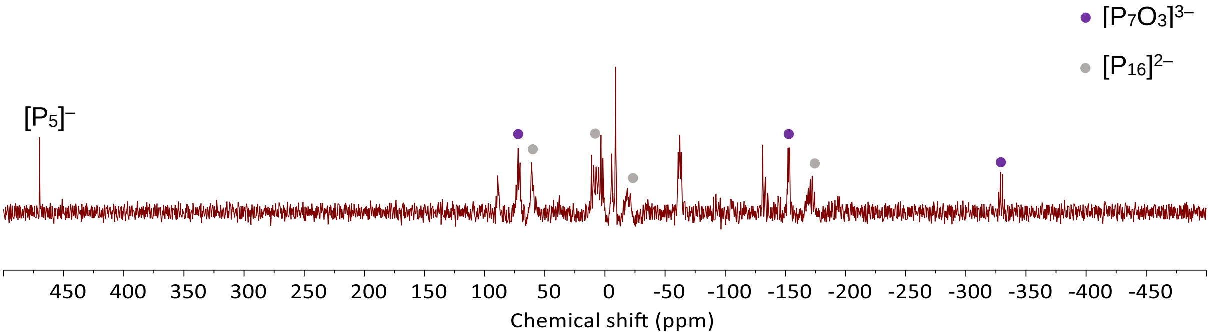

Figure S55. ^{31}P NMR spectrum (162 MHz, DMF) of the incremental addition of 4 eq. HBpin to $[P_7O_3]^{3-}$, followed by addition of N_2O.

3.9. Addition of N_2O to K_3As_7 + crypt, synthesis of [K(crypt)]$_2$[9]

To a J Young NMR tube, a solution of K_3As_7 (30.0 mg, 0.047 mmol, 1 eq.) and crypt (52 mg, 0.138 mmol, 3 eq.) in DMF (0.5 mL) was added. The reaction mixture was immediately frozen at –78 °C. While frozen, the headspace in the NMR tube was evacuated. The headspace was refilled with N_2O (1 atm) and the NMR tube was removed from the cooling bath and the solution was allowed to thaw. An immediate colour change was observed from dark red to colourless. Some small amounts of black precipitate formed during the reaction and the reaction mixture was filtered. Slow

diffusion of diethyl ether into the filtrate at –30 °C resulted in clear large needle-type crystals. The supernatant was removed from the crystals. The crystals were further washed with diethyl ether and subsequently dried under reduced pressure yielding a white powder.

Yield: 28.2 mg, 53%.

Elemental analysis for $C_{39}H_{79}As_4K_2N_5O_{20}$: calcd.: C 35.60, H 6.05, N 5.32; found C 35.38, H 5.83, N 5.22

Mass spectrometry (ESI): $[As_4O_7+H]^-$ ([**9**+H]$^-$): cald. 412.6586; found: 412.6592.

Infrared (ATR): ν: 884 cm^{-1} (external As–O stretching), 780 cm^{-1} (internal As–O symmetric stretching), 684 cm^{-1} (internal As–O asymmetric stretching).

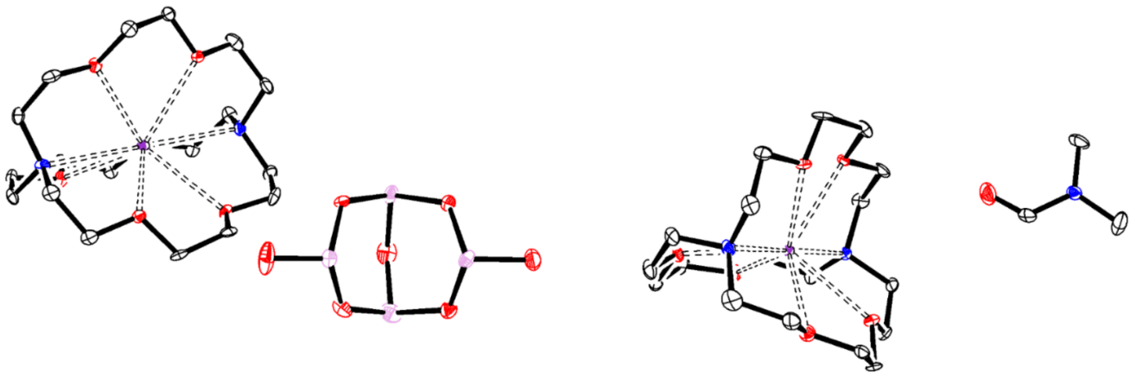

Figure S56. Molecular structure of $[K(crypt)]_2$[**9**]·DMF. Anisotropic displacement ellipsoids pictured at 50% probability. Hydrogen atoms omitted for clarity. Positional disorder omitted for clarity and major component (*ca.* 80% occupancy) shown. Carbon: black; arsenic: plum; oxygen: red; potassium: violet; nitrogen: blue. CCDC code: 2434061.

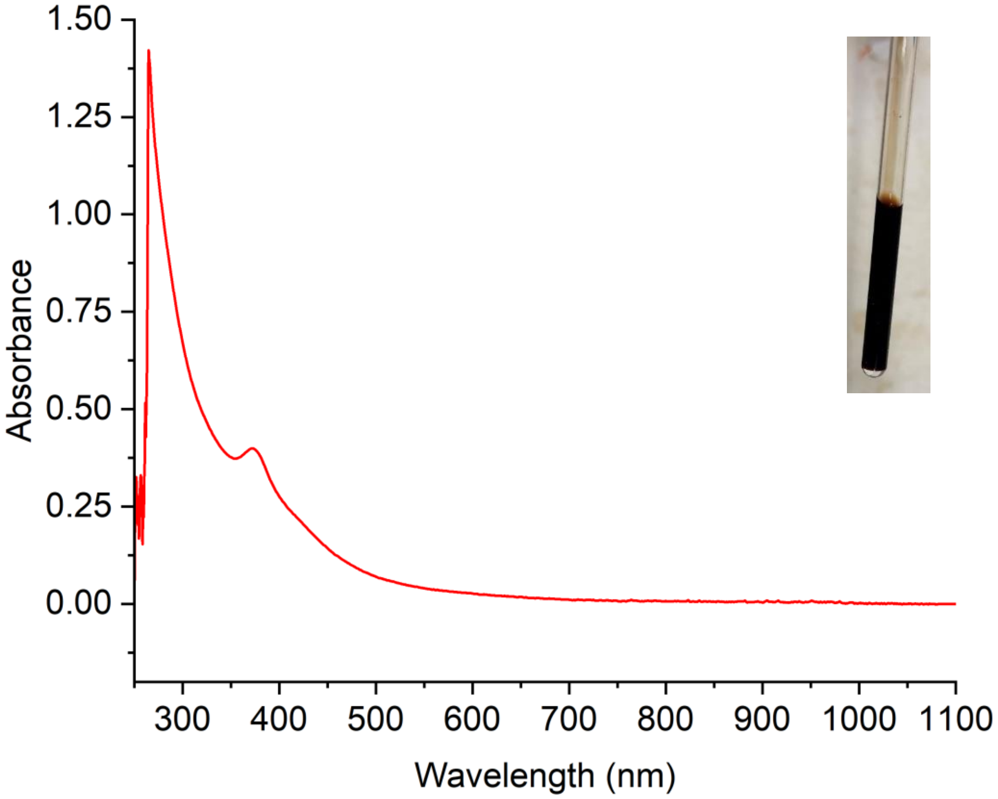

Figure S57. UV-Vis spectrum (0.06 mM, DMF) of K_3As_7 + 3 eq. crypt.

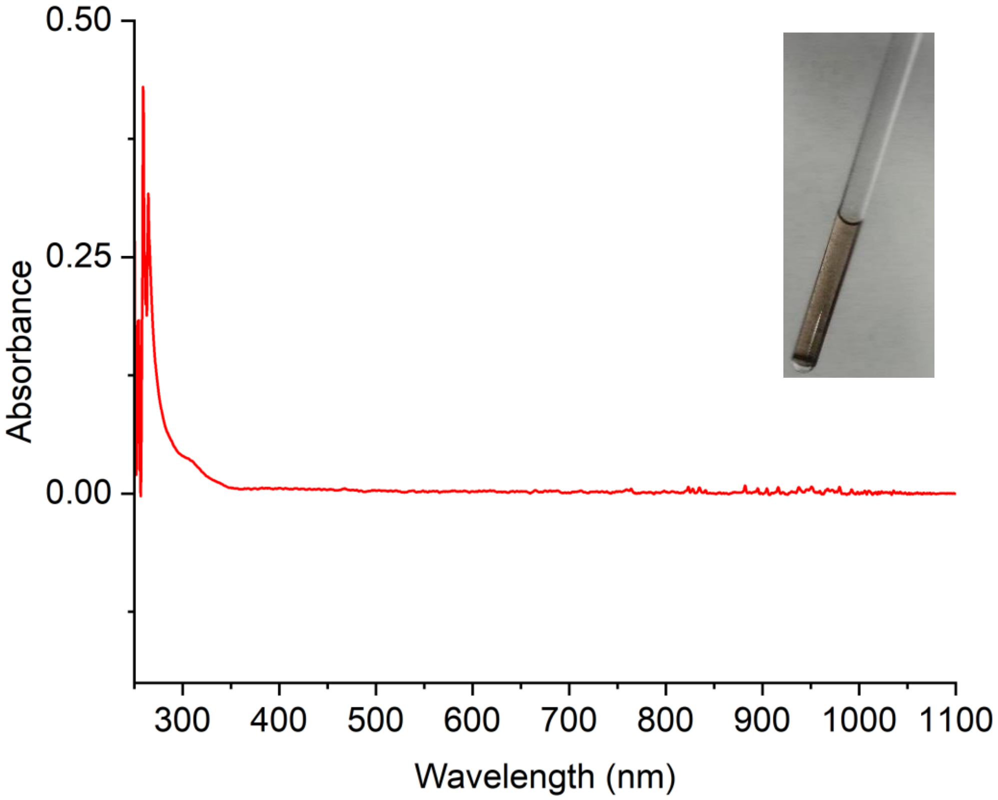

Figure S58. UV-Vis spectrum (0.06 mM, DMF) of [K(crypt)]$_2$[**9**].

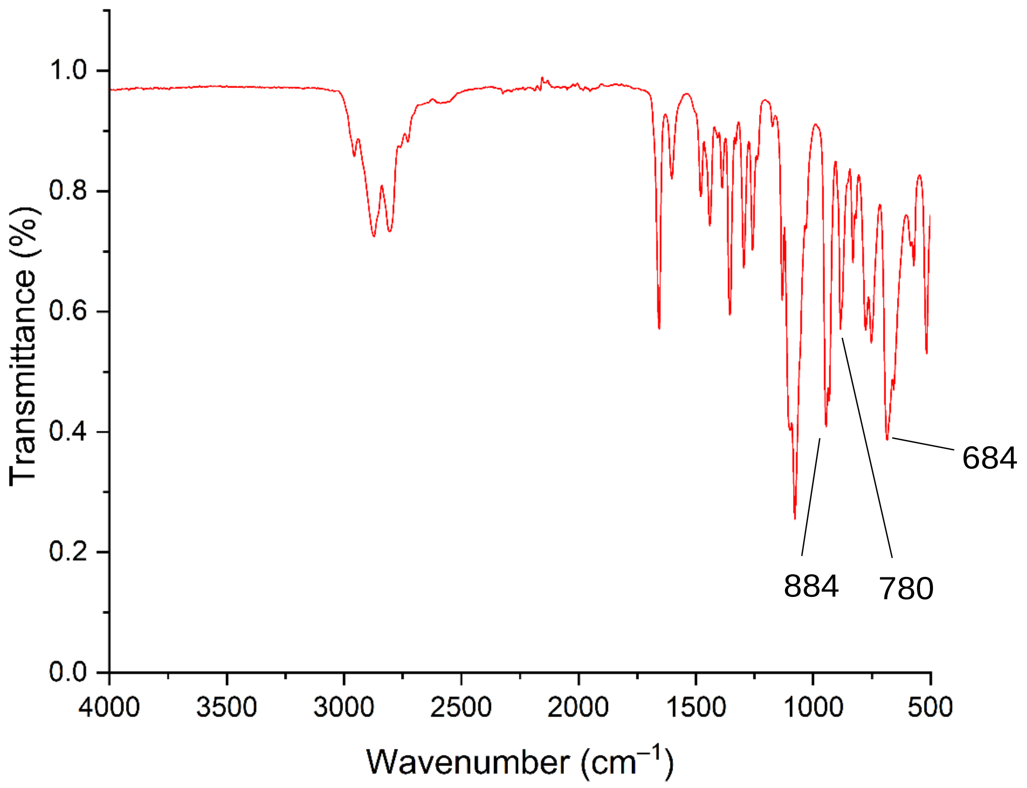

Figure S59. IR spectrum of [K(crypt)]$_2$[**9**].

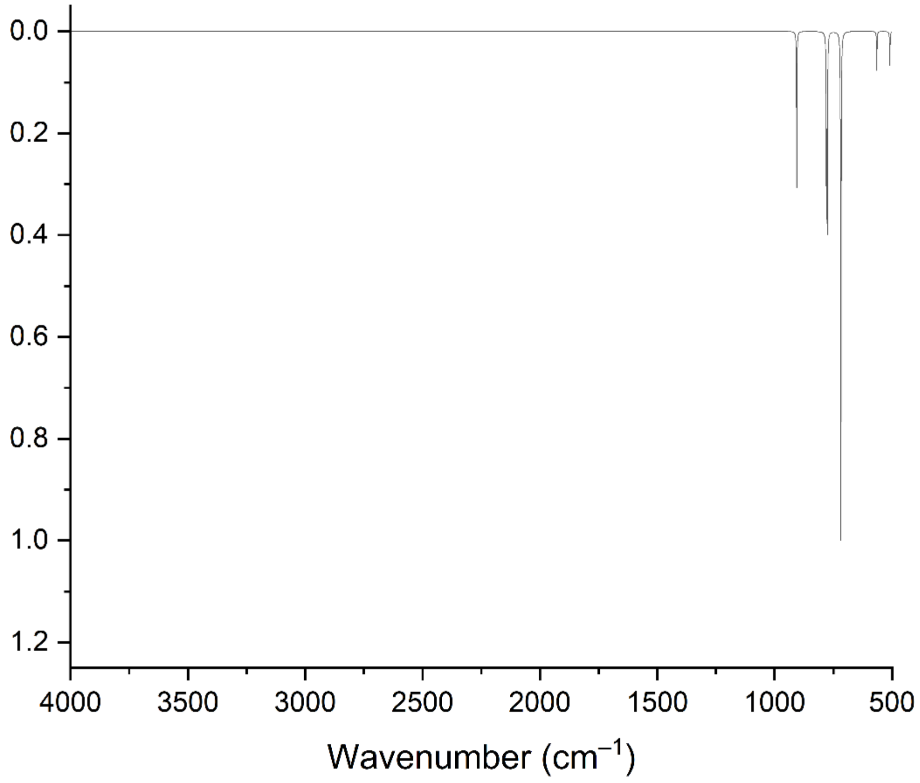

Figure S60. Calculated IR spectrum of [**9**]$^{2-}$.

3.10. Addition of HBpin to [K(crypt)]$_2$[9].

To a J Young NMR tube, a solution of [K(crypt)]$_2$[**9**] (4.8 mg, 0.004 mmol, 1 eq.) in DMF (0.5 mL) and HBpin (58 µL, 0.4 mmol, 100 eq.) was added and allowed to react for 30 min. The reaction was monitored by ^{1}H and ^{11}B NMR spectroscopy, which confirmed the formation of **7** in the reaction mixture. The reaction mixture was analyzed by HRMS techniques, see below. The reaction mixture was degassed and the headspace was refilled the N_2O (1 atm), [K(crypt)]$_2$[**9**] could again be observed by HRMS techniques.

Mass spectrometry before addition of N_2O (ESI): [As$_5$]$^-$: cald. 374.6080; found: 374.6077; [As$_{16}$]$^{2-}$: cald. 599.3733; found: 599.3725; [As$_{21}$]$^{3-}$: cald. 524.4517; found: 524.4507.

Mass spectrometry after addition of N_2O (ESI): $[HAs_4O_7]^-$: cald. 412.6586; found: 412.6548.

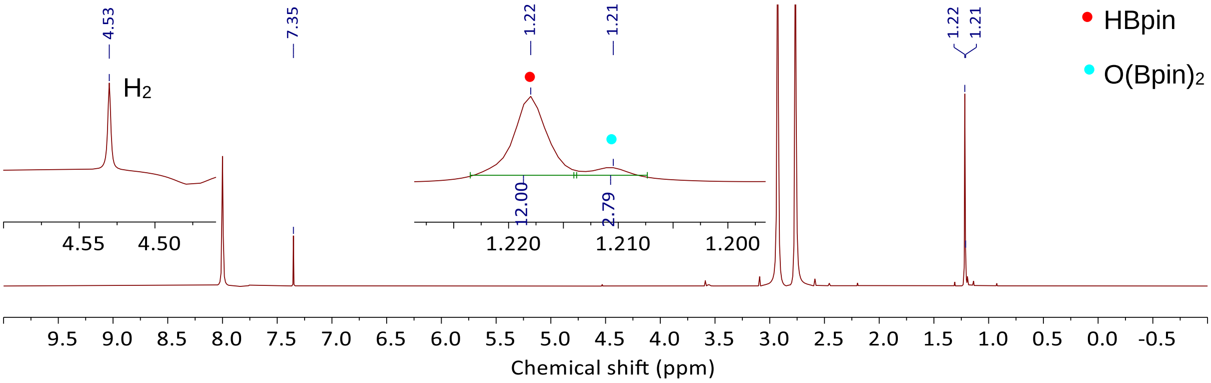

Figure S61. ^{1}H NMR spectrum (400 MHz, DMF) of the addition of 100 eq. HBpin to $[K(crypt)]_2$[**9**].

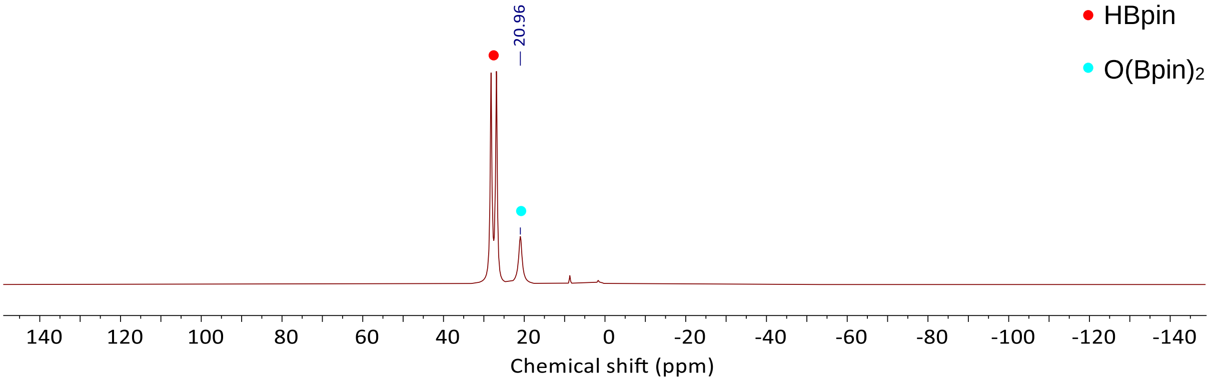

Figure S62. ^{11}B NMR spectrum (128 MHz, DMF) of the addition of 100 eq. HBpin to $[K(crypt)]_2$[**9**].

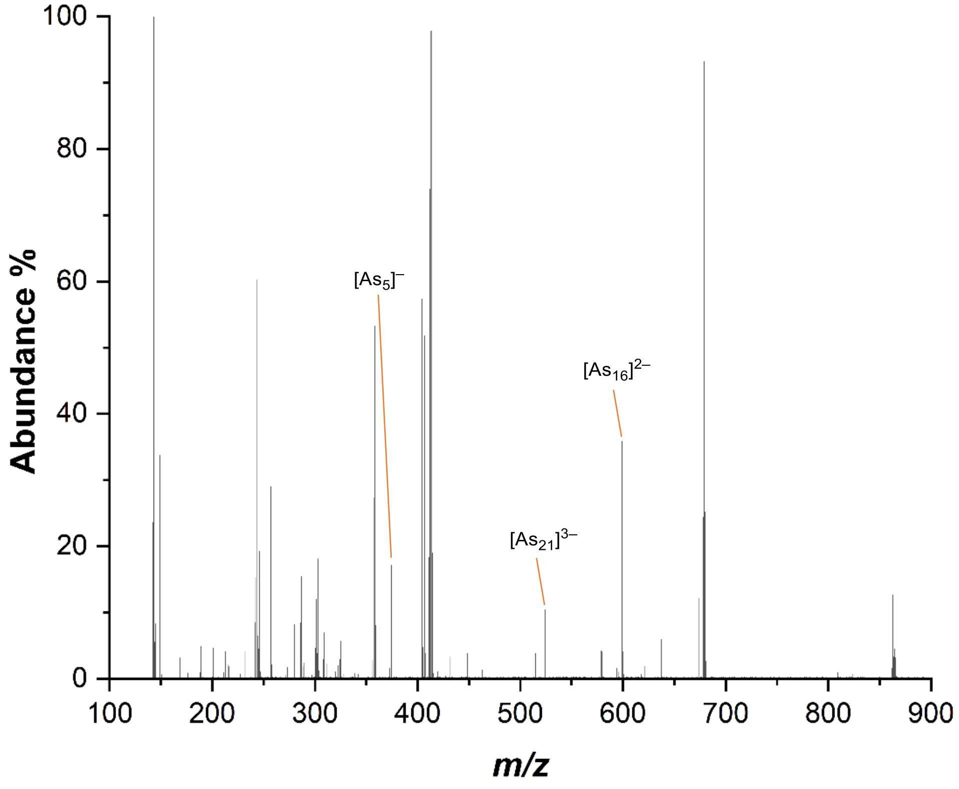

Figure S63. Mass spectrometry (ESI) results after the addition of 100 eq. HBpin to $[K(crypt)]_2$[**9**].

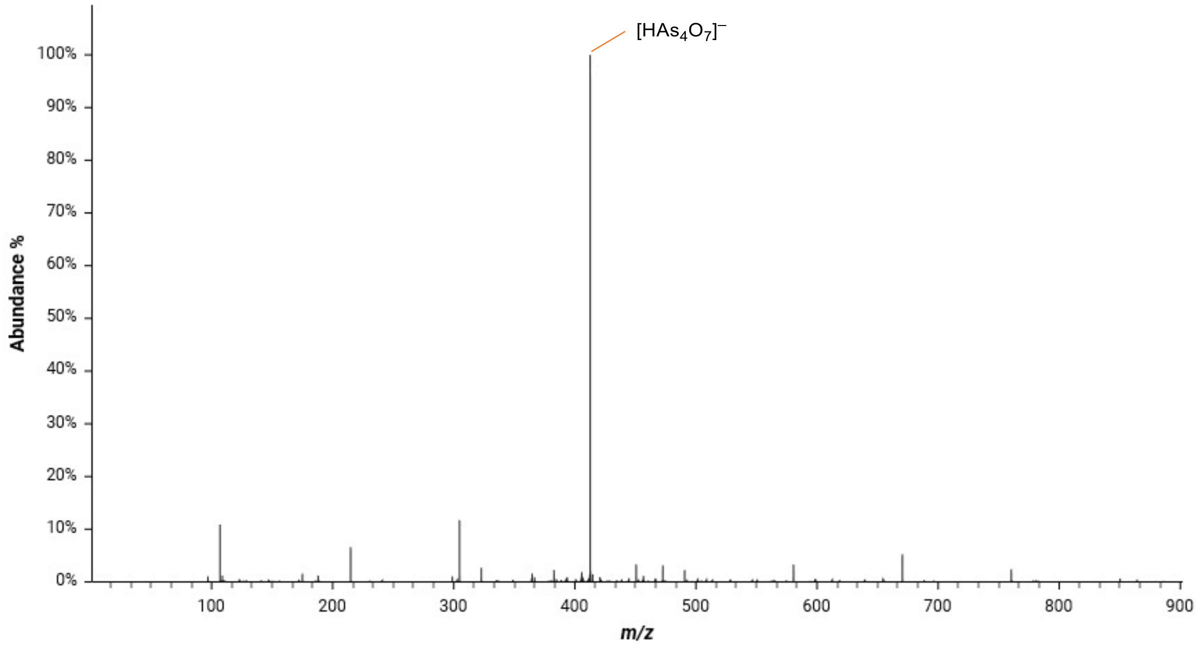

Figure S64. Mass spectrometry (ESI) results after the addition of 100 eq. HBpin to $[K(crypt)]_2$[**9**] and subsequent addition of N_2O.

3.11. N_2O hydroboration catalyzed by [K(18c6)]$_2$[P_{16}]

To a J Young NMR tube, a solution of [K(18c6)]$_2$[P_{16}] (0.7 μmol, 0.01 eq.) in DMF (0.5 mL) and HBpin (10.7 μL, 0.074 mmol, 1.0 eq.) was added. C_6H_6 (10 μL, 0.11 mmol) was added as an internal standard. The reaction mixture was immediately frozen at –78 °C. While frozen, the headspace in the NMR tube was evacuated. The headspace was refilled with N_2O (1 atm) and the NMR tube was removed from the cooling bath and the solution was allowed to thaw. The reaction was monitored by ^{1}H, ^{11}B and $^{11}B\{^{1}H\}$ NMR spectroscopy. NMR conv. was determined by integration of the crude ^{1}H NMR spectrum using the C_6H_6 as internal standard (^{1}H δ = 7.35 ppm). NMR conv. observed: 74% **7** and 25% **8**.

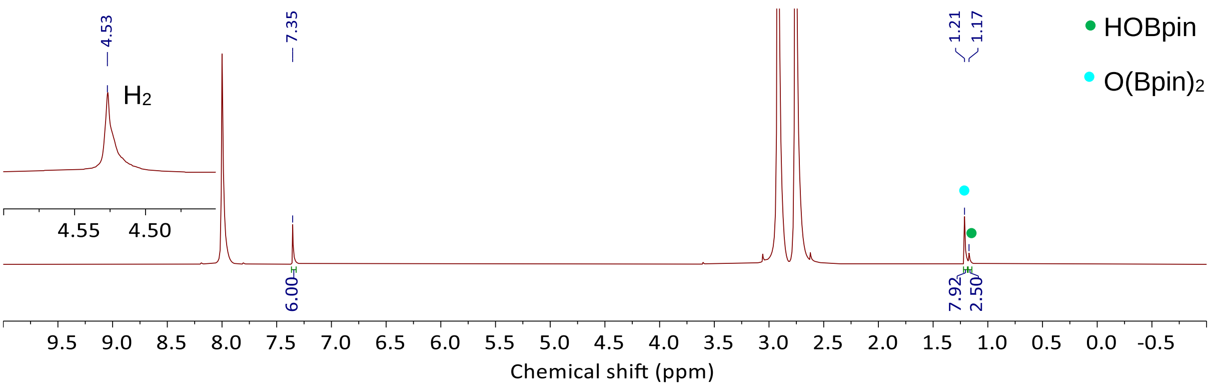

Figure S65. ^{1}H NMR spectrum (128 MHz, DMF) of the hydroboration of N_2O catalyzed by [K(18c6)]$_2$[P_{16}] using HBpin.

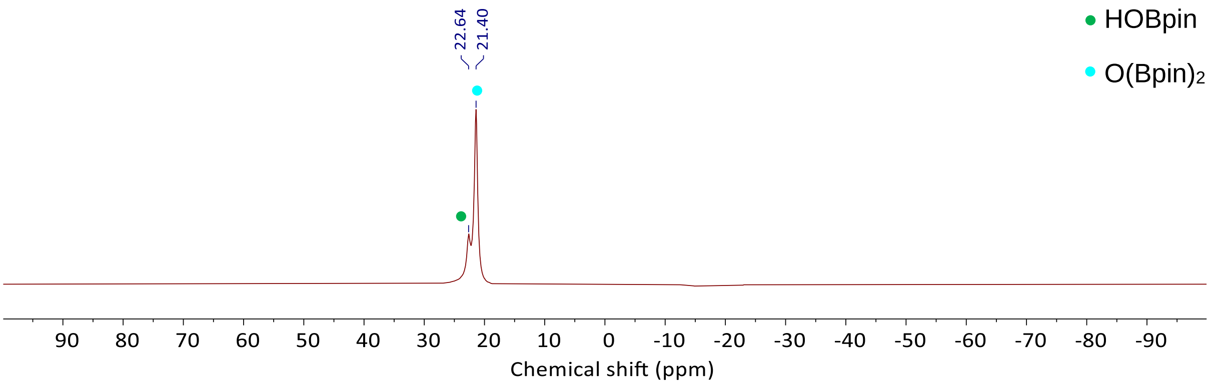

Figure S66. ^{11}B NMR spectrum (128 MHz, DMF) of the hydroboration of N_2O catalyzed by [K(18c6)]$_2$[P_{16}] using HBpin in DMF.

4. N_2O Reduction with Pnictogen Oxide Clusters

4.1. Pnictogen oxides mediated hydroboration of N_2O

To a J Young NMR tube, catalyst (specific loading given in Table S6) in DMF (0.5 mL) and HBpin (10.7 µL, 0.074 mmol, 1.0 eq.) were added. C_6H_6 (10 µL, 0.11 mmol) was added as an internal standard. The reaction mixture was immediately frozen at –78 °C. While frozen, the headspace in the NMR tube was evacuated. The headspace was refilled with N_2O (1 atm) and the NMR tube was removed from the cooling bath and the solution was allowed to thaw. The reaction was monitored by ^{1}H, ^{11}B and ^{11}B{^{1}H} NMR spectroscopy. NMR conv. was determined by integration of the crude ^{1}H NMR spectrum using the C_6H_6 as internal standard (^{1}H δ = 7.35 ppm). Below are given the NMR spectra of entry 1, Table S6 as representative examples.

Table S6. Catalytic N_2O Reduction with Pnictogen Oxide Clusters

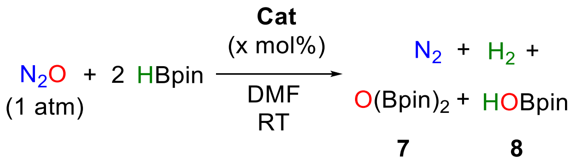

Entry	Cat	Loading (x mol%)	Time (h)	**7** Conv. (%)[a]	**8** Conv. (%)[a]	Total Conv. (%)[a]
1	[K(crypt)]$_2$[**9**]	1	1	70	29	>99
2		0.5	2	79	20	>99
3		0.1	24	98	0	98
4	P_4O_{10}	10	24	5	0	5
5	As_4O_6	10	24	99	0	>99
6	Sb_4O_6	10	24	90	0	90

[a] NMR conv. was determined by integration of the crude ^{1}H NMR spectrum using C_6H_6 as internal standard.

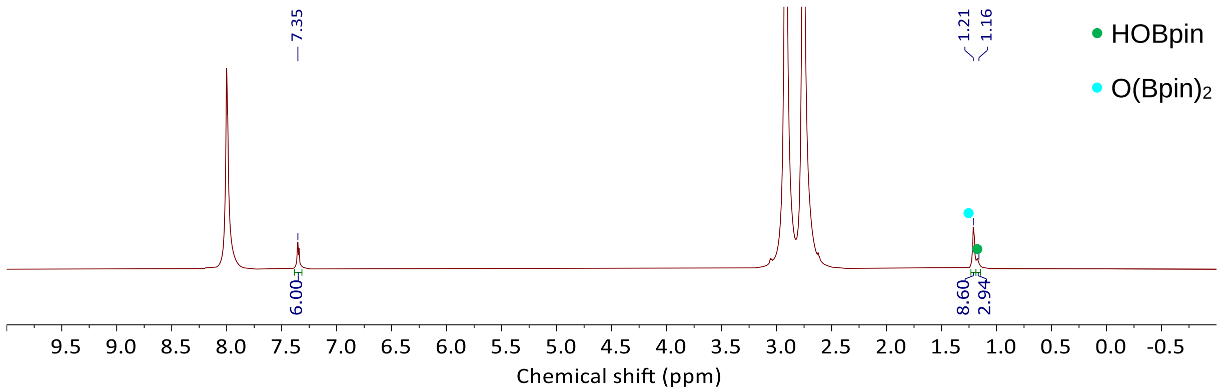

Figure S67. ^{1}H NMR spectrum (400 MHz, DMF) of the hydroboration of N_2O catalyzed by $[K(crypt)]_2$[**9**] using HBpin in DMF.

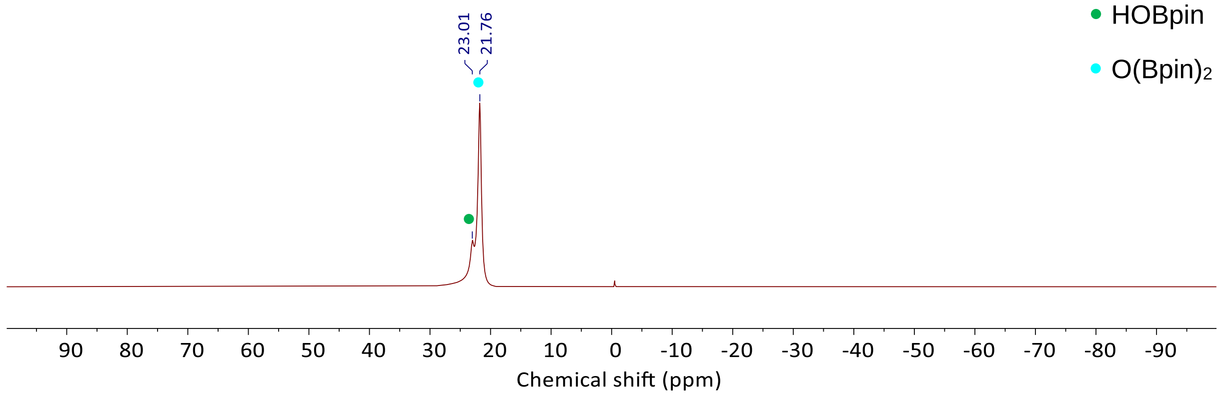

Figure S68. ^{11}B NMR spectrum (128 MHz, DMF) of the hydroboration of N_2O catalyzed by $[K(crypt)]_2$[**9**] using HBpin in DMF.

4.2. N_2O reduction under areobic conditions

The observation that $[K(crypt)]_2$[**9**] could be dissolved in water and without any indication of decomposition, prompted the studied towards N_2O hydroboration using areobic conditions.

In a fumehood not under Schlenk conditions, to a J Young NMR tube $[K(crypt)]_2$[**9**] (0.7 μmol, 0.01 eq.) in untreated 'wet' DMF (0.5 mL) and HBpin (10.7 μL, 0.074 mmol, 1.0 eq.) were added. C_6H_6 (10 μL, 0.11 mmol) was added as an internal standard. The reaction mixture was immediately frozen at –78 °C. While frozen, the headspace in the NMR tube was evacuated. The headspace was refilled with N_2O (1 atm) and the NMR tube was removed from the cooling bath and the solution was allowed to thaw. The reaction was monitored by ^{1}H, ^{11}B and ^{11}B{^{1}H} NMR spectroscopy. NMR conv.

was determined by integration of the crude ^1H NMR spectrum using the C_6H_6 as internal standard (^1H δ = 7.35 ppm). In this reaction prior to the addition of N_2O, HBpin can react with residual water in the DMF solvent to give **7**. Thus, a background reaction under the same conditions without N_2O present was run, and confirmed a small background conversion (8% **7**; Figure S71). which was subtracted from the total conversion.

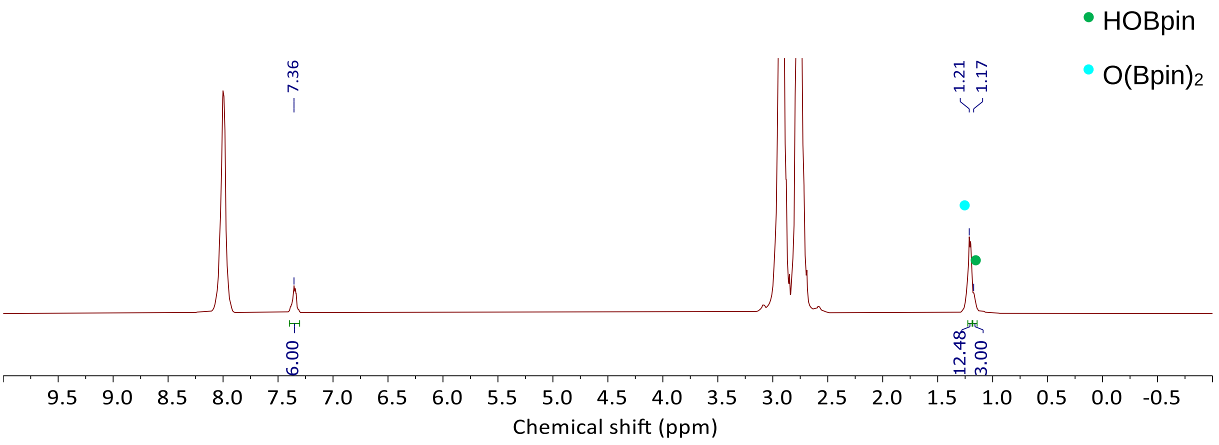

Figure S69. ^1H NMR spectrum (400 MHz, DMF) of the hydroboration of N_2O catalyzed by $[K(crypt)]_2[\mathbf{9}]$ using HBpin in 'wet' DMF.

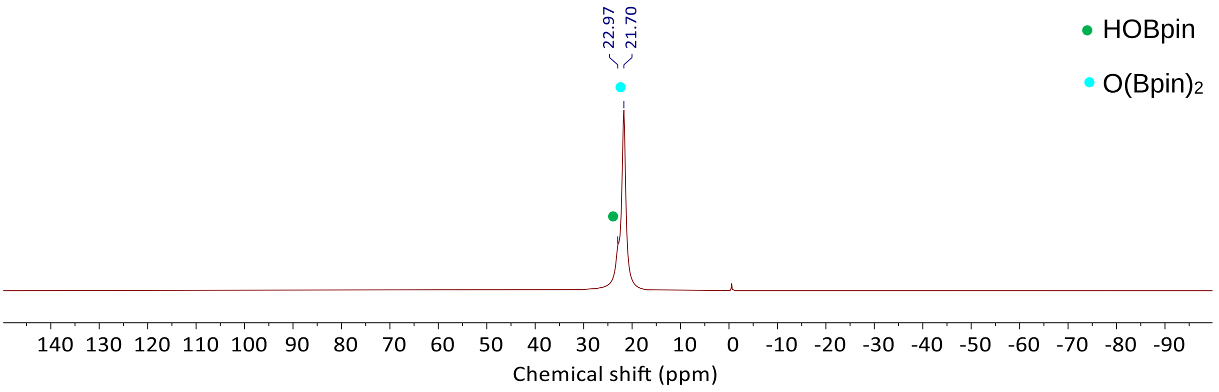

Figure S70. ^{11}B NMR spectrum (128 MHz, DMF) of the hydroboration of N_2O catalyzed by $[K(crypt)]_2[\mathbf{9}]$ using HBpin in 'wet' DMF.

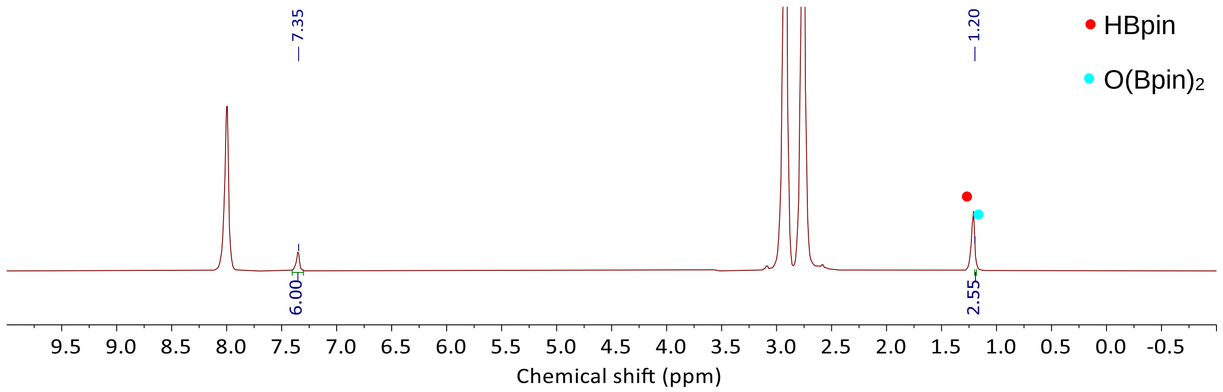

Figure S71. ^{1}H NMR spectrum (400 MHz, DMF) of the background hydroboration of 'wet' DMF catalyzed by $[K(crypt)]_2[\mathbf{9}]$ using HBpin.

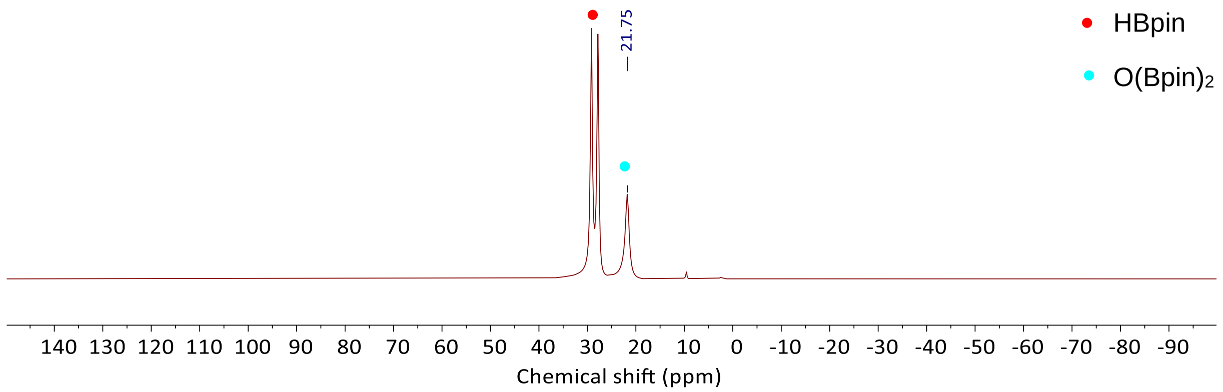

Figure S72. ^{11}B NMR spectrum (128 MHz, DMF) of the background hydroboration of 'wet' DMF catalyzed by $[K(crypt)]_2[\mathbf{9}]$ using HBpin.

5. Catalyst recycling and recovery studies

5.1. Catalyst recycling in the hydroboration of N_2O

To a J Young NMR tube, a solution of catalyst (0.7 μmol, 0.01 eq. for $[K(18c6)]_2$[**4**], $[K(18c6)]_3$[**5**], $[K(18c6)]_3$[**6**], $[K(crypt)]_2$[**9**] and $[K(18c6)]_2[P_{16}]$, and 7 μmol, 0.1 eq. for As_4O_6) in DMF (0.5 mL) and HBpin (10.7 μL, 0.074 mmol, 1.0 eq.) was added. C_6H_6 (10 μL, 0.11 mmol) was added as an internal standard. The reaction mixture was immediately frozen at –78 °C. While frozen, the headspace in the NMR tube was evacuated. The headspace was refilled with N_2O (1 atm) and the NMR tube was removed from the cooling bath and the solution was allowed to thaw. The reaction mixture was monitored by ^{1}H, ^{11}B and $^{11}B\{^{1}H\}$ NMR spectroscopy. NMR conv. was determined by integration of the crude ^{1}H NMR spectrum using the C_6H_6 as internal standard (^{1}H δ = 7.35 ppm). This was repeated another 9 times.

For $[K(18c6)]_2[P_{16}]$, only 12% conv. to **7** was observed after cycle 3, thus no further cycles were performed.

For $[K(crypt)]_2$[**9**] and As_4O_6, only 5% conv. and 4% conv. to **7**, respectively was observed after cycle 2, thus no further cycles were performed.

See Table S7 and S8 for full conversion.

Table S7. Catalyst ([K(18c6)]$_2$[**4**], [K(18c6)]$_3$[**5**], and [K(18c6)]$_3$[**6**]) recycling conversions.

	[K(18c6)]$_2$[**4**] (1 mol%)		[K(18c6)]$_3$[**5**] (1 mol%)		[K(18c6)]$_3$[**6**] (1 mol%)	
Cycle	NMR Conv. **7** (%)	NMR Conv. **8** (%)	NMR Conv. **7** (%)	NMR Conv. **8** (%)	NMR Conv. **7** (%)	NMR Conv. **8** (%)
1	94	0	84	15	74	25
2	92	0	93	6	79	20
3	88	0	97	0	98	0
4	99	0	95	0	98	0
5	98	0	91	0	92	0
6	97	0	94	0	95	0
7	98	0	91	0	96	0
8	80	0	87	0	90	0
9	78	0	83	0	86	0
10	75	0	80	0	78	0

NMR conv. was determined by the ratio reductant/product by integration of the crude.

Table S8. Catalyst ([K(18c6)]$_2$[P$_{16}$], [K(18c6)]$_2$[P$_{16}$], As_4O_6) recycling conversions.

	[K(18c6)]$_2$[P$_{16}$] (1 mol%)		[K(crypt)]$_2$[**9**] (1 mol%)		As_4O_6 (10 mol%)	
Cycle	NMR Conv. **7** (%)	NMR Conv. **8** (%)	NMR Conv. **7** (%)	NMR Conv. **8** (%)	NMR Conv. **7** (%)	NMR Conv. **8** (%)
1	87	12	99	0	99	0
2	79	0	5	0	4	0
3	12	0				

NMR conv. was determined by the ratio reductant/product by integration of the crude

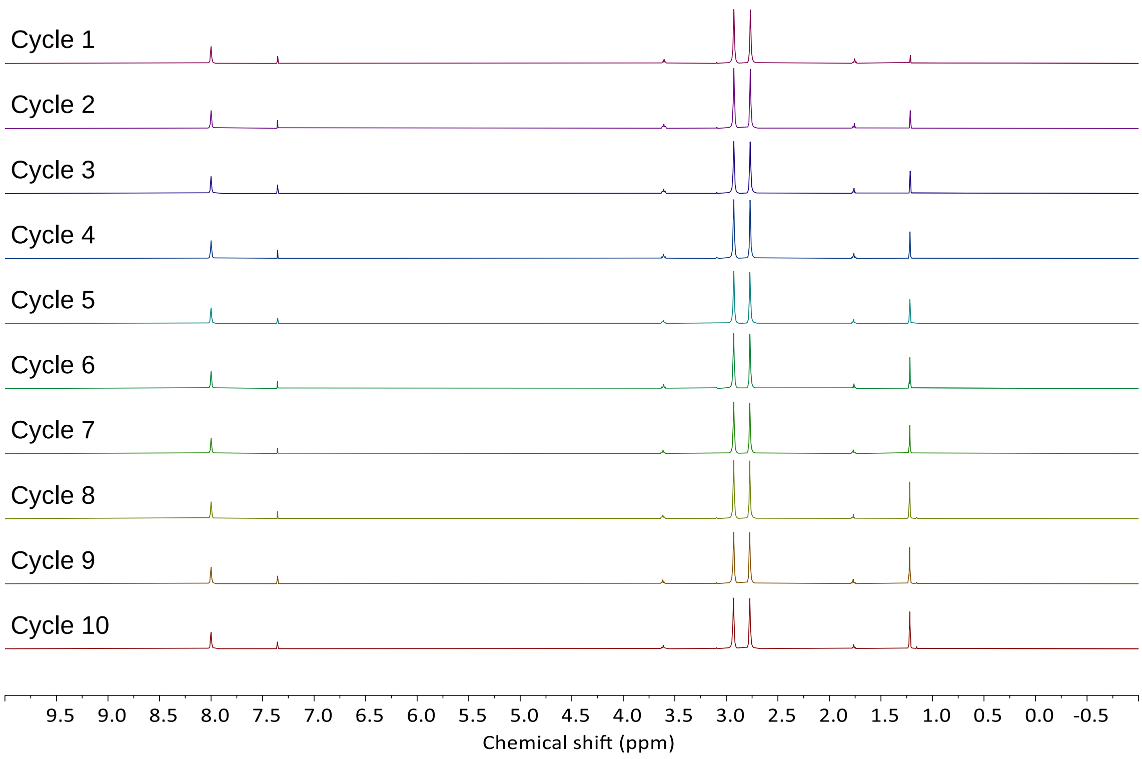

Figure S73. Stacked ^{1}H NMR spectra (400 MHz, DMF) of [K(18c6)]$_3$[**4**] (1 mol%) catalyst recycling in the hydroboration of N_2O.

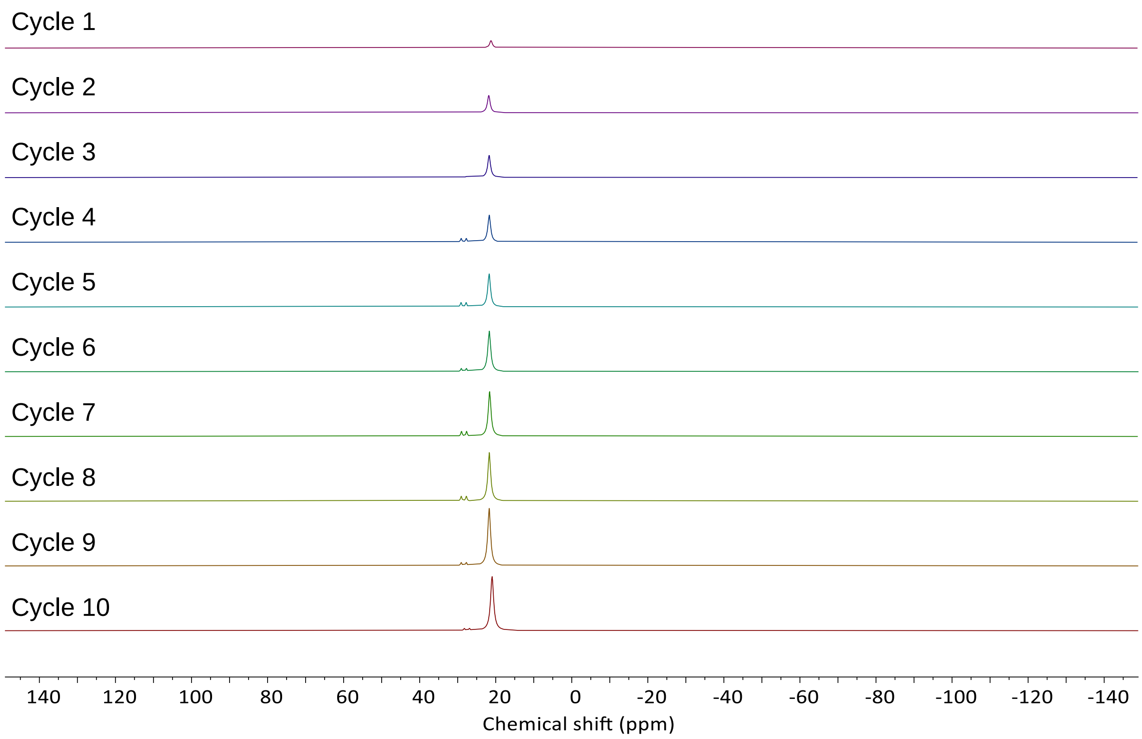

Figure S74. Stacked ^{11}B NMR spectra (128 MHz, DMF) of [K(18c6)]$_3$[**4**] (1 mol%) catalyst recycling in the hydroboration of N_2O.

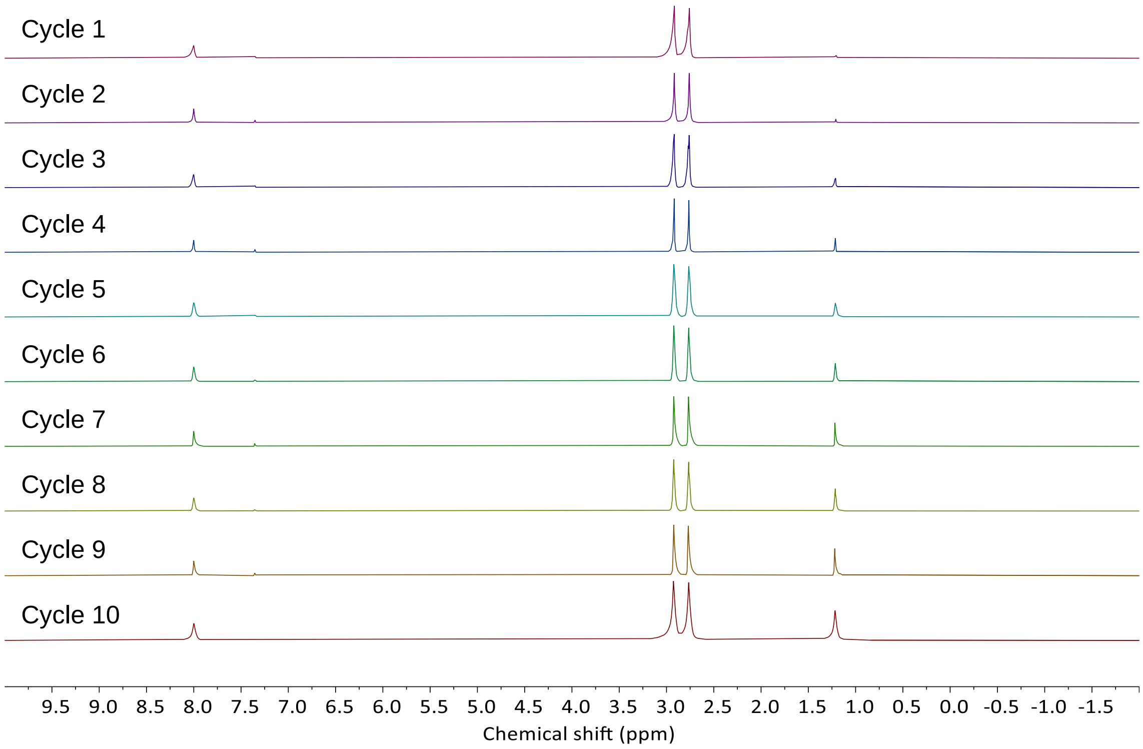

Figure S75. Stacked ^{1}H NMR spectra (400 MHz, DMF) of [K(18c6)]$_3$[**5**] (1 mol%) catalyst recycling in the hydroboration of N_2O.

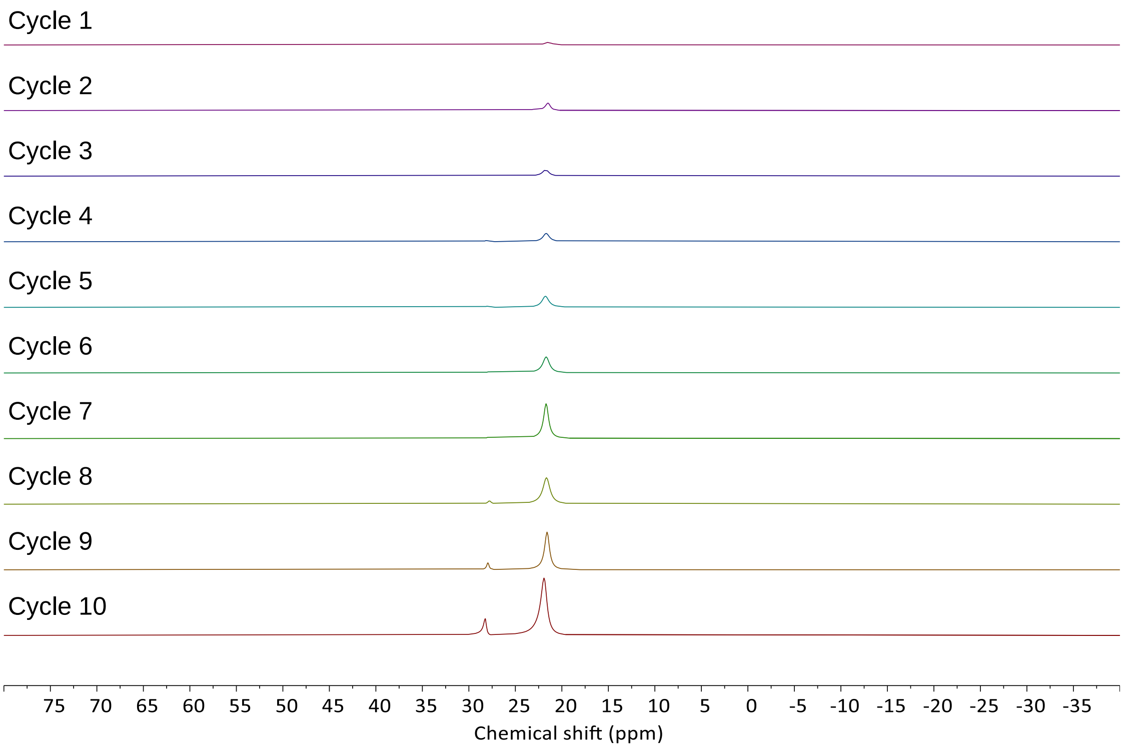

Figure S76. Stacked ^{11}B NMR spectra (128 MHz, DMF) of [K(18c6)]$_3$[**5**] (1 mol%) catalyst recycling in the hydroboration of N_2O.

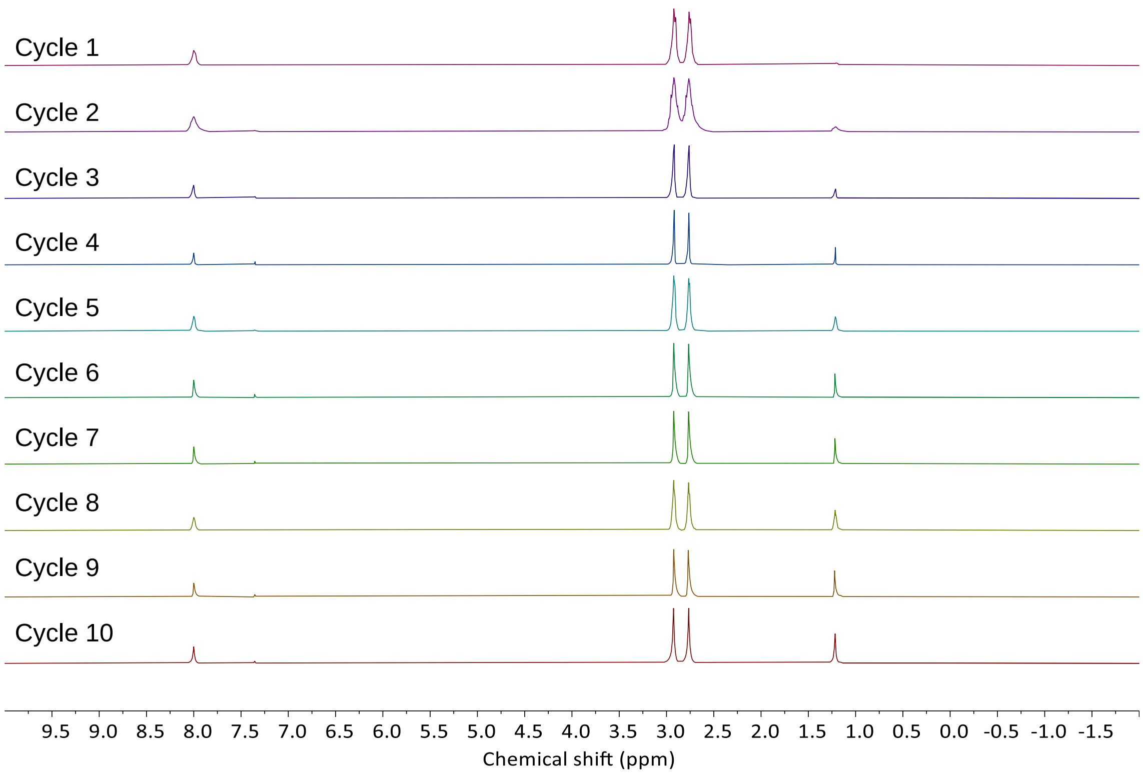

Figure S77. Stacked ^{1}H NMR spectra (400 MHz, DMF) of $[K(18c6)]_3$[**6**] (1 mol%) catalyst recycling in the hydroboration of N_2O.

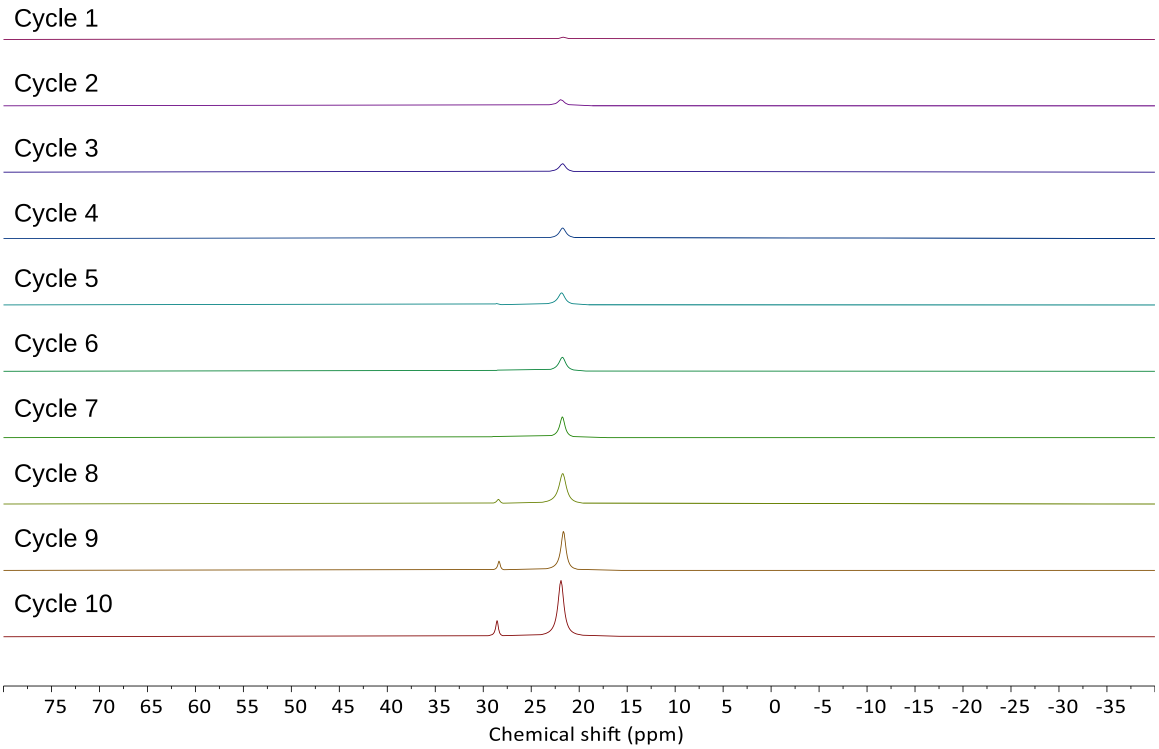

Figure S78. Stacked ^{11}B NMR spectra (128 MHz, DMF) of $[K(18c6)]_3$[**6**] (1 mol%) catalyst recycling in the hydroboration of N_2O.

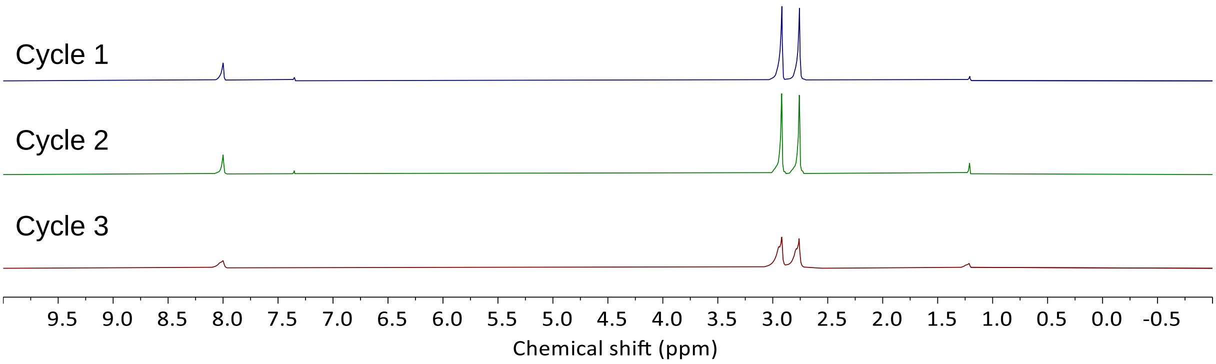

Figure S79. Stacked ^{1}H NMR spectra (400 MHz, DMF) of $[K(18c6)]_2[P_{16}]$ (1 mol%) catalyst recycling in the hydroboration of N_2O.

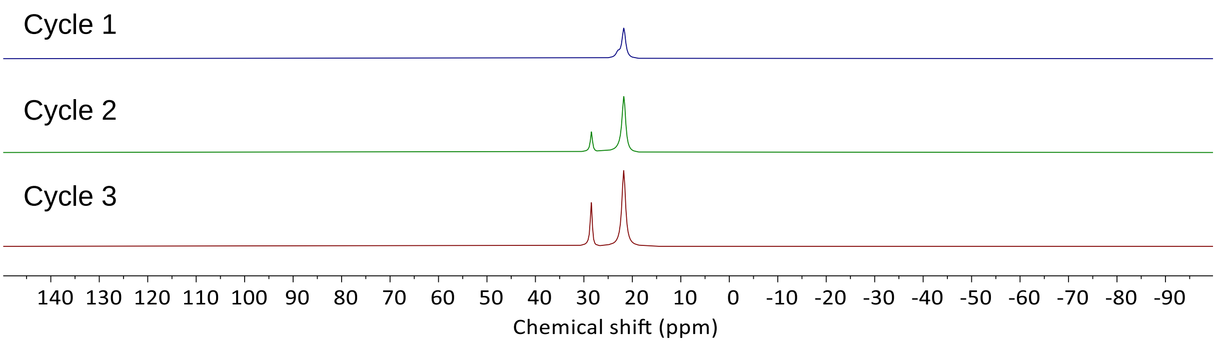

Figure S80. Stacked ^{11}B NMR spectra (128 MHz, DMF) of $[K(18c6)]_2[P_{16}]$ (1 mol%) catalyst recycling in the hydroboration of N_2O.

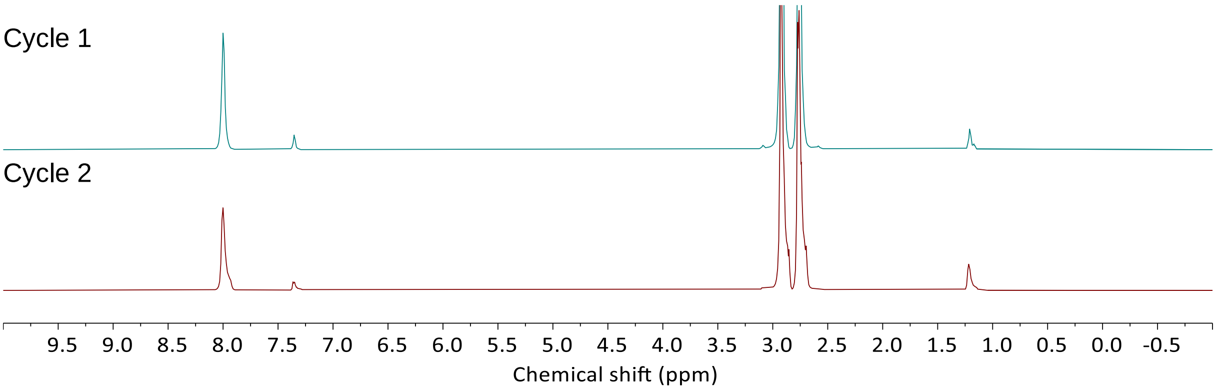

Figure S81. Stacked ^{1}H NMR spectra (400 MHz, DMF) of $[K(crypt)]_2[As_4O_7]$ (1 mol%) catalyst recycling in the hydroboration of N_2O.

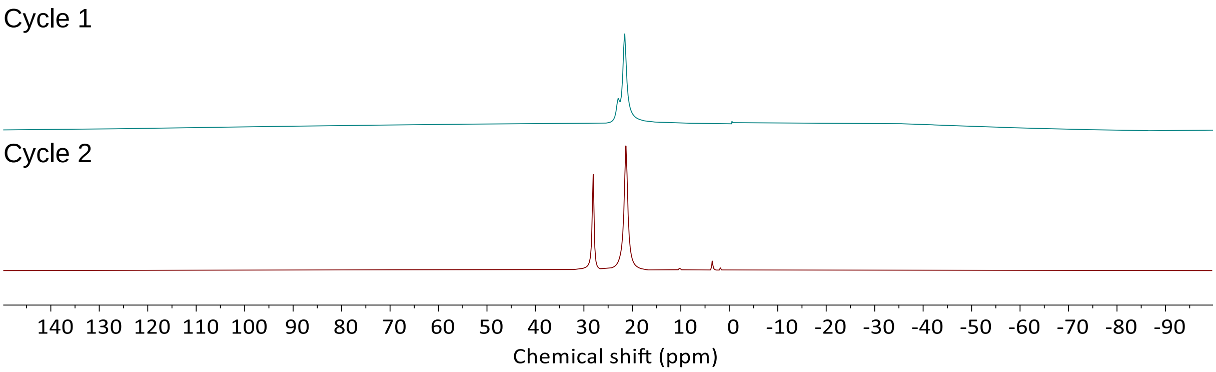

Figure S82. Stacked ^{11}B NMR spectra (128 MHz, DMF) of $[K(crypt)]_2[As_4O_7]$ (1 mol%) catalyst recycling in the hydroboration of N_2O.

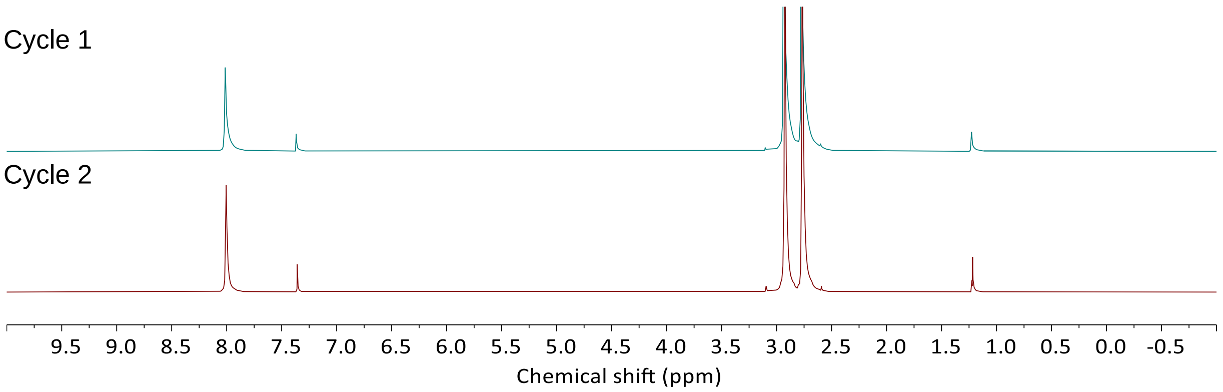

Figure S83. Stacked ^{1}H NMR spectra (400 MHz, DMF) of As_4O_6 (10 mol%) catalyst recycling in the hydroboration of N_2O.

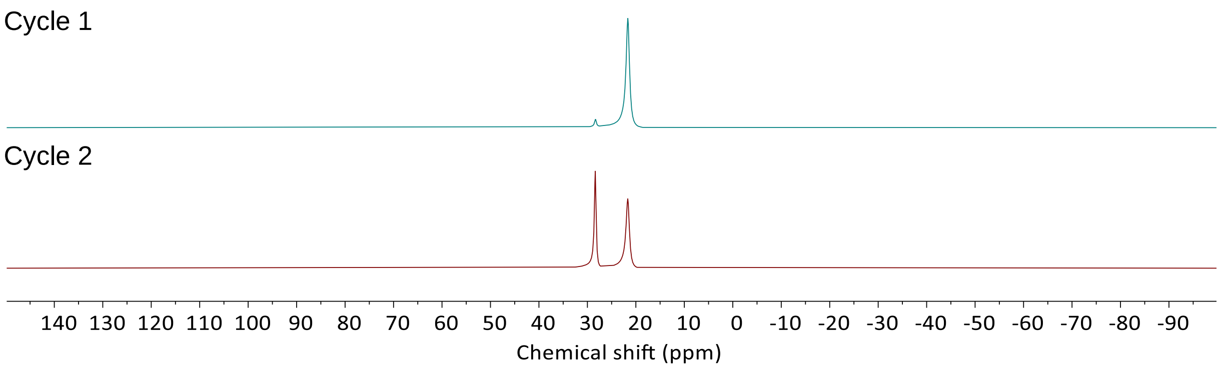

Figure S84. Stacked ^{11}B NMR spectra (128 MHz, DMF) of As_4O_6 (10 mol%) catalyst recycling in the hydroboration of N_2O.

5.2. Catalytic hydroboration of N_2O using recovered catalysts

To a J Young NMR tube, a solution of catalyst (0.7 μmol, 0.01 eq. [K(18c6)]$_2$[**4**], [K(18c6)]$_3$[**5**], or [K(18c6)]$_3$[**6**]) in DMF (0.5 mL) and HBpin (10.7 μL, 0.074 mmol, 1.0 eq.) was added. C_6H_6 (10 μL, 0.11 mmol) was added as an internal standard. The reaction mixture was immediately frozen at –78 °C. While frozen, the headspace in the NMR tube was evacuated. The headspace was refilled with N_2O (1 atm) and the NMR tube was removed from the cooling bath and the solution was allowed to thaw. The reaction mixture was allowed to react for 2h and was monitored by ^{1}H, ^{11}B and $^{11}B\{^{1}H\}$ NMR spectroscopy. NMR conv. was determined by integration of the crude ^{1}H NMR spectrum using the C_6H_6 as internal standard (^{1}H δ = 7.35 ppm). The reaction mixture was transferred into a J Young ampoule. The volatiles were removed from the reaction mixture under reduced pressure and the remaining solids were washed with

hexane (3 x 5 mL). The residue was dried under reduced pressure and subsequently was dissolved in DMF (0.5 mL) and transferred in a J Young NMR tube. HBpin (10.7 µL, 0.074 mmol, 1.0 eq.) was added. C_6H_6 (10 µL, 0.11 mmol) was added as an internal standard. The reaction mixture was immediately frozen at –78 °C. While frozen, the headspace in the NMR tube was evacuated. The headspace was refilled with N_2O (1 atm) and the NMR tube was removed from the cooling bath and the solution was allowed to thaw. The reaction mixture was monitored by ^{1}H, ^{11}B and $^{11}B\{^{1}H\}$ NMR spectroscopy. NMR conv. was determined by integration of the crude ^{1}H NMR spectrum using the C_6H_6 as internal standard (^{1}H δ = 7.35 ppm).

Similar TONs (+/– 5) were obtained despite 'fresh' vs. 'recovered' catalysts being used. A slight decrease in TOF (5-10%) was observed, which could be due to imperfect recovery of the catalysts rather than decomposition of the catalysts.

Table S9. Catalyst ([K(18c6)]$_2$[**4**], [K(18c6)]$_3$[**5**], and [K(18c6)]$_3$[**6**]) recovery conversions.

Cycle	Time (min)	[K(18c6)]$_2$[**4**] (1 mol%)		[K(18c6)]$_3$[**5**] (1 mol%)		[K(18c6)]$_3$[**6**] (1 mol%)	
		NMR Conv. **7** (%)	NMR Conv. **8** (%)	NMR Conv. **7** (%)	NMR Conv. **8** (%)	NMR Conv. **7** (%)	NMR Conv. **8** (%)
1 (Fresh catalysts)	120	90	9	87	12	80	19
2 (recovered catalysts)	120-140	89	8	95	0	88	11

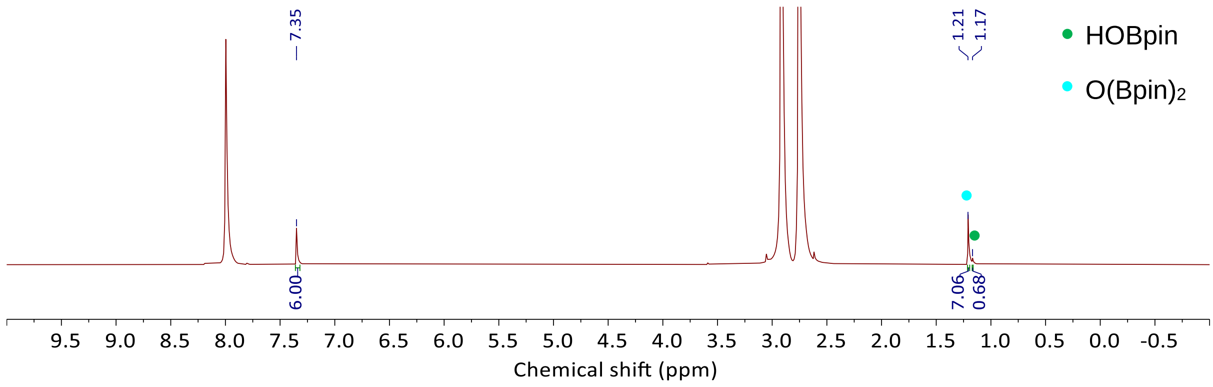

Figure S85. ^{1}H NMR spectra (400 MHz, DMF) of $[K(18c6)]_2$[**4**] recovered catalyst recycling in the hydroboration of N_2O.

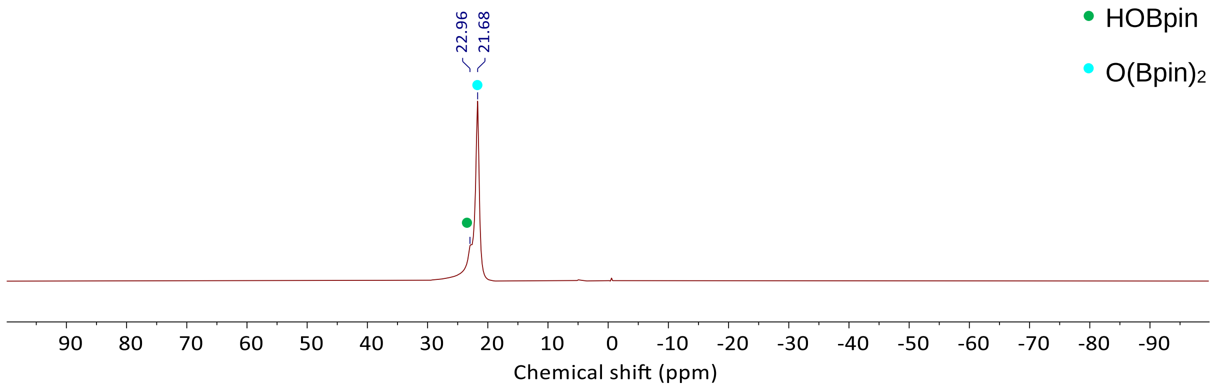

Figure S86. ^{11}B NMR spectra (128 MHz, DMF) of $[K(18c6)]_2$[**4**] recovered catalyst recycling in the hydroboration of N_2O.

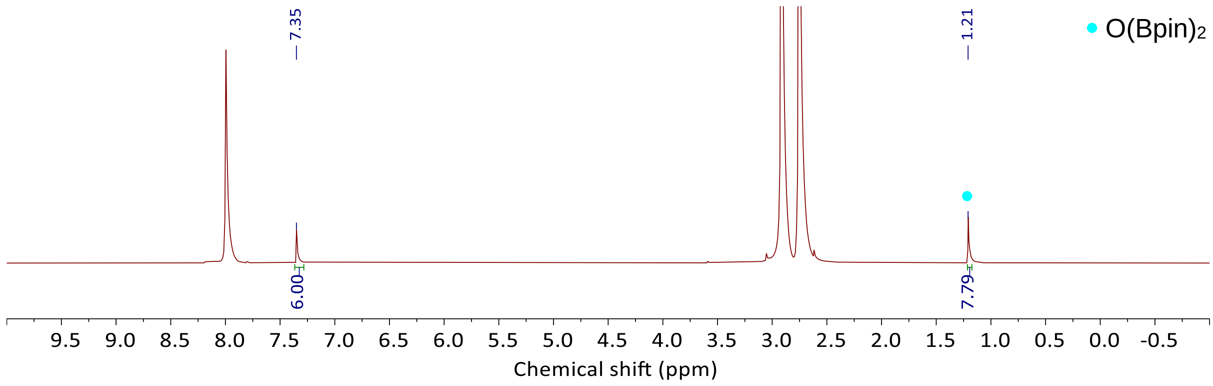

Figure S87. ^{1}H NMR spectra (400 MHz, DMF) of $[K(18c6)]_3$[**5**] recovered catalyst recycling in the hydroboration of N_2O.

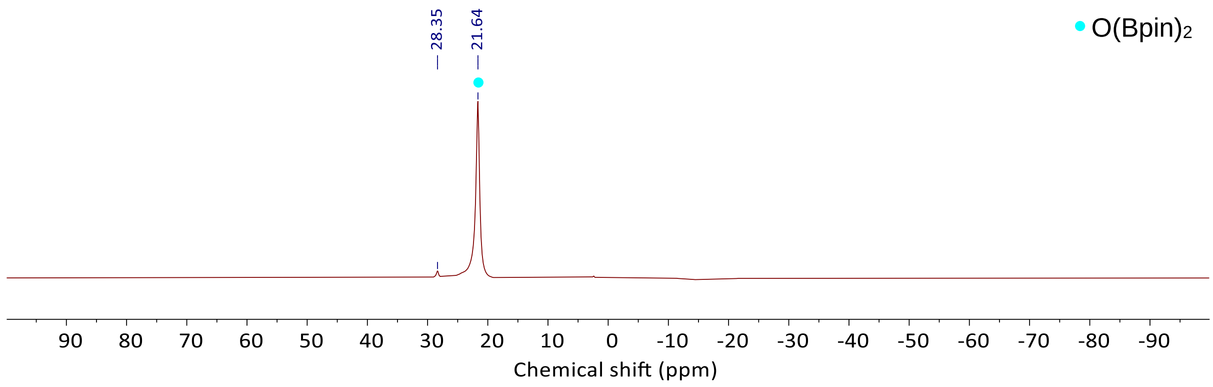

Figure S88. ^{11}B NMR spectra (128 MHz, DMF) of [K(18c6)]$_3$[**5**] recovered catalyst recycling in the hydroboration of N_2O.

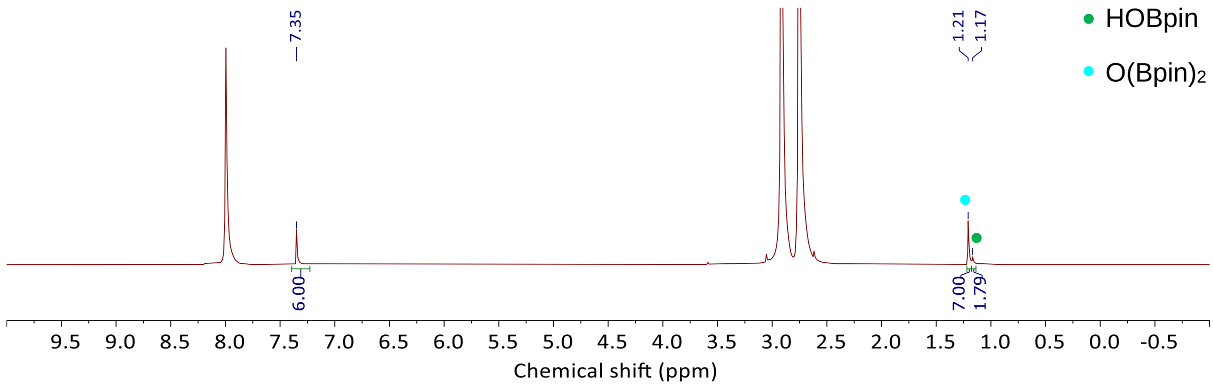

Figure S89. ^{1}H NMR spectra (400 MHz, DMF) of [K(18c6)]$_3$[**6**] recovered catalyst recycling in the hydroboration of N_2O.

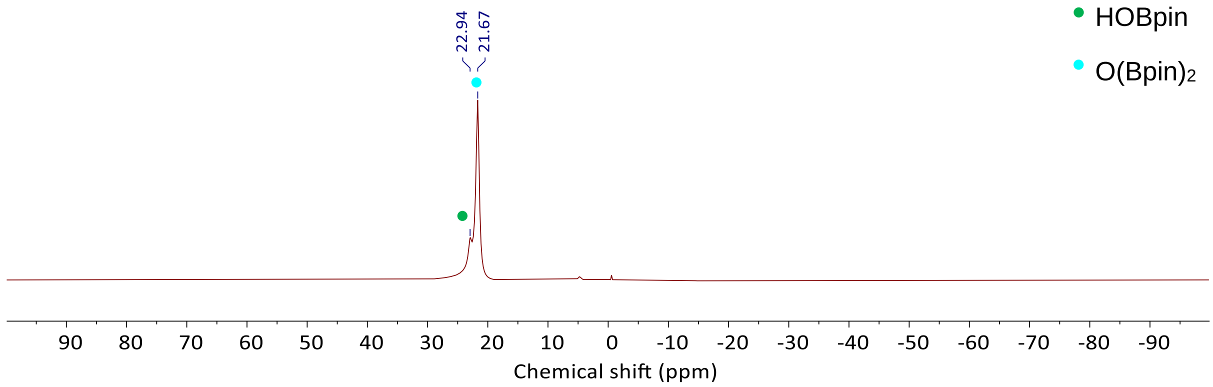

Figure S90. ^{11}B NMR spectra (128 MHz, DMF) of [K(18c6)]$_3$[**6**] recovered catalyst recycling in the hydroboration of N_2O.

6. CO_2 vs N_2O selectivity studies

For the reactions below, 1 atm CO_2 and 1 atm N_2O was added to separate empty J Young NMR tubes. The gas filled tubes were used to condense the appropriate amount of gas into the reaction NMR tube: approximately 1 atm partial pressure of N_2O and 1 atm partial pressure of CO_2, with a total of 2 atm pressure in the NMR tube.

We note that the independent reaction of $[K(18c6)]_3$[**5**] and $[K(18c6)]_3$[**6**] with N_2O is extremely rapid compared to the independent reaction of $[K(18c6)]_3$[**5**] and $[K(18c6)]_3$[**6**] with CO_2. At this time, we believe that the observed selective reduction of N_2O over CO_2 is related to the rapid adsorption of N_2O on the cluster, and rapid and irreversible O-transfer, however this is still being investigated.

6.1. Reactivity of $[K(18c6)]_3$[5] with N_2O and CO_2 gas mixture

To a J Young NMR tube, a solution of $[K(18c6)]_3$[**5**] (0.7 µmol, 0.01 eq.) in DMF (0.5 mL) and HBpin (10.7 µL, 0.074 mmol, 1.0 eq.) was added. C_6H_6 (10 µL, 0.11 mmol) was added as an internal standard (^{1}H δ = 7.35 ppm). The reaction mixture was immediately frozen and then degassed by freeze-pump-thaw cycles, after which N_2O was condensed in followed by CO_2. The reaction was allowed to react overnight and was monitored by ^{1}H, ^{11}B and ^{13}C{^{1}H} NMR spectroscopy.

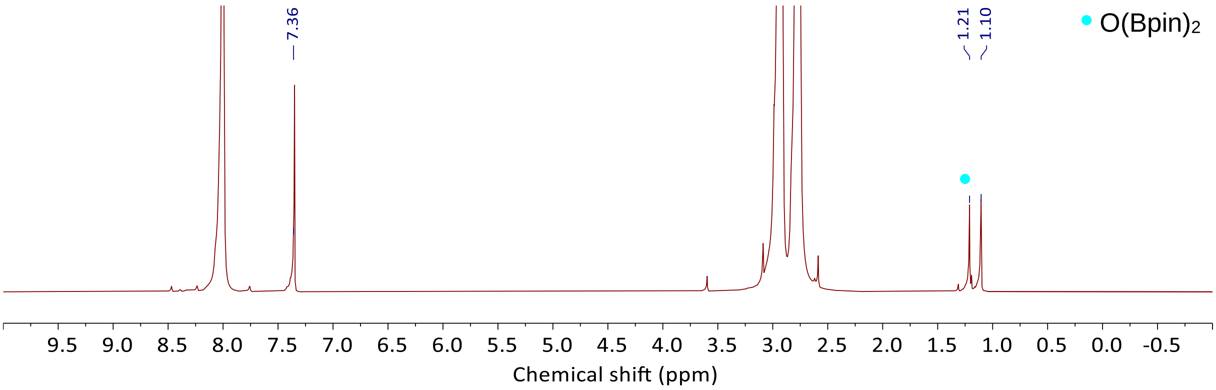

Figure S91. ^{1}H NMR spectrum (400 MHz, DMF) of the hydroboration of CO_2 and N_2O gas mixture catalyzed by $[K(18c6)]_3$[**5**] using HBpin.

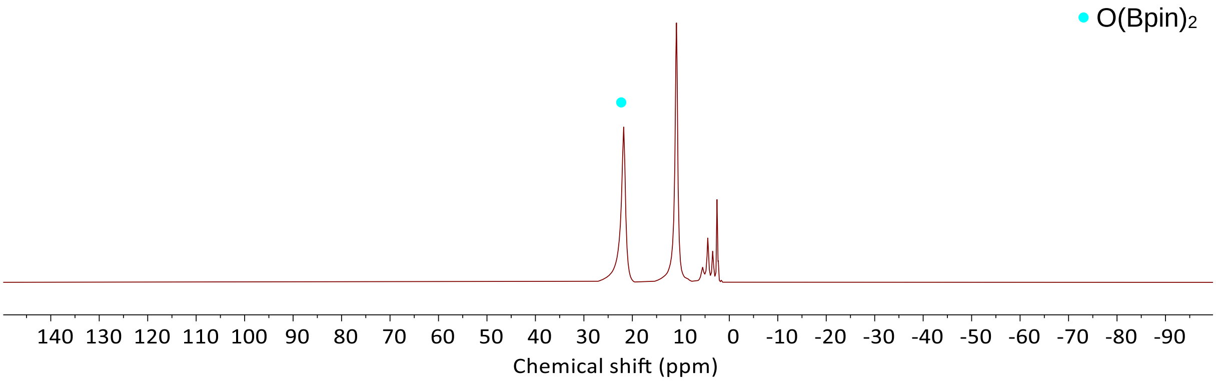

Figure S92. ^{11}B NMR spectrum (128 MHz, DMF) of the hydroboration of CO_2 and N_2O gas mixture catalyzed by $[K(18c6)]_3$[**5**] using HBpin.

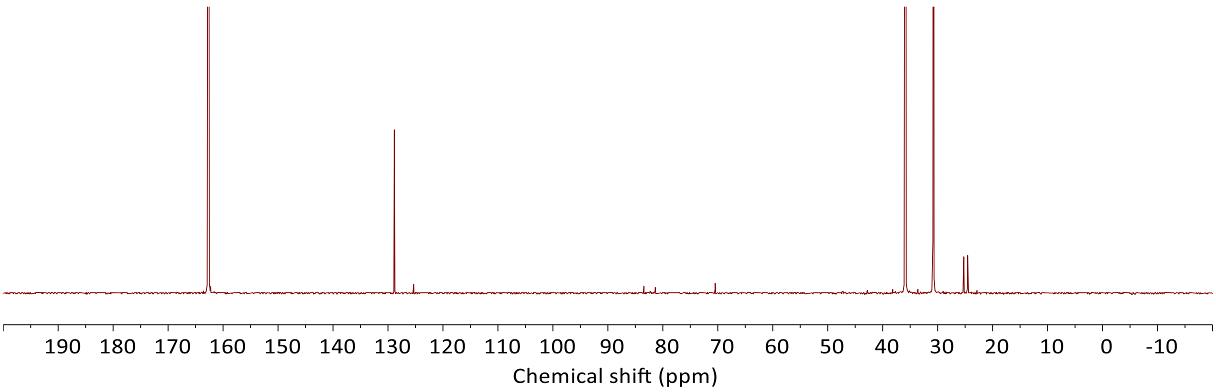

Figure S93. $^{13}C\{^1H\}$ NMR spectrum (101 MHz, DMF) of the hydroboration of CO_2 and N_2O gas mixture catalyzed by $[K(18c6)]_3$[**5**] using HBpin.

6.2. Reactivity of $[K(18c6)]_3[6]$ with N_2O and CO_2 gas mixture

To a J Young NMR tube, a solution of $[K(18c6)]_3$[**6**] (0.7 μmol, 0.01 eq.) in DMF (0.5 mL) and HBpin (10.7 μL, 0.074 mmol, 1.0 eq.) was added. C_6H_6 (10 μL, 0.11 mmol) was added as an internal standard (^1H δ = 7.35 ppm). The reaction mixture was immediately frozen and then degassed by freeze-pump-thaw cycles, after which N_2O was condensed in followed by CO_2. The reaction was allowed to react overnight and was monitored by ^1H, ^{11}B and $^{13}C\{^1H\}$ NMR spectroscopy.

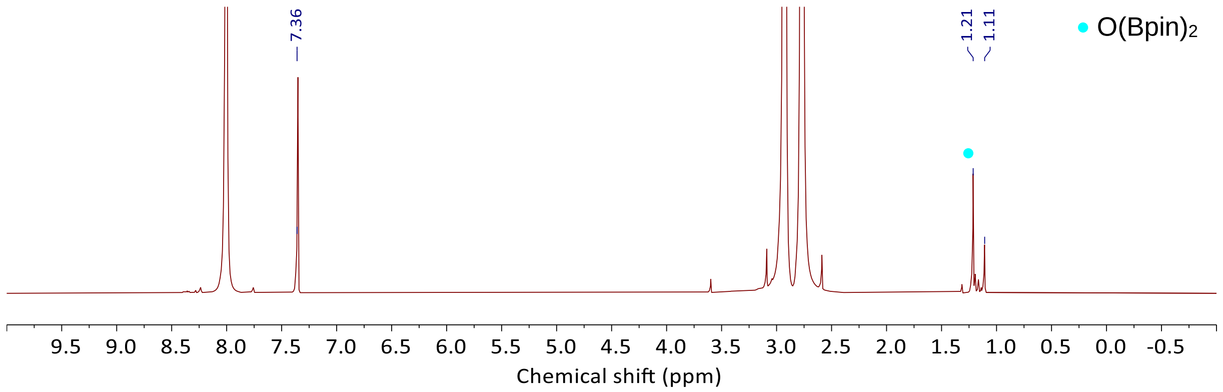

Figure S94. ^{1}H NMR spectrum (400 MHz, DMF) of the hydroboration of CO_2 and N_2O gas mixture catalyzed by $[K(18c6)]_3$[**6**] using HBpin.

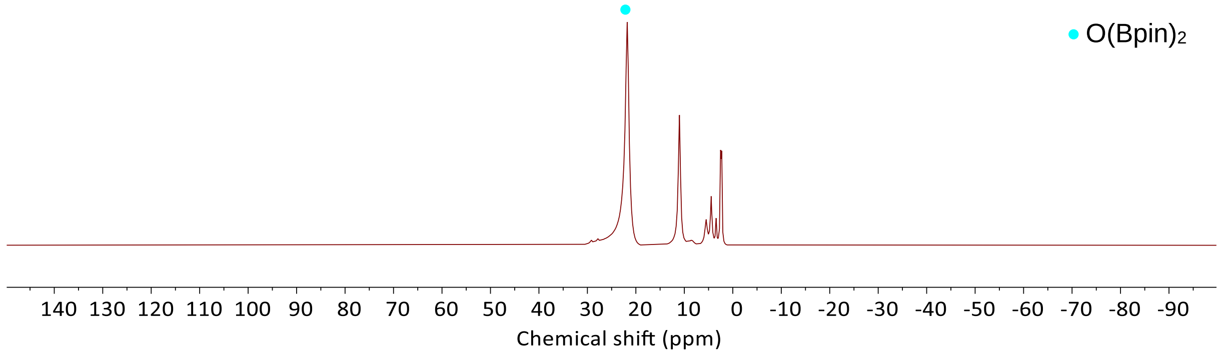

Figure S95. ^{11}B NMR spectrum (128 MHz, DMF) of the hydroboration of CO_2 and N_2O gas mixture catalyzed by $[K(18c6)]_3$[**6**] using HBpin.

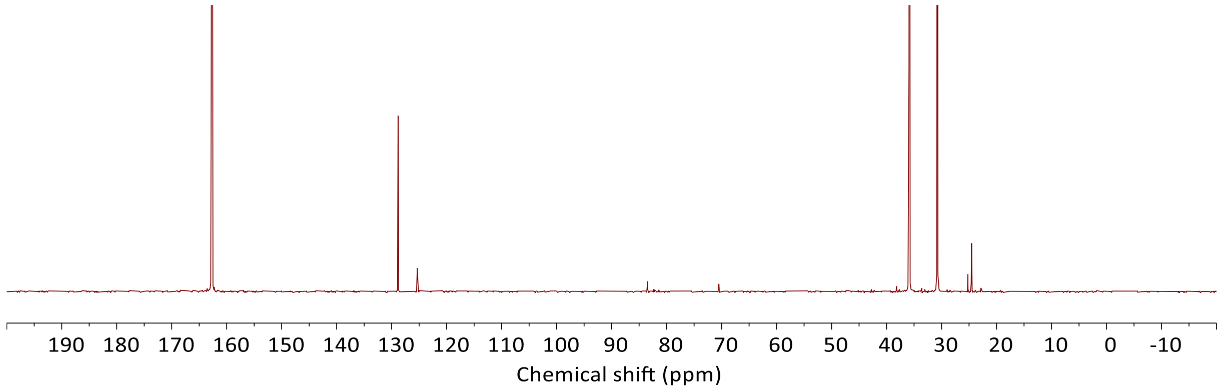

Figure S96. $^{13}C\{^{1}H\}$ NMR spectrum (101 MHz, DMF) of the hydroboration of CO_2 and N_2O gas mixture catalyzed by $[K(18c6)]_3$[**6**] using HBpin.

7. Oxygen transfer to sulfur

7.1. Stoichiometric Reaction $[K(crypt)]_2[As_4O_7]$ with S_8

To a sample vial, S_8 (4.8 mg, 0.019 mmol, 1 eq.) and a solution of $[K(crypt)]_2$[**9**] (23.0 mg, 0.019 mmol, 1 eq.) in DMF (0.5 mL) was added. The mixture was allowed to react overnight. An immediate colour change was observed from colourless to dark red. The reaction mixture was filtered and slow diffusion of diethyl ether into the filtrate at –30 °C resulted in red needle-type crystals. The supernatant was removed from the crystals. The crystals were further washed with diethyl ether and subsequently dried under reduced pressure yielding a red powder.

Yield: 10.1 mg, 47% (based on As_4O_7 providing 7 oxygen atoms).

Mass spectrometry of reaction mixture (ESI): $[S_7O_6H]^-$: cald. 320.7824; found: 320.7880; $[S_8O_6]^{2-}$: cald. 175.8736; found: 175.8733; $[AsS_2]^-$: cald. 138.8663; found: 138.8668; $[AsS_3]^-$: cald. 170.8384; found: 170.8386; $[AsS_5]^-$: cald. 234.7825; found: 234.7858; $[AsS_6]^-$: cald. 266.7546; found: 266.7474.

Infrared (ATR): ν: 1236 cm^{-1} (S–O asymmetric stretching), 1095 cm^{-1} (symmetric S–O stretching), 601 cm^{-1} (S–O bending).

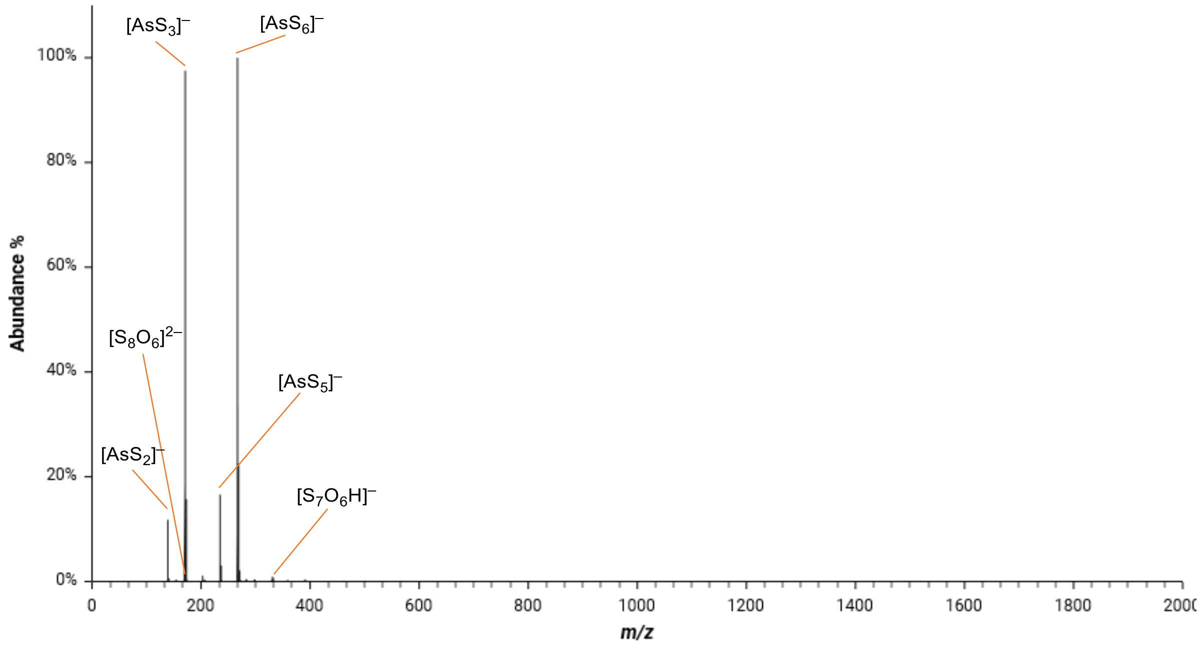

Figure S97. Mass spectrometry (ESI) results of $[K(crypt)]_2$[**9**] + S_8.

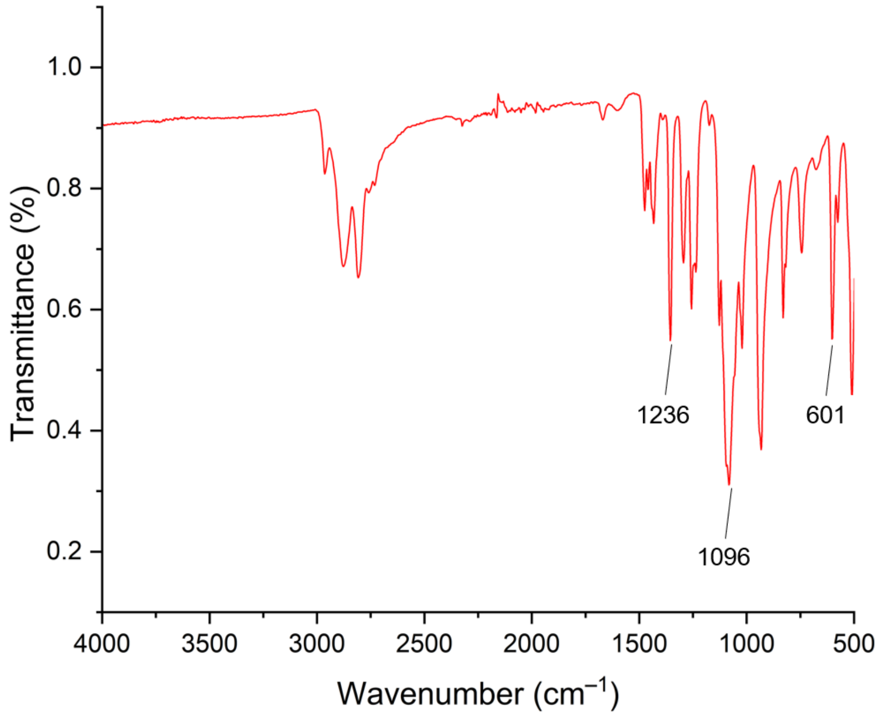

Figure S98. IR spectrum of [K(crypt)]$_2$[**9**] + S_8.

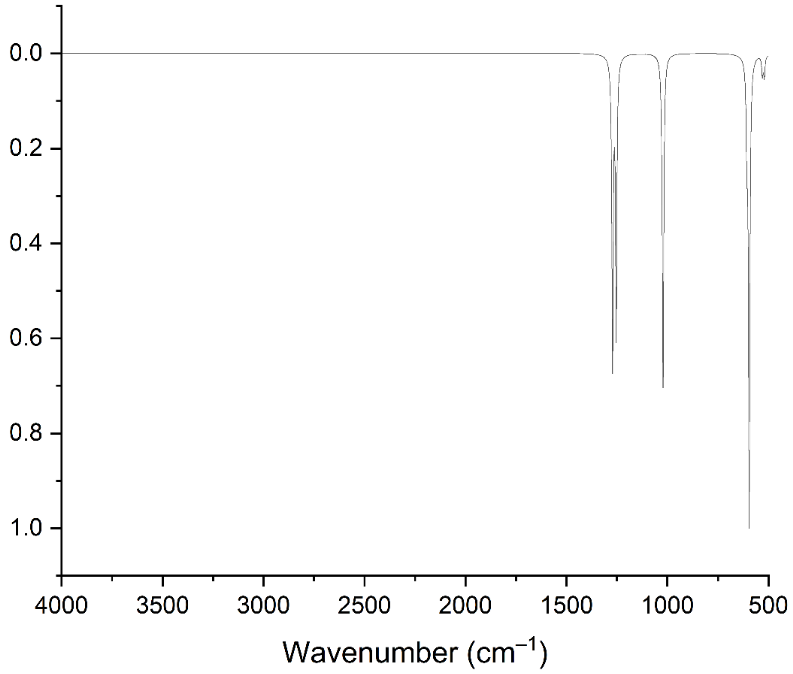

Figure S99. Calculated IR spectrum of $[S_7O_6]^{2-}$.

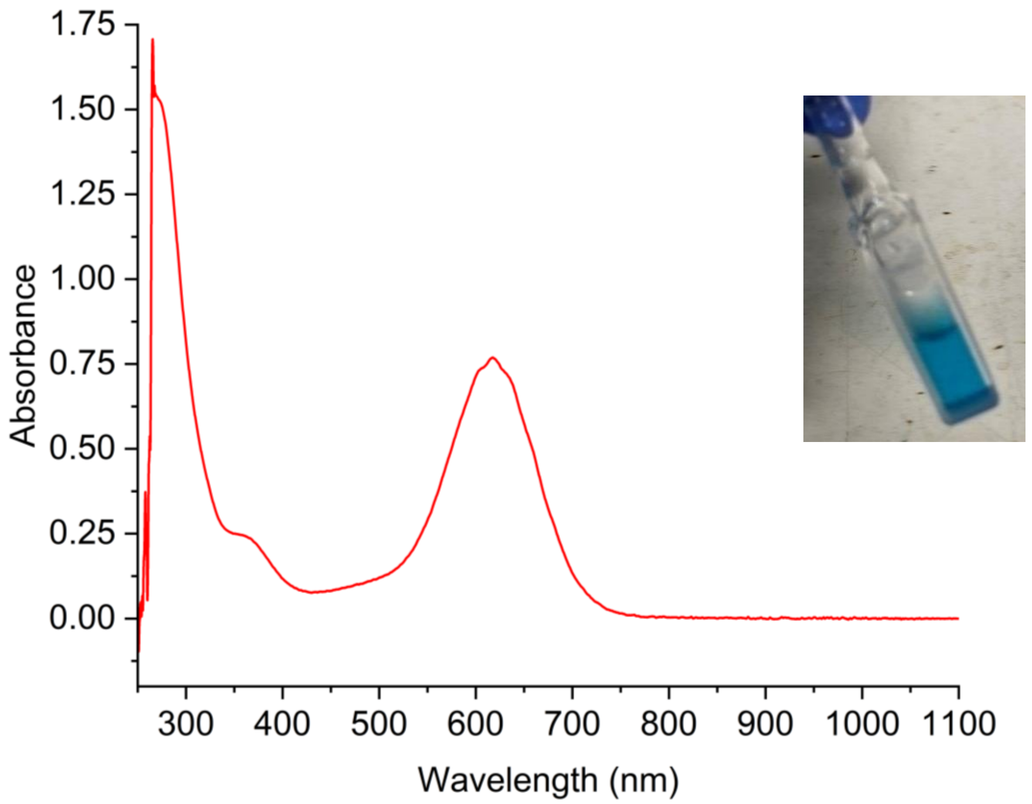

Figure S100. UV-Vis spectrum (0.06 mM, DMF) of [K(crypt)]$_2$[**9**] + S_8. Upon dilution to 0.06 mM, [K(crypt)]$_2$[**9**] + S_8 appears blue. At higher concentration it appears red.

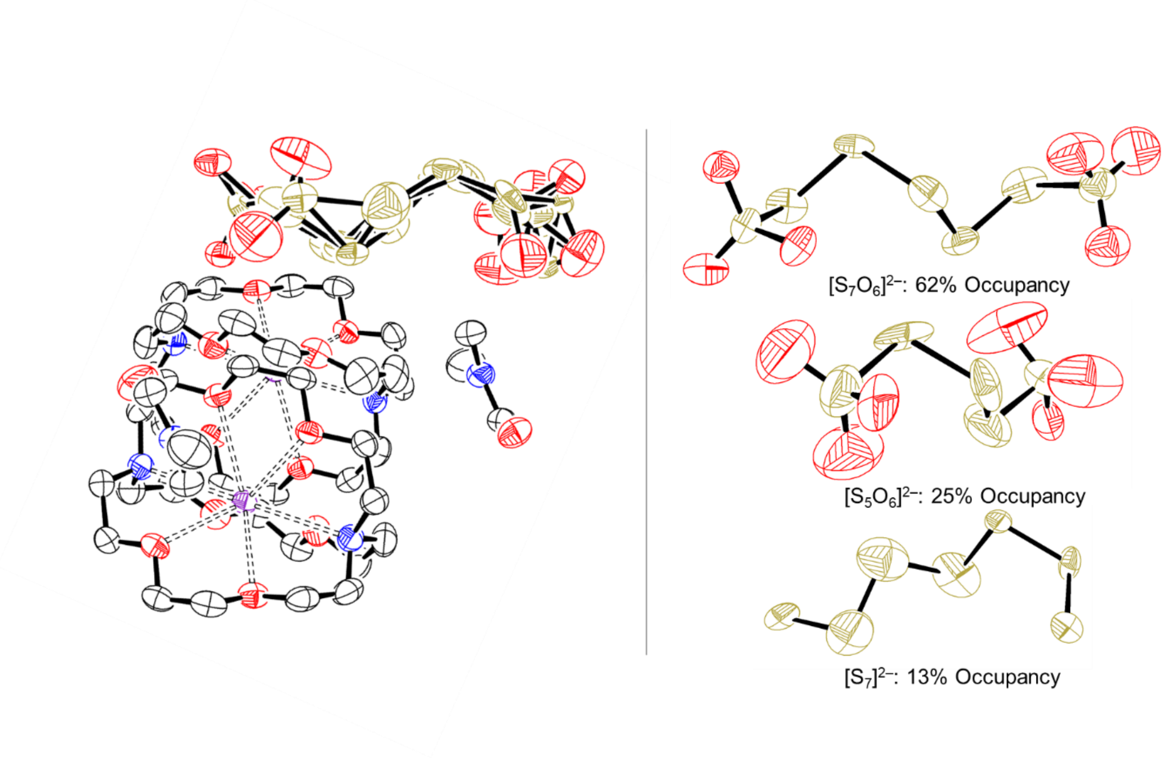

Figure S101. Molecular structure of $[K(crypt)]_2[S_{6.4}O_{5.2}]$. Anisotropic displacement ellipsoids pictured at 50% probability. Hydrogen atoms omitted for clarity. Sulfur: Bronze; Nitrogen: Blue; Carbon: White; Potassium: Purple; Oxygen: Red. CCDC code: 2434058. 3 anionic components were modelled displaced on the right.

7.2. Attempted catalytic oxygenation of S_8 by $[K(crypt)]_2[As_4O_7]$

To a J Young NMR tube S_8 (4.8 mg, 0.019 mmol, 1 eq.) and a solution of $[K(crypt)]_2$[**9**] (at loadings between 0-20 mol%) in DMF (0.5 mL) was added. The reaction mixture was degassed by freeze-pump-thaw cycles and the headspace was refilled with N_2O (1 atm). The mixture was allowed to react overnight. The NMR tubes was opened in the glovebox and 10 µL of the solution was introduced onto the FT-IR spectrometer to track the reaction. The NMR tubes was closed again and the reaction mixture was degassed by freeze-pump-thaw cycles and the headspace was refilled with N_2O (1 atm) after which 10 µL of the solution was introduced onto the FT-IR spectrometer and no change on intensity was observed in the IR spectrum over 24 hours. Based on these findings we believe transfer of oxygen from N_2O to S_8 is not catalyzed by $[K(crypt)]_2$[**9**].

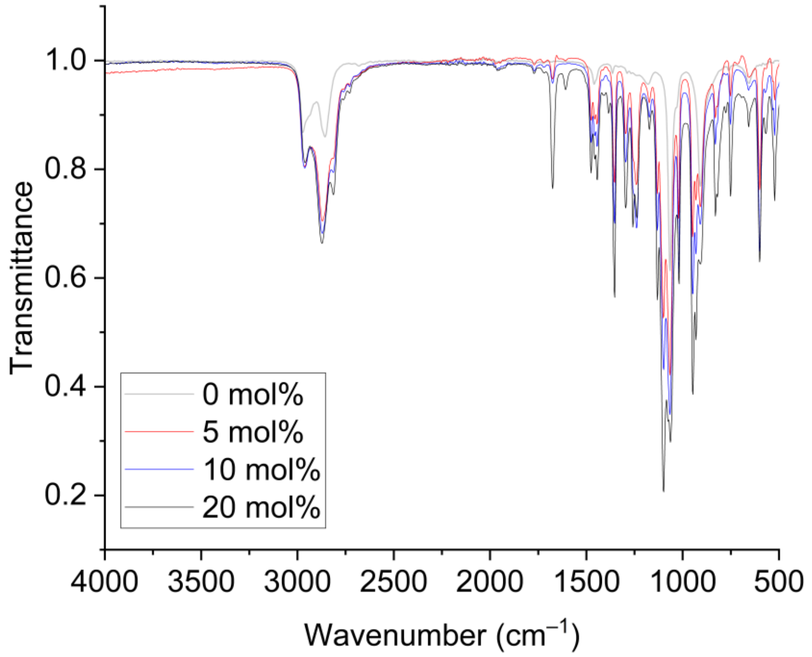

Figure S102. IR spectrum of [K(crypt)]$_2$[**9**] + S_8 at different catalysts loading.

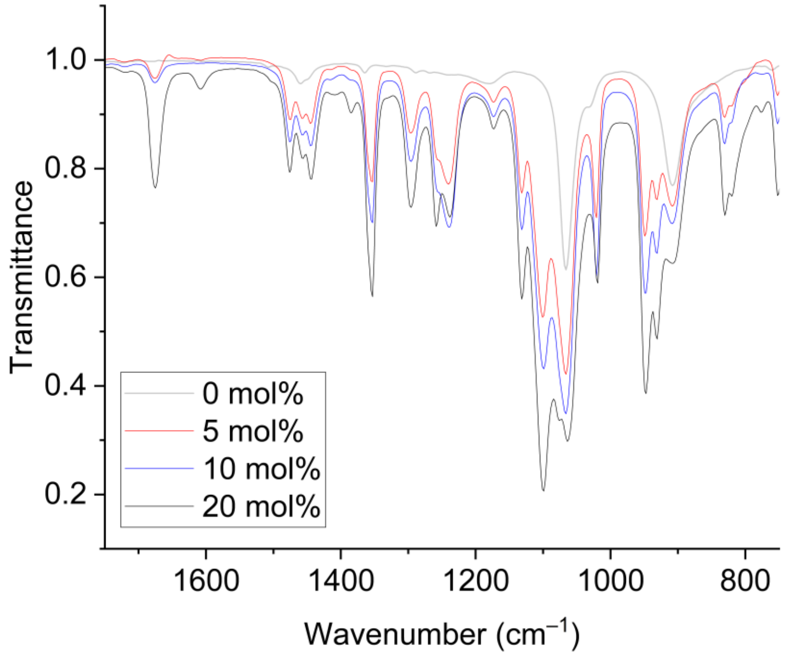

Figure S103. Expanded IR spectrum of [K(crypt)]$_2$[**9**] + S_8 at different catalysts loading.

7.3. Reaction $[K(crypt)]_2[As_4O_7]$ with SO_2

To a J Young NMR tube, a solution of $[K(crypt)]_2$[**9**] (23.0 mg, 0.019 mmol, 1 eq.) in DMF (0.5 mL) was added. The reaction mixture was degassed and the headspace was refilled with SO_2. An immediate colour change was observed from colourless to blue. The reaction mixture was allowed to react overnight, subsequently filtered and slow diffusion of diethyl ether into the filtrate resulted in colourless block-type crystals. The supernatant was removed from the crystals. The crystals were further washed with diethyl ether and subsequently dried under reduced pressure yielding a white powder.

Yield: 20.4 mg, 74% (based on As_4O_7 providing 7 oxygen atoms).

Infrared (ATR): ν: 1158 cm^{-1} (S–O symmetric stretching), 1047 cm^{-1} (asymmetric S–O stretching), 593 cm^{-1} (S–O rocking).

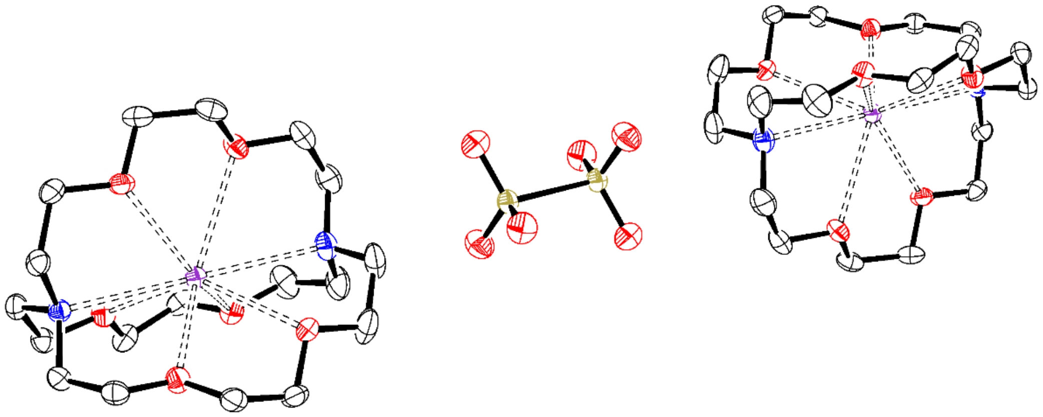

Figure S104. Molecular structure of $[K(crypt)]_2[S_2O_6]$. Anisotropic displacement ellipsoids pictured at 50% probability. Hydrogen atoms omitted for clarity. Sulfur: Bronze; Nitrogen: Blue; Carbon: White; Potassium: Purple; Oxygen: Red. CCDC code: 2434057.

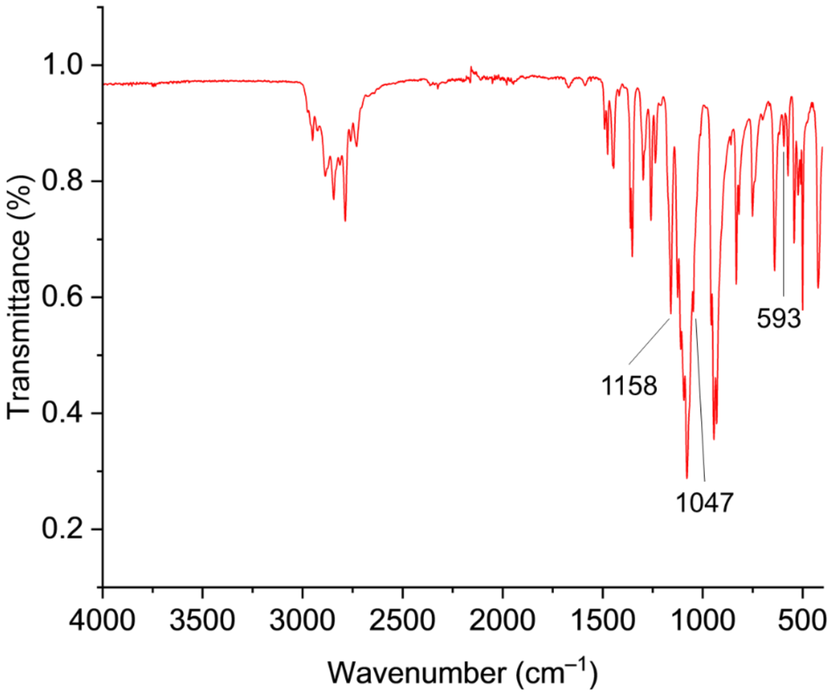

Figure S105. IR spectrum of [K(crypt)]$_2$[**9**] + SO_2.

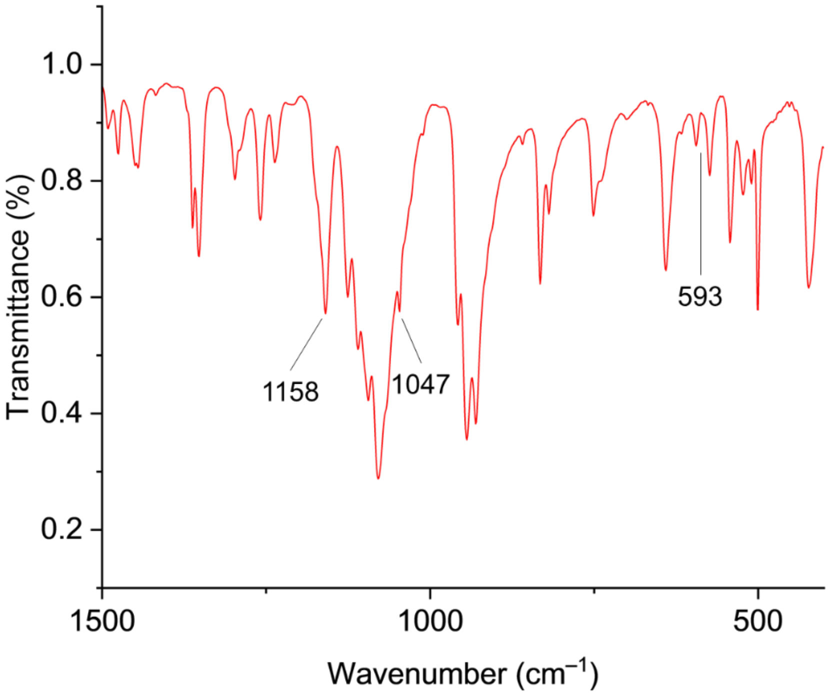

Figure S106. IR spectrum of [K(crypt)]$_2$[**9**] + SO_2. Detailed.

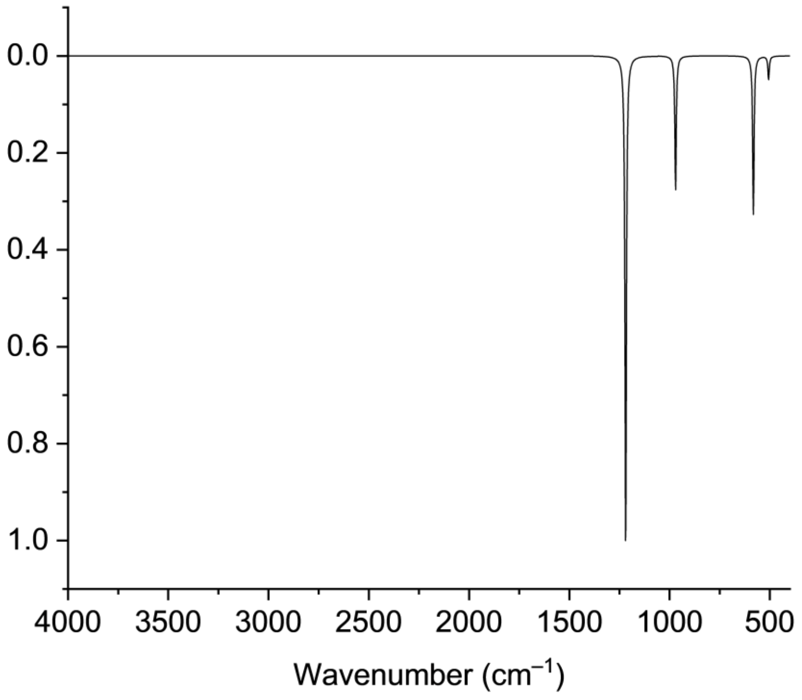

Figure S107. Calculated IR spectrum of $[S_2O_6]^{2-}$.

7.4. Stoichiometric reaction $[K(crypt)]_2[As_4O_7]$ with 4-nitrophenyl disulfide

To a sample vial, 4-nitrophenyl disulfide (5.0 mg, 0.016 mmol, 1 eq.) and a solution of $[K(crypt)]_2$[**9**] (20.1 mg, 0.016 mmol, 1 eq.) in DMF-d_7 (0.5 mL) was added. The reaction mixture was allowed to react overnight and monitored using ^{1}H and ^{13}C{^{1}H} NMR spectroscopy. Complete consumption of the 4-nitrophenyl disulfide was observed. C_6H_6 (10 μL, 0.11 mmol) was added as an internal standard (^{1}H δ = 7.35 ppm) and total NMR conversion determined to be to 98% with one major new product and a second minor new product.

The volatiles were removed under a reduced pressure to afford a dark red oily solid. A small sample was analyzed by IR spectroscopy studies. The remainder was dissolved in D_2O (0.5 mL) to acquire a ^{1}H NMR spectrum and compared to literature values.[22]

When catalytic amounts (10 mol% loading) of $[K(crypt)]_2$[**9**] is added to 4-nitrophenyl disulfide under N_2O atmosphere. Only partial consumption of 4-nitrophenyl disulfide

was observed, and no catalytic turnover was achieved. Further, 4-nitrophenyl disulfide did not react independently with N_2O or As_4O_6.

Mass spectrometry (ESI): $[C_6H_4NO_5S]^-$: cald. 201.9816; found: 201.9812; $[C_6H_4NO_6S]^-$: cald. 217.9759; found: 217.9630.

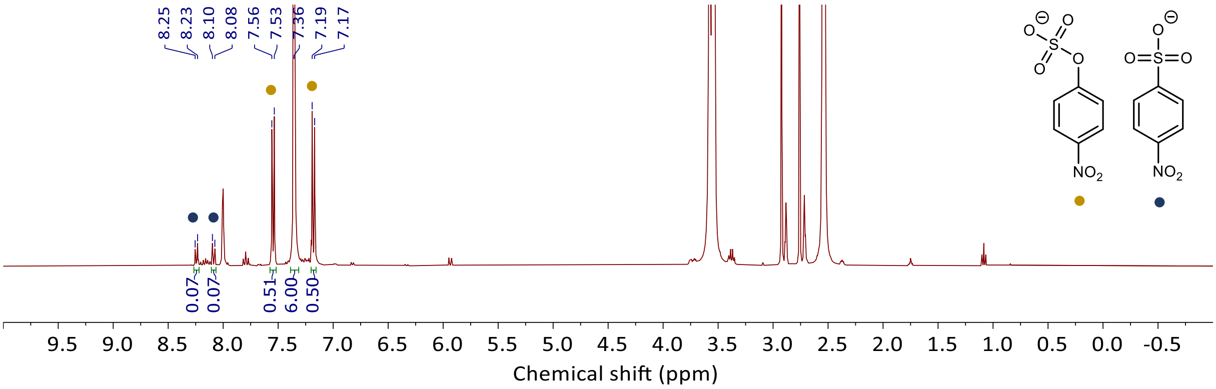

Figure S108. ^{1}H NMR spectrum (500 MHz, DMF-d$_7$) of [K(crypt)]$_2$[**9**] + 4-nitrophenyl disulfide.

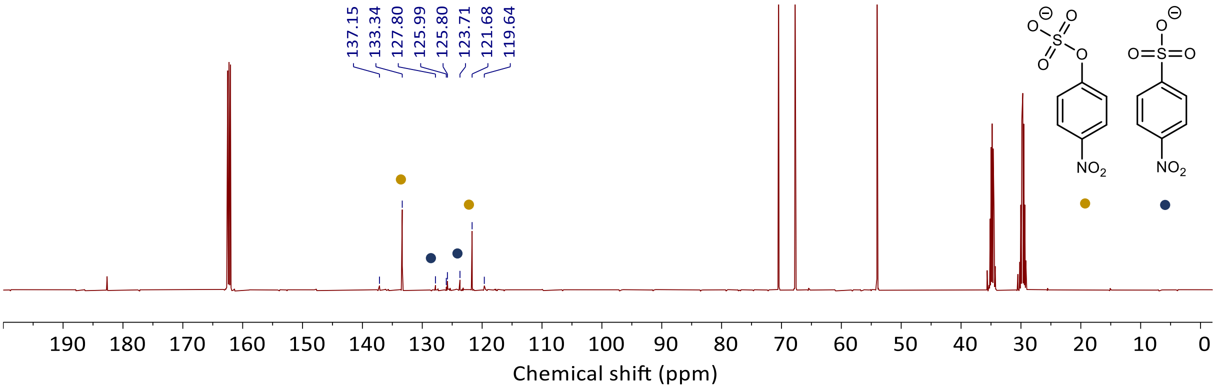

Figure S109. ^{13}C{^{1}H} NMR spectrum (125 MHz, DMF-d$_7$) of [K(crypt)]$_2$[**9**] + 4-nitrophenyl disulfide.

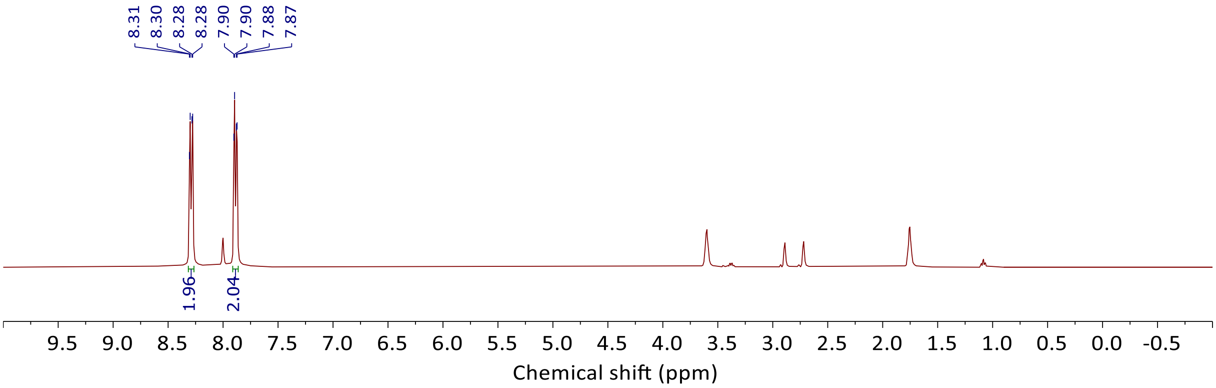

Figure S110. ^{1}H NMR spectrum (500 MHz, DMF-d$_7$) of 4-nitrophenyl disulfide.

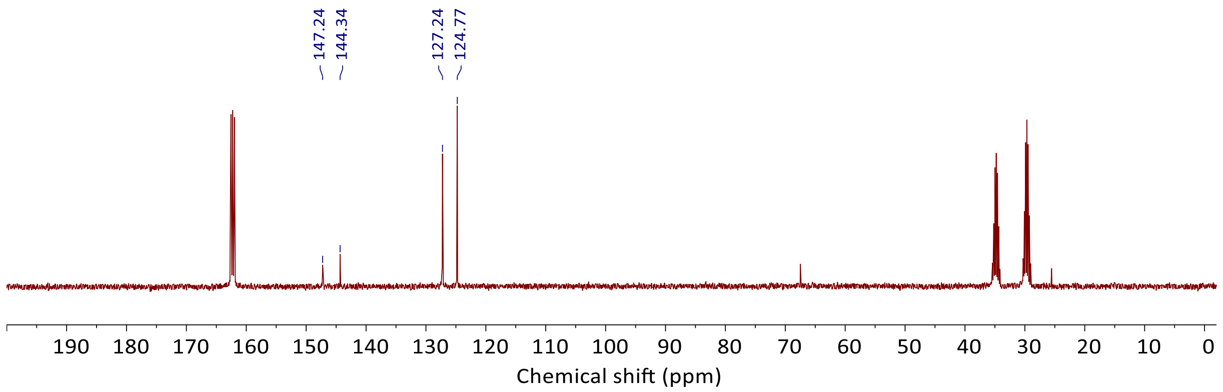

Figure S111. $^{13}C\{^{1}H\}$ NMR spectrum (125 MHz, DMF-d_7) 4-nitrophenyl disulfide.

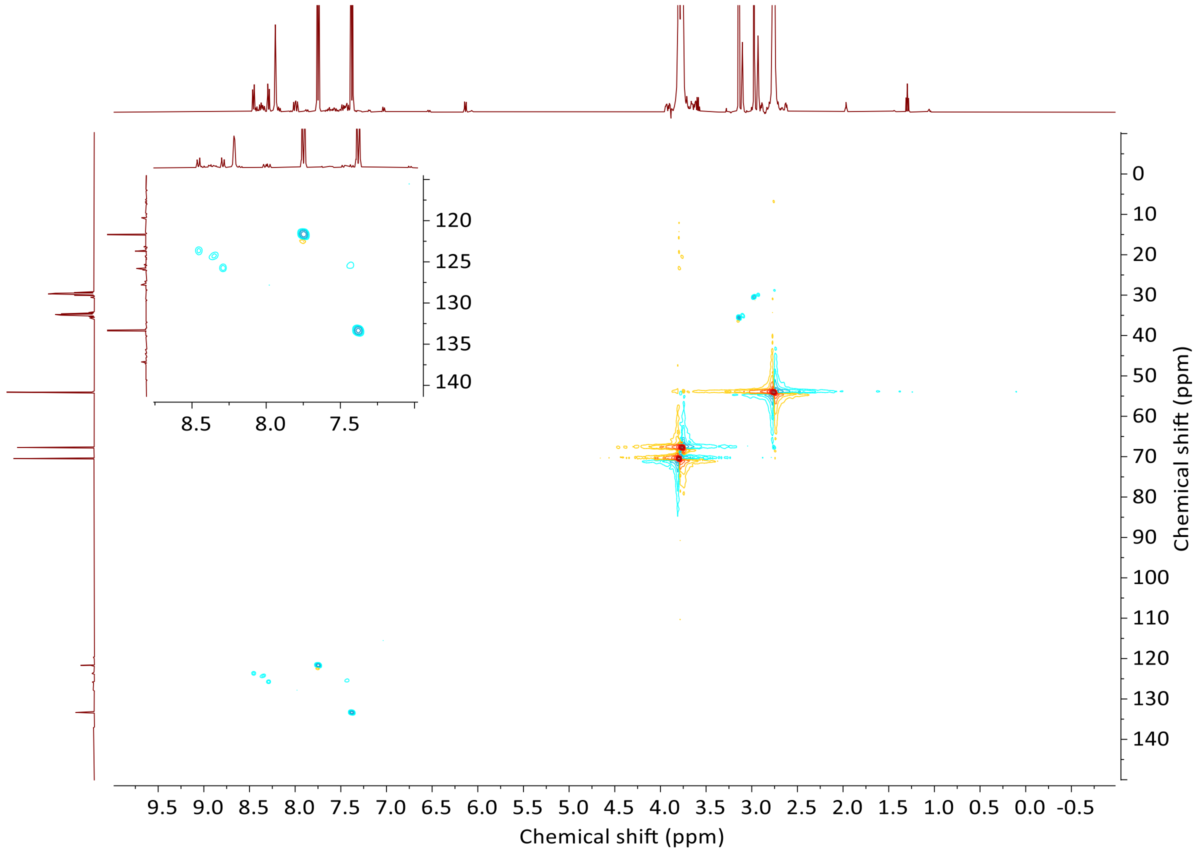

Figure S112. ^{1}H ^{13}C HSQC NMR spectrum (500, 125 MHz, DMF-d_7) of $[K(crypt)]_2[\mathbf{9}]$ + 4-nitrophenyl disulfide.

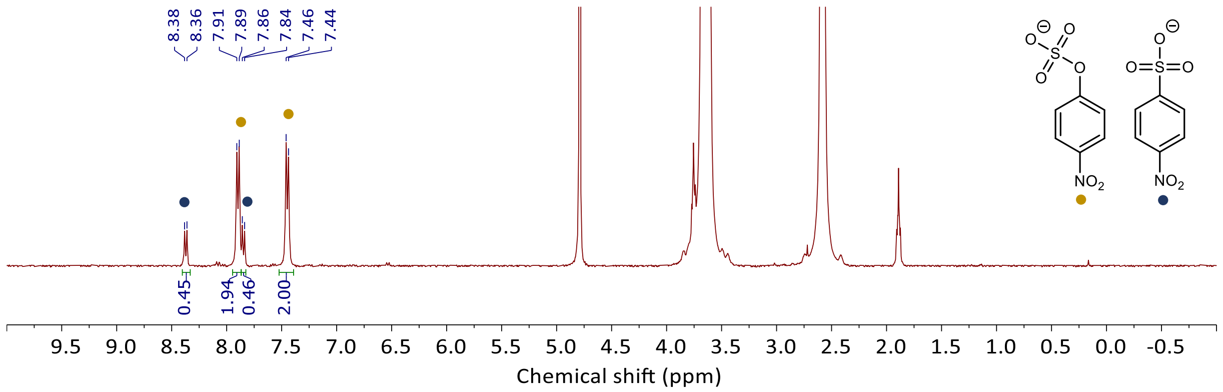

Figure S113. ^1H NMR spectrum (400 MHz, D_2O) of [K(crypt)]$_2$[**9**] + 4-nitrophenyl disulfide.

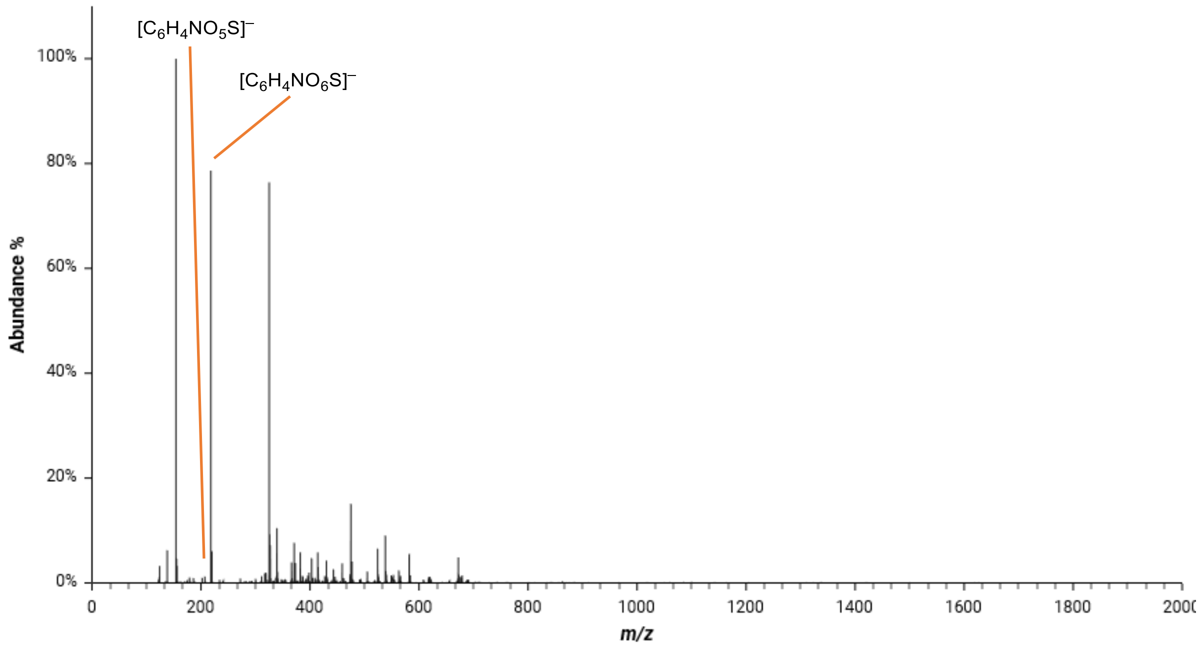

Figure S114. Mass spectrometry (ESI) results of [K(crypt)]$_2$[**9**] + 4-nitrophenyl disulfide.

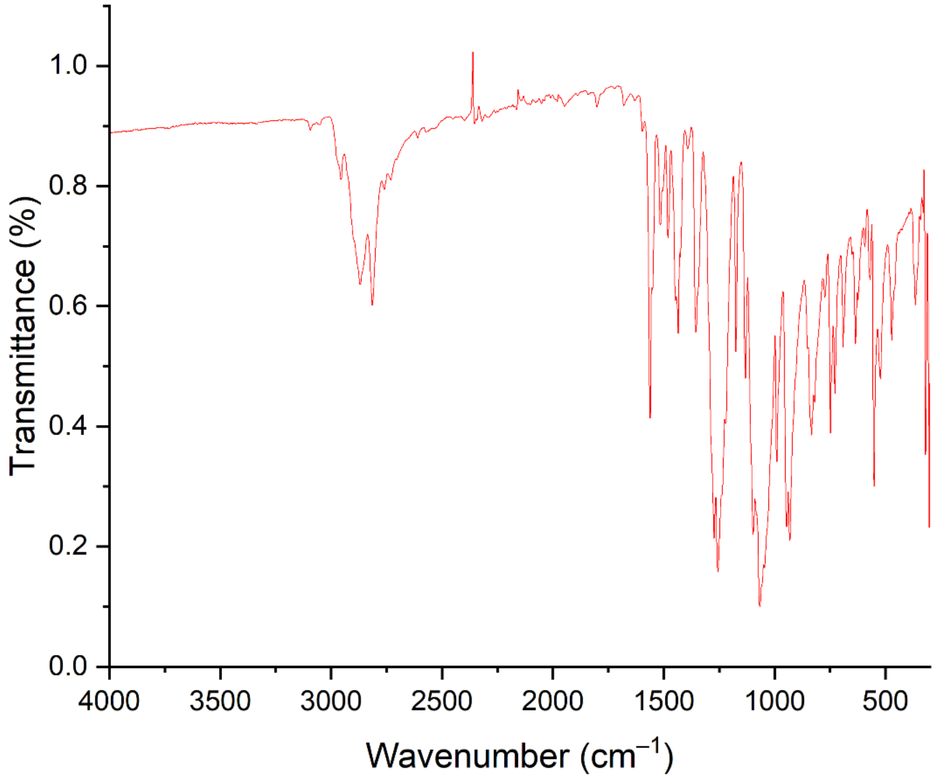

Figure S115. IR spectrum of [K(crypt)]$_2$[**9**] + 4-nitrophenyl disulfide.

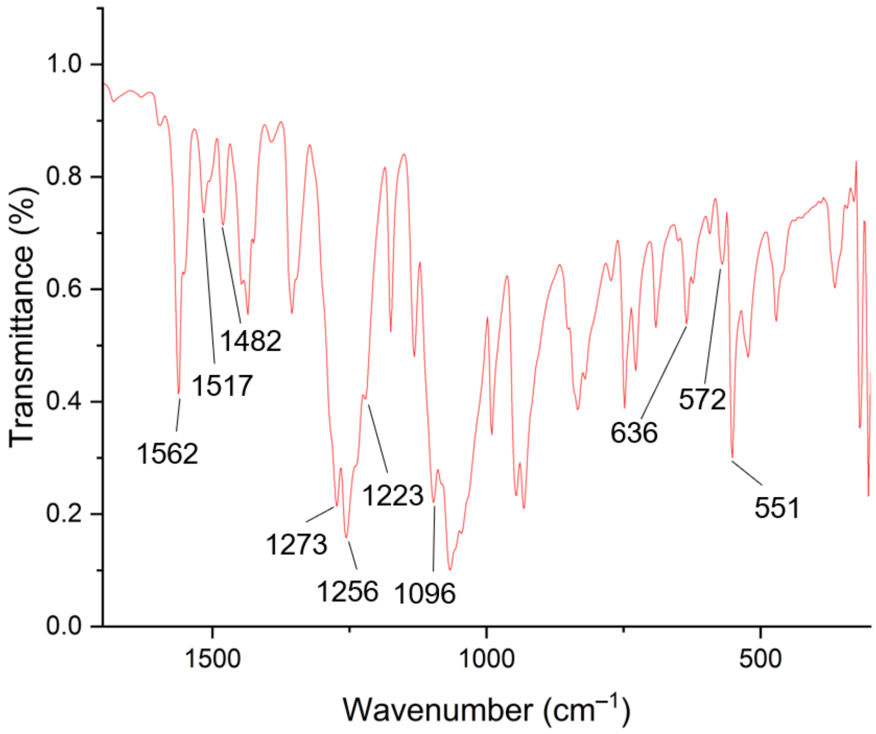

Figure S116. IR spectrum of [K(crypt)]$_2$[**9**] + 4-nitrophenyl disulfide. Detailed.

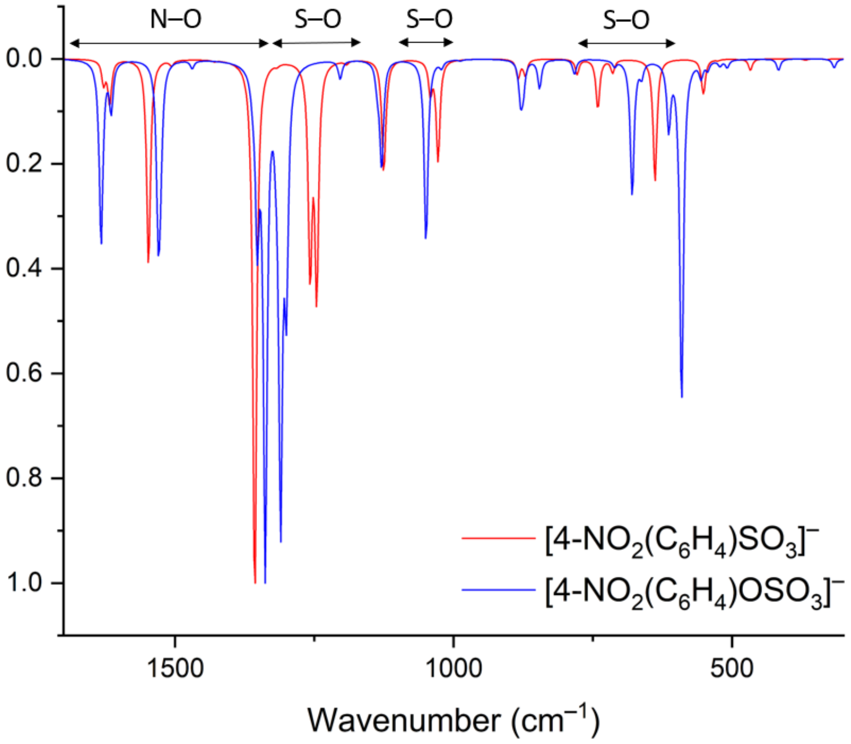

Figure S117. Calculated IR spectrum of $[4\text{-}NO_2(C_6H_4)SO_3]^-$ and $[4\text{-}NO_2(C_6H_4)OSO_3]^-$.

8. Hydroboration of nitro-functionalized compounds

8.1. General Procedure for the Hydroboration of Nitro-functionalized compounds

To a J Young NMR tube, a solution of $[K(18c6)]_3$[**6**] (1 µmol, 0.01 eq.) in DMF (16 µL), a solution of nitroarene (**10a-10j**) (0.1 mmol, 1 eq.) in *o*DFB (0.5 mL) and HBpin (72.5 µL, 0.5 mmol, 5.0 eq.) was added. Toluene (25 µL, 0.24 mmol) was added as an internal standard. The reaction mixture was allowed to react for 24 h at 50 °C. The reaction mixture was monitored by ^{1}H, ^{11}B and ^{11}B{^{1}H} NMR spectroscopy. NMR conv. was determined by integration of the crude ^{1}H NMR spectrum using the toluene as internal standard (^{1}H δ = 2.31 ppm). All volatiles were removed *in vacuo* and the resulting residue was extracted with diethyl ether (3 x 3 mL). The diether ether extract was filtered and to the clear solution 2 M HCl in diethyl ether solution (0.5 mL) was added yielding a precipitate. The precipitate was filtered from the solution and washed with diethyl ether (3 x 3 mL). Recrystallization of the precipitate from a methanol and diethyl ether or a 1-propanol and diethyl ether solution yielded the ammonium chloride salts as a white or off-white solids.

8.2. Characterization Data Hydroboration of Nitroarenes

Annotated NMR spectroscopic and mass spectrometry data below is for the isolated salts except for in the case of **12h**, which could not be isolated. For known products, characterization data was consistent with literature reports and the citations are provided below.

8.2.1. Hydroboration of **10a**.[14, 23]

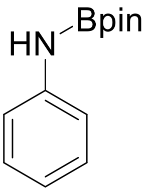

11a

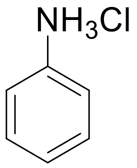

12a

^{1}H NMR (400 MHz, 298 K, CD_3OD): δ = 7.47 – 7.58 (m, 3H, *Ar*), 7.38 – 7.48 (m, 2H, Ar) ppm.

$^{13}C\{^{1}H\}$ NMR (101 MHz, 298 K, CD_3OD): δ = 130.54 (s, *Ar*), 129.91 (s, *Ar*), 128.89 (s, *Ar*), 122.76 (s, *Ar*) ppm.

Mass spectrometry (ESI): C_6H_8N ($[M^+]$): calcd.: 94.0657; found: 94.0648.

NMR conv.: >99%

Isolated Yield: 95%

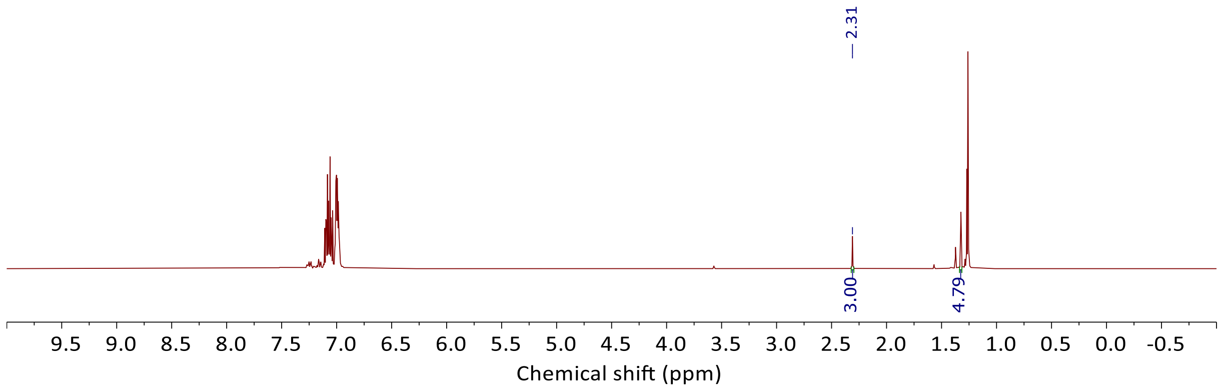

Figure S118. ^{1}H NMR spectrum (400 MHz, *o*DFB) of crude **11a**.

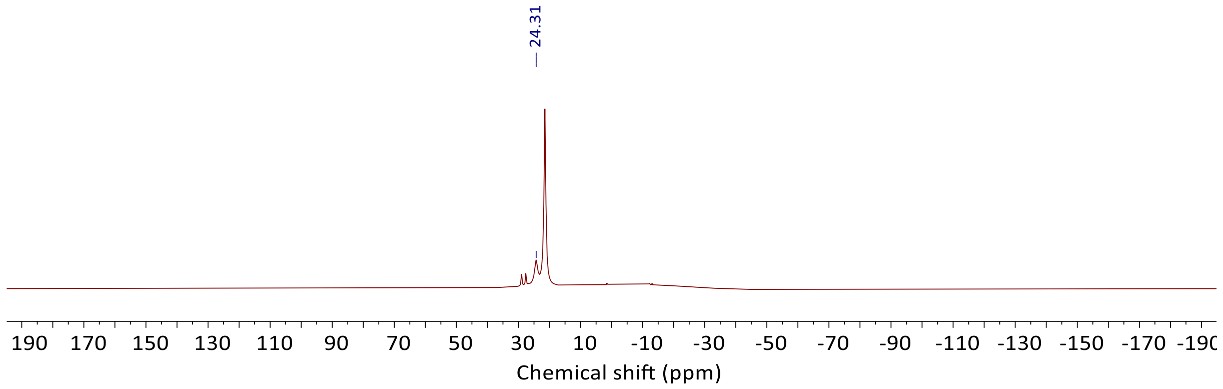

Figure S119. $^{11}B\{^{1}H\}$ NMR spectrum (128 MHz, *o*DFB) of crude **11a**.

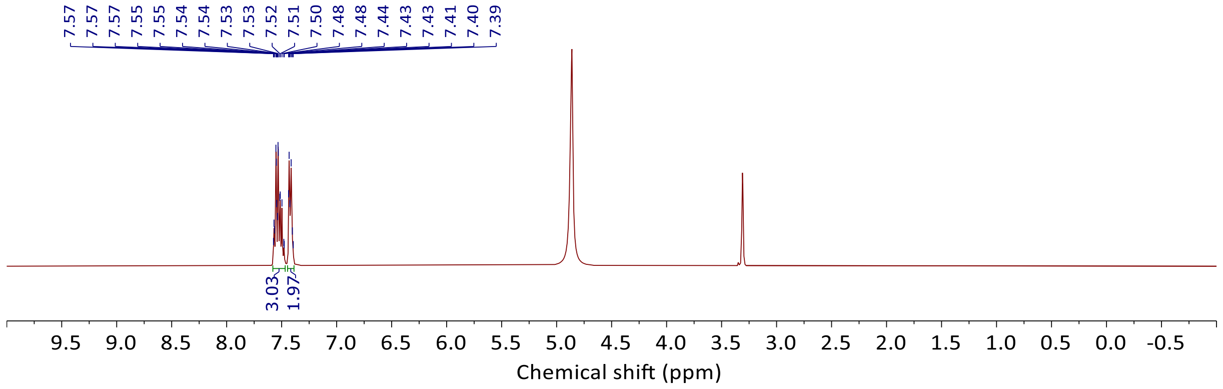

Figure S120. ^{1}H NMR spectrum (400 MHz, CD_3OD) of isolated **12a**.

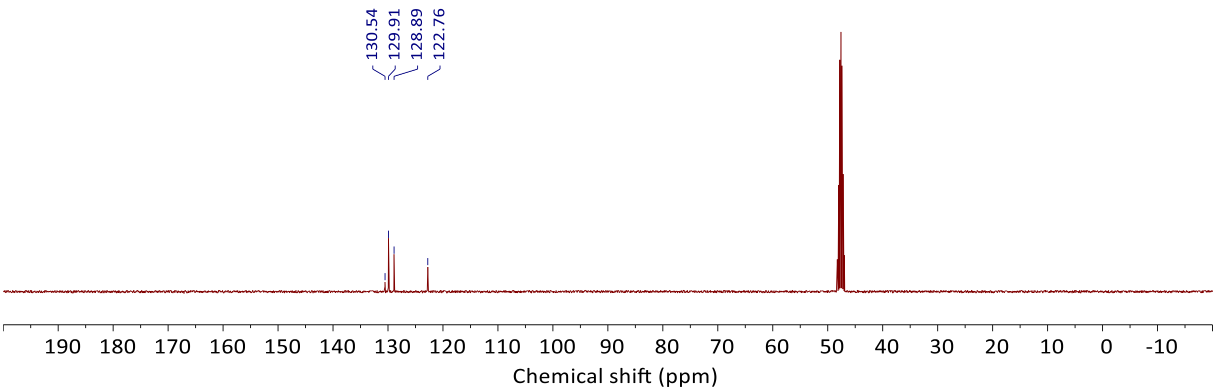

Figure S121. $^{13}C\{^{1}H\}$ NMR spectrum (101 MHz, CD_3OD) of isolated **12a**.

8.2.2. Hydroboration of **10b**.[23]

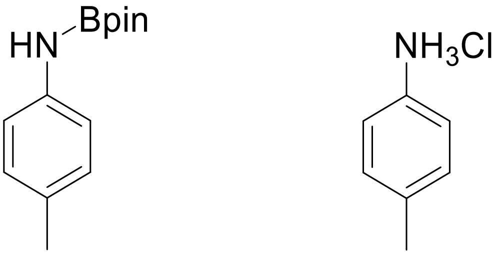

11b **12b**

^{1}H NMR (400 MHz, 298 K, CD_3OD): δ = 7.35 (d, $^{3}J_{HH}$ = 8.3 Hz, 2H, *Ar*), 7.30 (d, $^{3}J_{HH}$ = 8.3 Hz, 2H, *Ar*), 2.39 (s, 3H, Ar–*Me*) ppm.

$^{13}C\{^{1}H\}$ NMR (101 MHz, 298 K, CD_3OD): δ = 139.21 (s, *Ar*), 130.31 (s, *Ar*), 127.87 (s, *Ar*), 122.49 (s, *Ar*), 19.59 (s, Ar–*Me*) ppm.

Mass spectrometry (ESI): $C_7H_{10}N$ ([M^+]): calcd.: 108.0808; found: 108.0808

NMR conv.: 94%

Isolated Yield: 90%

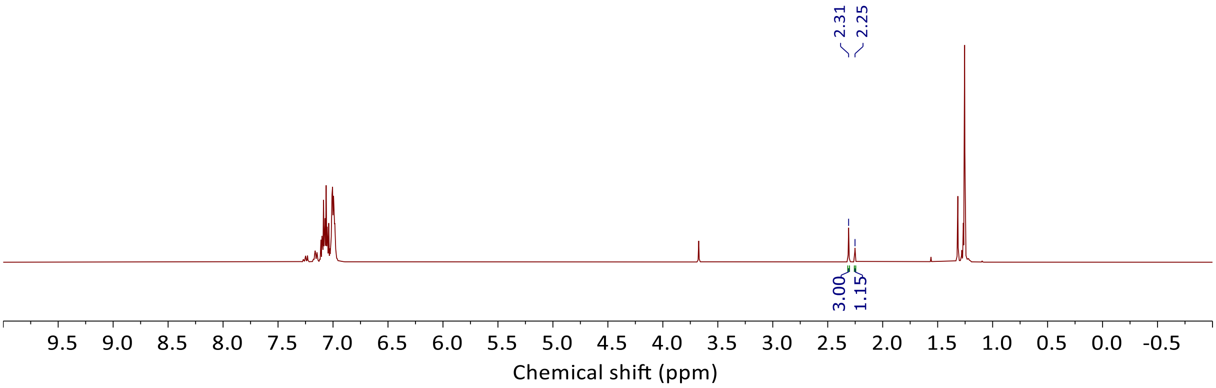

Figure S122. ^{1}H NMR spectrum (400 MHz, *o*DFB) of crude **11b**.

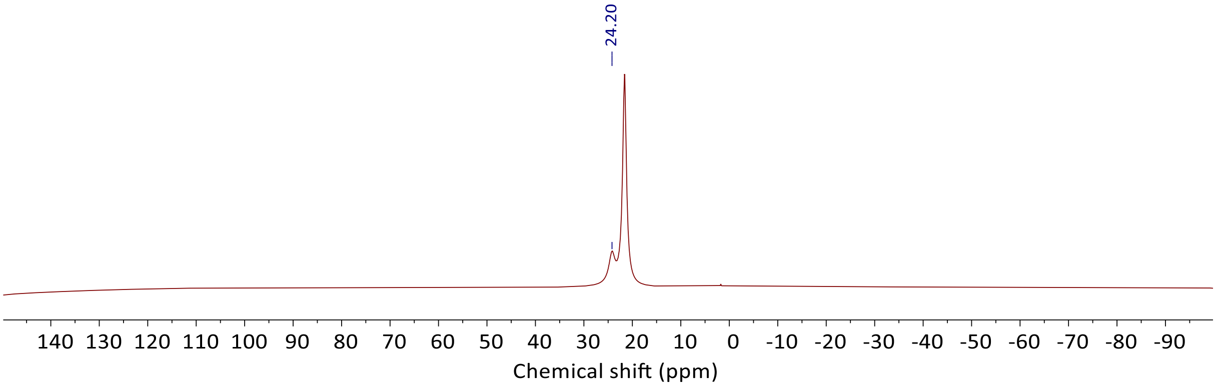

Figure S123. $^{11}B\{^{1}H\}$ NMR spectrum (128 MHz, *o*DFB) of crude **11b**.

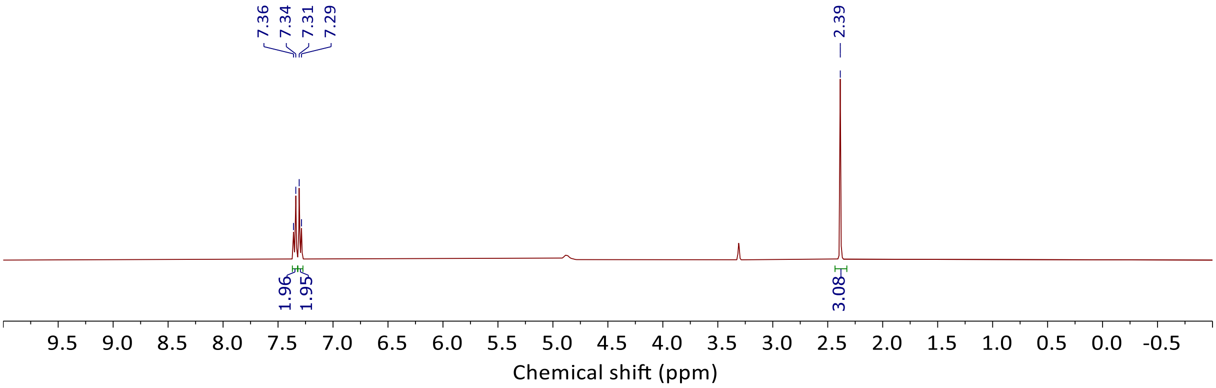

Figure S124. ^{1}H NMR spectrum (400 MHz, CD_3OD) of isolated **12b**.

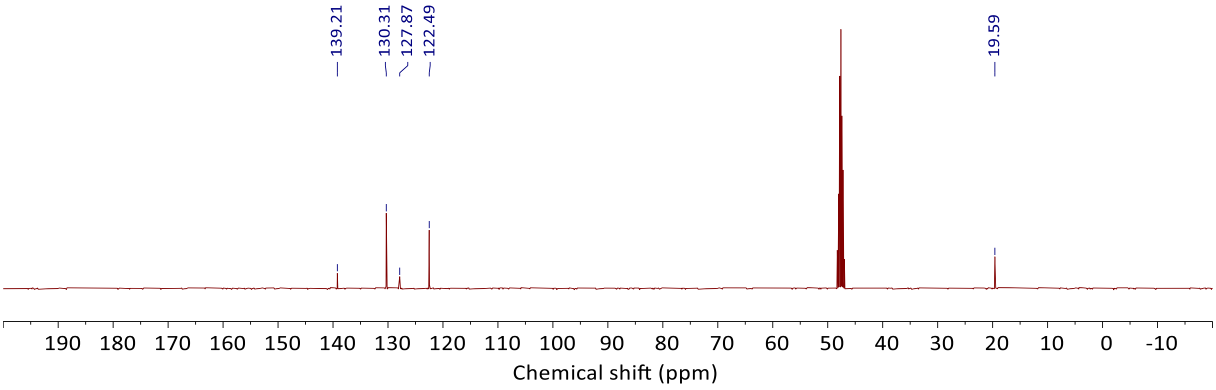

Figure S125. $^{13}C\{^{1}H\}$ NMR spectrum (101 MHz, CD_3OD) of isolated **12b**.

8.2.3. Hydroboration of **10c**.[23]

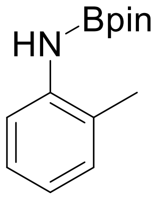

11c

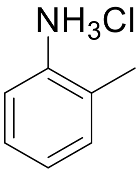

12c

^{1}H NMR (400 MHz, 298 K, CD_3OD): δ = 7.32 – 7.46 (m, 4H, *Ar*), 2.43 (s, 3H, Ar–*Me*) ppm.

^{13}C{^{1}H} NMR (101 MHz, 298 K, CD_3OD): δ = 131.83 (s, *Ar*), 131.57 (s, *Ar*), 129.25 (s, *Ar*), 129.07 (s, *Ar*), 127.29 (s, *Ar*), 122.80 (s, *Ar*), 15.60 (s, Ar–*Me*) ppm.

Mass spectrometry (ESI): $C_7H_{10}N$ ([M$^+$]): calcd.: 108.0808; found: 108.0838

NMR conv.: 95%

Isolated Yield: 90%

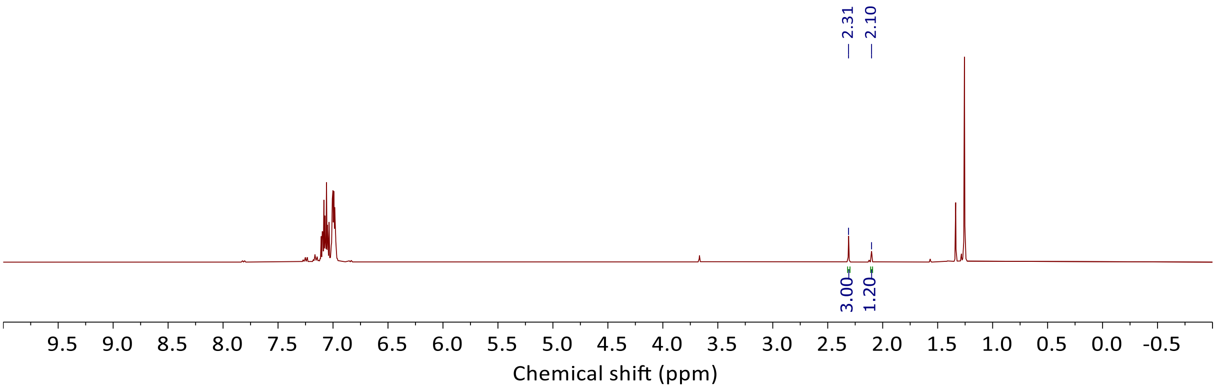

Figure S126. ^{1}H NMR spectrum (400 MHz, *o*DFB) of crude **11c**.

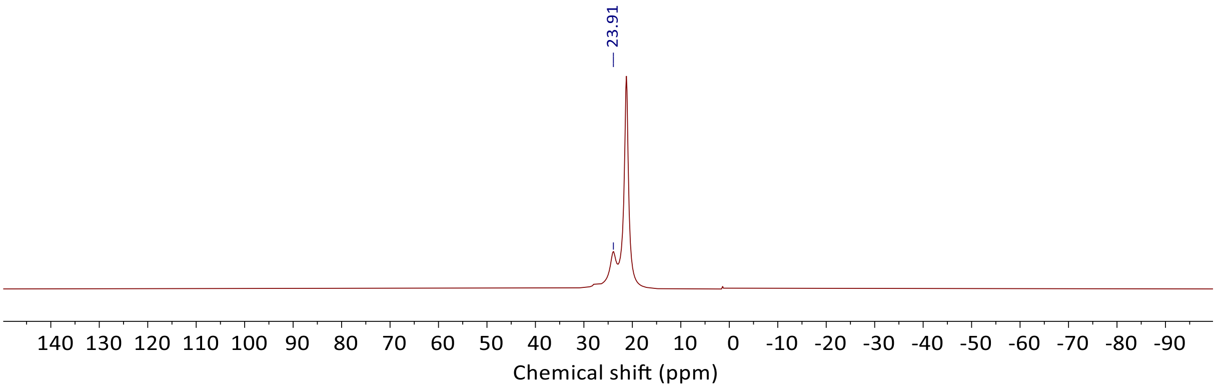

Figure S127. ^{11}B{^{1}H} NMR spectrum (128 MHz, *o*DFB) of crude **11c**.

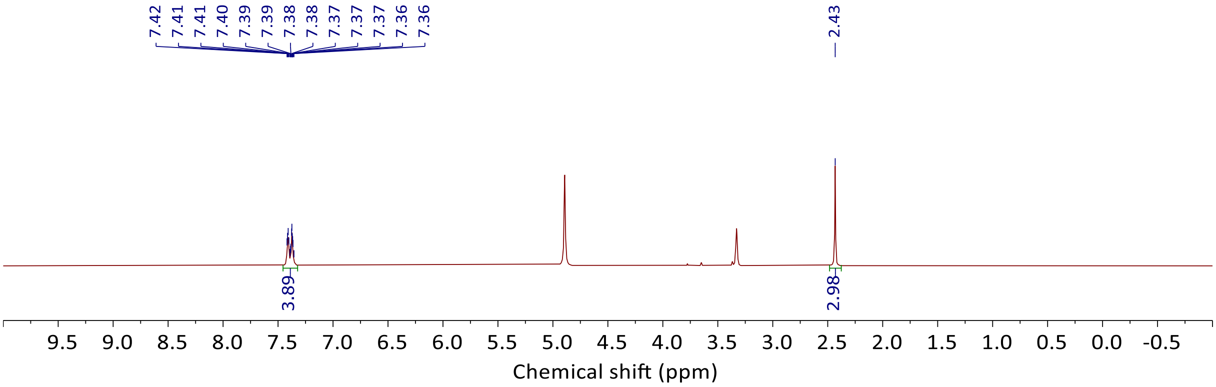

Figure S128. ^{1}H NMR spectrum (400 MHz, CD_3OD) of isolated **12c**.

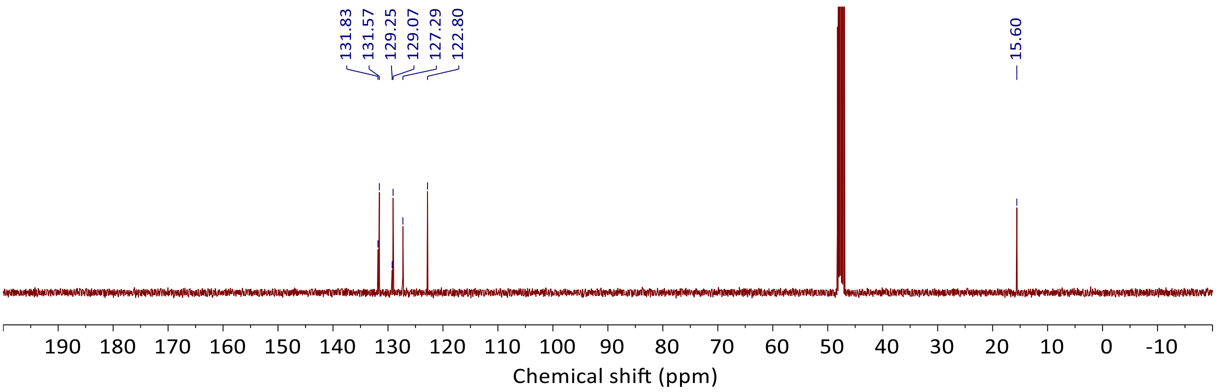

Figure S129. $^{13}C\{^1H\}$ NMR spectrum (101 MHz, CD_3OD) of isolated **12c**.

8.2.4. Hydroboration of **10d**.

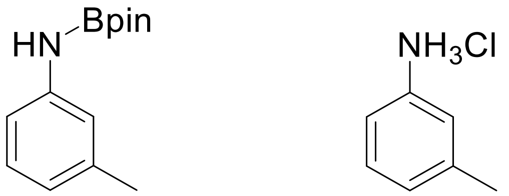

11d **12d**

^{1}H NMR (400 MHz, 298 K, CD_3OD): δ = 7.43 (t, $^3J_{HH}$ = 7.7 Hz, 1H), 7.33 (d, $^3J_{HH}$ = 7.7 Hz, 1H, *Ar*), 7.26 – 7.20 (m, 1H, *Ar*), 2.43 (s, 3H, Ar–*Me*) ppm.

$^{13}C\{^1H\}$ NMR (101 MHz, 298 K, CD_3OD): δ = 140.57 (s, *Ar*), 130.41 (s, *Ar*), 129.70 (s, *Ar*), 129.51 (s, *Ar*), 129.11 (s, *Ar*), 119.66 (s, *Ar*), 19.85 (s, Ar–*Me*) ppm.

Mass spectrometry (ESI): $C_7H_{10}N$ ([M$^+$]): calcd.: 108.0808; found: 108.0840

NMR conv.: >99%

Isolated Yield: 92%

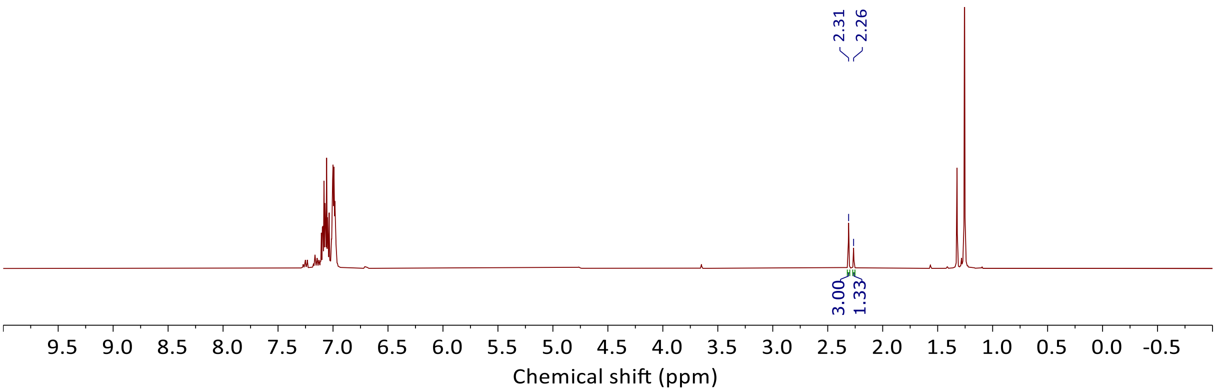

Figure S130. ^{1}H NMR spectrum (400 MHz, *o*DFB) of crude **11d**.

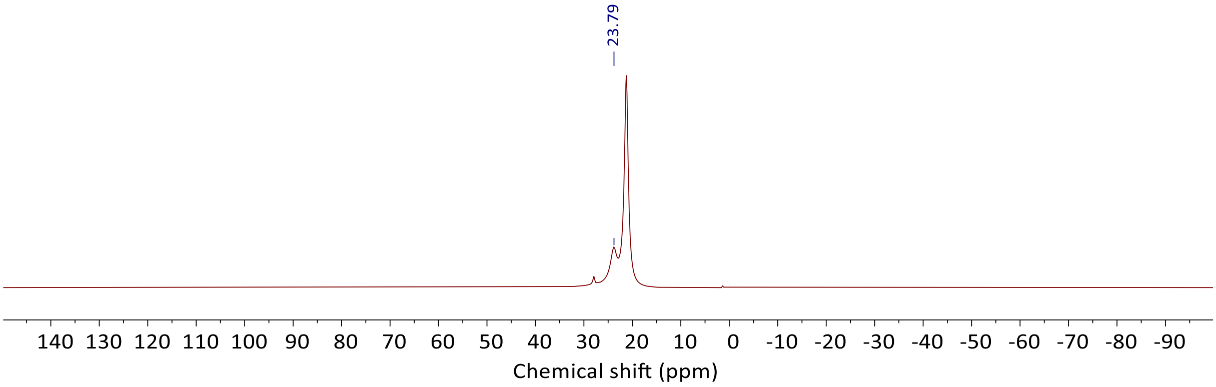

Figure S131. $^{11}B\{^1H\}$ NMR spectrum (128 MHz, *o*DFB) of crude **11d**.

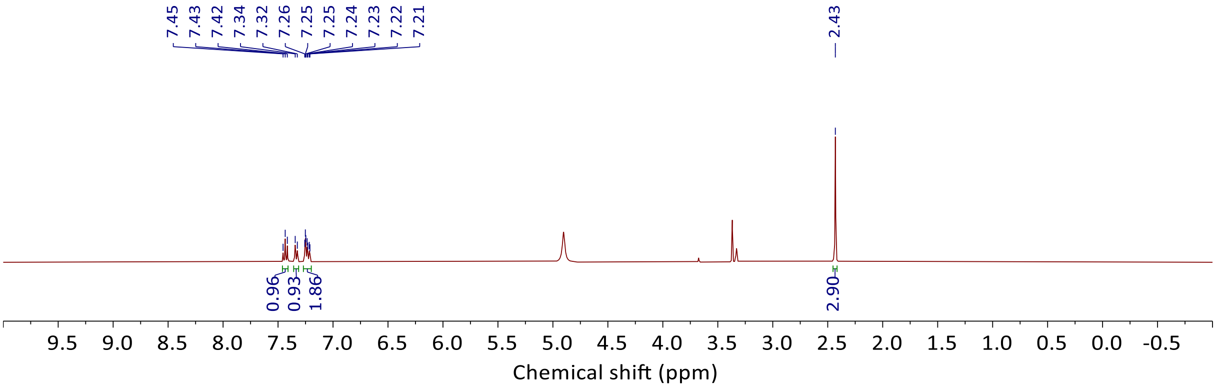

Figure S132. ^1H NMR spectrum (400 MHz, CD_3OD) of isolated **12d**.

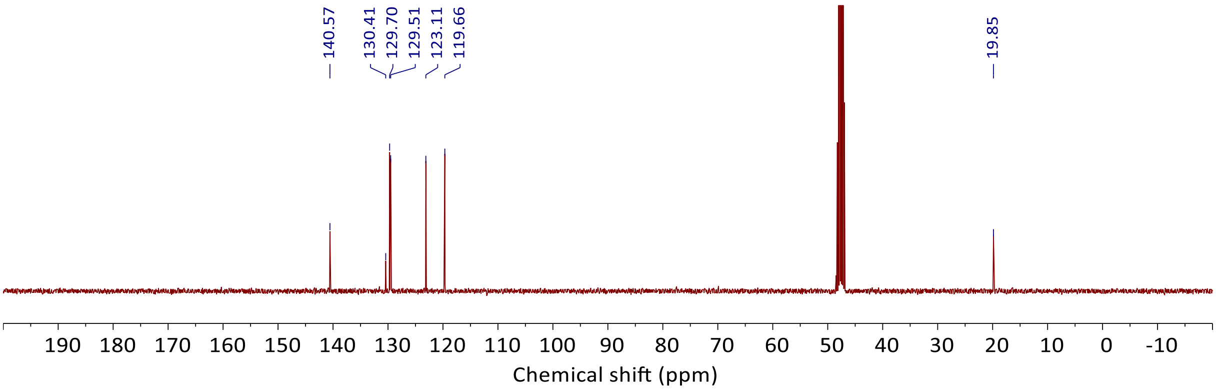

Figure S133. $^{13}C\{^1H\}$ NMR spectrum (101 MHz, CD_3OD) of isolated **12d**.

8.2.5. Hydroboration of **10e**.[14]

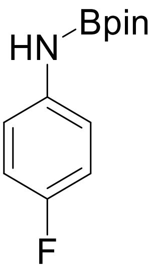

11e

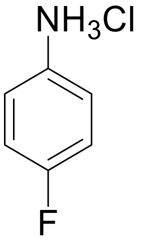

12e

^{1}H NMR (400 MHz, 298 K, CD_3OD): δ = 7.47 (dd, $^{3}J_{HH}$ = 8.6 Hz, $^{4}J_{HF}$ = 4.4 Hz, 2H, *Ar*), 7.30 (dd, $^{3}J_{HH}$ = 8.6 Hz, $^{3}J_{HF}$ = 8.6 Hz, 2H, *Ar*), ppm.

^{13}C{^{1}H} NMR (101 MHz, 298 K, CD_3OD): δ = 162.55 (d, $^{1}J_{CF}$ = 247.5 Hz, *Ar*), 126.68 (s, *Ar*), 124.96 (d, $^{3}J_{CF}$ = 9.2 Hz, *Ar*), 116.69 (d, $^{2}J_{CF}$ = 23.0 Hz, *Ar*) ppm.

^{19}F{^{1}H} NMR (377 MHz, 298 K, CD_3OD): δ = –114.32 (s) ppm.

Mass spectrometry (ESI): C_6H_7FN ([M^{+}]): calcd.: 112.0557; found: 112.0548.

NMR conv.: >99%

Isolated Yield: 91%

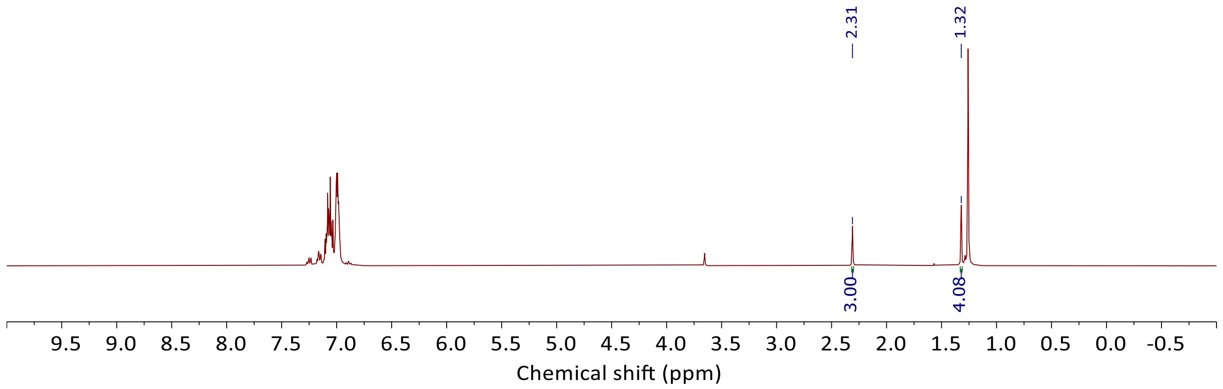

Figure S134. ^{1}H NMR spectrum (400 MHz, *o*DFB) of crude **11e**.

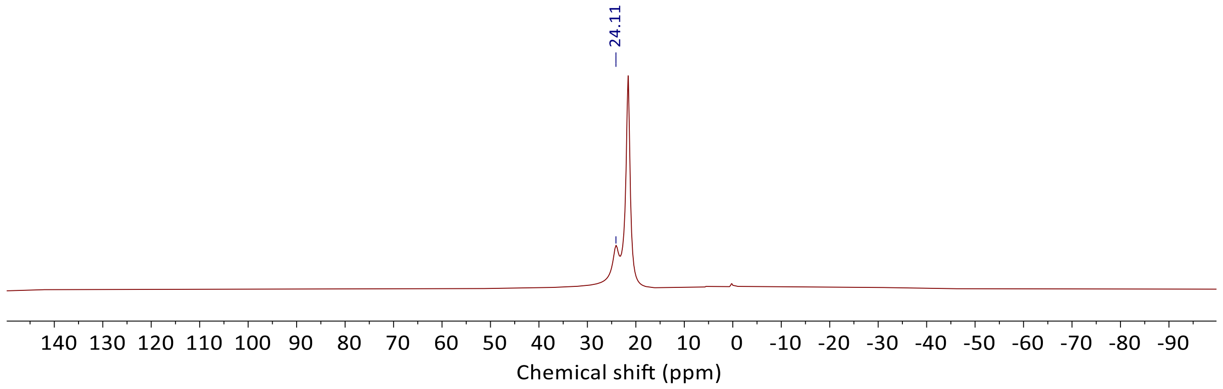

Figure S135. ^{11}B{^{1}H} NMR spectrum (128 MHz, *o*DFB) of crude **11e**.

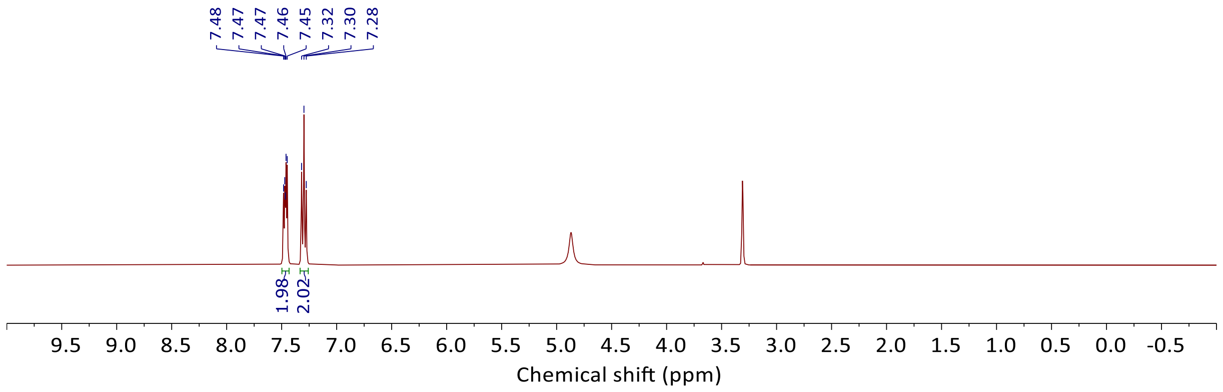

Figure S136. ^{1}H NMR spectrum (400 MHz, CD_3OD) of isolated **12e**.

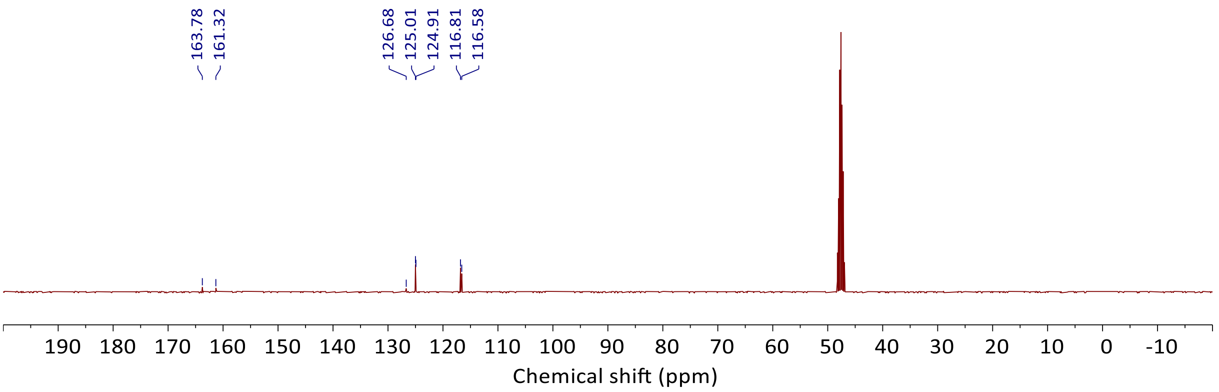

Figure S137. $^{13}C\{^1H\}$ NMR spectrum (101 MHz, CD_3OD) of isolated **12e**.

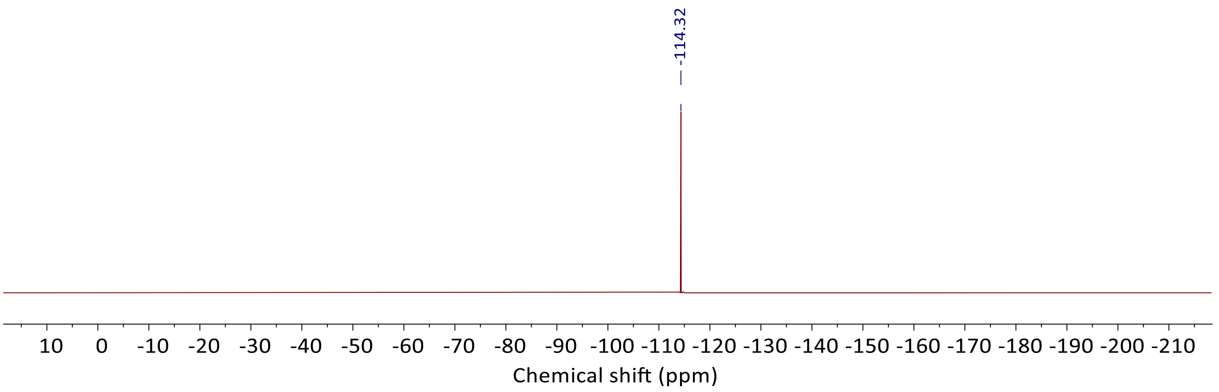

Figure S138. $^{19}F\{^1H\}$ NMR spectrum (377 MHz, CD_3OD) of isolated **12e**.

8.2.6. Hydroboration of **10f.**[23]

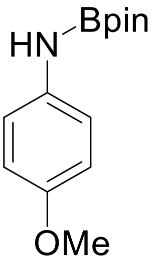

11f

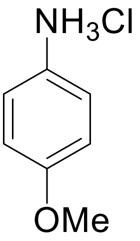

12f

^{1}H NMR (400 MHz, 298 K, CD_3OD): δ = 7.34 (d, $^3J_{HH}$ = 9.0 Hz, 2H, *Ar*), 7.06 (d, $^3J_{HH}$ = 9.0 Hz, 2H, *Ar*), 3.83 (s, 3H, Ar–O*Me*) ppm.

$^{13}C\{^1H\}$ NMR (101 MHz, 298 K, CD_3OD): δ = 160.07 (s, *Ar*), 123.83 (s, *Ar*), 122.87 (s, *Ar*), 114.92 (s, *Ar*), 3.83 (s, Ar–O*Me*) ppm.

Mass spectrometry (ESI): $C_7H_{10}NO$ ([M$^+$]): calcd.: 124.0757; found: 124.0757

NMR conv.: 88%

Isolated Yield: 82%

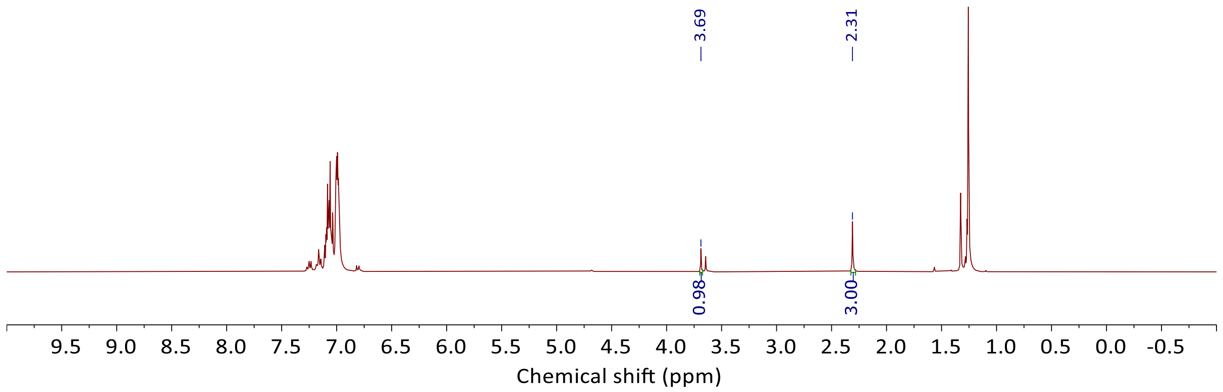

Figure S139. ^{1}H NMR spectrum (400 MHz, *o*DFB) of crude **11f**.

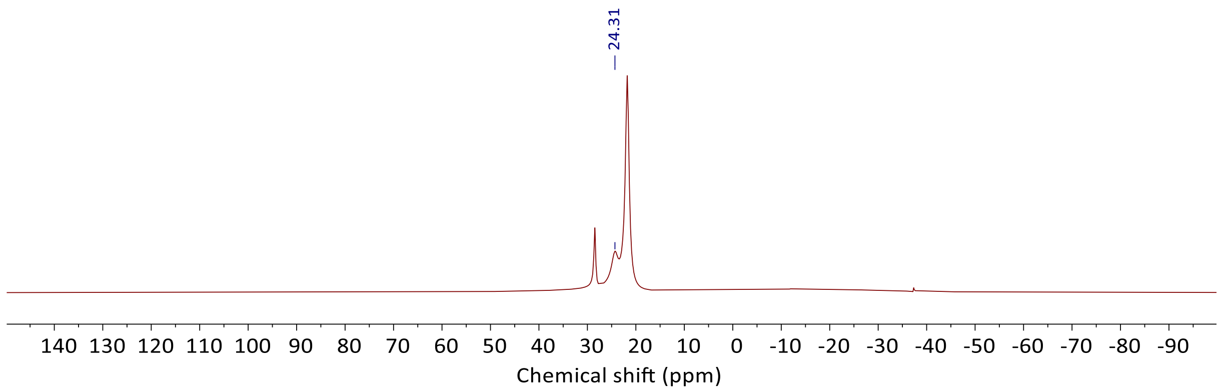

Figure S140. ^{11}B{^{1}H} NMR spectrum (128 MHz, *o*DFB) of crude **11f**.

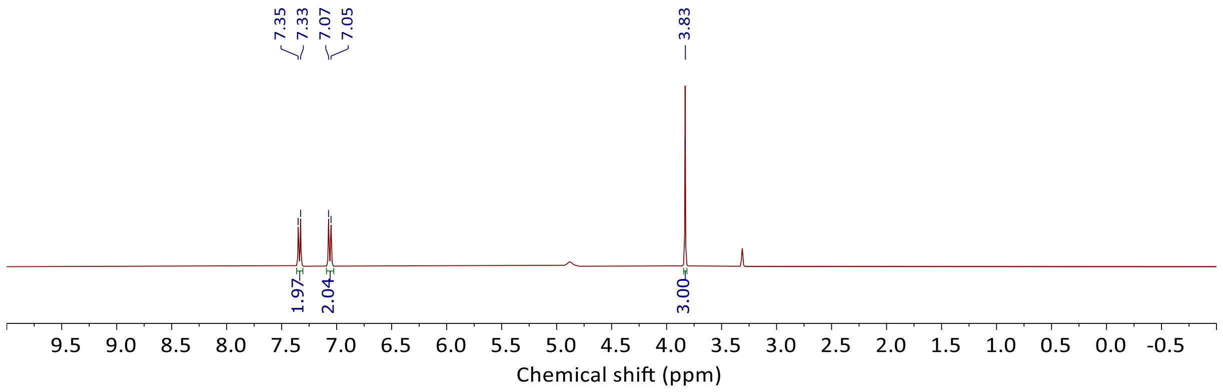

Figure S141. ^{1}H NMR spectrum (400 MHz, CD_3OD) of isolated **12f**.

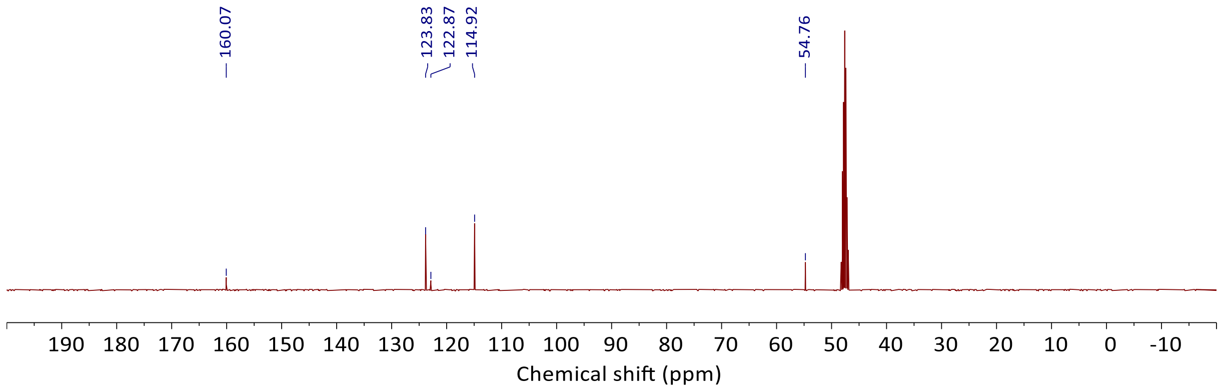

Figure S142. ^{13}C{^{1}H} NMR spectrum (101 MHz, CD_3OD) of isolated **12f**.

8.2.7. Hydroboration of **10g**.[14]

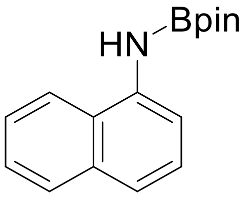

11g

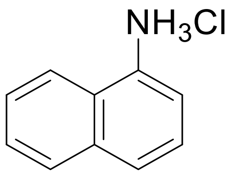

12g

^{1}H NMR (400 MHz, 298 K, CD_3OD): δ = 8.02 – 8.09 (m, 3H, *Ar*), 7.65 – 7.79 (m, 3H, *Ar*), 7.58 - 7.62 (m, 1H, *Ar*) ppm.

^{13}C{^{1}H} NMR (101 MHz, 298 K, CD_3OD): δ = 134.28 (s, *Ar*), 129.41 (s, *Ar*), 128.59 (s, *Ar*), 127.59 (s, *Ar*), 127.13 (s, *Ar*), 126.64 (s, *Ar*), 126.42 (s, *Ar*), 124.97 (s, *Ar*), 124.83 (s, *Ar*), 120.28 (s, *Ar*) ppm.

Mass spectrometry (ESI): $C_{10}H_{10}N$ ([M^+]): calcd.: 144.0808; found: 144.0802

NMR conv.: 94%

Isolated Yield: 75%

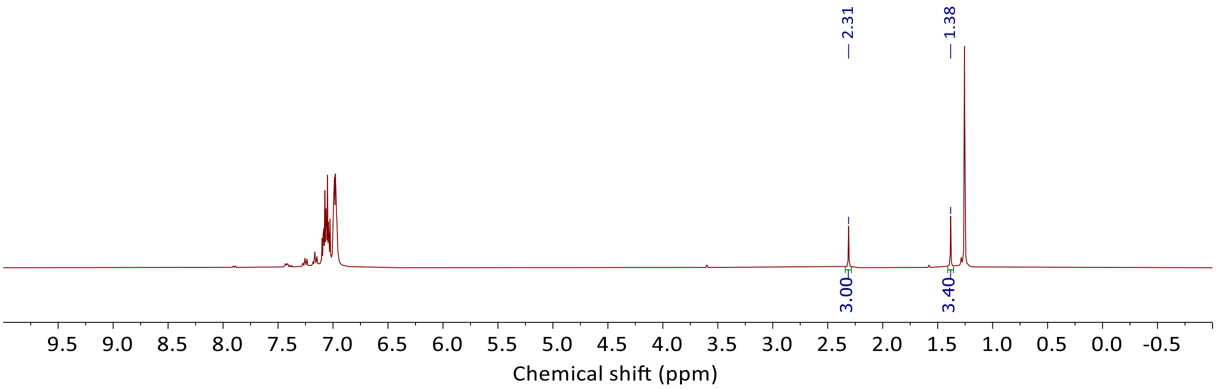

Figure S143. ^{1}H NMR spectrum (400 MHz, *o*DFB) of crude **11g**.

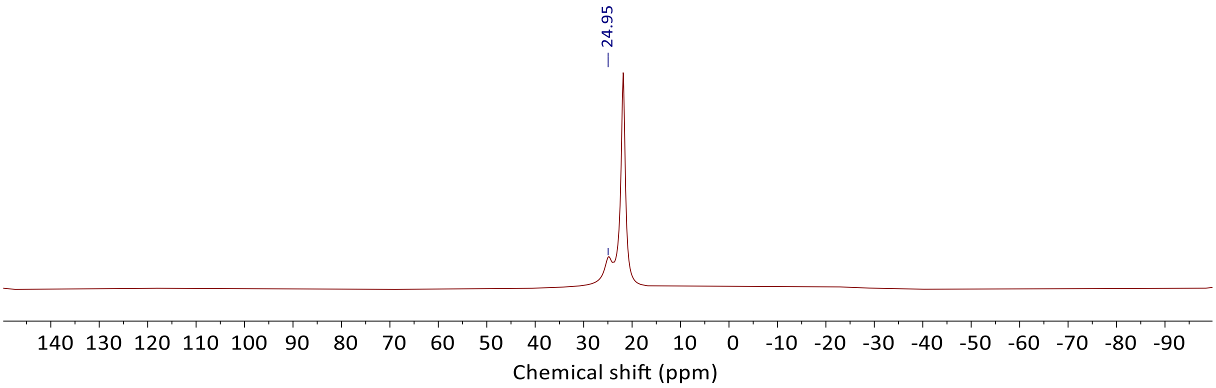

Figure S144. ^{11}B{^{1}H} NMR spectrum (128 MHz, *o*DFB) of crude **11g**.

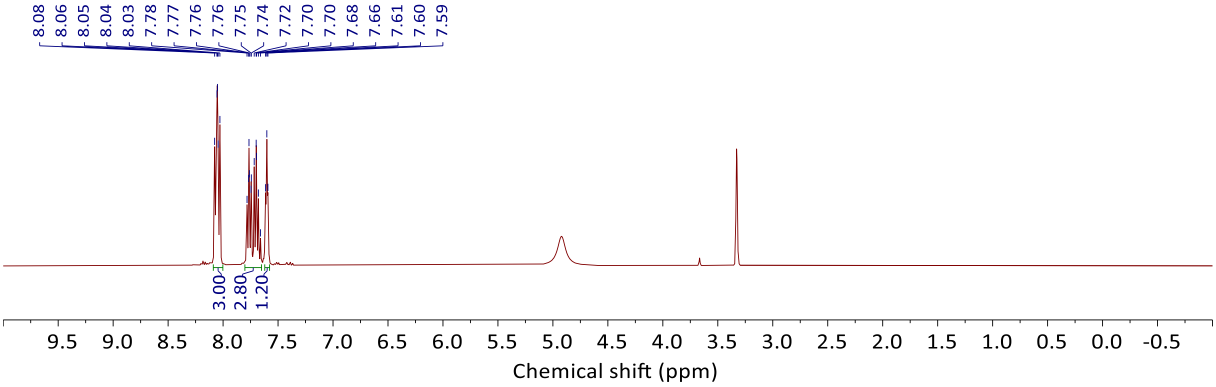

Figure S145. ^{1}H NMR spectrum (400 MHz, CD_3OD) of isolated **12g**.

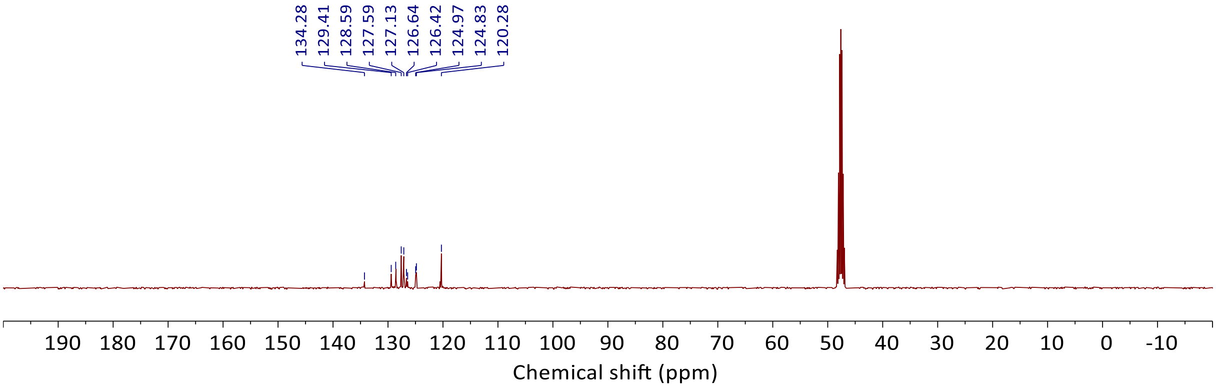

Figure S146. ^{13}C{^{1}H} NMR spectrum (101 MHz, CD_3OD) of isolated **12g**.

8.2.8. Hydroboration of **10h**.

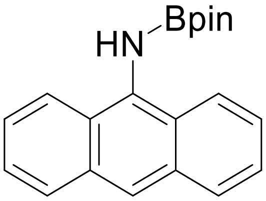

11h

^{1}H NMR (400 MHz, 298 K, reaction mixture): δ = 8.53 (d, $^{3}J_{HH}$ = 8.8 Hz, 2H, *Ar*), 8.10 (s, 2H, *Ar*), 7.86 (d, $^{3}J_{HH}$ = 8.4 Hz, 2H, *Ar*), 7.56 (t, $^{3}J_{HH}$ = 8.8 Hz, 2H, *Ar*), 7.43 (t, $^{3}J_{HH}$ = 8.2 Hz, 2H, *Ar*), 1.27 (s, 12H, B*pin*) ppm.

^{11}B NMR (128 MHz, 298 K, reaction mixture): δ = 25.26 (s) ppm.

Mass spectrometry (ESI): $C_{14}H_{11}N$ ([M^{+}]): calcd.: 194.0964; found: 194.0961

NMR conv.: 58%

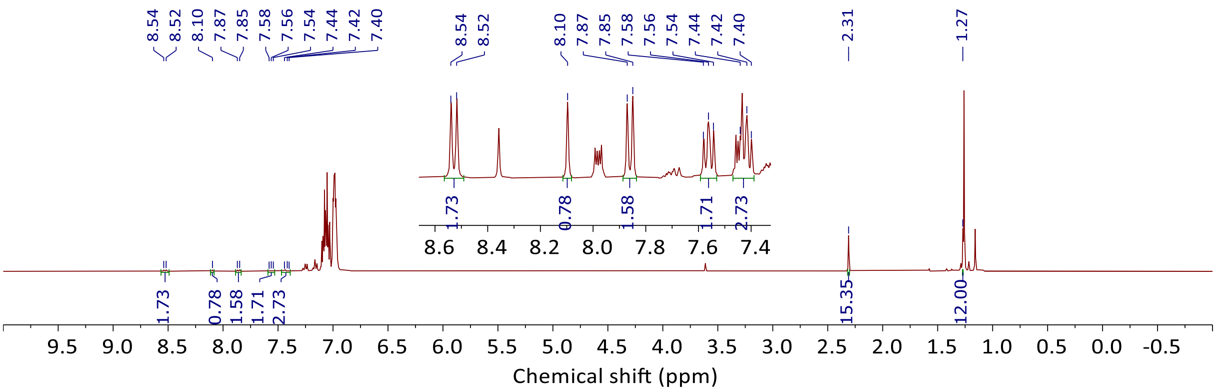

Figure S147. ^{1}H NMR spectrum (400 MHz, *o*DFB) of crude **11h**.

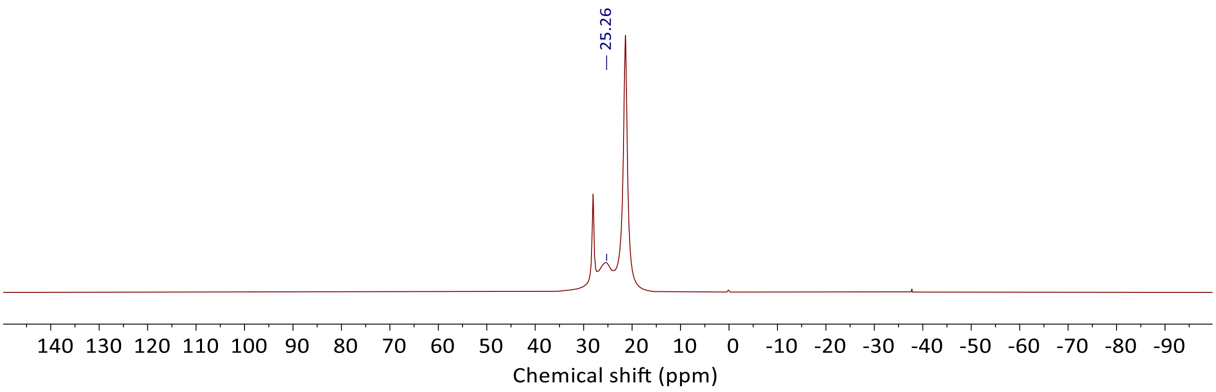

Figure S148. $^{11}B\{^{1}H\}$ NMR spectrum (128 MHz, *o*DFB) of crude **11h**.

8.2.9. Hydroboration of **10i**.

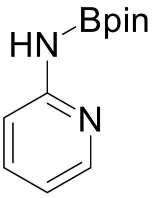

11i

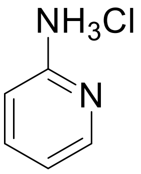

12i

^{1}H NMR (400 MHz, 298 K, CD_3OD): δ = 7.85 (ddd, $^{3}J_{HH}$ = 9.0 Hz, 7.0 Hz, $^{4}J_{HH}$ = 1.6 Hz, 1H, *Ar*), 7.80 (ddd, $^{3}J_{HH}$ = 6.5, $^{4}J_{HH}$ = 1.5 Hz, 0.8 Hz, 1H, *Ar*), 6.91 (ddd, $^{3}J_{HH}$ = 9.2, $^{4}J_{HH}$ = 1.0 Hz, 1.0 Hz, 1H, *Ar*), 6.86 (ddd, $^{3}J_{HH}$ = 6.7 Hz, 6.7 Hz, $^{4}J_{HH}$ = 1.1 Hz, 1H, *Ar*) ppm.

$^{13}C\{^{1}H\}$ NMR (101 MHz, 298 K, CD_3OD): δ = 154.02 (s, *Ar*), 143.02 (s, *Ar*), 134.58 (s, *Ar*), 113.38 (s, *Ar*), 110.24 (s, *Ar*) ppm.

Mass spectrometry (ESI): $C_7H_{10}N$ ([M^+]): calcd.: 95.0604; found: 95.0605

NMR conv.: 76%

Isolated Yield: 68%

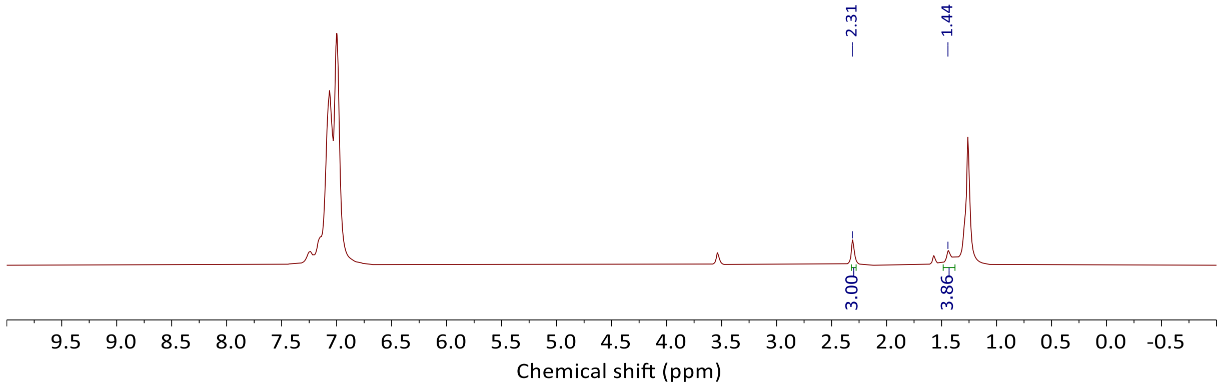

Figure S149. ^{1}H NMR spectrum (400 MHz, *o*DFB) of crude **11i**.

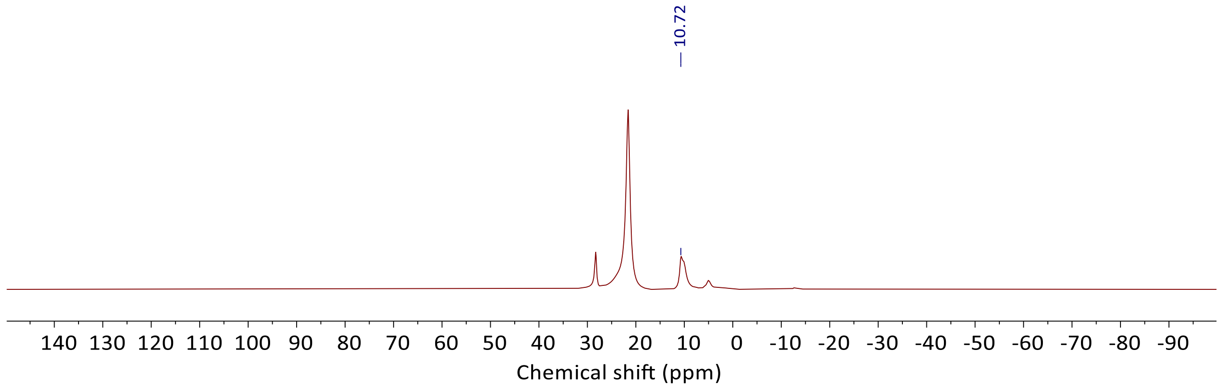

Figure S150. ^{11}B{^{1}H} NMR spectrum (128 MHz, *o*DFB) of crude **11i**.

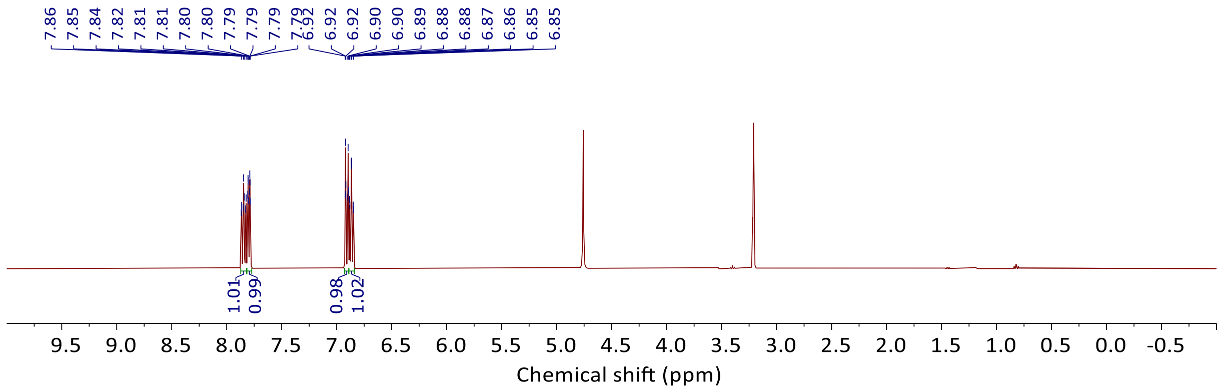

Figure S151. ^{1}H NMR spectrum (400 MHz, CD_3OD) of isolated **12i**.

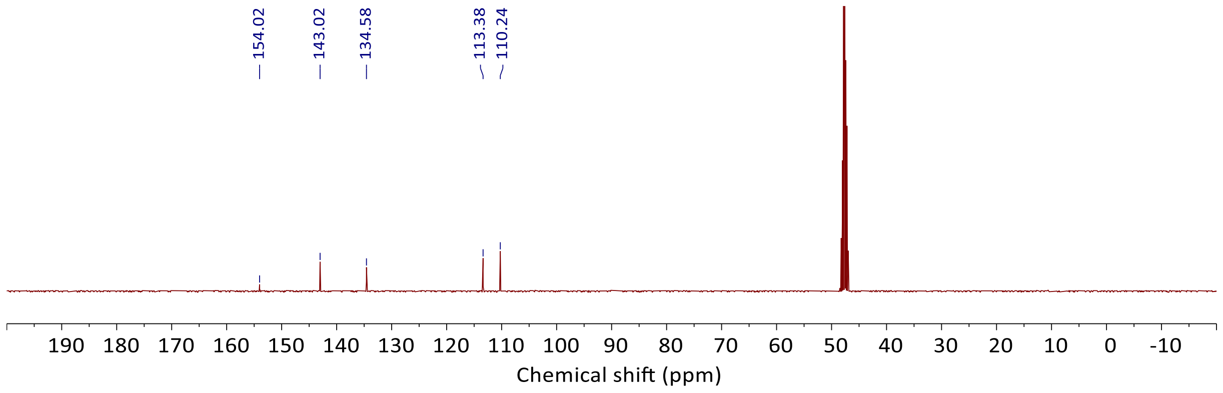

Figure S152. ^{13}C{^{1}H} NMR spectrum (101 MHz, CD_3OD) of isolated **12i**.

8.2.10. Hydroboration of **10j**.[14]

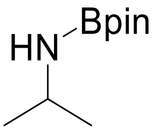

11j

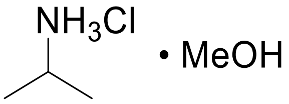

12j

^{1}H NMR (400 MHz, 298 K, CD_3OD): δ = 3.43 (hept, $^3J_{HH}$ = 6.6 Hz, 1H, Me_2CHN), 1.36 (s, 3H, *Me*OH), 1.32 (d, $^3J_{HH}$ = 6.6 Hz, 3H, *Me*) ppm.

^{13}C{^{1}H} NMR (101 MHz, 298 K, CD_3OD): δ = 48.45 (s, *Me*OH), 43.75 (s, Me_2CHN), 19.46 (s, *Me*), 43.75 (s, Me_2CHN) ppm.

Mass spectrometry (ESI): $C_7H_{10}N$ ([M^+]): calcd.: 60.0808; found: 60.0801

NMR conv.: 83%

Isolated Yield: 74%

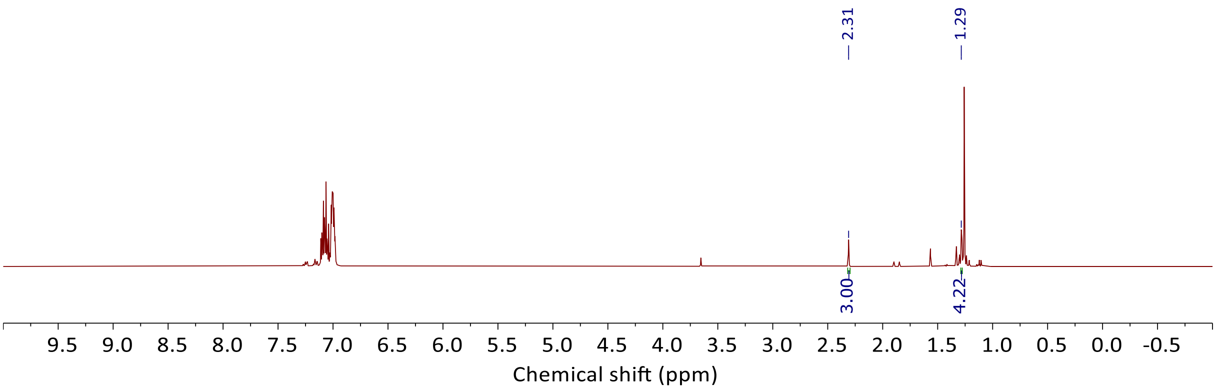

Figure S153. ^{1}H NMR spectrum (400 MHz, *o*DFB) of crude **11j**.

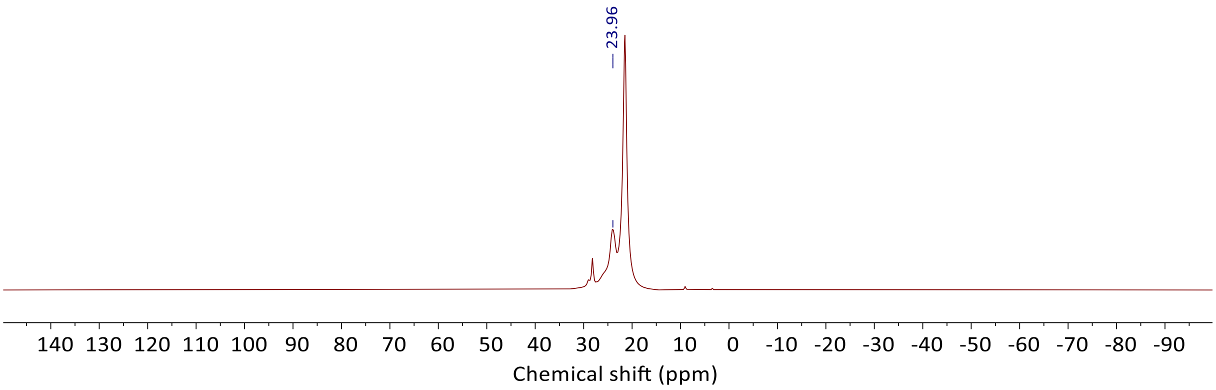

Figure S154. ^{11}B{^{1}H} NMR spectrum (128 MHz, *o*DFB) of crude **11j**.

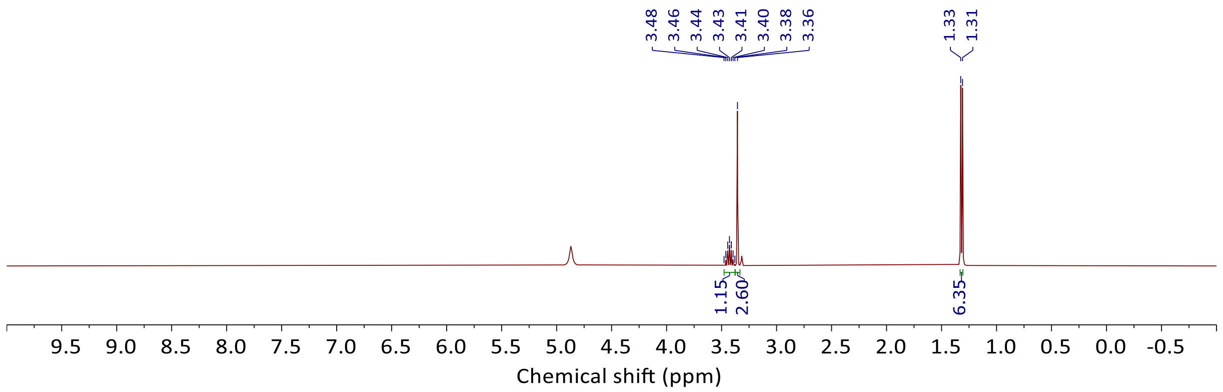

Figure S155. ^{1}H NMR spectrum (400 MHz, CD_3OD) of isolated **12j**.

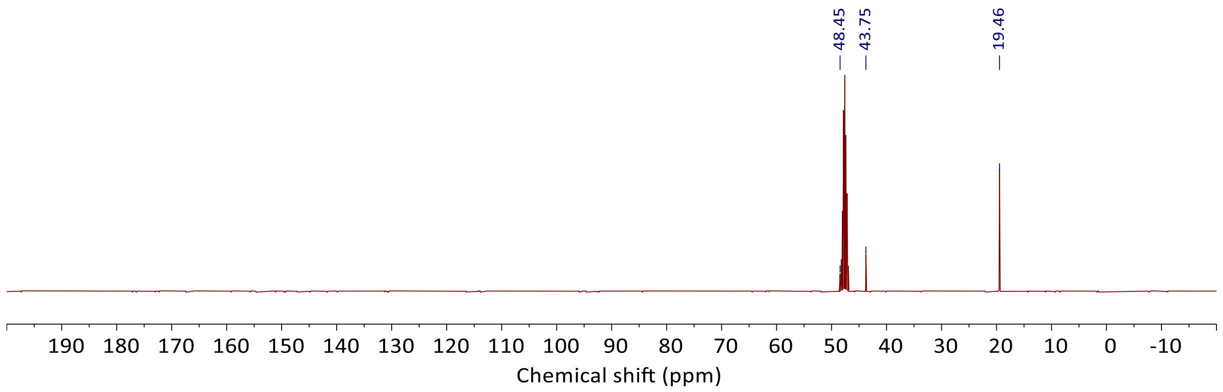

Figure S156. ^{13}C{^{1}H} NMR spectrum (101 MHz, CD_3OD) of isolated **12j**.

9. Crystallography Tables

Identification code	$[K(18c6)]_3[P_7]$	$[K(18c6)]_3[As_7]$
Empirical formula	$C_{41}H_{77}K_3NO_{18}P_7$	$C_{36}H_{72}As_7K_3O_{18}$
Formula weight	1206.12	1434.67
Temperature/K	150.15	150.00(10)
Crystal system	triclinic	orthorhombic
Space group	P-1	$Pna2_1$
a/Å	13.5879(2)	27.0296(3)
b/Å	13.9644(2)	16.27800(10)
c/Å	15.9476(2)	15.94070(10)
α/°	88.0860(10)	90
β/°	83.8280(10)	90
γ/°	84.1290(10)	90
Volume/Å^3	2991.92(7)	7013.71(10)
Z	2	4
ρ_{calc}g/cm^3	1.339	1.359
μ/mm^{-1}	4.328	5.816
F(000)	1272.0	2880.0
Crystal size/mm^3	0.4 × 0.268 × 0.194	0.411 × 0.32 × 0.207
Radiation	CuKα (λ = 1.54184)	Cu Kα (λ = 1.54184)
2Θ range for data collection/°	6.364 to 152.428	7.762 to 162.2
Index ranges	-16 ≤ h ≤ 17, -17 ≤ k ≤ 16, -20 ≤ l ≤ 20	-33 ≤ h ≤ 33, -20 ≤ k ≤ 19, -16 ≤ l ≤ 20
Reflections collected	67781	82609
Independent reflections	12376 [R_{int} = 0.0310, R_{sigma} = 0.0165]	12209 [R_{int} = 0.0458, R_{sigma} = 0.0283]
Data/restraints/parameters	12376/216/822	12209/1/642
Goodness-of-fit on F^2	1.031	1.035
Final R indexes [I>=2σ (I)]	R_1 = 0.0338, wR_2 = 0.0930	R_1 = 0.0327, wR_2 = 0.0860
Final R indexes [all data]	R_1 = 0.0350, wR_2 = 0.0942	R_1 = 0.0356, wR_2 = 0.0883
Largest diff. peak/hole / e Å^{-3}	$C_{41}H_{77}K_3NO_{18}P_7$	0.64/-0.43
CCDC	2434059	2434060

Identification code	$[K(crypt)]_2[As_4O_7]$	$[K(crypt)]_2[S_{6.4}O_{5.2}]$
Empirical formula	$C_{39}H_{79}As_4K_2N_5O_{20}$	$C_{42}H_{86}K_2N_6O_{19.19}S_{6.42}$
Formula weight	1315.47	1266.39
Temperature/K	100.15(10)	150.15
Crystal system	monoclinic	triclinic
Space group	$P2_1$	P-1
a/Å	12.15440(10)	12.8621(3)
b/Å	21.0341(2)	13.2954(3)
c/Å	12.39970(10)	20.3883(6)
α/°	90	102.296(2)
β/°	118.0560(10)	95.596(2)
γ/°	90	111.865(2)
Volume/Å^3	2797.55(5)	3100.81(14)
Z	2	2
ρ_{calc}g/cm^3	1.562	1.356
μ/mm^{-1}	4.751	3.963
F(000)	1356.0	1349.0
Crystal size/mm^3	0.141 × 0.078 × 0.052	0.576 × 0.151 × 0.086
Radiation	CuKα (λ = 1.54184)	CuKα (λ = 1.54184)
2Θ range for data collection/°	8.244 to 152.488	7.44 to 152.722
Index ranges	-15 ≤ h ≤ 15, -25 ≤ k ≤ 26, -15 ≤ l ≤ 15	-16 ≤ h ≤ 15, -16 ≤ k ≤ 16, -25 ≤ l ≤ 25
Reflections collected	33333	63194
Independent reflections	11130 [R_{int} = 0.0321, R_{sigma} = 0.0338]	12834 [R_{int} = 0.0651, R_{sigma} = 0.0395]
Data/restraints/parameters	11130/1/715	12834/666/863
Goodness-of-fit on F^2	1.013	1.026
Final R indexes [I>=2σ (I)]	R_1 = 0.0268, wR_2 = 0.0631	R_1 = 0.0651, wR_2 = 0.1739
Final R indexes [all data]	R_1 = 0.0276, wR_2 = 0.0634	R_1 = 0.0845, wR_2 = 0.1901
Largest diff. peak/hole / e Å^{-3}	1.03/-0.65	0.68/-0.52
CCDC	2434061	2434058

Identification code	$[K(crypt)]_2[S_2O_6]$
Empirical formula	$C_{18}H_{36}KN_2O_9S$
Formula weight	495.65
Temperature/K	150.00(10)
Crystal system	trigonal
Space group	R-3
a/Å	12.0247(2)
b/Å	12.0247(2)
c/Å	28.1520(5)
α/°	90
β/°	90
γ/°	120
Volume/Å^3	3525.24(13)
Z	6
ρ_{calc}g/cm^3	1.401
μ/mm^{-1}	3.250
F(000)	1590.0
Crystal size/mm^3	0.337 × 0.158 × 0.06
Radiation	Cu Kα (λ = 1.54184)
2Θ range for data collection/°	9.054 to 152.034
Index ranges	-15 ≤ h ≤ 15, -15 ≤ k ≤ 15, -35 ≤ l ≤ 33
Reflections collected	22210
Independent reflections	1638 [R_{int} = 0.0763, R_{sigma} = 0.0263]
Data/restraints/parameters	1638/0/94
Goodness-of-fit on F^2	1.056
Final R indexes [I>=2σ (I)]	R_1 = 0.0350, wR_2 = 0.0968
Final R indexes [all data]	R_1 = 0.0454, wR_2 = 0.1034
Largest diff. peak/hole / e Å^{-3}	0.39/-0.45
CCDC	2434057

10. Density Functional Theory

10.1. Computational Methods for Infrared Studies

Density Functional Theory (DFT) calculations to aid the identification of IR stretches were performed with the Gaussian 09 program package (version g09, rev.d01).[24] Geometry optimisation and frequency calculations were performed using the b3lyp functional with the def2-TZVP basis set for all atoms.[25] No symmetry constraints were applied during optimisation. All minima were confirmed by the absence of imaginary frequencies. Initial geometries were prepared using X-ray diffraction coordinates where available and Facio V22.1.1.64 software. Calculated IR spectra were visualized using GaussView 5.0.

10.2. Computational Methods Mechanistic Studies

These DFT calculations were performed using the Gaussian 16 suite of programmes, revision C.01.[26] Following extensive benchmarking, the mn15 functional was used throughout,[27] along with the def2-TZVP basis set on all atoms.[28] The superfine integration grid was used, and the influence of the solvent was modelled using the PCM with parameters appropriate to *N*,*N*-dimethylformamide. Free energies were computed using entropic contributions determined from frequency calculations and adjusted using the quasi-harmonic approximation proposed by Grimme.[29] Energetic Span Model analyses were conducted using the AUTOF program developed by Kozuch and Shaik.[30]

The Energetic Span Model (ESM) suggests that rate-determining (RD) states, instead of a single RD-step, provide a more accurate interpretation of the kinetics in catalytic cycles.[31] The TOF-determining transition states (TDTS) and TOF-determining intermediates (TDI) collectively gauge the apparent activation energy and determine the kinetics and TOF of the catalytic reaction. In addition, the degree of TOF control (X_{TOF}) indicates the influence of the corresponding state, either transition state or intermediate, on the overall TOF. Therefore, the TDI and TDTS can be identified by larger X_{TOF} values. It is worth noting that within this framework the TDTS is not necessarily the highest energy state, and multiple states may affect the kinetics. The

results of the ESM analysis for both the [**5**]$^{3-}$ and [**6**]$^{3-}$ cycles are given in Table S12 In both cases the TDTS is **TS1**, and the TDI is **I2**. Also given are the relative TOFs for [**5**]$^{3-}$ and [**6**]$^{3-}$ cycles – that for the As system is significantly larger than for the P, in qualitative agreement with experiment.

Table S10: Relative Gibbs (electronic) energies (kcal/mol) of the $[As_7]^{3-}$ analogue of the $[P_7]^{3-}$ cycle (see left hand side of Figure 3b of main paper and Figure S157).

[5]$^{3-}$-As – TS1-As	31.3 (21.9)
TS1-As – I1'-As	-91.9 (-81.4)
I1'-As – I1'-HBPin-As	-18.8 (-33.4)
I1'-HBPin-As – TS2-As	21.2 (23.1)
TS2-As – I2-As	-53.3 (-39.3)
I2-As – TS3-As	21.4 (7.2)
TS3-As – [5]$^{3-}$-As	-17.9 (-2.0)

Table S11: Relative Gibbs (electronic) energies (kcal/mol) of the $[As_7]^{3-}$ analogue of the stepwise reactions of $[P_7]^{3-}$ with N_2O (see Figure 3b of main paper and Figure S158).

[5]$^{3-}$-As – TS1-As	31.3 (21.9)
TS1-As – I1'-As	-91.9 (-81.4)
I1'-As – TS4-As	35.4 (25.6)
TS4-As – I1''-As	-91.6 (-82.0)
I1''-As – TS5-As	34.2 (26.1)
TS5-As – I1'''-As	-94.8 (-84.3)

Table S12: Degree of TOF control (X_{TOF}) for intermediates and transition states, and relative overall TOF, for the $[P_7]^{3-}$- and $[As_7]^{3-}$-catalyzed reduction of N_2O (see left hand side of Figure 3b of main paper and Figure S157).

	P_7^{3-} cycle		As_7^{3-} cycle	
Step	$X_{TOF, I}$	$X_{TOF, T}$	$X_{TOF, I}$	$X_{TOF, T}$
$[5]^{3-}$ – TS1	0.00	**1.00**	0.00	**1.00**
I1' – I1'-HBPin	0.00	0.00	0.00	0.00
I1'-HBPin – TS2	0.00	0.00	0.00	0.00
I2 – TS3	**1.00**	0.00	**1.00**	0.00
Relative TOF (298.15 K)	1		67.8	

Figure S157: Computed mechanism for the [**5**]$^{3-}$-catalyzed reduction of N_2O (see left hand side of Figure 3b of main paper). Gibbs energies (kcal/mol) and, in parentheses, electronic energies are given for the individual steps. Imaginary wavenumbers are given for the transition states.

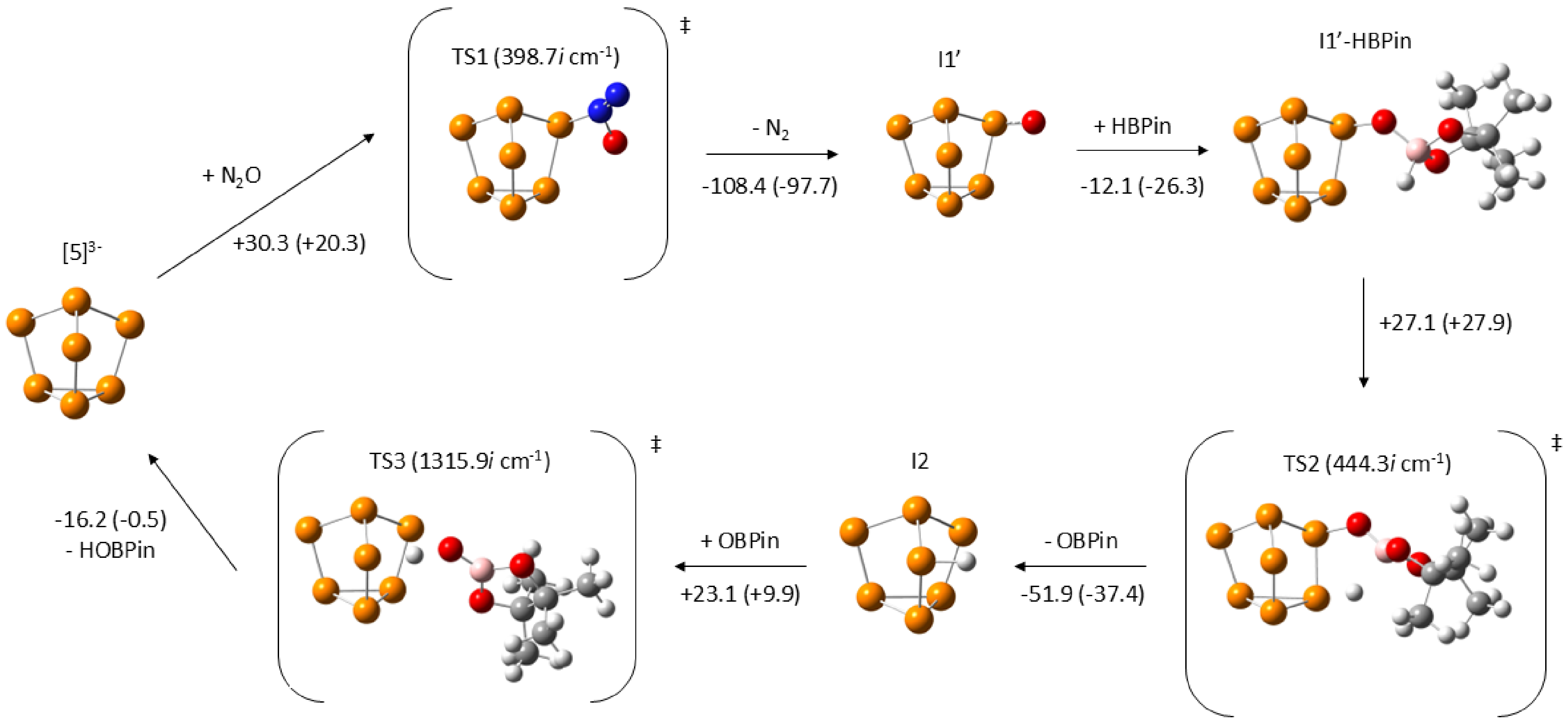

Figure S158: Computed mechanism for the off-cycle reaction of N_2O with **I1’** (Pn = P; see Figure 3b of main paper). Gibbs energies (kcal/mol) and, in parentheses, electronic energies are given for the individual steps. Imaginary wavenumbers are given for the transition states.

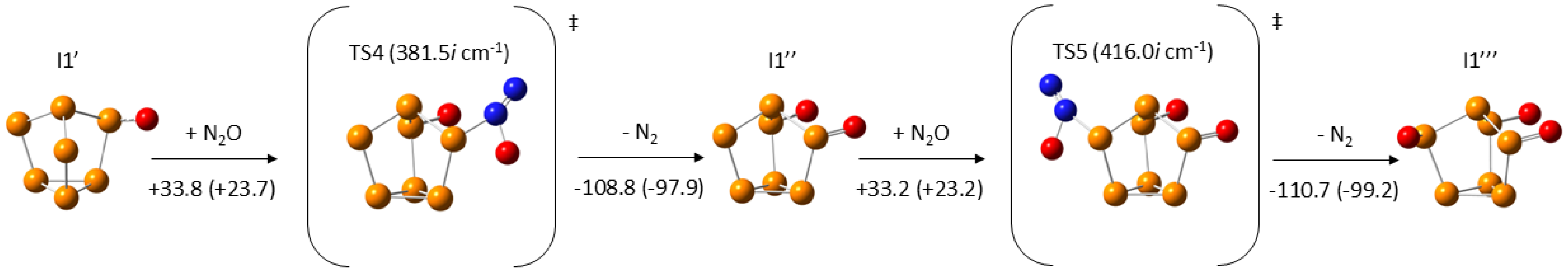

11. References

(1) Cicač-Hudi, M.; Bender, J.; Schlindwein, S. H.; Bispinghoff, M.; Nieger, M.; Grützmacher, H.; Gudat, D. Direct Access to Inversely Polarized Phosphaalkenes from Elemental Phosphorus or Polyphosphides. *Eur. J. Inorg. Chem.* **2016**, *2016* (5), 649–658.

(2) Jobbins, W. D.; van IJzendoorn, B.; Vitorica-Yrezabal, I. J.; Whitehead, G. F. S.; Mehta, M. Reactivity of Tetrel Functionalized Heptapnictogen Clusters Towards Heteroallenes. *Dalton Trans.* **2023**, *52* (8), 2384–2391.

(3) a) Turbervill, R. S. P.; Goicoechea, J. M. Hydrophosphination of Carbodiimides Using Protic Heptaphosphide Cages: A Unique Effect of the Bimodal Activity of Protonated Group 15 Zintl Ions. *Organometallics* **2012**, *31* (6), 2452–2462. b) Goicoechea, J. M. Hydropnictination Reactions of Carbodiimides and Isocyanates with Protonated Heptaphosphide and Heptaarsenide Zintl Ions. *Eur. J. Inorg. Chem.* **2014**, *2014* (10), 1660–1668.

(4) van IJzendoorn, B.; Albawardi, S. F.; Vitorica-Yrezabal, I. J.; Whitehead, G. F. S.; McGrady, J. E.; Mehta, M. A Zintl Cluster for Transition Metal-Free Catalysis: C═O Bond Reductions. *J. Am. Chem. Soc.* **2022**, *144* (46), 21213–21223.

(5) Knapp, C.; Zhou, B.; Denning, M. S.; Rees, N. H.; Goicoechea, J. M. Reactivity Studies of Group 15 Zintl Ions Towards Homoleptic Post-Transition Metal Organometallics: A 'Bottom-Up' Approach to Bimetallic Molecular Clusters. *Dalton Trans.* **2010**, *39* (2), 426–436.

(6) van IJzendoorn, B.; Albawardi, S. F.; Jobbins, W. D.; Whitehead, G. F. S.; McGrady, J. E.; Mehta, M. Transforming Carbon Dioxide into a Methanol Surrogate Using Modular Transition Metal-Free Zintl Ions. *Nat. Commun.* **2024**, *15* (1), 10030.

(7) Espinal-Viguri, M.; Neale, S. E.; Coles, N. T.; Macgregor, S. A.; Webster, R. L. Room Temperature Iron-Catalyzed Transfer Hydrogenation and Regioselective Deuteration of Carbon–Carbon Double Bonds. *J. Am. Chem. Soc.* **2019**, *141* (1), 572–582.

(8) Sheldrick, G. M. *Acta. Cryst.* **2015**, *A71*, 3–8. Dolomanov, O. V.; Bourhis, L. J.; Gildea, R. J.; Howard, J. A. K.; Puschmann, H. OLEX2: A Complete Structure Solution, Refinement and Analysis Program. *J. Appl. Cryst.* **2009**, *42*, 339–341.

(9) Turbervill, R. Solution Reactivity Studies of Group 15 Zintl Anions Towards Unsaturated Substrates. Oxford University, UK, 2014.

(10) Baudler, M. Chain and Ring Phosphorus Compounds—Analogies between Phosphorus and Carbon Chemistry. *Angew. Chem. Int. Ed. Eng.* **1982**, *21* (7), 492–512.

(11) Pang, Y.; Leutzsch, M.; Nöthling, N.; Cornella, J. Catalytic Activation of N_2O at a Low-Valent Bismuth Redox Platform. *J. Am. Chem. Soc.* **2020**, *142* (46), 19473–19479.

(12) Lei, B.; Cao, F.; Chen, M.; Wang, X.; Mo, Z. Bisgermylene-Stabilized Stannylone: Catalytic Reduction of Nitrous Oxide and Nitro Compounds via Element-Ligand Cooperativity. *J. Am. Chem. Soc.* **2024**, *146* (26), 17817–17826.

(13) Anthore-Dalion, L.; Nicolas, E.; Cantat, T. Catalytic Metal-Free Deoxygenation of Nitrous Oxide with Disilanes. *ACS Catalysis* **2019**, *9* (12), 11563–11567.

(14) Chen, X.; Wang, H.; Du, S.; Driess, M.; Mo, Z. Deoxygenation of Nitrous Oxide and Nitro Compounds Using Bis(N-Heterocyclic Silylene)Amido Iron Complexes as Catalysts. *Angew. Chem. Int. Ed.* **2022**, *61* (7), e202114598.

(15) Bermejo, J.; Ortega-Lepe, I.; Santos, L. L.; Rendón, N.; López-Serrano, J.; Álvarez, E.; Suárez, A. Nitrous Oxide Activation by Picoline-Derived Ni–CNP Hydrides. *Chem. Commun.* **2024**, *60* (12), 1575–1578.

(16) Hood, T. M.; Charman, R. S. C.; Liptrot, D. J.; Chaplin, A. B. Copper(I) Catalysed Diboron(4) Reduction of Nitrous Oxide. *Angew. Chem. Int. Ed.* **2024**, *63* (45), e202411692.

(17) Zeng, R.; Feller, M.; Ben-David, Y.; Milstein, D. Hydrogenation and Hydrosilylation of Nitrous Oxide Homogeneously Catalyzed by a Metal Complex. *J. Am. Chem. Soc.* **2017**, *139* (16), 5720–5723.

(18) Jurt, P.; Abels, A. S.; Gamboa-Carballo, J. J.; Fernández, I.; Le Corre, G.; Aebli, M.; Baker, M. G.; Eiler, F.; Müller, F.; Wörle, M.; et al. Reduction of Nitrogen Oxides by Hydrogen with Rhodium(I)–Platinum(II) Olefin Complexes as Catalysts. *Angew. Chem. Int. Ed.* **2021**, *60* (48), 25372–25380.

(19) Ortega-Lepe, I.; Sánchez, P.; Santos, L. L.; Lara, P.; Rendón, N.; López-Serrano, J.; Salazar-Pereda, V.; Álvarez, E.; Paneque, M.; Suárez, A. Catalytic Nitrous Oxide Reduction with H_2 Mediated by Pincer Ir Complexes. *Inorg. Chem.* **2022**, *61* (46), 18590–18600.

(20) Nappen, S. T.; Gamboa-Carballo, J. J.; Tschanen, E.; Ricatto, F.; Wörle, M. D.; Thomas, A.; Trincado, M.; Grützmacher, H. Water Serving as Cocatalyst for the Highly Efficient Homogeneously Catalyzed Conversion of N_2O/H_2 Mixtures with Optimized Rhodium NHC Complexes. *Angew. Chem. Int. Ed.* **2025**, *64* (25), e202502616.

(21) Baudler, M.; Floruss, A. Beiträge zur Chemie des Phosphors. 233. $Li_3P_7O_3$ und $Li_2HP_7O_2$ – die ersten Oxido-heptaphosphane(3). *Z. Anorg. Allg. Chem.* **1995**, *621* (2), 207–212.

(22) a) Denehy, E.; White, J. M.; Williams, S. J. Ground State Structures of Sulfate Monoesters and Sulfamates Reveal Similar Reaction Coordinates for Sulfuryl and Sulfamyl Transfer. *Chem. Commun.* **2006**, (3), 314–316. b) Conway, L. P.; Mikkola, S.; O'Donoghue, A. C.; Hodgson, D. R. W. The Synthesis, Conformation and Hydrolytic Stability of an N,S-bridging Thiophosphoramidate Analogue of Thymidylyl-3′,5′-thymidine. *Org. Biomol. Chem.* **2016**, *14* (30), 7361–7367. c) Dutov, M. D.; Serushkina, O. V.; Shevelev, S. A. New Procedure for Nucleophilic Sulfonation of Aromatic Nitro Compounds: Destructive Oxidation of S-arylthioglycolic Acids Esters. *Russ. J. Org. Chem.* **2007**, *43* (8), 1167–1169. d) Burlingham, B. T.; Pratt, L. M.; Davidson, E. R.; Shiner, V. J.; Fong, J.; Widlanski, T. S. 34S Isotope Effect on Sulfate Ester Hydrolysis: Mechanistic Implications. *J. Am. Chem. Soc.* **2003**, *125* (43), 13036–13037.

(23) Gudun, K. A.; Zakarina, R.; Segizbayev, M.; Hayrapetyan, D.; Slamova, A.; Khalimon, A. Y. Cobalt-Catalyzed Deoxygenative Hydroboration of Nitro Compounds and Applications to One-Pot Synthesis of Aldimines and Amides. *Adv. Synth. Catal.* **2022**, *364* (3), 601–611.
(24) a) Hohenberg, P.; Kohn, W. Inhomogeneous Electron Gas. *Phys. Rev.* **1964**, *136* (3B), B864–B871. b) Kohn, W.; Sham, L. J. Self-Consistent Equations Including Exchange and Correlation Effects. *Phys. Rev.* **1965**, *140* (4A), A1133–A1138. c) Peng, C.; Ayala, P. Y.; Schlegel, H. B.; Frisch, M. J. Using Redundant Internal Coordinates to Optimize Equilibrium Geometries and Transition States. *J. Comput. Chem.* **1996**, *17* (1), 49–56. d) Gaussian 09, R. A., M. J. Frisch, G. W. Trucks, H. B. Schlegel, G. E. Scuseria, M. A. Robb, J. R. Cheeseman, G. Scalmani, V. Barone, G. A. Petersson, H. Nakatsuji, X. Li, M. Caricato, A. Marenich, J. Bloino, B. G. Janesko, R. Gomperts, B. Mennucci, H. P. Hratchian, J. V. Ortiz, A. F. Izmaylov, J. L. Sonnenberg, D. Williams-Young, F. Ding, F. Lipparini, F. Egidi, J. Goings, B. Peng, A. Petrone, T. Henderson, D. Ranasinghe, V. G. Zakrzewski, J. Gao, N. Rega, G. Zheng, W. Liang, M. Hada, M. Ehara, K. Toyota, R. Fukuda, J. Hasegawa, M. Ishida, T. Nakajima, Y. Honda, O. Kitao, H. Nakai, T. Vreven, K. Throssell, J. A. Montgomery, Jr., J. E. Peralta, F. Ogliaro, M. Bearpark, J. J. Heyd, E. Brothers, K. N. Kudin, V. N. Staroverov, T. Keith, R. Kobayashi, J. Normand, K. Raghavachari, A. Rendell, J. C. Burant, S. S. Iyengar, J. Tomasi, M. Cossi, J. M. Millam, M. Klene, C. Adamo, R. Cammi, J. W. Ochterski, R. L. Martin, K. Morokuma, O. Farkas, J. B. Foresman, and D. J. Fox, Gaussian, Inc., Wallingford CT, 2016.
(25) a) Perdew, J. P.; Burke, K.; Ernzerhof, M. Generalized Gradient Approximation Made Simple. *Phys. Rev. Lett.* **1996**, *77* (18), 3865–3868. b) Perdew, J. P.; Ernzerhof, M.; Burke, K. Rationale for Mixing Exact Exchange with Density Functional Approximations. *J. Chem. Phys.* **1996**, *105* (22), 9982–9985. c) Krishnan, R.; Binkley, J. S.; Seeger, R.; Pople, J. A. Self-Consistent Molecular Orbital Methods. XX. A Basis Set for Correlated Wave Functions. *J. Chem. Phys.* **1980**, *72* (1), 650–654.
(26) Gaussian 16 Revision C.01; M. J. Frisch, G. W. T., H. B. Schlegel, G. E. Scuseria, M. A. Robb, J. R. Cheeseman, G. Scalmani, V. Barone, G. A. Petersson, H. Nakatsuji, X. Li, M. Caricato, A. V. Marenich, J. Bloino, B. G. Janesko, R. Gomperts, B. Mennucci, H. P. Hratchian, J. V. Ortiz, A. F. Izmaylov, J. L. Sonnenberg, D. Williams-Young, F. Ding, F. Lipparini, F. Egidi, J. Goings, B. Peng, A. Petrone, T. Henderson, D. Ranasinghe, V. G. Zakrzewski, J. Gao, N. Rega,; G. Zheng, W. L., M. Hada, M. Ehara, K. Toyota, R. Fukuda, J. Hasegawa, M. Ishida, T. Nakajima, Y. Honda, O. Kitao, H. Nakai, T. Vreven, K. Throssell, J. A. Montgomery, Jr., J. E. Peralta, F. Ogliaro, M. J. Bearpark, J. J. Heyd, E. N. Brothers, K. N. Kudin, V. N. Staroverov, T. A. Keith, R. Kobayashi, J. Normand, K. Raghavachari, A. P. Rendell, J. C. Burant, S. S. Iyengar, J. Tomasi, M. Cossi, J. M. Millam, M. Klene, C. Adamo, R. Cammi, J. W. Ochterski, R. L. Martin, K. Morokuma, O. Farkas, J. B. Foresman, and D. J. Fox, Gaussian, Inc., Wallingford CT, 2019.
(27) Yu, H. S.; He, X.; Li, S. L.; Truhlar, D. G. MN15: A Kohn–Sham Global-Hybrid Exchange–Correlation Density Functional with Broad Accuracy for Multi-Reference and Single-Reference Systems and Noncovalent Interactions. *Chem. Sci.* **2016**, *7* (8), 5032–5051.
(28) Weigend, F. Accurate Coulomb-Fitting Basis Sets for H to Rn. *Phys. Chem. Chem. Phys.* **2006**, *8* (9), 1057–1065.
(29) Grimme, S. Supramolecular Binding Thermodynamics by Dispersion-Corrected Density Functional Theory. *Chem. Eur. J.* **2012**, *18* (32), 9955–9964.
(30) Uhe, A.; Kozuch, S.; Shaik, S. Automatic Analysis of Computed Catalytic Cycles. *J. Comput. Chem.* **2011**, *32* (5), 978–985.
(31) a) Kozuch, S.; Shaik, S. How to Conceptualize Catalytic Cycles? The Energetic Span Model. *Acc. Chem. Res.* **2011**, *44* (2), 101–110. b) Kozuch, S.; Martin, J. M. L. The Rate-Determining Step is Dead. Long Live the Rate-Determining State! *ChemPhysChem* **2011**, *12* (8), 1413–1418. c) Kozuch, S. A Refinement of Everyday Thinking: The Energetic Span Model for Kinetic Assessment of Catalytic Cycles. *WIREs Comput. Mol. Sci.* **2012**, *2* (5), 795–815. d) Amatore, C.; Jutand, A. Mechanistic and Kinetic Studies of Palladium Catalytic Systems. *J. Organomet. Chem.* **1999**, *576* (1), 254–278.
